# Whole genome sequence analysis of BT-474 using complete Genomics’ standard and long fragment read technologies

**DOI:** 10.1186/s13742-016-0113-x

**Published:** 2016-02-09

**Authors:** Serban Ciotlos, Qing Mao, Rebecca Yu Zhang, Zhenyu Li, Robert Chin, Natali Gulbahce, Sophie Jia Liu, Radoje Drmanac, Brock A. Peters

**Affiliations:** Complete Genomics, Inc., 2071 Stierlin Court, Mountain View, CA 94043 USA; BGI-Shenzhen, Shenzhen, 518083 China

**Keywords:** Long Fragment Read, Complete Genomics, BT-474, BT474, Whole genome sequencing, Breast cancer

## Abstract

**Background:**

The cell line BT-474 is a popular cell line for studying the biology of cancer and developing novel drugs. However, there is no complete, published genome sequence for this highly utilized scientific resource. In this study we sought to provide a comprehensive and useful data set for the scientific community by generating a whole genome sequence for BT-474.

**Findings:**

Five μg of genomic DNA, isolated from an early passage of the BT-474 cell line, was used to generate a whole genome sequence (114X coverage) using Complete Genomics’ standard sequencing process. To provide additional variant phasing and structural variation data we also processed and analyzed two separate libraries of 5 and 6 individual cells to depths of 99X and 87X, respectively, using Complete Genomics’ Long Fragment Read (LFR) technology.

**Conclusions:**

BT-474 is a highly aneuploid cell line with an extremely complex genome sequence. This ~300X total coverage genome sequence provides a more complete understanding of this highly utilized cell line at the genomic level.

**Electronic supplementary material:**

The online version of this article (doi:10.1186/s13742-016-0113-x) contains supplementary material, which is available to authorized users.

## Data description

### Utility of the dataset

The cell line BT-474 was isolated by Lasfargues et al. [1] in 1978, from a biopsy of invasive ductal carcinoma from a 60 year old Caucasian female. Since that time it has become one of the most heavily utilized cell lines for breast cancer research. At the time of writing, entering the search term “BT-474 OR BT474” into PubMed resulted in 973 unique articles. Surprisingly, the complete genome sequence of this cell line has yet to be published. In this paper, we fill that void in the collective scientific knowledge by providing high coverage whole genome data for BT-474.

Previous studies have shown that BT-474 has a modal number of chromosomes approximating tetraploidy, and most of these chromosomes are covered with megabase-sized amplifications, deletions, and other structural rearrangements [2]. In an effort to provide better coverage of these complex rearranged regions, and to provide variant phasing and error correcting information, we generated high coverage libraries from long genomic DNA (~40 kb) using Long Fragment Read (LFR) technology [3, 4], and supplemented those libraries with a standard (STD) short mate pair library (~500 bp) [5] for a combined total coverage of over ~300X. We hope the freely available resource provided in this paper will benefit our understanding of the biology of cancer, and ultimately help to improve therapies for patients.

### Library generation

DNA was isolated from low passage number BT-474 cells, procured from the American Type Culture Collection (ATCC, Manassas, VA, USA), using a RecoverEase dialysis kit (Agilent, Santa Clara, CA, USA). This material was further fragmented to 300–800 base pairs using a Covaris E220 (Covaris, Woburn, MA, USA), and processed using Complete Genomics’ proprietary standard library construction [5]. For LFR libraries, approximately 5 cells were collected and deposited into a 1.5 ml microtube with 10 μl of distilled water. Cells were lysed, and DNA was denatured using 1 μl of 20 mM KOH and 0.5 mM EDTA. Denatured genomic DNA was dispersed across a 384-well plate. In each well, long genomic fragments (~40 kb) were amplified, fragmented, and tagged with a unique barcode adapter as previously described [3]. All libraries were sequenced using Complete Genomics’ nanoarray sequencing platform [5].

### BT-474 genome analysis

**Fig. 1:**
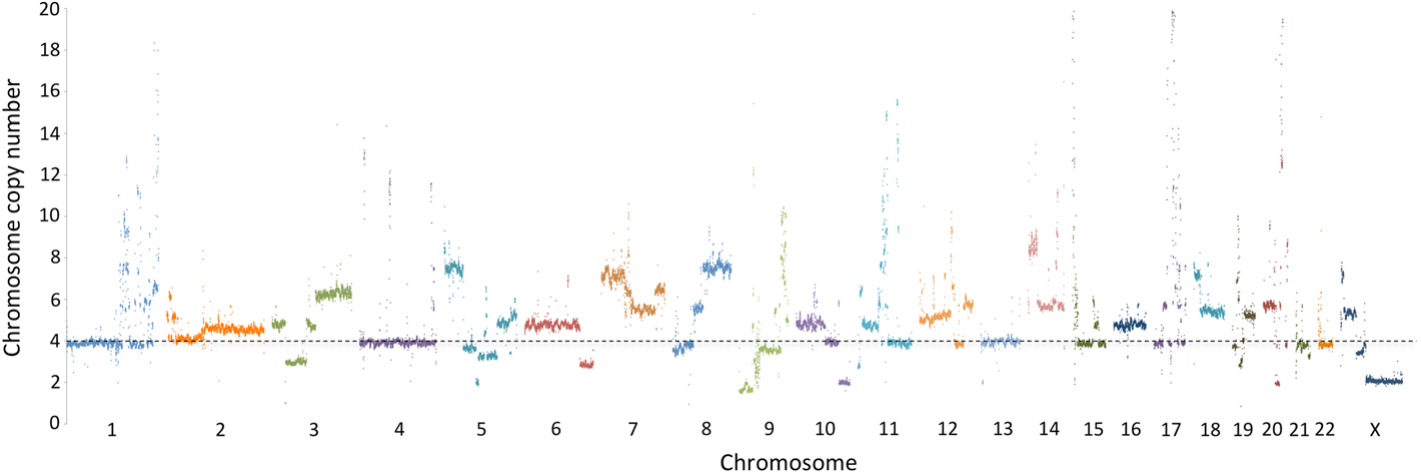
100 kb read coverage. For the standard library of BT-474 reads were averaged across consecutive 100 kb bins, normalized to a tetraploid copy number, and plotted such that each dot represents the coverage of a single 100 kb region of the genome. Y-axis shows haploid copy number; x-axis shows genome position increasing from left to right for chromosome and position

**Fig. 2:**
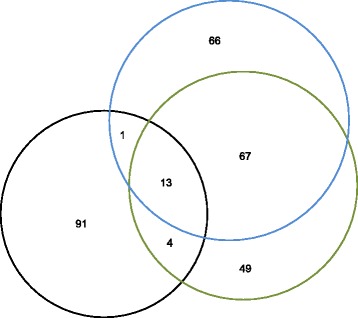
Overlap of inter-chromosomal translocations between libraries. Interchromosomal translocations were called for each library. The overlap between libraries was determined by considering translocations found within 5 kb of each other, and in the same orientation, to be the same event. This also resulted in the aggregation of multiple close translocations within the same sample into a single event. In total, 109 translocations were found in the standard library (black), and 147 and 133 were found in LFR libraries 1 (blue) and 2 (green), respectively. Of these, 85 interchromosomal translocations are shared between at least two libraries

Read data of 343, 298, and 261 Gb from the STD, LFR1, and LFR2 libraries, respectively, were mapped to the NCBI human reference genome (build 37) using Complete Genomics’ pipeline [3, 5, 6] (Table 1), resulting in close to ~100X coverage in each of the libraries. The high coverage allowed more than 90 % of the genome and exome of each library to be called (Table 1.). Plotting reads falling within 100 kb consecutive windows for the BT-474 standard library resulted in the expected complex pattern of amplifications affecting almost all chromosomes [2] (Fig. 1). Known amplifications of ERBB2 and the HOX gene cluster on chromosome 17 [2] are readily identifiable from this plot, as well as many other megabase-sized highly amplified regions. Analysis of both standard and LFR libraries resulted in the discovery of 110, 175, and 145 interchromosomal translocations in the STD, LFR1, and LFR2 libraries, respectively (Table 2). Clustering these translocations, based on windows of 5 kb around the breakpoints, led to the overlap of many translocations within and between libraries, and an overall reduction in the total number of translocations to 291 (Table 2 and Fig. 2). Additionally, comparing our results to a published RNA sequencing analysis of BT-474 [7, 8] demonstrated that three of the five coding interchromosomal translocations were called in our data (Table 3). In the remaining two translocations that were not called by our algorithms, raw reads were found to support their existence in our libraries; for the *STARD3-DOK5* translocation, improved algorithms would most likely detect this event. In the case of the *TRPC4AP-MRPL45* translocation only one mate pair read in the STD library was found in support, making it unlikely to have been called even with modifications to our algorithms.

**Table 1:** BT-474 genome statistics

Metric	BT-474 STD	BT-474 LFR1	BT-474 LFR2
Fully called genome fraction	0.972	0.915	0.900
Fully called exome fraction	0.988	0.928	0.920
Gross mapping yield (Gb)	343	298	261
Both mates mapped yield (Gb)	306	217	171
Genome fraction with sequence coverage ≥ 5x	0.997	0.981	0.978
Exome fraction with sequence coverage ≥ 5x	0.999	0.980	0.980
SNV total count	3,241,932	2,856,624	2,890,506
Homozygous SNV count	1,531,723	1,382,653	1,241,444
Heterozygous SNV count	1,635,402	1,195,290	1,239,735
Het/Hom ratio	1.07	0.86	1.00
ENA sample accession number	ERS823996	ERS823998	ERS823997

STD and LFR libraries were mapped to the NCBI reference genome build 37. An explanation of the genome statistics are as follows: fully called genome fraction, the fraction of the genome for which both alleles at each position are confidently called; fully called exome fraction, the fraction of the coding part of the genome for which both alleles are confidently called; gross mapping yield (Gb), the number of gigabases of read data that can be mapped to NCBI reference genome build 37; both mates mapped yield (Gb), the number of gigabases of read data where both arms of a mate pair can be mapped to NCBI reference genome build 37; genome fraction with sequence coverage ≥ 5x, fraction of the genome with at least 5 reads covering a single position; exome fraction with sequence coverage ≥ 5x, fraction of the coding genome with at least 5 reads covering a single position; SNV total count, the total number of single nucleotide variants called in each library; homozygous SNV count, the total number of homozygous variants called in each library; heterozygous SNV count, the total number of heterozygous variants called in each library; Het/Hom ratio, the ratio of heterozygous variants over homozygous variants (this number is typically around 1.6 for a Caucasian genome); ENA sample accession number, the European Nucleotide Archive accession number to locate the raw read data for each library

**Table 2:** Potential translocations identified in BT-474

SV ID	chrA	chrA start	chrA end	chrB	chrB start	chrB end	Breakpoint orientation	Library
1	chr1	5715019	5715320	chr12	34295113	34295399	rf	LFR1
2	chr1	2.32E + 08	2.32E + 08	chr14	1.06E + 08	1.06E + 08	fr	STD
3	chr1	1.68E + 08	1.68E + 08	chr14	35524861	35524861	rf	STD
4	chr1	1.54E + 08	1.54E + 08	chr15	71431472	71431472	rf	STD
5	chr1	5735134	5736117	chr16	33921851	33923571	ff	LFR1
5	chr1	5735257	5735628	chr16	33921851	33922429	ff	LFR2
5	chr1	5735793	5735873	chr16	33923066	33923356	ff	LFR2
5	chr1	5735820	5736219	chr16	33922686	33922846	ff	LFR2
5	chr1	5735833	5736106	chr16	33922267	33922440	ff	LFR2
5	chr1	5736076	5736104	chr16	33923217	33923483	ff	LFR2
6	chr1	5735201	5735384	chr16	33922780	33923001	fr	LFR2
6	chr1	5735268	5735489	chr16	33922912	33923205	fr	LFR1
6	chr1	5735623	5736183	chr16	33921696	33922803	fr	LFR1
6	chr1	5735806	5735854	chr16	33921697	33921850	fr	LFR2
6	chr1	5735811	5736104	chr16	33922722	33922916	fr	LFR2
7	chr1	5735044	5735977	chr16	33924087	33924848	rf	LFR2
7	chr1	5735136	5735386	chr16	33924399	33925038	rf	LFR1
7	chr1	5735186	5735521	chr16	33923732	33924006	rf	LFR1
7	chr1	5735651	5736227	chr16	33921710	33922453	rf	LFR1
7	chr1	5735793	5736102	chr16	33923138	33923604	rf	LFR1
7	chr1	5735798	5735892	chr16	33924400	33924690	rf	LFR1
7	chr1	5735825	5736119	chr16	33921857	33922502	rf	LFR2
8	chr1	16979636	16979817	chr3	18950158	18950300	ff	LFR1
9	chr1	33084225	33084414	chr3	1.19E + 08	1.19E + 08	ff	LFR2
9	chr1	33084282	33084474	chr3	1.19E + 08	1.19E + 08	ff	LFR1
10	chr1	16964949	16965018	chr3	49732982	49733419	rr	LFR1
11	chr1	45317764	45317764	chr3	98899084	98899084	rr	STD
12	chr1	1.09E + 08	1.09E + 08	chr3	1.1E + 08	1.1E + 08	rr	LFR2
13	chr1	1.18E + 08	1.18E + 08	chr4	1.85E + 08	1.85E + 08	ff	LFR1
13	chr1	1.18E + 08	1.18E + 08	chr4	1.85E + 08	1.85E + 08	ff	LFR2
13	chr1	1.18E + 08	1.18E + 08	chr4	1.85E + 08	1.85E + 08	ff	STD
14	chr1	1.92E + 08	1.92E + 08	chr4	1.8E + 08	1.8E + 08	ff	STD
15	chr1	2.04E + 08	2.04E + 08	chr4	78668596	78668596	ff	STD
16	chr1	2.04E + 08	2.04E + 08	chr4	79751139	79751139	ff	STD
17	chr1	2.04E + 08	2.04E + 08	chr4	1.81E + 08	1.81E + 08	ff	STD
18	chr1	55705456	55705813	chr4	93827471	93827698	fr	LFR1
19	chr1	1.73E + 08	1.73E + 08	chr4	1.85E + 08	1.85E + 08	fr	LFR2
19	chr1	1.73E + 08	1.73E + 08	chr4	1.85E + 08	1.85E + 08	fr	LFR1
19	chr1	1.73E + 08	1.73E + 08	chr4	1.85E + 08	1.85E + 08	fr	STD
20	chr1	1.77E + 08	1.77E + 08	chr4	11996415	11996415	fr	STD
21	chr1	1.91E + 08	1.91E + 08	chr4	1.8E + 08	1.8E + 08	fr	STD
22	chr1	2.32E + 08	2.32E + 08	chr4	78692986	78692986	fr	STD
23	chr1	2.43E + 08	2.43E + 08	chr4	77435897	77435897	fr	STD
24	chr1	2.44E + 08	2.44E + 08	chr4	1.8E + 08	1.8E + 08	fr	STD
25	chr1	1.18E + 08	1.18E + 08	chr4	76245513	76245513	rf	STD
26	chr1	1.5E + 08	1.5E + 08	chr4	76064378	76064378	rf	STD
27	chr1	1.68E + 08	1.68E + 08	chr4	1.84E + 08	1.84E + 08	rf	LFR2
27	chr1	1.68E + 08	1.68E + 08	chr4	1.84E + 08	1.84E + 08	rf	LFR1
28	chr1	1.73E + 08	1.73E + 08	chr4	1.84E + 08	1.84E + 08	rf	LFR1
28	chr1	1.73E + 08	1.73E + 08	chr4	1.84E + 08	1.84E + 08	rf	LFR2
29	chr1	1.9E + 08	1.9E + 08	chr4	1.81E + 08	1.81E + 08	rf	STD
30	chr1	1.96E + 08	1.96E + 08	chr4	1.78E + 08	1.78E + 08	rf	STD
31	chr1	2.38E + 08	2.38E + 08	chr4	10923854	10923854	rf	STD
32	chr1	2.47E + 08	2.47E + 08	chr4	1.84E + 08	1.84E + 08	rf	STD
33	chr1	1.6E + 08	1.6E + 08	chr4	1.84E + 08	1.84E + 08	rr	LFR2
33	chr1	1.6E + 08	1.6E + 08	chr4	1.84E + 08	1.84E + 08	rr	LFR1
34	chr1	1.68E + 08	1.68E + 08	chr4	1.81E + 08	1.81E + 08	rr	STD
34	chr1	1.68E + 08	1.68E + 08	chr4	1.81E + 08	1.81E + 08	rr	LFR2
35	chr1	2.03E + 08	2.03E + 08	chr4	1.86E + 08	1.86E + 08	rr	STD
36	chr1	2.12E + 08	2.12E + 08	chr4	11068353	11068353	rr	STD
37	chr1	2.47E + 08	2.47E + 08	chr4	76847438	76847438	rr	STD
38	chr1	24121289	24121629	chr6	33332915	33333041	rr	LFR1
39	chr1	2.46E + 08	2.46E + 08	chr6	15316532	15316532	rr	STD
40	chr1	39932050	39932711	chr7	1.28E + 08	1.28E + 08	fr	LFR2
41	chr1	2.33E + 08	2.33E + 08	chr8	1.09E + 08	1.09E + 08	rr	STD
42	chr1	86398799	86398985	chr9	1.01E + 08	1.01E + 08	fr	LFR1
43	chr1	86398798	86398975	chr9	1.01E + 08	1.01E + 08	rf	LFR1
43	chr1	86398885	86398990	chr9	1.01E + 08	1.01E + 08	rf	LFR2
44	chr1	91852797	91853069	chrX	1.08E + 08	1.08E + 08	fr	LFR2
45	chr10	37308042	37308294	chr11	80339889	80339922	ff	LFR1
46	chr10	37346634	37346708	chr11	80314502	80314529	rr	LFR1
47	chr10	37352123	37352151	chr11	80308433	80308539	rr	LFR1
48	chr10	37358278	37358481	chr11	80304293	80304322	rr	LFR1
49	chr10	91896284	91896284	chr12	48949541	48949541	rf	STD
50	chr10	45423935	45424170	chr15	80737067	80737361	fr	LFR1
51	chr10	14617439	14617862	chr20	48375830	48376358	ff	LFR2
51	chr10	14617482	14617870	chr20	48375829	48376359	ff	LFR1
52	chr10	14617547	14617844	chr20	48375844	48375915	fr	LFR2
52	chr10	14617564	14617697	chr20	48379370	48379617	fr	LFR1
52	chr10	14617692	14617855	chr20	48375863	48376323	fr	LFR1
53	chr10	14617753	14617855	chr20	48375843	48375872	rf	LFR1
54	chr10	14617325	14617830	chr20	48379454	48379795	rr	LFR1
54	chr10	14617328	14617780	chr20	48379543	48379794	rr	LFR2
55	chr10	1.02E + 08	1.02E + 08	chrX	90335650	90335650	fr	STD
56	chr11	90074805	90074805	chr12	1.34E + 08	1.34E + 08	fr	STD
57	chr11	19002870	19003273	chr14	99742364	99742854	fr	LFR1
57	chr11	19003015	19003277	chr14	99742432	99742672	fr	LFR2
58	chr11	20165549	20165739	chr14	99742938	99743430	rf	LFR2
58	chr11	20165552	20165915	chr14	99742909	99743449	rf	LFR1
59	chr11	60484539	60484539	chr15	32224148	32224148	ff	STD
59	chr11	60486401	60486401	chr15	32226056	32226056	ff	STD
60	chr11	356458	357242	chr15	80736711	80737504	fr	LFR1
60	chr11	357798	357987	chr15	80736802	80737555	fr	LFR1
61	chr11	356453	356667	chr16	57422575	57422884	rf	LFR1
62	chr11	69089738	69089738	chr17	15332776	15332776	ff	STD
63	chr11	83028981	83028981	chr17	41486752	41486752	fr	STD
64	chr11	1.14E + 08	1.14E + 08	chr19	13813499	13813499	rf	STD
65	chr11	15224406	15224520	chr19	44055845	44055982	rr	LFR1
66	chr11	7393836	7393836	chr20	21102359	21102359	ff	STD
67	chr11	5902563	5902802	chrX	1.48E + 08	1.48E + 08	rf	LFR1
68	chr12	1.1E + 08	1.1E + 08	chr15	24519744	24519744	rf	STD
69	chr12	452573	452724	chr17	36813705	36813991	ff	LFR1
69	chr12	452611	452663	chr17	36813712	36813886	ff	LFR2
70	chr12	452410	452681	chr17	36813885	36814117	fr	LFR1
71	chr12	185129	185463	chr20	62949947	62950060	ff	LFR1
71	chr12	186282	186515	chr20	62948717	62949064	ff	LFR1
72	chr12	86378558	86378558	chr20	33736935	33736935	rr	STD
73	chr12	6127534	6127658	chr22	17177775	17177979	fr	LFR1
74	chr13	23541983	23542335	chr14	1.05E + 08	1.05E + 08	ff	LFR1
74	chr13	23542172	23542381	chr14	1.05E + 08	1.05E + 08	ff	LFR2
75	chr13	23542100	23542325	chr14	1.05E + 08	1.05E + 08	fr	LFR1
75	chr13	23542240	23542333	chr14	1.05E + 08	1.05E + 08	fr	LFR1
76	chr13	23541989	23542331	chr14	1.05E + 08	1.05E + 08	rf	LFR1
77	chr13	28033720	28033934	chr14	88237607	88237796	rr	LFR1
78	chr13	22378828	22379005	chr17	36680874	36680916	ff	LFR2
78	chr13	22379054	22379054	chr17	36680719	36680719	ff	STD
79	chr13	36547226	36547559	chr17	57361699	57361862	rr	LFR1
80	chr13	49820612	49820612	chr17	74931251	74931251	rr	STD
81	chr13	19275960	19275960	chr18	14358156	14358156	fr	STD
82	chr13	50980006	50980186	chr20	52105309	52105592	ff	LFR1
83	chr13	42998891	42999005	chr20	45810438	45810647	fr	LFR2
84	chr14	20268504	20268687	chr15	22386856	22386940	fr	LFR2
85	chr14	20291830	20291936	chr15	22409820	22410082	fr	LFR1
86	chr14	20268232	20268636	chr15	22386664	22386879	rf	LFR1
86	chr14	20268607	20269066	chr15	22386868	22387058	rf	LFR2
86	chr14	20269074	20269333	chr15	22386858	22387158	rf	LFR1
87	chr14	88711033	88711033	chr15	27026222	27026222	rf	STD
88	chr14	36726853	36726853	chr17	46922038	46922038	rf	STD
88	chr14	36726909	36727476	chr17	46922166	46922208	rf	LFR2
89	chr14	90314501	90314501	chr20	45462878	45462878	rf	STD
90	chr14	31150555	31150749	chr20	53380309	53380666	rr	LFR2
91	chr14	89177518	89177518	chrX	1.27E + 08	1.27E + 08	fr	STD
92	chr15	20485801	20486106	chr16	33376252	33376612	ff	LFR1
93	chr15	80736769	80736829	chr16	86047419	86047441	rf	LFR1
94	chr15	20485845	20486155	chr16	33376088	33376404	rr	LFR1
95	chr15	20563679	20564143	chr17	77680247	77680760	ff	LFR1
96	chr15	20563232	20563428	chr17	77680598	77680764	rf	LFR1
97	chr15	20562710	20563176	chr17	77681355	77681423	rr	LFR1
98	chr15	25765731	25765818	chr20	52374415	52374605	ff	LFR2
99	chr15	24728638	24729126	chr20	52602846	52603206	fr	LFR1
99	chr15	24728764	24728985	chr20	52603050	52603167	fr	LFR2
100	chr15	24836949	24837393	chr20	52608640	52609262	fr	LFR1
100	chr15	24837101	24837400	chr20	52608843	52609205	fr	LFR2
101	chr15	25765047	25765694	chr20	52374394	52374795	rf	LFR2
101	chr15	25765059	25765749	chr20	52374395	52374772	rf	LFR1
102	chr15	25765081	25765289	chr20	52374585	52374639	rr	LFR2
103	chr15	20497712	20497759	chr22	29081791	29081826	fr	LFR1
103	chr15	20498161	20498285	chr22	29081672	29081823	fr	LFR2
104	chr15	73192028	73192028	chrX	11725501	11725501	rf	STD
105	chr16	33687791	33688498	chr17	36686929	36687323	ff	LFR1
105	chr16	33687793	33688628	chr17	36686788	36687070	ff	LFR2
105	chr16	33687820	33688566	chr17	36687287	36687558	ff	LFR2
105	chr16	33688711	33688949	chr17	36686318	36686384	ff	LFR1
105	chr16	33690641	33691077	chr17	36684052	36684801	ff	LFR1
106	chr16	33708218	33708371	chr17	36655897	36655935	ff	LFR2
106	chr16	33709597	33709917	chr17	36654618	36655128	ff	LFR1
107	chr16	33715838	33715930	chr17	36648811	36648948	ff	LFR1
107	chr16	33717810	33718019	chr17	36646876	36646932	ff	LFR1
107	chr16	33722448	33722598	chr17	36642174	36642455	ff	LFR2
108	chr16	33728271	33728527	chr17	36636182	36636239	ff	LFR2
109	chr16	33688406	33688600	chr17	36686656	36686774	fr	LFR1
110	chr16	33687774	33688478	chr17	36686570	36687084	rr	LFR1
110	chr16	33688224	33689111	chr17	36686331	36686789	rr	LFR2
110	chr16	33688921	33689026	chr17	36686074	36686449	rr	LFR1
111	chr16	33722451	33722930	chr17	36641699	36642406	rr	LFR2
111	chr16	33722452	33722565	chr17	36641896	36642074	rr	LFR1
111	chr16	33723574	33723794	chr17	36640643	36640778	rr	LFR1
112	chr16	33731254	33731590	chr17	36632956	36633284	rr	LFR2
113	chr16	33404337	33404694	chr19	33490646	33490733	ff	LFR1
114	chr16	33406683	33406798	chr19	33488693	33489123	rr	LFR1
115	chr16	33950104	33950328	chrX	1.37E + 08	1.37E + 08	rf	LFR2
116	chr17	58459088	58459088	chr19	17536050	17536050	fr	STD
117	chr17	79759265	79759265	chr19	13827948	13827948	rf	STD
118	chr17	35343594	35343697	chr20	55629651	55630116	ff	LFR2
118	chr17	35343595	35343697	chr20	55629656	55630016	ff	LFR1
118	chr17	35344040	35344109	chr20	55630114	55630166	ff	LFR1
119	chr17	37025773	37025975	chr20	50986305	50986555	ff	LFR1
119	chr17	37025774	37025907	chr20	50986270	50986504	ff	LFR2
119	chr17	37026501	37026658	chr20	50986315	50986584	ff	LFR2
119	chr17	37026948	37027140	chr20	50986368	50986424	ff	LFR1
120	chr17	37979129	37979487	chr20	51393212	51393650	ff	LFR2
121	chr17	47200252	47200570	chr20	52670606	52670990	ff	LFR1
121	chr17	47200551	47200581	chr20	52670822	52670946	ff	LFR2
122	chr17	37025773	37026039	chr20	50986244	50986474	fr	LFR2
123	chr17	37308699	37308887	chr20	45998011	45998274	fr	LFR2
124	chr17	37944301	37944887	chr20	56991287	56991886	fr	LFR1
124	chr17	37944305	37944881	chr20	56991325	56991886	fr	LFR2
125	chr17	50126561	50127342	chr20	56659875	56660528	fr	LFR1
125	chr17	50126917	50127317	chr20	56660109	56660527	fr	LFR2
126	chr17	35343665	35344256	chr20	55629529	55630497	rf	LFR1
126	chr17	35343669	35344183	chr20	55629529	55630106	rf	LFR2
127	chr17	36467936	36467959	chr20	56168308	56168561	rf	LFR2
128	chr17	37025771	37026391	chr20	50986245	50986940	rf	LFR1
128	chr17	37025771	37026159	chr20	50986245	50987008	rf	LFR2
129	chr17	37199707	37199886	chr20	53865721	53865808	rf	LFR2
130	chr17	37950137	37950419	chr20	51445007	51445204	rf	LFR2
131	chr17	21549459	21549788	chr20	26078984	26079241	rr	LFR2
132	chr17	35343685	35343955	chr20	55629543	55629778	rr	LFR2
132	chr17	35343764	35343787	chr20	55629689	55629717	rr	LFR1
132	chr17	35344990	35345255	chr20	55630150	55630327	rr	LFR2
133	chr17	37025777	37026095	chr20	50986254	50986852	rr	LFR2
133	chr17	37025782	37026005	chr20	50986252	50986500	rr	LFR1
134	chr17	37308510	37309069	chr20	45999004	45999288	rr	LFR2
134	chr17	37308533	37308943	chr20	45998950	45999287	rr	LFR1
135	chr17	37944405	37944589	chr20	56991699	56991841	rr	LFR2
136	chr17	58448378	58448378	chrX	7718236	7718236	rr	STD
137	chr18	43367885	43367885	chr20	50570359	50570359	ff	STD
138	chr18	14174515	14174667	chr21	15356960	15357387	ff	LFR1
139	chr18	14150833	14151055	chr21	15379674	15379805	rr	LFR1
140	chr18	14174298	14174539	chr21	15357069	15357326	rr	LFR1
141	chr18	14473900	14474154	chr21	15053964	15054043	rr	LFR2
142	chr19	17652120	17652329	chr20	55386003	55386161	ff	LFR2
142	chr19	17652251	17652438	chr20	55386065	55386453	ff	LFR1
143	chr19	17651835	17651994	chr20	55387244	55387326	fr	LFR1
143	chr19	17651858	17652155	chr20	55386129	55386636	fr	LFR1
144	chr19	17217090	17217398	chr20	56890092	56890284	rf	LFR1
145	chr19	17217090	17217570	chr20	56889905	56890286	rr	LFR1
146	chr19	24538478	24538792	chr21	28834528	28834780	rr	LFR1
147	chr19	9948763	9948799	chr22	16103776	16103874	ff	LFR1
148	chr19	9948910	9949090	chr22	16103680	16103777	rf	LFR1
149	chr2	1.88E + 08	1.88E + 08	chr10	84979145	84979145	ff	STD
150	chr2	2.33E + 08	2.33E + 08	chr11	43356771	43356771	fr	STD
151	chr2	3931101	3931427	chr12	1.24E + 08	1.24E + 08	ff	LFR1
151	chr2	3931129	3931361	chr12	1.24E + 08	1.24E + 08	ff	LFR2
151	chr2	3931172	3931453	chr12	1.24E + 08	1.24E + 08	ff	LFR2
152	chr2	3930891	3931629	chr12	1.24E + 08	1.24E + 08	fr	LFR2
152	chr2	3931052	3931629	chr12	1.24E + 08	1.24E + 08	fr	LFR1
153	chr2	3931690	3932147	chr12	1.24E + 08	1.24E + 08	rf	LFR2
154	chr2	3931163	3931384	chr12	1.24E + 08	1.24E + 08	rr	LFR2
155	chr2	49457015	49457015	chr14	32953403	32953403	ff	STD
156	chr2	13550951	13551227	chr15	20593199	20593601	ff	LFR1
157	chr2	13531819	13531839	chr15	20613163	20613338	rr	LFR2
158	chr2	26214538	26214538	chr15	84212788	84212788	rr	STD
159	chr2	57462432	57462432	chr18	40323965	40323965	rr	STD
160	chr2	2.14E + 08	2.14E + 08	chr20	18756222	18756222	rr	STD
161	chr2	95541846	95541846	chr21	9907919	9907919	fr	STD
162	chr2	95546582	95546582	chr21	9903866	9903866	rf	STD
163	chr2	11929934	11929973	chr4	1.62E + 08	1.62E + 08	fr	LFR2
164	chr2	1.33E + 08	1.33E + 08	chr4	1.91E + 08	1.91E + 08	rf	LFR1
165	chr2	1.96E + 08	1.96E + 08	chr6	88117195	88117195	fr	STD
166	chr2	26673663	26673663	chr7	19518115	19518115	rr	STD
167	chr2	1.33E + 08	1.33E + 08	chr8	69218921	69218921	fr	STD
168	chr2	2.13E + 08	2.13E + 08	chr8	31022034	31022034	rf	STD
169	chr2	27549389	27549460	chr9	1.25E + 08	1.25E + 08	ff	LFR2
170	chr2	1.16E + 08	1.16E + 08	chr9	1.31E + 08	1.31E + 08	fr	LFR1
170	chr2	1.16E + 08	1.16E + 08	chr9	1.31E + 08	1.31E + 08	fr	LFR2
171	chr20	46597442	46598072	chrX	1.29E + 08	1.29E + 08	fr	LFR2
171	chr20	46597699	46598021	chrX	1.29E + 08	1.29E + 08	fr	LFR1
172	chr20	52609811	52610360	chrX	1.29E + 08	1.29E + 08	rf	LFR2
173	chr21	27928545	27928545	chrX	65187504	65187504	rr	STD
174	chr3	96420323	96420323	chr13	86760280	86760280	rr	STD
175	chr3	86385874	86386256	chr14	86684733	86685055	ff	LFR1
175	chr3	86385880	86386147	chr14	86684072	86684160	ff	LFR1
175	chr3	86385893	86386230	chr14	86684069	86684158	ff	LFR2
176	chr3	86385872	86385872	chr14	86684054	86684054	rf	STD
176	chr3	86385893	86386680	chr14	86684053	86684588	rf	LFR1
176	chr3	86385899	86386572	chr14	86684058	86684376	rf	LFR2
177	chr3	90045826	90046098	chr14	91586438	91586701	rf	LFR1
177	chr3	90045828	90046105	chr14	91586456	91586703	rf	LFR2
177	chr3	90045832	90045832	chr14	91586507	91586507	rf	STD
178	chr3	75690113	75690113	chr20	62909459	62909459	rf	STD
179	chr3	655747	656029	chr21	9496278	9496583	ff	LFR2
179	chr3	655838	656046	chr21	9496264	9496451	ff	LFR1
180	chr3	75719349	75720145	chr4	1.91E + 08	1.91E + 08	ff	LFR1
180	chr3	75719873	75720142	chr4	1.91E + 08	1.91E + 08	ff	LFR2
180	chr3	75720887	75720942	chr4	1.91E + 08	1.91E + 08	ff	LFR1
180	chr3	75721439	75721741	chr4	1.91E + 08	1.91E + 08	ff	LFR1
181	chr3	75723970	75724039	chr4	1.91E + 08	1.91E + 08	ff	LFR1
182	chr3	75747839	75748224	chr4	1.91E + 08	1.91E + 08	ff	LFR1
183	chr3	75719642	75719870	chr4	1.91E + 08	1.91E + 08	rr	LFR1
183	chr3	75721591	75721650	chr4	1.91E + 08	1.91E + 08	rr	LFR1
184	chr3	75747286	75747286	chr4	1.54E + 08	1.54E + 08	rr	STD
185	chr3	12369884	12370300	chr5	1.4E + 08	1.4E + 08	ff	LFR1
186	chr3	86387776	86387933	chr5	1.07E + 08	1.07E + 08	fr	LFR2
187	chr3	1.73E + 08	1.73E + 08	chr5	1.6E + 08	1.6E + 08	rr	STD
188	chr3	1.11E + 08	1.11E + 08	chr8	1.29E + 08	1.29E + 08	rf	LFR2
188	chr3	1.11E + 08	1.11E + 08	chr8	1.29E + 08	1.29E + 08	rf	LFR1
189	chr4	3635640	3636110	chr12	54478622	54478665	fr	LFR2
190	chr4	85990622	85990622	chr13	62513878	62513878	ff	STD
191	chr4	76990685	76990685	chr18	38126235	38126235	fr	STD
192	chr4	60751891	60751891	chr19	28845486	28845486	ff	STD
193	chr4	1.58E + 08	1.58E + 08	chr6	1.56E + 08	1.56E + 08	rf	STD
194	chr4	19639102	19639102	chr7	31696780	31696780	ff	STD
195	chr4	45015011	45015011	chr9	68364250	68364250	rf	STD
196	chr5	6531783	6531965	chr12	56387363	56387445	ff	LFR2
197	chr5	8415846	8415916	chr12	1.28E + 08	1.28E + 08	ff	LFR1
197	chr5	8416280	8416280	chr12	1.28E + 08	1.28E + 08	ff	STD
198	chr5	86788474	86788474	chr12	86766512	86766512	rr	STD
199	chr5	7299974	7300569	chr15	23105044	23105293	rr	LFR2
199	chr5	7300174	7300563	chr15	23105058	23105220	rr	LFR1
200	chr5	1.76E + 08	1.76E + 08	chr18	55381589	55381589	rf	STD
201	chr5	21572991	21573383	chr6	57575272	57575891	fr	LFR1
201	chr5	21573007	21573381	chr6	57575371	57575890	fr	LFR2
202	chr5	21572935	21572983	chr6	57574921	57575108	rr	LFR1
202	chr5	21572977	21573267	chr6	57575687	57575894	rr	LFR1
203	chr6	5841223	5841372	chr13	21911223	21911450	ff	LFR1
204	chr6	5856658	5856821	chr13	21896539	21896784	ff	LFR1
205	chr6	5853402	5853892	chr13	21899030	21899151	fr	LFR1
206	chr6	5850286	5850599	chr13	21901385	21901675	rr	LFR1
207	chr6	382040	382402	chr16	33428252	33428505	ff	LFR2
208	chr6	381822	382432	chr16	33428022	33428507	fr	LFR2
208	chr6	381989	382279	chr16	33428277	33428507	fr	LFR1
209	chr6	13191321	13191418	chr17	64637390	64637867	ff	LFR2
210	chr6	24683309	24684093	chr22	32927903	32928520	ff	LFR1
210	chr6	24683391	24684010	chr22	32927906	32928399	ff	LFR2
211	chr6	24683769	24684707	chr22	32927923	32928504	fr	LFR1
211	chr6	24683857	24684093	chr22	32927920	32928028	fr	LFR2
211	chr6	24684004	24684224	chr22	32928267	32928449	fr	LFR2
212	chr6	24684004	24684488	chr22	32927933	32928508	rf	LFR1
212	chr6	24684106	24684364	chr22	32927996	32928300	rf	LFR2
213	chr6	24683986	24684639	chr22	32927919	32928539	rr	LFR1
213	chr6	24683986	24684642	chr22	32927923	32928539	rr	LFR2
214	chr6	1.38E + 08	1.38E + 08	chr7	62982424	62982424	ff	STD
215	chr6	1.35E + 08	1.35E + 08	chr7	97337901	97337901	rf	STD
216	chr6	1.08E + 08	1.08E + 08	chr8	1.3E + 08	1.3E + 08	ff	STD
217	chr7	44911099	44911099	chr11	73527439	73527439	ff	STD
218	chr7	94842773	94842987	chr12	1.22E + 08	1.22E + 08	ff	LFR1
218	chr7	94842775	94842989	chr12	1.22E + 08	1.22E + 08	ff	LFR2
219	chr7	57605341	57605452	chr13	63648812	63648972	rr	LFR1
220	chr7	57606301	57606665	chr13	63637531	63637816	rr	LFR1
221	chr7	68915392	68915614	chr14	87380505	87380635	ff	LFR1
222	chr7	68914899	68914986	chr14	87380505	87380863	rf	LFR1
222	chr7	68914900	68915522	chr14	87380475	87380793	rf	LFR2
222	chr7	68915002	68915002	chr14	87380338	87380338	rf	STD
222	chr7	68915317	68915665	chr14	87380500	87380550	rf	LFR1
223	chr7	26252426	26252890	chr15	40853843	40854161	fr	LFR1
223	chr7	26252584	26252823	chr15	40853900	40854157	fr	LFR2
224	chr7	26241374	26241409	chr15	40854421	40854478	rf	LFR2
225	chr7	26248047	26248068	chr15	40854195	40854374	rf	LFR2
226	chr7	48721650	48721717	chr15	80736884	80737051	rr	LFR1
227	chr7	1.28E + 08	1.28E + 08	chr16	76865777	76865777	rr	STD
228	chr7	62620648	62620648	chr19	31885728	31885728	ff	STD
229	chr7	62548355	62548355	chr19	31687406	31687406	fr	STD
230	chr7	70387920	70387920	chr19	18866124	18866124	rf	STD
231	chr7	51723223	51723374	chr20	53953189	53953275	fr	LFR2
232	chr7	1857216	1857334	chr22	23801287	23801442	ff	LFR1
233	chr7	1863449	1863848	chr22	23794251	23794417	ff	LFR1
233	chr7	1866697	1866826	chr22	23791255	23791393	ff	LFR2
233	chr7	1866832	1867009	chr22	23791347	23791471	ff	LFR1
234	chr7	1879235	1879668	chr22	23778295	23778753	ff	LFR2
235	chr7	1879512	1879690	chr22	23778650	23778989	fr	LFR2
236	chr7	1856208	1856668	chr22	23801668	23801951	rr	LFR1
237	chr7	1879271	1879662	chr22	23778582	23778970	rr	LFR1
237	chr7	1879328	1879707	chr22	23778473	23778856	rr	LFR2
238	chr7	1.35E + 08	1.35E + 08	chr9	1.23E + 08	1.23E + 08	rf	STD
239	chr7	54993146	54993146	chr9	19568536	19568536	rr	STD
240	chr7	49199145	49199504	chrX	56881003	56881383	rf	LFR2
240	chr7	49199163	49199163	chrX	56881006	56881006	rf	STD
240	chr7	49199173	49199467	chrX	56881018	56881256	rf	LFR1
241	chr8	6198076	6198076	chr10	27693593	27693593	ff	STD
242	chr8	56402863	56403275	chr11	65046567	65046743	ff	LFR2
242	chr8	56402895	56403155	chr11	65046510	65046594	ff	LFR1
243	chr8	56542081	56542449	chr11	65392273	65392518	ff	LFR2
244	chr8	56618489	56618658	chr11	69301454	69301749	ff	LFR1
245	chr8	56626636	56627138	chr11	69075163	69075841	ff	LFR2
245	chr8	56626715	56627162	chr11	69075169	69075698	ff	LFR1
246	chr8	56628913	56629070	chr11	63637435	63637636	ff	LFR2
247	chr8	56638956	56639544	chr11	69201605	69202126	ff	LFR1
247	chr8	56639001	56639542	chr11	69201602	69202252	ff	LFR2
247	chr8	56639575	56639575	chr11	69201602	69201602	ff	STD
248	chr8	53545260	53545939	chr11	63501366	63501663	fr	LFR1
248	chr8	53545412	53545943	chr11	63501208	63501666	fr	LFR2
249	chr8	56542079	56542423	chr11	69308780	69309221	fr	LFR1
249	chr8	56542247	56542426	chr11	69308909	69309226	fr	LFR2
250	chr8	56592024	56592480	chr11	69239427	69239705	fr	LFR1
250	chr8	56592726	56592933	chr11	69239152	69239432	fr	LFR1
250	chr8	56593422	56593609	chr11	69239602	69239726	fr	LFR1
251	chr8	56617861	56618657	chr11	69301020	69301802	fr	LFR1
251	chr8	56618111	56618654	chr11	69301096	69301802	fr	LFR2
252	chr8	56628853	56628964	chr11	63637401	63637659	fr	LFR2
253	chr8	56651729	56652151	chr11	63572629	63572923	fr	LFR2
253	chr8	56652180	56652180	chr11	63572962	63572962	fr	STD
254	chr8	53540108	53540108	chr11	65515203	65515203	rf	STD
254	chr8	53540111	53540761	chr11	65515201	65515831	rf	LFR1
254	chr8	53540114	53540571	chr11	65515204	65515904	rf	LFR2
255	chr8	56396567	56396986	chr11	65393113	65393461	rf	LFR2
256	chr8	56419536	56419720	chr11	69011441	69011604	rf	LFR2
257	chr8	56540148	56540364	chr11	69312955	69313432	rf	LFR1
257	chr8	56540240	56540357	chr11	69313100	69313123	rf	LFR2
258	chr8	56542225	56542349	chr11	65392321	65392431	rf	LFR2
259	chr8	56574975	56575440	chr11	69322803	69322902	rf	LFR1
259	chr8	56574989	56575241	chr11	69322299	69322566	rf	LFR1
260	chr8	56591741	56591785	chr11	69263733	69263914	rf	LFR2
261	chr8	56592013	56592173	chr11	69239328	69239428	rf	LFR1
262	chr8	56628885	56628980	chr11	63637431	63637676	rf	LFR2
263	chr8	56639309	56639547	chr11	69201647	69201669	rf	LFR2
264	chr8	56687408	56688087	chr11	63575002	63575212	rf	LFR2
264	chr8	56687458	56687964	chr11	63575000	63575180	rf	LFR1
264	chr8	56687469	56687469	chr11	63575013	63575013	rf	STD
265	chr8	56714805	56715355	chr11	69312481	69312733	rf	LFR1
265	chr8	56714817	56715176	chr11	69312481	69312725	rf	LFR2
266	chr8	53564403	53564403	chr11	64929683	64929683	rr	STD
266	chr8	53564509	53564736	chr11	64929594	64929673	rr	LFR1
266	chr8	53564523	53564616	chr11	64929528	64929706	rr	LFR2
267	chr8	56366241	56366635	chr11	65393247	65393466	rr	LFR2
268	chr8	56419535	56419894	chr11	69010974	69011951	rr	LFR1
268	chr8	56419535	56420039	chr11	69011144	69011949	rr	LFR2
269	chr8	56504226	56504323	chr11	69312583	69312730	rr	LFR2
270	chr8	56574919	56575636	chr11	69322411	69322981	rr	LFR1
270	chr8	56574928	56574928	chr11	69322992	69322992	rr	STD
270	chr8	56574937	56575286	chr11	69322494	69322979	rr	LFR2
271	chr8	56591968	56592652	chr11	69239154	69239756	rr	LFR1
271	chr8	56591968	56592535	chr11	69239231	69239751	rr	LFR2
272	chr8	56619853	56620129	chr11	63582316	63582822	rr	LFR1
272	chr8	56619853	56620317	chr11	63582318	63582819	rr	LFR2
272	chr8	56619862	56619862	chr11	63582835	63582835	rr	STD
273	chr8	56628848	56629267	chr11	63637002	63637728	rr	LFR2
273	chr8	56628852	56629331	chr11	63637182	63637728	rr	LFR1
274	chr8	56642108	56642193	chr11	65392314	65392377	rr	LFR2
275	chr8	56643714	56643849	chr11	65039398	65039590	rr	LFR1
276	chr8	56687889	56688158	chr11	63575021	63575048	rr	LFR2
277	chr8	97728261	97728261	chr12	43918028	43918028	fr	STD
278	chr8	1.46E + 08	1.46E + 08	chr12	34296283	34296283	rf	STD
279	chr8	15289494	15289872	chr13	74313862	74313987	rf	LFR1
280	chr8	87976380	87976380	chr14	41325734	41325734	fr	STD
281	chr8	28478037	28478037	chr18	30473685	30473685	rr	STD
282	chr8	1E + 08	1E + 08	chr19	15711500	15711500	rr	STD
283	chr9	34466629	34467226	chr10	75030572	75030835	rr	LFR1
283	chr9	34466653	34466653	chr10	75030853	75030853	rr	STD
283	chr9	34466688	34467009	chr10	75030571	75030836	rr	LFR2
284	chr9	96384411	96384411	chr13	19645269	19645269	fr	STD
285	chr9	96382829	96382829	chr13	19646432	19646432	rf	STD
286	chr9	68418384	68418769	chr14	78101716	78101914	ff	LFR2
286	chr9	68418458	68418714	chr14	78101720	78101914	ff	LFR1
287	chr9	34467563	34467886	chr14	90356846	90357250	fr	LFR2
287	chr9	34467653	34467866	chr14	90356804	90357230	fr	LFR1
288	chr9	1.05E + 08	1.05E + 08	chr16	33957220	33957220	rf	STD
289	chr9	1.36E + 08	1.36E + 08	chr17	67794936	67794936	ff	STD
290	chr9	68792588	68792588	chr17	79312079	79312079	rf	STD
291	chr9	1.24E + 08	1.24E + 08	chr20	42578457	42578457	rr	STD

All potential translocations identified in each library are listed. Translocations were clustered by 5 kb windows around the breakpoints to allow for comparison between different libraries. An explanation of the fields are as follows: SV ID, this is the ID for each translocation after clustering; chrA, the chromosome for the A side of the translocation; chrA start, the start of the breakpoint region for the chrA side; chrA end, the end of the breakpoint region for the chrA side; chrB, the chromosome for the B side of the translocation; chrB start, the start of the breakpoint region for the chrB side; chrB end, the end of the breakpoint region for the chrB side; breakpoint orientation, the direction of the translocation on each side of the translocation as compared to NCBI reference genome build 37, “f” represents the forward direction and “r” represents the reverse direction, the first letter is the chrA region and the second letter is the chrB region; library, the library for which each individual translocation was found

**Table 3:** Translocations confirmed by published RNA sequencing data

ChrA	GeneA	ChrA start	ChrA end	GeneA strand	ChrB	GeneB	ChrB start	ChrB end	GeneB strand	Paper	Supported by SV data	Supported by raw reads
1	AHCTF1	247,002,400	247,094,726	-	4	NAAA	76,831,808	76,862,166	-	Kangaspeska et al.	yes SV_ID37	yes
17	STARD3	37,793,333	37,820,454	+	20	DOK5	53,092,266	53,267,710	+	Edgren et al.	no	yes
20	VAPB	56,964,175	57,026,157	+	17	IKZF3	37,913,968	38,020,441	-	Edgren et al.	yes SV_ID124	yes
20	TRPC4AP	33,590,207	33,680,618	-	17	MRPL45	36,452,989	36,479,101	+	Kangaspeska et al.	no	yes
20	RAB22A	56,884,771	56,942,563	+	19	MYO9B	17,186,591	17,324,104	+	Edgren et al.	yes SV_ID145	yes

Translocations identified in the standard and LFR libraries were compared to RNA sequencing identification of translocations in BT-474 from publications of Edgren et al. [7] and Kangaspeska et al. [8]. An explanation of the fields are as follows: chrA, the chromosome for the A side of the translocation; geneA, the gene affected on the A side of the translocation; chrA start, the start of the breakpoint region for the chrA side; chrA end, the end of the breakpoint region for the chrA side; geneA strand, the coding direction of the gene on the A side of the translocation; chrB, the chromosome for the B side of the translocation; geneB, the gene affected on the B side of the translocation; chrB start, the start of the breakpoint region for the chrB side; chrB end, the end of the breakpoint region for the chrB side; geneB strand, the coding direction of the gene on the B side of the translocation; paper, the publication in which the translocation was identified; supported by SV data (if there is evidence from at least one of our libraries to support this translocation a “yes” will appear in the table followed by the SV ID for our translocation, otherwise a “no” will appear); supported by raw reads (if there is evidence from at least one mate-pair read in one of our libraries to support this translocation a “yes” will appear in the table followed by the SV ID for our translocation, otherwise a “no” will appear)

**Fig. 3:**
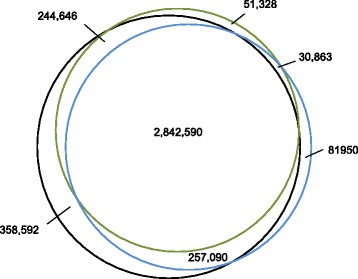
Overlap of called variations between libraries. Single nucleotide variants (SNVs) numbering 3.24 million were called in the STD library, and over 2.85 million in each of the LFR libraries. The overlap between each library was compared and plotted. The standard library (black), and LFR libraries 1 (blue) and 2 (green) are highly overlapping, demonstrating that the majority of the variant calls are highly reproducible between separately processed sequencing libraries

**Fig. 4:**
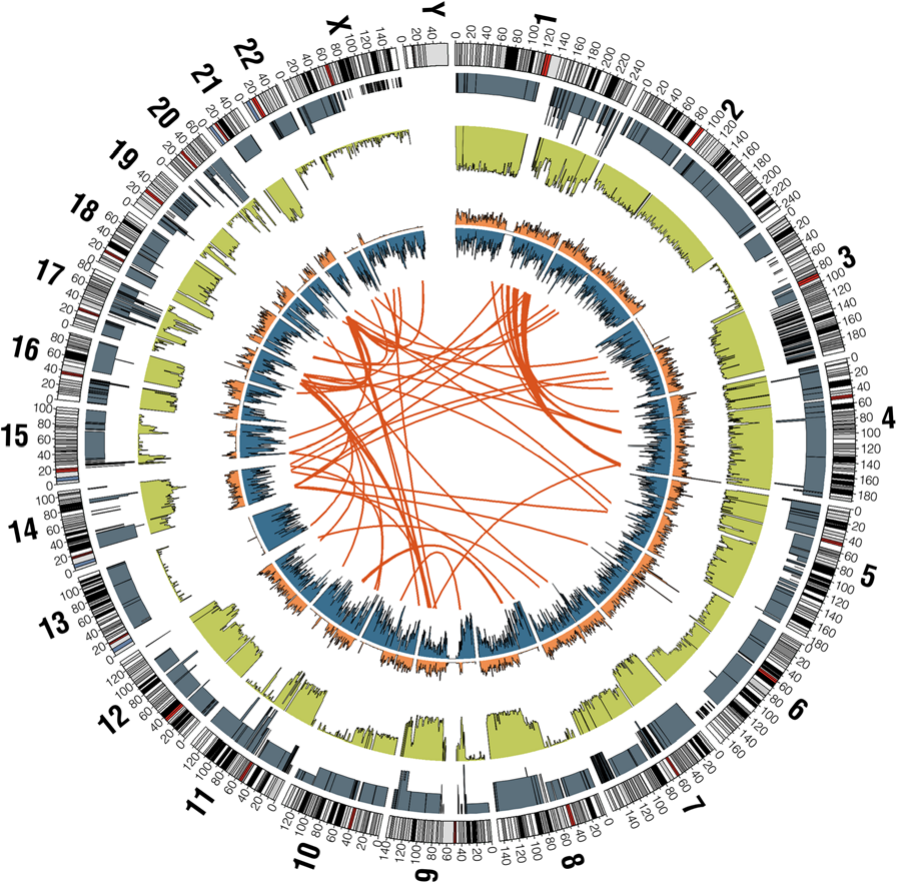
Circos plot of the BT-474 genome. Chromosome number (in large bold numbers and letters), chromosome position (in small numbers), and a karyotype ideogram form the outer circle of the plot. The remaining circles are described in order of outermost to innermost: called ploidy (the copy number of region; blue-gray), Lesser Allele Fraction (LAF, the fraction of the lesser allele, 0.5 for a heterozygous SNP, 0 for a homozygous SNP; green), density of heterzogous SNPs (orange), and density of homozygous SNPs (blue). Lines in the center of the plot represent interchromosomal junctions

Single nucleotide variants (SNVs) numbering 3.24 million were called in the STD library, and over 2.85 million in each of the LFR libraries. Of these, 2.84 million were called in all libraries (Fig. 3), demonstrating good reproducibility between different methods of library construction. For all libraries the ratio of heterozygous to homozygous was close to 1; a ratio much lower than the expected ~1.6 for Caucasian genomes. This is most likely the result of loss of heterozygosity (LOH) from the deletion or multi-copy amplifications of large portions, and/or the complete parental copy of almost all chromosomes in the BT-474 genome, as seen in our data (Fig. 1 and Fig. 4), and as previously described [2]. This was confirmed by estimating what would happen to heterozygous variants in the NA12878 genome (the sample used by the ‘Genome in a Bottle’ Consortium [9]) in two scenarios: if the same percentage of the genome was LOH based on 1) the percentage of the genome lost, or 2) the percentage of variants lost (22.2 % and 20.3 %, respectively, Table 4). In both cases the ratio of heterozygous to homozygous variants was reduced to close to 1 (Table 5).

**Table 4:** Calculation of the amount of LOH in BT-474

Chromosome	Base pairs	Variations	Centromere position (Mbp)	% LOH	LOH (bp)	LOH (variations)
1	249,250,621	4,401,091	125	0.0 %	0	0
2	243,199,373	4,607,702	93.3	0.0 %	0	0
3	198,022,430	3,894,345	91	40.4 %	80000000	1573295
4	191,154,276	3,673,892	50.4	0.0 %	0	0
5	180,915,260	3,436,667	48.4	0.0 %	0	0
6	171,115,067	3,360,890	61	0.0 %	0	0
7	159,138,663	3,045,992	59.9	0.0 %	0	0
8	146,364,022	2,890,692	45.6	34.2 %	50000000	987501
9	141,213,431	2,581,827	49	24.8 %	35000000	639910
10	135,534,747	2,609,802	40.2	41.0 %	55534747	1069355
11	135,006,516	2,607,254	53.7	40.7 %	55006516	1062289
12	133,851,895	2,482,194	35.8	0.0 %	0	0
13	115,169,878	1,814,242	17.9	100.0 %	115169878	1814242
14	107,349,540	1,712,799	17.6	0.0 %	0	0
15	102,531,392	1,577,346	19	75.0 %	76898544	1183010
16	90,354,753	1,747,136	36.6	0.0 %	0	0
17	81,195,210	1,491,841	24	0.0 %	0	0
18	78,077,248	1,448,602	17.2	0.0 %	0	0
19	59,128,983	1,171,356	26.5	0.0 %	0	0
20	63,025,520	1,206,753	27.5	0.0 %	0	0
21	48,129,895	787,784	13.2	0.0 %	0	0
22	51,304,566	745,778	14.7	100.0 %	51304566	745778
X	155,270,560	2,174,952	60.6	100.0 %	155270560	2174952
Total	3,036,303,846	55,470,937	1,028		674,184,811	11,250,331
% of total in LOH					22.2 %	20.3 %

Loss of heterozygosity (LOH) in the genome of BT-474 was calculated based on the genomic distance covered by large areas (>10 mb), where the lesser allele fraction is zero. In our data the lesser allele was calculated in 100 kb windows, based on read counts at all fully called variant loci. An explanation of the fields are as follows: chromosome, the chromosome for which each LOH region was calculated; base pairs, the total number of base pairs for each chromosome based on the NCBI reference genome build 37; variations, total number of confirmed variations for each chromosome based on Ensembl genome browser release 68; centromere position (Mbp), the location in megabases of the centromere for each chromosome; %LOH, the percentage of each chromosome estimated to have LOH; LOH (bp), the total number of base pairs for each chromosome that are estimated to be found in LOH regions; the total number of variations from Ensembl genome browser release 68 that are found within the estimated LOH region

**Table 5:** NA12878 simulation of LOH event in BT-474

	Variants in NA12878	Variants based on LOH simulation by length	Variants based on LOH simulation by number of variations
Hom SNPs	1,306,544	1,768,557	1,729,016
Het SNPs	2,081,141	1,619,128	1,658,669
Ratio	1.59	0.92	0.96

The percentage of the BT-474 genome found to be in regions of LOH was used to simulate what would happen to the heterozygous and homozygous variants in the genome of NA12878 if that same amount of LOH was to occur. The simulation demonstrated that the LOH variants, and the increase in homozygous variants as a result, caused a shift in the Het/Hom SNP ratio from 1.59 to 0.92–0.96, similar to the average ratio of the three libraries of 0.98 seen in BT-474. An explanation of the fields are as follows: hom SNPs, total number of homozygous variants; het SNPs, total number of heterozygous variants; variants in NA12878, number of variants of each category in the genome of NA12878; variants based on LOH simulation by length, the change in the number of variants based on applying the percentage of LOH as calculated from the total length of the LOH region in BT-474; variants based on LOH simulation by number of variations, the change in the number of variants based on applying the percent of LOH as calculated from the total number of variants in the LOH region in BT-474

**Fig. 5:**
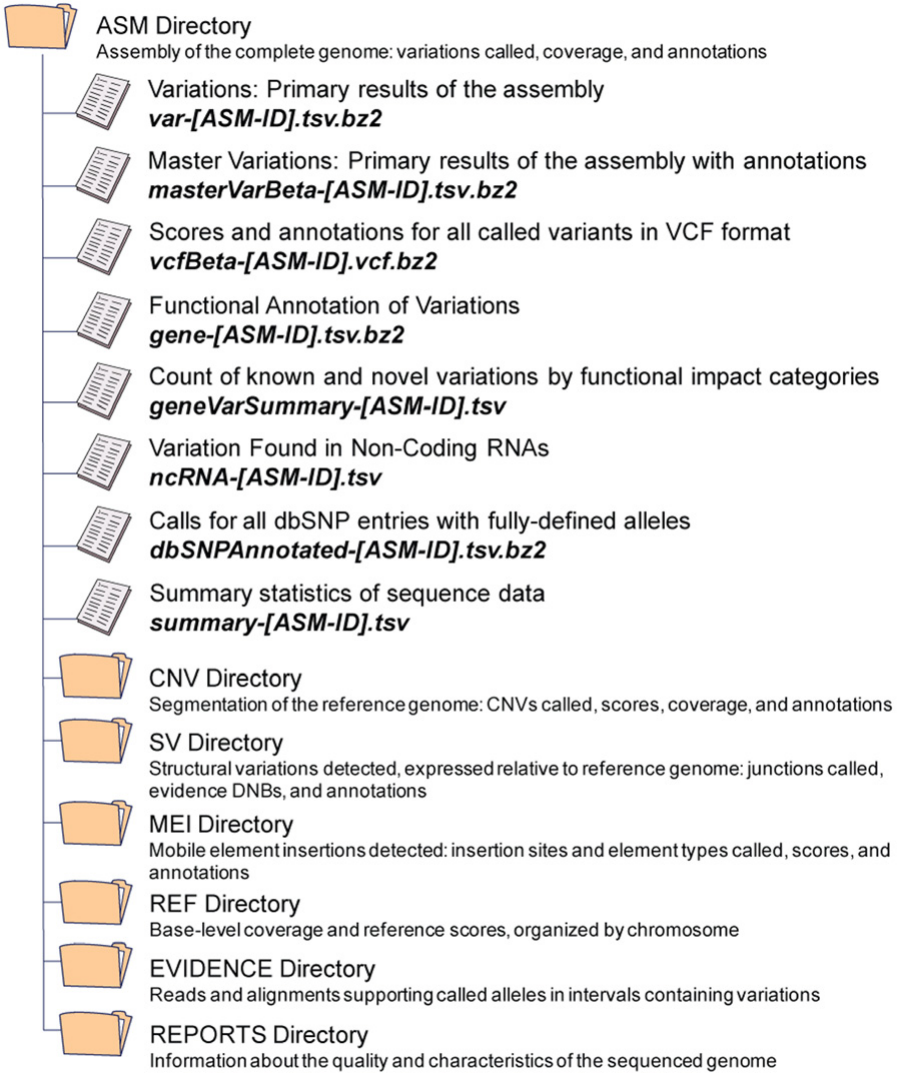
Data directory tree. The output from the LFR process consists of a series of files and folders. A complete description of everything contained within the Complete Genomics data package can be found in the Additional file 3

Analysis of the coding regions of a comprehensive list of known cancer-causing genes [10, 11] identified 67 small variants (<50 base pairs, Additional file 1). Most of these are probably inherited variants with no involvement in tumor formation, however variants in *TP53* and *PIK3CA*, previously found as somatic mutations in many tumors [12], were found in this cell line (Additional file 1). Also identified in our data: a potentially inherited variant in *CHEK2*, listed as ‘likely to be pathogenic’ in the ClinVar database [13]. To demonstrate the quality of our variant calls we compared them to a list generated by targeted sequencing of BT-474 as part of the Cancer Cell Line Encyclopedia (CCLE) project [14]. When the data from all three libraries were combined, 92 % of the variants found in CCLE were also called in our data, suggesting that our BT-474 genome is of good quality (Fig. 5 and Additional file 2). Further, 130 variants were found in two or more of our libraries that were not found in the CCLE data. This is either because the exons in which these variants were found were not covered as part of the CCLE target set, these variants were missed in the CCLE sequencing analysis, or to a lesser extent they are false positives in our dataset (Additional file 2).

## Availability of supporting data

### Complete Genomics data formats

**Fig. 6:**
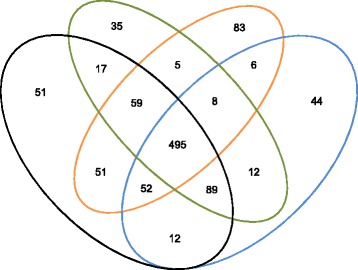
Overlap of called variations between libraries and CCLE data. Variants in the standard (black), and LFR 1 (blue) and 2 (green) libraries from this study found in the genes analyzed in the CCLE study, were compared to those variants called for BT-474 by the CCLE (orange). Eighty-nine percent of variants found by CCLE (orange) in BT-474 were also found within at least one of our libraries

The entire data set from Complete Genomics, provided here, consists of a series of files and directories covering various categories of whole genome analysis (Fig. 6). A complete description of all files and the methods used to generate them can be found in the “Standard Sequencing Service Data File Formats v2.5” document provided by Complete Genomics, Inc. (available in Additional file 3 [15]).

### LFR-specific files

Data packages from LFR do not include directories for structural variation (SV) or mobile element insertion (MEI; for more information on the content of these directories see the “Standard Sequencing Service Data File Formats v2.5” file mentioned above. In addition, one of the fields in the variant file (hapLink) is modified and there are six new fields described below:

- hapLink: LFR phased variants have an ID with the pattern: “Phased_#_#_#”, where # is an integer, the first two #s describe unique contigs, and the last # in the series is either 1 or 0 and represents the two possible haplotypes for each contig. All SNPs sharing the same “Phased_#_#_#” are from the same haplotype.
- wellCount: total number of LFR wells (out of 384) containing sequence reads calling the variant or reference allele. This metric is used to identify polymerase-induced false positive calls, since it is unlikely that random polymerase errors will occur in multiple different wells. A complete explanation of this concept can be found in Peters et al. [4].
- wellIDs: contains the IDs of the specific wells from which reads calling the variant come.
- exclusiveWellCount: this is the number of wells at each locus that have reads calling only the variant or the reference allele (not both). For true heterozygous variants this number should be close to that obtained for “wellCount”.
- SharedWellCount: at each locus this is the number of wells that contain reads calling both alleles. For true heterozygous variants this should be low; having a high number here suggests mapping errors. For homozygous variants almost all of the well counts should be in this field.
- MinExclusiveWellCountInThisLocus: this is the minimum number of exclusive wells (non-shared well counts) at each locus.
- MaxExculisveWellCountInThisLocus: this is the maximum number of exclusive wells (non-shared well counts) at each locus.

### LFR structural variant analysis files

Each LFR genome contains an LFR-specific structural variant file in the ASM directory (see Fig. 2 for directory tree). This file is generated using a novel algorithm that identifies unexpected mate-pairs that are found in more than one compartment of an LFR library (manuscript in preparation). A full description of the headers can be found within each file under the Excel tab labeled “Header Description”.

Read and mapping data for all genomes reported here are available at the European Nucleotide Archive (ENA) under study accession number PRJEB10587. Sample accession numbers for each sequence library can be found in Table 1. Supporting data is also available from the *GigaScience* GigaDB database [16].

## Additional files

Additional file 1:
**Cancer-associated genes with variants in BT-474.** (PDF 123 kb) Additional file 2:
**Comparison of calls to CCLE.** (PDF 1347 kb) Additional file 3:
**Standard Sequencing Service Data File Formats v2.** (PDF 6212 kb)

## Abbreviations

LFR: Long Fragment Read technology
STD: Complete genomics standard library
SV: Structural variations
ENA: European Nucleotide Archive
SKY: Spectral karyotype
SNV: Single nucleotide variant
CCLE: Cancer Cell Line Encyclopedia
