# Supplementary material for: Whole genome sequence analysis of BT-474 using complete Genomics’ standard and long fragment read technologies
Source: Gigascience. 2016 Feb 9;5:8. doi: 10.1186/s13742-016-0113-x (PMC4748558; doi:10.1186/s13742-016-0113-x)
Supplement: Additional file 1: — Cancer-associated genes with variants in BT-474. (PDF 123 kb) [file 13742_2016_113_MOESM1_ESM.pdf]

Table 6. Cancer associated genes with variants in BT-474

Chr	Start	End	Reference	Variant	Gene	Significance	Database reference ID	1000 genome frequency	Internal LFR library frequency	Wellderly study frequency	STD	LFR1	LFR2	Impact	Nucleotide position	Protein position	Reference amino acid	Sample amino acid	STD allele 1 var score	STD allele 2 var score	LFR1 allele 1 var score	LFR1 allele 1 well count	LFR1 allele 2 var score	LFR1 allele 2 well count	LFR1 shared well count	LFR2 allele 1 var score	LFR2 allele 1 well count	LFR2 allele 2 var score	LFR2 allele 2 well count	LFR2 shared well count	Found in CCLE
chr3	38521297	38521298	A	T	ACVR2B	Unknown					het			NONSENSE	963	314	K	*	125(ref)	125											Y
chr11	108201034	108201035	G	A	ATM	Unknown					het			MISSENSE	7786	2468	E	K	593(ref)	598											Y
chr3	142188336	142188337	A	C	ATR	Unknown	dbsnp.125:rs28910273			0.004	het			MISSENSE	6515	2132	Y	D	535(ref)	1134											N
chr2	215617177	215617178	C	G	BARD1	Unknown	dbsnp.98:rs2228453;dbsnp.125:rs28997576	0.008	0.02	0.026	het	hom		MISSENSE	1804	557	C	S	446(ref)	587	1525	15	165	15	15						N
chr6	136599651	136599652	G	C	BCLAF1	Unknown					het			MISSENSE	619	123	Q	E	227(ref)	227											N
chrX	39933228	39933229	T	G	BCOR	Unknown					hom	hom	hom	MISSENSE	1661	457	H	P	136	1132	909	12	89	12	12	82	13	727	13	13	N
chr17	41245470	41245471	C	T	BRCA1	Benign	dbsnp.113:rs4986850	0.043	0.0406504	0.081	hom	hom	hom	MISSENSE	2308	693	D	N	152	1508	25	10	434	10	10	19	7	181	7	7	N
chr13	32914235	32914236	C	T	BRCA2	Other	dbsnp.113:rs4987117	0.007	0.02	0.026	hom	hom	hom	MISSENSE	5970	1915	T	M	752	94	186	8	28	8	8	343	7	27	7	7	N
chr13	32968849	32968850	C	A	BRCA2	Unknown					het			NONSENSE	9507	3094	S	*	372(ref)	450											Y
chr19	15355281	15355282	G	A	BRD4	Unknown					het			MISSENSE	2562	781	P	S	329(ref)	329											N
chr17	59886056	59886057	G	A	BRIP1	Unknown					het		het	MISSENSE	994	230	S	L	376(ref)	376						593(ref)	18(ref)	593	11	1	Y
chr22	29083948	29083951	CGG	TGA	CHEK2	Unknown				0.018		het		MISSENSE	1766	565	PR	PH			92(ref)	9(ref)	75	17	0						N
chr22	29091787	29091788	T	C	CHEK2	Likely Pathogenic	dbsnp.137:rs200928781	0.003	0.0165289	0.004			het	MISSENSE	1240	390	Y	C								78(ref)	14(ref)	72	18	0	N
chr19	42798826	42798827	C	G	CIC	Unknown					het			MISSENSE	4438	1467	L	V	414(ref)	414											Y
chr2	211523337	211523338	C	T	CPS1	Unknown					het		hom	MISSENSE	3816	1228	R	W	288(ref)	1184						110	20	999	20	20	N
chr7	101917523	101917524	G	T	CUX1	Unknown					het			NONSENSE	1513	463	E	*	261(ref)	261											N
chr3	57391501	57391502	C	G	DNAH12	Unknown					het			MISSENSE	6577	2133	D	H	340(ref)	340											N
chr3	57456276	57456277	G	C	DNAH12	Unknown					het			MISSENSE	2178	666	F	L	283(ref)	460											N
chr18	33750154	33750155	C	G	ELP2	Unknown					het		het	MISSENSE	2187	710	Q	E	384(ref)	384						150(ref)	7(ref)	150	6	0	N
chr1	16456762	16456763	C	T	EPHA2	Unknown	dbsnp.126:rs35903225	0.002	0.0285714	0.022	het		het	MISSENSE	2781	876	R	H	509(ref)	639						160(ref)	6(ref)	243	6	0	N
chr6	152129062	152129063	C	T	ESR1	Unknown	COSMIC:mut:1559733;dbsnp.134:rs139960913		0.008	0.006	het		het	MISSENSE	233	6	H	Y	1016(ref)	300						279(ref)	6(ref)	385	7	0	N
chr7	148525903	148525904	C	G	EZH2	Benign	dbsnp.100:rs2302427	0.073	0.0887097	0.075	het	het		MISSENSE	628	146	D	H	325(ref)	308	376(ref)	12(ref)	190	7	0						N
chr16	89815074	89815075	A		FANCA	Unknown					het			FRAMESHIFT	3381	1114	S	L	330(ref)	330											N
chr16	89883006	89883007	A	T	FANCA	Unknown	dbsnp.89:rs1800282	0.04	0.0869565	0.089	het			MISSENSE	58	6	V	D	261(ref)	261											N
chr3	10107586	10107587	A	G	FANCD2	Unknown		0.001	0.0219298			het		MISSENSE	2386	770	K	R			79(ref)	7(ref)	79	18	1						N
chr3	10114614	10114615	C	T	FANCD2	Unknown			0.00420168			het		MISSENSE	2632	852	P	L			71(ref)	7(ref)	58	8	0						N
chr1	152285053	152285054	G	C	FLG	Unknown					het			MISSENSE	2343	770	H	D	319(ref)	319											N
chr5	180048661	180048662	C	T	FLT4	Unknown					het			MISSENSE	1978	634	A	T	305(ref)	305											N
chr6	26031884	26031885	C	G	HIST1H3B	Unknown	COSMIC:mut:333040				het	het	het	MISSENSE	403	135	R	T	905(ref)	497	590(ref)	7(ref)	355	8	0	562(ref)	8(ref)	307	12	0	N
chr12	121435449	121435450	C	T	HNF1A	Unknown					het	het		NONSENSE	1505	495	Q	*	453(ref)	453	280(ref)	9(ref)	280	8	0						Y
chr12	53586254	53586255	G	A	ITGB7	Unknown	dbsnp.120:rs11539433	0.005	0.004	0.011	het	het		MISSENSE	2164	672	H	Y	695(ref)	1807	981(ref)	7(ref)	981	8	2						N
chr2	141777553	141777554	C	T	LRP1B	Unknown	dbsnp.131:rs77234491	0.002	0.00403226	0.003	het			MISSENSE	2878	636	R	Q	208(ref)	208											N
chr2	39515383	39515384	G	A	MAP4K3	Unknown					het			MISSENSE	1676	451	T	I	281(ref)	281											Y
chr22	22162071	22162072	G	C	MAPK1	Unknown					het		het	MISSENSE	422	61	H	Q	284(ref)	284						327(ref)	7(ref)	335	7	0	Y
chr15	42041742	42041743	G	A	MGA	Unknown					het	het		MISSENSE	5491	1771	E	K	1028	705(ref)	449	9	887(ref)	13(ref)	0						Y
chr11	118365017	118365018	G	A	MLL	Unknown					het			MISSENSE	5216	1732	D	N	685(ref)	685											Y
chr12	49438622	49438623	C	T	MLL2	Unknown					het			MISSENSE	4866	1623	E	K	233(ref)	233											N
chr19	36220996	36220997	C	T	MLL4	Unknown	COSMIC:mut:1392940				het	het	het	NONSENSE	5046	1683	O	*	601(ref)	601	79(ref)	13(ref)	79	7	0	122(ref)	9(ref)	122	6	0	N
chr17	27434114	27434115	C	A	MYO18A	Unknown					het	het		MISSENSE	3581	1142	V	L	1131(ref)	2249	884(ref)	9(ref)	884	9	0						N
chr12	124821522	124821523	G	C	NCOR2	Unknown					het			MISSENSE	6181	1954	S	C	179(ref)	179											N
chr5	176684146	176684147	C	G	NSD1	Unknown					het	het		MISSENSE	5098	1654	S	C	529(ref)	529	84(ref)	21(ref)	84	7	1						Y
chr5	176715854	176715855	C	G	NSD1	Unknown					het			MISSENSE	5539	1794	L	V	365(ref)	365											Y
chr2	242082261	242082263	TG	CA	PASK	Unknown	dbsnp.116:rs6709462;dbsnp.134:rs144572631				het		hom	MISSENSE	317	62	T	M		988						409	11	51	11	11	N
chr3	178916945	178916946	G	C	PIK3CA	Pathogenic	COSMIC:mut:12580;COSMIC:mut:582516				het			MISSENSE	489	111	K	N	497(ref)	497											Y
chr12	133257836	133257837	C	A	POLE	Unknown	dbsnp.126:rs34047482	0.014	0.00819672	0.021	het			MISSENSE	134	31	A	S	237(ref)	1391											N
chr19	52714669	52714670	C	T	PPP2R1A	Unknown	dbsnp.132:rs112759633				het			MISSENSE	722	143	S	F	467(ref)	467											N
chr8	117864885	117864886	C	A	RAD21	Unknown					het	het		MISSENSE	1510	408	G	V	226(ref)	226	502(ref)	17(ref)	502	6	0						Y
chr3	49412972	49412973	C	T	RHOA	Unknown	dbsnp.120:rs11552761				het			MISSENSE	325	17	G	E	279(ref)	523											Y
chr1	155874289	155874290	C	G	RIT1	Unknown					het		het	MISSENSE	443	81	E	O	340(ref)	340						250(ref)	6(ref)	250	6	1	N
chr6	117642452	117642453	C	A	ROS1	Unknown					het		het	MISSENSE	5944	1916	V	L	487(ref)	487						385	7	385(ref)	10(ref)	0	Y
chr13	23928670	23928671	C	T	SACS	Unknown	dbsnp.123:rs17325713	0.009	0.0322581	0.026	hom	hom		MISSENSE	2668	694	A	T	1435	138	42	9	121	9	9						N
chr5	36680530	36680531	G	C	SLC1A3	Unknown					het	het		MISSENSE	1604	377	E	O	559(ref)	559	242(ref)	17(ref)	242	8	0						N
chr2	74477511	74477512	G	C	SLC4A5	Unknown					het			MISSENSE	2008	537	I	M	262(ref)	262											N
chr8	55371788	55371789	C	T	SOX17	Unknown	dbsnp.137:rs200011294	0.001	0.00436681	0.005	het	hom	het	MISSENSE	682	160	A	V	228(ref)	714	14	8	703	8	8	416(ref)	11(ref)	428	7	0	N
chr17	40490776	40490777	G	C	STAT3	Unknown					het	het		MISSENSE	739	174	F	L	732(ref)	732	213(ref)	10(ref)	213	8	1						Y
chr4	106156186	106156187	C	T	TET2	Unknown	dbsnp.123:rs17253672	0.02	0.048	0.056	het	het		MISSENSE	1574	363	P	L	486(ref)	836	895(ref)	10(ref)	895	9	0						N
chr3	30686251	30686252	G	C	TGFBR2	Unknown					het		hom	MISSENSE	564	61	M	I	246(ref)	1287						35	13	436	13	13	N
chr10	98188497	98188498	C	G	TLL2	Unknown					het		het	MISSENSE	768	176	Q	H	502(ref)	502						260(ref)	7(ref)	260	6	0	N
chr17	7577084	7577085	C	T	TP53	Pathogenic	COSMIC:mut:10722;COSMIC:mut:137087;dbsnp.132:rs112431538				hom	hom	hom	MISSENSE	734	153	E	K	2041	222	138	19	1483	19	19	96	15	811	15	15	Y
chr19	10469974	10469975	A	C	TYK2	Unknown	COSMIC:mut:438469;dbsnp.121:rs12720356	0.035	0.0939597	0.098	het			MISSENSE	2428	684	I	S	494(ref)	494											N
chr19	10477205	10477206	G	A	TYK2	Benign	dbsnp.79:rs280523	0.077	0.0446429	0.081	het	hom		INSERT+	893	172	T	TE	651(ref)	651	445	13	41	13	13						N
chr16	701955	701956	G	A	WDR90	Unknown					het			MISSENSE	1023	324	D	N	135(ref)	135											N
chr16	716511	716512	C	T	WDR90	Unknown	dbsnp.126:rs35506206			0.011	het			MISSENSE	4851	1600	R	W	217(ref)	766											N
chr1	155640114	155640115	C	T	YY1AP1	Unknown	dbsnp.127:rs41264945	0.017	0.076	0.054	het	het	het	MISSENSE	943	176	D	N	335(ref)	335	489(ref)	10(ref)	489	6	0	280(ref)	10(ref)	531	6	0	N
chr1	155657900	155657901	A	G	YY1AP1	Unknown	dbsnp.134:rs143953255		0.0168067	0.008			het	MISSENSE	546	119	S	P								42(ref)	9(ref)	40	10	1	N
chr20	52192511	52192512	G	C	ZNF217	Unknown	dbsnp.127:rs41274706	0.001	0.00806452	0.004	het		hom	MISSENSE	3061	931	Q	E	94(ref)	3837						167	91	2898	91	91	N
chr8	37554797	37554798	G	T	ZNF703	Unknown					het			MISSENSE	575	127	G	C	199(ref)	288											N
