# Supplementary material for: Whole genome sequence analysis of BT-474 using complete Genomics’ standard and long fragment read technologies
Source: Gigascience. 2016 Feb 9;5:8. doi: 10.1186/s13742-016-0113-x (PMC4748558; doi:10.1186/s13742-016-0113-x)
Supplement: Additional file 2: — Comparison of calls to CCLE. (PDF 1347 kb) [file 13742_2016_113_MOESM2_ESM.pdf]

Table 7. Comparison of calls to CCLE

Gene	Chr	Start	End	Reference	Variant	Database reference ID	Impact	Nucleotide position	Protein position	Reference amino acid	Sample amino acid	STD	LFR1	LFR2	Found in CCLE
AAK1	chr2	69741753	69741756	TGT		rs55712143	IN_FRAME_DEL		541542		Q	not called	not called	not called	Y
AATK	chr17	79093821	79093822	A	G	dbsnp.126:rs36000545	MISSENSE	3876	1266	F	S	hom	not called	not called	Y
AATK	chr17	79095628	79095629	C	A	COSMIC:mut:437761;COSMIC:mut:1480153;dbsnp.116:rs7503604	MISSENSE	2186	703	G	C	hom	not called	not called	N
ABCA3	chr16	2369591	2369592	C	T	dbsnp.132:rs117603931	MISSENSE	1574	288	R	K	het	het	het	N
ABCC4	chr13	95859034	95859035	C	A	dbsnp.100:rs2274407	MISSENSE	1030	304	K	N	hom	hom	hom	Y
ACACA	chr17	35605016	35605017	C	G		MISSENSE	2739	743	V	L	not called	not called	het	N
ACACB	chr12	109634833	109634834	A	G	dbsnp.123:rs17848802	MISSENSE	2511	835	M	V	het	het	het	Y
ACACB	chr12	109696837	109696838	G	A	dbsnp.96:rs2075260	MISSENSE	6429	2141	V	I	hom	hom	hom	Y
ACVR2A	chr2	148602770	148602771	C	T		MISSENSE	201	17	S	F	het	not called	not called	N
ACVR2B	chr3	38521297	38521298	A	T		NONSENSE	963	314	K	*	het	not called	het	Y
ADAM12	chr10	128019024	128019025	C	G	dbsnp.107:rs3740199	MISSENSE	554	48	G	R	hom	hom	half called	Y
ADAM22	chr7	87564496	87564497	C	G	dbsnp.100:rs2279542	MISSENSE	320	81	P	R	het	het	het	Y
ADAM28	chr8	24211330	24211331	G	A	dbsnp.116:rs7814768	MISSENSE	2375	765	V	M	hom	hom	hom	Y
ADAMTS18	chr16	77325324	77325325	G	T	COSMIC:mut:148121;dbsnp.126:rs35478105	MISSENSE	3658	1080	S	R	het	het	het	Y
ADAMTS18	chr16	77328989	77328990	C	A	dbsnp.121:rs12935394	MISSENSE	3254	946	A	S	het	het	het	Y
ADAMTS18	chr16	77353972	77353973	G	T	dbsnp.119:rs9930984	MISSENSE	2723	769	L	I	het	het	het	Y
ADAMTS18	chr16	77359918	77359919	A	T	dbsnp.120:rs11640912	MISSENSE	2294	626	L	I	het	het	het	Y
ADAMTS19	chr5	128863470	128863471	A	G	dbsnp.116:rs6595908	MISSENSE	1098	367	S	G	hom	hom	hom	Y
ADAMTS19	chr5	129040055	129040056	A	T	dbsnp.120:rs11749126	MISSENSE	3265	1089	Y	F	het	hom	het	Y
ADAMTS2	chr5	178540974	178540975	G	A	dbsnp.86:rs1054480	MISSENSE	3630	1177	P	S	het	het	het	Y
ADARB2	chr10	1230967	1230968	C	T	dbsnp.100:rs2271275	MISSENSE	2249	626	A	T	hom	hom	hom	Y
ADCK4	chr19	41211055	41211056	T	C	COSMIC:mut:148631;COSMIC:mut:148632;dbsnp.108:rs3865452	MISSENSE	421	133	H	R	het	het	hom	Y
ADHFE1	chr8	67380527	67380528	T	C	dbsnp.86:rs1060242	MISSENSE	1378	449	C	R	hom	hom	hom	Y
AFF3	chr2	100175361	100175362	G	C		NONSENSE	3478	1112	S	*	het	het	het	Y
AIM1	chr6	106967184	106967185	A	C	dbsnp.87:rs1159148	MISSENSE	1364	293	Q	P	het	het	not called	Y
AIM1	chr6	106999821	106999822	G	A	COSMIC:mut:150149;dbsnp.100:rs2297970	MISSENSE	4670	1395	C	Y	not called	not called	het	Y
AKAP12	chr6	151669874	151669875	A	G	dbsnp.120:rs10872670	MISSENSE	588	117	K	E	hom	hom	hom	Y
AKAP12	chr6	151670171	151670172	A	C	dbsnp.107:rs3734799	MISSENSE	885	216	K	Q	hom	hom	half called	Y
AKAP12	chr6	151671816	151671818	GG	TC		MISSENSE	2530	764	R	I	not called	not called	het	N
AKAP12	chr6	151674116	151674116		GAG	dbsnp.107:rs3842128;dbsnp.132:rs113116275;dbsnp.126:rs34338625	INSERT	4830	1532		E	hom	hom	hom	Y
AKAP12	chr6	151674325	151674326	A	C	dbsnp.107:rs3823310	MISSENSE	5039	1600	E	D	hom	hom	hom	Y
AKAP6	chr14	33291639	33291641	TA	AG		MISSENSE	4790	1541	Y	S	not called	not called	het	N
AKAP9	chr7	91630619	91630620	G	T	dbsnp.116:rs6964587	MISSENSE	1613	463	M	I	het	het	het	Y
AKAP9	chr7	91631992	91631993	A	G		MISSENSE	2986	921	K	R	not called	het	not called	N
AKAP9	chr7	91652178	91652178		AAC	COSMIC:mut:112144;COSMIC:mut:112145;dbsnp.119:rs10644111;dbsnp.132:rs111673064;dbsnp.126:rs34733695	INSERT	4228	1336		Q	het	het	half called	Y
AKAP9	chr7	91712697	91712698	A	G	dbsnp.116:rs6960867	MISSENSE	8599	2792	N	S	het	het	not called	Y
AKAP9	chr7	91714893	91714934	TTCAGCAGGTTTCAGAACCTTGGCTAGAAGAGAGAAAAGCT	TTCAGCAGGTTTCAGAATCTTGGCTAGAAGAGAGAAAAGCT	dbsnp.86:rs1063242	MISSENSE	9142	2979	P	S	not called	half called	not called	N
AKAP9	chr7	91714910	91714911	C	T	dbsnp.86:rs1063242	MISSENSE	9159	2979	P	S	hom	not called	hom	Y
AKAP9	chr7	91715608	91715609	A	G	dbsnp.129:rs61757673	MISSENSE	9316	3031	Q	R	het	het	het	Y
ALK	chr2	29416365	29416366	G	C	dbsnp.92:rs1881421	MISSENSE	5493	1529	D	E	het	hom	het	Y
ALK	chr2	29416480	29416481	T	C	dbsnp.92:rs1881420	MISSENSE	5378	1491	K	R	het	hom	half called	Y
ALK	chr2	29416571	29416572	T	C	dbsnp.89:rs1670283	MISSENSE	5287	1461	I	V	hom	hom	hom	Y
ALPK1	chr4	113352396	113352397	G	A	dbsnp.96:rs2074388	MISSENSE	1920	565	G	D	hom	hom	hom	Y
ALPK1	chr4	113352627	113352628	A	G	dbsnp.121:rs13148353	MISSENSE	2151	642	H	R	hom	hom	hom	Y
ALPK1	chr4	113352898	113352899	G	A	dbsnp.96:rs2074379	MISSENSE	2422	732	M	I	hom	hom	hom	Y
ALPK1	chr4	113353284	113353285	T	C	dbsnp.120:rs11726117	MISSENSE	2808	861	M	T	hom	hom	hom	Y
ALPK2	chr18	56149098	56149099	T	C	dbsnp.116:rs7240666	MISSENSE	6682	2157	I	V	hom	hom	hom	Y
ALPK2	chr18	56202767	56202768	C	A	dbsnp.107:rs3809983	MISSENSE	4864	1551	A	S	hom	hom	hom	Y
ALPK2	chr18	56203073	56203074	G	A	dbsnp.107:rs3809982	MISSENSE	4558	1449	P	S	hom	hom	hom	Y
ALPK2	chr18	56203897	56203898	T	G	dbsnp.107:rs3809977	MISSENSE	3734	1174	H	P	hom	hom	hom	Y
ALPK2	chr18	56204249	56204250	A	C	dbsnp.107:rs3809976	MISSENSE	3382	1057	L	V	hom	hom	hom	Y
ALPK2	chr18	56204387	56204402	CAGTTGATGTGTCCT		dbsnp.130:rs67925233;dbsnp.130:rs67804623	DELETE	3230	1006	EDTST		hom	hom	hom	Y
ALPK2	chr18	56204670	56204671	A	T	dbsnp.111:rs4940404	MISSENSE	2961	916	N	K	hom	hom	hom	Y
ALPK2	chr18	56204746	56204747	G	A	dbsnp.107:rs3826593	MISSENSE	2885	891	T	I	hom	hom	hom	Y
ALPK2	chr18	56204931	56204932	T	G	dbsnp.107:rs3809973	MISSENSE	2700	829	K	N	hom	hom	hom	Y
ALPK2	chr18	56204944	56204945	C	G	dbsnp.107:rs3809972	MISSENSE	2687	825	R	T	hom	hom	hom	Y
ALPK2	chr18	56204990	56204991	C	T	dbsnp.107:rs3809970	MISSENSE	2641	810	G	S	hom	hom	hom	Y
ALPK2	chr18	56205261	56205262	A	C	dbsnp.120:rs12103986	MISSENSE	2370	719	H	Q	hom	hom	hom	Y
ALPK2	chr18	56279024	56279025	T	G	dbsnp.116:rs6566987	MISSENSE	218	2	K	T	het	het	het	Y

ALPK3	chr15	85401258	85401259	C	T	dbsnp.79:rs306197	MISSENSE	4062	1299	P	L	hom	hom	hom	N
ALS2	chr2	202625614	202625615	C	T	dbsnp.106:rs3219156	MISSENSE	1457	368	V	M	hom	hom	hom	Y
AMZ2	chr17	66246415	66246416	A	G	dbsnp.106:rs3213690	MISSENSE	236	30	N	D	hom	hom	hom	Y
AMZ2	chr17	66252988	66252989	A	G	dbsnp.86:rs1139707	MISSENSE	1110	321	D	G	not called	het	not called	N
ANKK1	chr11	113266820	113266821	G	A	dbsnp.116:rs7118900	MISSENSE	808	239	A	T	hom	not called	hom	Y
ANKK1	chr11	113270014	113270015	G	C	dbsnp.111:rs4938016	MISSENSE	1417	442	G	R	hom	half called	hom	Y
ANKK1	chr11	113270827	113270828	G	A	dbsnp.89:rs1800497	MISSENSE	2230	713	E	K	hom	not called	not called	Y
APC	chr5	112176755	112176756	T	A	dbsnp.80:rs459552	MISSENSE	5549	1822	V	D	het	not called	not called	Y
ARHGAP29	chr1	94639446	94639447	C	T	dbsnp.92:rs1999272	MISSENSE	4132	1255	G	D	hom	hom	hom	Y
ARHGAP29	chr1	94669544	94669545	C	G		MISSENSE	1071	235	E	Q	het	not called	het	Y
ARHGEF10	chr8	1857590	1857591	G	A	dbsnp.100:rs2294039	MISSENSE	2275	700	V	I	hom	hom	hom	Y
ARHGEF12	chr11	120337959	120337960	C	G		MISSENSE	2803	933	Q	E	het	het	het	Y
ASPSCR1	chr17	79954543	79954544	T	A	dbsnp.116:rs8074498	MISSENSE	851	252	L	Q	hom	hom	half called	Y
ASTN1	chr1	176863835	176863836	G	C	dbsnp.100:rs2281180	MISSENSE	3013	934	H	Q	het	het	het	Y
ASTN1	chr1	176863866	176863867	T	C	dbsnp.98:rs2228956	MISSENSE	2982	924	H	R	het	het	het	Y
ATM	chr11	108139183	108139185	CT	GA		MISSENSE	3070	896	L	D	not called	not called	het	N
ATM	chr11	108201034	108201035	G	A		MISSENSE	7786	2468	E	K	het	het	het	Y
ATP10A	chr15	25925093	25925094	C	G	dbsnp.107:rs3816800	MISSENSE	3999	1298	R	S	hom	hom	hom	Y
ATP11A	chr13	113479819	113479820	A	G	COSMIC:mut:147717;dbsnp.80:rs368865	MISSENSE	1036	317	M	V	hom	hom	hom	Y
ATP13A5	chr3	193031925	193031926	C	T	dbsnp.100:rs2280268	MISSENSE	2214	739	G	S	hom	hom	hom	Y
ATP13A5	chr3	193080413	193080414	C	G	dbsnp.116:rs6797429	MISSENSE	396	133	E	Q	het	het	het	Y
ATP13A5	chr3	193081121	193081122	G	T	COSMIC:mut:77312;dbsnp.120:rs12637558	MISSENSE	286	96	S	Y	het	het	het	Y
ATP8B1	chr18	55322501	55322502	C	T	dbsnp.121:rs12968116	MISSENSE	2974	952	R	Q	het	het	het	Y
ATR	chr3	142178143	142178144	C	T	COSMIC:mut:149485;dbsnp.98:rs2229032	MISSENSE	7395	2425	R	Q	het	not called	het	Y
ATR	chr3	142188336	142188337	A	C	dbsnp.125:rs28910273	MISSENSE	6515	2132	Y	D	het	het	het	Y
ATR	chr3	142281611	142281612	A	G	COSMIC:mut:149487;dbsnp.98:rs2227928	MISSENSE	753	211	M	T	het	het	not called	Y
ATRX	chrX	76937962	76937963	G	C	dbsnp.102:rs3088074	MISSENSE	3016	929	Q	E	hom	hom	hom	Y
ATRX	chrX	76949378	76949379	C	T		MISSENSE	649	140	D	N	not called	het	not called	N
AURKA	chr20	54961462	54961463	T	C	dbsnp.86:rs1047972	MISSENSE	526	57	I	V	hom	hom	hom	Y
AURKA	chr20	54961540	54961541	A	T	dbsnp.100:rs2273535	MISSENSE	448	31	F	I	het	hom	het	Y
AURKB	chr17	8108330	8108331	A	G	dbsnp.86:rs1059476	MISSENSE	953	298	M	T	hom	hom	hom	Y
BAX	chr19	49458970	49458970		G		FRAMESHIFT	182	41	E	G	not called	het	not called	N
BCAR1	chr16	75276774	75276775	G	A	dbsnp.86:rs1035539	MISSENSE	647	122	P	S	het	not called	het	N
BCL2A1	chr15	80263216	80263217	C	T	dbsnp.107:rs3826007	MISSENSE	426	82	G	D	het	not called	het	Y
BCL2A1	chr15	80263344	80263345	A	C	dbsnp.86:rs1138358	MISSENSE	298	39	N	K	het	het	het	Y
BCL2A1	chr15	80263405	80263406	C	T	dbsnp.86:rs1138357	MISSENSE	237	19	C	Y	het	het	not called	Y
BCL9	chr1	147091971	147091972	C	T	dbsnp.107:rs3820129	MISSENSE	2750	671	P	S	het	het	het	Y
BCR	chr22	23627368	23627369	A	G	dbsnp.78:rs140504	MISSENSE	2982	796	N	S	hom	half called	hom	Y
BIRC5	chr17	76219590	76219591	G	A	dbsnp.96:rs2071214	MISSENSE	574	152	E	K	hom	hom	hom	Y
BIRC6	chr2	32713704	32713706	AA	AT	COSMIC:mut:148833;dbsnp.100:rs2366894	MISSENSE	8152	2674	T	S	not called	half called	not called	N
BIRC6	chr2	32713705	32713706	A	T	COSMIC:mut:148833;dbsnp.100:rs2366894	MISSENSE	8153	2674	T	S	hom	not called	hom	Y
BLM	chr15	91292702	91292704	GA	TC		MISSENSE	301	69	E	S	not called	not called	het	N
BMP4	chr14	54417521	54417522	A	G	dbsnp.63:rs17563	MISSENSE	873	152	V	A	hom	hom	hom	Y
BRCA1	chr17	41223093	41223094	T	C	dbsnp.89:rs1799966	MISSENSE	5068	1613	S	G	hom	hom	hom	Y
BRCA1	chr17	41243999	41244000	T	C	COSMIC:mut:148277;dbsnp.60:rs16942	MISSENSE	3779	1183	K	R	hom	hom	hom	N
BRCA1	chr17	41244434	41244435	T	C	dbsnp.60:rs16941	MISSENSE	3344	1038	E	G	hom	hom	hom	Y
BRCA1	chr17	41244935	41244936	G	A	COSMIC:mut:148278;dbsnp.86:rs799917	MISSENSE	2843	871	P	L	hom	hom	hom	N
BRCA1	chr17	41245470	41245471	C	T	dbsnp.113:rs4986850	MISSENSE	2308	693	D	N	hom	hom	hom	Y
BRCA2	chr13	32914235	32914236	C	T	dbsnp.113:rs4987117	MISSENSE	5970	1915	T	M	hom	hom	hom	Y
BRCA2	chr13	32968849	32968850	C	A		NONSENSE	9507	3094	S	*	het	het	not called	Y
BRD2	chr6	32946963	32946964	G	A	dbsnp.79:rs206778	MISSENSE	3242	647	D	N	het	not called	not called	N
BRD4	chr19	15355281	15355282	G	A		MISSENSE	2562	781	P	S	het	not called	not called	N
BRD4	chr19	15376247	15376247		GGGGGGGCACTGGCGGGGGCGTCTGCAGTGGCTG		FRAMESHIFT	988	256	QPQPPPAPAPQP	PATADAPASAPP	not called	het	not called	N
BRDT	chr1	92428494	92428495	C	A	dbsnp.120:rs10783071	MISSENSE	327	62	Q	K	hom	hom	hom	Y
BRDT	chr1	92445256	92445257	C	G	dbsnp.102:rs3088232	MISSENSE	1373	410	N	K	hom	hom	hom	Y
BRDT	chr1	92445266	92445267	G	A		MISSENSE	1383	414	D	N	het	het	het	Y
BRDT	chr1	92457842	92457843	C	T	rs10747493	MISSENSE		700	P	L	not called	not called	not called	Y

BRDT	chr1	92479759	92479761	CA	TT		DISRUPT	2919	926		CTGCTTTTTCTATTG	not called	not called	het	N
BRIP1	chr17	59763346	59763347	A	G	dbsnp.113:rs4986764	MISSENSE	3060	919	S	P	het	het	het	N
BRIP1	chr17	59886056	59886057	G	A		MISSENSE	994	230	S	L	het	not called	het	Y
BRSK1	chr19	55815035	55815035		C		FRAMESHIFT	1404	379	R	P	not called	not called	het	N
BUB1B	chr15	40493175	40493176	C	T		MISSENSE	1773	521	S	F	het	het	het	Y
C15orf2	chr15	24922229	24922230	C	G	rs3742950	MISSENSE		406	Q	E	not called	not called	not called	Y
C15orf55	chr15	34640217	34640218	C	T	rs374230	MISSENSE		50	P	L	not called	not called	not called	Y
C6orf97	chr6	151894504	151894505	T	C	rs953767	MISSENSE		324	F	S	not called	not called	not called	Y
C6orf97	chr6	151936676	151936677	G	A	rs6929137	MISSENSE		604	V	I	not called	not called	not called	Y
C6orf97	chr6	151939180	151939181	G	A	rs3734804	MISSENSE		683	V	I	not called	not called	not called	Y
CAMK1G	chr1	209785205	209785206	G	A	dbsnp.120:rs11119315	MISSENSE	1086	329	V	I	het	het	het	Y
CAMKK1	chr17	3775847	3775848	T	C	dbsnp.116:rs7214723	MISSENSE	1271	375	E	G	hom	hom	hom	Y
CAMKK2	chr12	121691095	121691096	G	A	dbsnp.86:rs1132780	MISSENSE	1915	363	R	C	het	het	het	Y
CAMKK2	chr12	121712076	121712077	T	A	dbsnp.107:rs3817190	MISSENSE	1081	85	T	S	het	het	het	Y
CARD14	chr17	78171943	78171944	G	C	dbsnp.96:rs2066964	MISSENSE	1835	547	R	S	hom	hom	not called	N
CARD14	chr17	78178892	78178893	C	T	dbsnp.120:rs11652075	MISSENSE	2625	820	R	W	hom	not called	not called	N
CARD9	chr9	139266495	139266496	C	T	COSMIC:mut:455594;dbsnp.108:rs4077515	MISSENSE	200	12	S	N	het	not called	not called	Y
CASC5	chr15	40898642	40898643	G	C	COSMIC:mut:147880;dbsnp.116:rs7177192	MISSENSE	288	43	R	T	hom	hom	hom	Y
CASC5	chr15	40903050	40903051	G	A	dbsnp.116:rs8030491	MISSENSE	465	102	R	Q	hom	not called	hom	Y
CASC5	chr15	40903683	40903684	A	G	dbsnp.121:rs12911738	MISSENSE	419	87	T	A	hom	not called	hom	Y
CASC5	chr15	40913839	40913840	G	T	dbsnp.100:rs2412541	MISSENSE	1538	460	A	S	hom	not called	hom	Y
CASC5	chr15	40916236	40916237	A	G	COSMIC:mut:147883;dbsnp.123:rs17747633	MISSENSE	3935	1259	K	E	hom	hom	hom	Y
CASP2	chr7	142988735	142988736	C	G		MISSENSE	324	60	L	V	het	het	het	Y
CASP7	chr10	115439568	115439569	C	T	dbsnp.116:rs7921977	MISSENSE	141	19	T	I	hom	not called	not called	N
CASP8	chr2	202122994	202122995	A	G	dbsnp.107:rs3769823	MISSENSE	241	14	K	R	het	not called	het	Y
CBFA2T3	chr16	88951593	88951594	C	T	dbsnp.129:rs61757659	MISSENSE	1265	326	R	H	het	not called	not called	Y
CCDC50	chr3	191093174	191093175	T	A	dbsnp.94:rs2028574	MISSENSE	1362	258	I	N	het	het	het	Y
CCDC50	chr3	191093309	191093310	A	G	dbsnp.111:rs4677728	MISSENSE	1497	303	K	R	het	het	het	Y
CCDC50	chr3	191097965	191097966	T	C	COSMIC:mut:149545;dbsnp.79:rs293813	MISSENSE	1056	156	M	T	het	het	het	Y
CCDC6	chr10	61552691	61552692	G	T	COSMIC:mut:146940;dbsnp.86:rs1053266	MISSENSE	2043	470	P	T	hom	hom	hom	Y
CCL4	chr17	34432662	34432664	AT	GA	dbsnp.86:rs1049807;dbsnp.89:rs1719152	MISSENSE	315	79	ES	ET	not called	hom	hom	N
CCL4	chr17	34432663	34432664	AT	GA	rs1049807;rs1719152	MISSENSE		80	S	T	not called	not called	not called	Y
CCND3	chr6	41903781	41903783	AG	CA	dbsnp.86:rs1051130;dbsnp.106:rs3218102	MISSENSE	947	177	AS	AA	het	not called	not called	N
CCND3	chr6	41903782	41903783	AG	CA	rs1051130;rs3218102	MISSENSE		259	S	A	not called	not called	not called	Y
CCNG1	chr5	162868909	162868910	T	G	dbsnp.120:rs11541970	MISSENSE	770	179	F	L	het	het	het	Y
CD44	chr11	35226154	35226155	A	G	dbsnp.119:rs9666607	MISSENSE	1683	417	K	R	hom	hom	hom	Y
CD44	chr11	35229672	35229673	T	C	dbsnp.88:rs1467558	MISSENSE	1869	479	I	T	hom	hom	hom	Y
CDC42BPA	chr1	227182032	227182033	G	A	dbsnp.100:rs2802269	MISSENSE	6038	1699	A	V	hom	hom	hom	Y
CDC42BPA	chr1	227216774	227216775	C	T	dbsnp.92:rs1929860	MISSENSE	4852	1304	V	I	het	het	het	Y
CDC42BPG	chr11	64597505	64597506	T	C	dbsnp.107:rs3741395	MISSENSE	3403	1135	Q	R	hom	hom	hom	Y
CDH11	chr16	65016086	65016087	A	C	dbsnp.76:rs35213	MISSENSE	1550	373	S	A	het	het	het	Y
CDH11	chr16	65022233	65022234	C	T	dbsnp.86:rs1130821	MISSENSE	1258	275	M	I	het	het	het	Y
CDH3	chr16	68721532	68721533	G	C	dbsnp.86:rs1126933	MISSENSE	2820	563	Q	H	hom	hom	not called	Y
CDK1	chr10	62547869	62547870	T	G		MISSENSE		124	V	G	not called	not called	not called	Y
CDK11A	chr1	1650786	1650787	T	C	rs1137003	MISSENSE		112	H	R	not called	not called	not called	Y
CDK11A	chr1	1650796	1650797	A	G	rs1059830	MISSENSE		109	C	R	not called	not called	not called	Y
CDK11B	chr1	1650831	1650832	A	G	rs72909030	MISSENSE		97	V	A	not called	not called	not called	Y
CDK11B	chr1	1650844	1650845	G	A	rs1059831	MISSENSE		93	R	W	not called	not called	not called	Y
CDK13	chr7	40027483	40027484	A	G	rs3735135	MISSENSE		500	T	A	not called	not called	not called	Y
CDK3	chr17	73997473	73997474	G	A	rs2069528	MISSENSE		18	G	S	not called	not called	not called	Y
CDKL1	chr14	50796880	50796881	G	C	dbsnp.119:rs9323183	MISSENSE	1015	330	L	V	het	het	not called	Y

CDKL2	chr4	76521524	76521525	C	T	dbsnp.129:rs56229013	DISRUPT	1846	441		TTTTTGTCGTTACAA	not called	het	not called	Y
CDX2	chr13	28537277	28537277		C		FRAMESHIFT	1090	306	V	G	not called	not called	het	N
CDX2	chr13	28537316	28537317	G	A	dbsnp.89:rs1805107	MISSENSE	1050	293	P	S	not called	hom	hom	Y
CEP110	chr9	123903839	123903840	G	A	dbsnp.123:rs17292952	MISSENSE	2695	889	A	T	not called	not called	het	N
CFI	chr4	110678924	110678925	T	C	rs11098044	MISSENSE		308	T	A	not called	not called	not called	Y
CHD5	chr1	6171834	6171835	G	A	dbsnp.134:rs139581412	MISSENSE	5359	1750	T	M	het	not called	not called	Y
CHD5	chr1	6184091	6184092	A	G	dbsnp.100:rs2843493	MISSENSE	4725	1539	S	P	het	hom	het	Y
CHD5	chr1	6196674	6196675	G	C		NONSENSE	2708	866	Y	*	het	half called	half called	Y
CHD8	chr14	21899630	21899631	C	T	dbsnp.119:rs10467770	MISSENSE	236	58	V	M	het	half called	het	N
CHD9	chr16	53341747	53341748	T	G	dbsnp.116:rs6499548	MISSENSE	7144	2312	D	E	hom	hom	hom	Y
CHEK1	chr11	125525194	125525195	A	G	dbsnp.83:rs506504	MISSENSE	2302	471	I	V	hom	hom	hom	Y
CHEK2	chr22	29083948	29083951	CGG	TGA		MISSENSE	1766	565	PR	PH	not called	het	not called	N
CHEK2	chr22	29091787	29091788	T	C	dbsnp.137:rs200928781	MISSENSE	1369	433	Y	C	not called	not called	het	N
CHL1	chr3	367697	367698	G	A	dbsnp.134:rs150837773	MISSENSE	418	50	D	N	het	not called	het	Y
CHL1	chr3	391099	391100	A	G	dbsnp.121:rs13060847	MISSENSE	1177	303	T	A	hom	hom	hom	Y
CHL1	chr3	439962	439963	A	G	dbsnp.116:rs6442827	MISSENSE	3418	1050	I	V	hom	hom	hom	Y
CHRNA3	chr15	78913116	78913117	G	A		MISSENSE	520	7	S	L	het	not called	het	N
CHUK	chr10	101977882	101977883	C	T	dbsnp.98:rs2230804	MISSENSE	856	268	V	I	hom	hom	hom	Y
CIC	chr19	42798826	42798827	C	G		MISSENSE	4438	1467	L	V	het	het	het	Y
CIITA	chr16	10995932	10995933	A	G	rs8046121	MISSENSE		175	R	G	not called	not called	not called	Y
CIITA	chr16	11002926	11002927	A	G	dbsnp.116:rs7197779	MISSENSE	2831	900	Q	R	hom	hom	hom	Y
CLK3	chr15	74907945	74907946	A	G	dbsnp.134:rs139096840	MISSENSE	611	51	T	A	het	not called	not called	N
CLTCL1	chr22	19189003	19189003		C	dbsnp.131:rs78649162;dbsnp.120:rs11386977	FRAMESHIFT	3676	1201	V	G	hom	hom	hom	Y
CNTN5	chr11	99690285	99690286	T	G	rs10790978	MISSENSE		23	S	A	not called	not called	not called	Y
CNTN5	chr11	99690427	99690428	T	G	dbsnp.116:rs7125822	MISSENSE	573	70	L	R	het	not called	not called	Y
CNTN5	chr11	99690460	99690461	A	G	dbsnp.120:rs10893933	MISSENSE	606	81	N	S	het	not called	not called	Y
CNTN5	chr11	100061970	100061971	C	A		NONSENSE	2032	565	S	*	not called	not called	het	N
COL14A1	chr8	121298155	121298156	G	C	dbsnp.123:rs17833992	MISSENSE	4288	1342	V	L	het	het	het	Y
COL18A1	chr21	46876305	46876306	G	A	dbsnp.120:rs11702494	MISSENSE	882	288	A	T	not called	not called	het	N
COL18A1	chr21	46924425	46924434	GGCCCCCCA		dbsnp.134:rs149296338;dbsnp.129:rs56328291;dbsnp.130:rs72301048;dbsnp.125:rs28696990	DELETE	3384	1123	PPG		half called	not called	not called	N
CPAMD8	chr19	17004048	17004049	T	C	dbsnp.86:rs1054533	MISSENSE	5700	1890	Q	R	hom	half called	not called	Y
CPAMD8	chr19	17025291	17025292	G	A	dbsnp.86:rs706761	MISSENSE	3975	1315	T	I	hom	hom	hom	Y
CPAMD8	chr19	17088298	17088299	T	C	dbsnp.92:rs1824152;dbsnp.129:rs56376819	MISSENSE	1809	593	H	R	hom	hom	hom	Y
CPAMD8	chr19	17088318	17088319	G	T	dbsnp.107:rs3745335;dbsnp.129:rs55790575	MISSENSE	1789	586	D	E	hom	hom	not called	Y
CPAMD8	chr19	17108134	17108135	C	T	dbsnp.107:rs3745340	MISSENSE	1053	341	R	Q	hom	hom	not called	Y
CPAMD8	chr19	17111296	17111297	A	G	dbsnp.111:rs4808551	MISSENSE	966	312	M	T	hom	not called	hom	Y
CREB1	chr2	208442253	208442254	C	A		NONSENSE	964	238	Y	*	het	half called	het	Y
CREB3L2	chr7	137600689	137600690	C	T	dbsnp.79:rs273957	MISSENSE	782	130	V	I	hom	hom	hom	Y
CREB3L2	chr7	137612913	137612916	TGG		dbsnp.130:rs66593747;dbsnp.106:rs3217268	DELETE	693	100	T		hom	hom	hom	Y
CRTC1	chr19	18876308	18876309	A	G	dbsnp.107:rs3746266	MISSENSE	1117	344	T	A	hom	hom	hom	Y
CSF2	chr5	131411459	131411460	T	C	dbsnp.76:rs25882	MISSENSE	381	117	I	T	het	not called	not called	Y
CSMD3	chr8	113241087	113241088	T	G	dbsnp.88:rs1592624	MISSENSE	10512	3452	N	H	hom	hom	hom	Y
CSMD3	chr8	113504813	113504814	C	T		MISSENSE	5028	1624	D	N	het	het	het	Y
CSMD3	chr8	114186002	114186003	T	C	dbsnp.96:rs2219898	MISSENSE	815	219	I	M	het	het	het	Y
CSNK1A1L	chr13	37679267	37679268	G	T	dbsnp.119:rs9576175	MISSENSE	533	42	D	E	hom	hom	hom	N
CSPG4	chr15	75970069	75970070	C	T	dbsnp.116:rs8023621	MISSENSE	5200	1703	R	H	het	hom	half called	N
CSPG4	chr15	75982084	75982085	C	T	rs79463888	MISSENSE		441	E	K	not called	not called	not called	Y
CTBP2	chr10	126678091	126678092	G	T		MISSENSE	1766	445	Q	K	not called	not called	het	N
CTBP2	chr10	126678220	126678222	CT	AC		MISSENSE	1636	401	AA	AS	not called	het	not called	N
CTBP2	chr10	126715153	126715154	A	G	dbsnp.107:rs3781412	MISSENSE	1299	392	L	P	hom	half called	not called	N
CTBP2	chr10	126715159	126715159		GCCGCAGGCTGGGGCTGCAGG		INSERT	1294	390		ALQPQPA	hom	not called	not called	N

CTBP2	chr10	126715435	126715436	C	T	dbsnp.107:rs3781411	MISSENSE	1017	298	R	Q	hom	not called	half called	Y
CTNNA2	chr2	80773106	80773107	G	C		MISSENSE	1737	487	D	H	het	het	het	Y
CUBN	chr10	16943370	16943371	G	C	dbsnp.100:rs2796835	MISSENSE	8201	2717	S	W	hom	hom	hom	Y
CUBN	chr10	16948389	16948390	G	C	dbsnp.89:rs1801236;dbsnp.107:rs3740168	MISSENSE	7775	2575	P	R	not called	not called	het	Y
CUBN	chr10	16967400	16967401	C	T	dbsnp.87:rs1276712	MISSENSE	6536	2162	C	Y	hom	hom	hom	Y
CUBN	chr10	17024502	17024503	G	A	dbsnp.89:rs1801231	MISSENSE	4726	1559	P	S	hom	hom	hom	Y
CUBN	chr10	17147520	17147521	G	T	dbsnp.89:rs1801224	MISSENSE	1216	389	P	T	not called	het	not called	Y
CUBN	chr10	17156150	17156151	A	G	dbsnp.89:rs1801222	MISSENSE	809	253	F	S	hom	hom	hom	Y
CYP1B1	chr2	38298138	38298139	T	C	COSMIC:mut:1408011;dbsnp.89:rs1800440	MISSENSE	1760	453	N	S	het	not called	not called	Y
CYP1B1	chr2	38302176	38302177	C	A	dbsnp.86:rs1056827	MISSENSE	757	119	A	S	het	het	hom	Y
CYP1B1	chr2	38302389	38302390	G	C	dbsnp.52:rs10012	MISSENSE	544	48	R	G	het	het	hom	Y
DAPK1	chr9	90322022	90322023	G	A	dbsnp.86:rs1056719	MISSENSE	4411	1346	S	N	hom	hom	hom	Y
DBN1	chr5	176885483	176885484	C	A		MISSENSE	1522	451	D	Y	het	hom	het	Y
DBN1	chr5	176885498	176885499	T	C	dbsnp.100:rs2544809	MISSENSE	1507	446	I	V	hom	hom	hom	Y
DCLK2	chr4	151177339	151177341	CC	TG	dbsnp.130:rs67917234;dbsnp.121:rs13152819;dbsnp.121:rs13152820;dbsnp.129:rs60939896	MISSENSE	2995	748	P	C	hom	not called	not called	N
DFFB	chr1	3786244	3786245	G	A	dbsnp.121:rs12738235	MISSENSE	909	196	R	K	het	not called	het	Y
DGKG	chr3	185990095	185990096	C	T	dbsnp.96:rs2193587	MISSENSE	1483	316	R	K	hom	hom	hom	Y
DHFR	chr5	79950507	79950508	C	T	rs1105525	MISSENSE		83	R	Q	not called	not called	not called	Y
DHFR	chr5	79950511	79950512	A	G	rs1105524	MISSENSE		82	W	R	not called	not called	not called	Y
DKK1	chr10	54076502	54076503	G	A	dbsnp.134:rs141377301	MISSENSE	890	246	R	Q	het	not called	not called	Y
DLC1	chr8	12957474	12957475	C	T	COSMIC:mut:150456;dbsnp.83:rs532841	MISSENSE	1121	280	V	M	hom	hom	hom	Y
DLC1	chr8	13356801	13356802	G	A	dbsnp.107:rs3816747	MISSENSE	1222	260	T	I	hom	hom	hom	Y
DLC1	chr8	13356817	13356819	TT	CG	dbsnp.120:rs11203494;dbsnp.120:rs11203495	MISSENSE	1205	254	QN	HD	hom	hom	hom	N
DLC1	chr8	13356818	13356819	TT	CG	rs11203494;rs11203495	MISSENSE		254255		D	not called	not called	not called	Y
DLC1	chr8	13357339	13357340	G	C	dbsnp.107:rs3816748	MISSENSE	684	81	L	V	hom	hom	half called	Y
DLC1	chr8	13357586	13357587	G	A	rs1383942	DE_NOVO_START_OUTOFFRAME					not called	not called	not called	Y
DMPK	chr19	46283267	46283268	C	T		MISSENSE	593	17	G	E	het	not called	not called	N
DNAH8	chr6	38750887	38750888	A	G	dbsnp.107:rs3823430	MISSENSE	2316	573	I	V	hom	hom	hom	Y
DNAH8	chr6	38773292	38773293	G	A	dbsnp.86:rs874808	MISSENSE	3019	807	G	E	hom	hom	hom	Y
DNAH8	chr6	38800163	38800164	G	A	COSMIC:mut:150078;dbsnp.119:rs9357283	MISSENSE	4203	1202	E	K	hom	hom	hom	Y
DPYD	chr1	97981394	97981395	T	C	dbsnp.89:rs1801159	MISSENSE	1763	543	I	V	het	het	not called	Y
DPYD	chr1	98165090	98165091	T	C	dbsnp.100:rs2297595	MISSENSE	632	166	M	V	het	hom	het	Y
DPYD	chr1	98348884	98348885	G	A	dbsnp.89:rs1801265	MISSENSE	221	29	C	R	het	het	not called	N
DST	chr6	56327848	56327849	C	T	dbsnp.120:rs11758339	MISSENSE	15231	5042	A	T	het	not called	not called	Y
DST	chr6	56373535	56373536	C	T	dbsnp.127:rs41271862	MISSENSE	11268	3721	D	N	het	het	half called	Y
DST	chr6	56417281	56417282	C	T	dbsnp.111:rs4715630	MISSENSE	8546	2813	M	I	hom	hom	hom	Y
DST	chr6	56417544	56417545	T	C	dbsnp.111:rs4715631	MISSENSE	8283	2726	T	A	hom	hom	hom	Y
DST	chr6	56482922	56482923	A	G	dbsnp.134:rs141573097	MISSENSE	6016	1970	F	S	het	het	het	N
DSTYK	chr1	205130412	205130413	A	G	dbsnp.108:rs3851294	MISSENSE	1984	641	C	R	hom	hom	hom	Y
DSTYK	chr1	205156896	205156897	C	G		MISSENSE	366	101	E	D	het	het	not called	Y
DUSP6	chr12	89745476	89745477	C	A	dbsnp.100:rs2279574	MISSENSE	819	114	V	L	het	het	not called	Y
DUSP6	chr12	89745507	89745510	GCT			DELETE	786	103	S		het	not called	not called	N
ECM1	chr1	150484986	150484987	G	A	dbsnp.52:rs13294	MISSENSE	1441	415	G	S	het	het	het	Y
EDN1	chr6	12296254	12296255	G	T	COSMIC:mut:150053;dbsnp.52:rs5370	MISSENSE	924	197	K	N	het	het	het	Y
EEF1D	chr8	144661981	144661982	C	T	dbsnp.137:rs199856046	MISSENSE	2265	642	A	T	het	het	het	Y
EEF1D	chr8	144671684	144671685	G	C	dbsnp.116:rs6985081	MISSENSE	908	189	D	E	not called	hom	hom	N
EEF1D	chr8	144672034	144672035	C	T	dbsnp.132:rs112907282	MISSENSE	558	73	D	N	het	not called	not called	N
EGF	chr4	110883120	110883121	G	A	dbsnp.120:rs11568943	MISSENSE	1743	431	R	K	het	het	het	Y
EGF	chr4	110901197	110901198	G	A	dbsnp.98:rs2237051	MISSENSE	2575	708	M	I	hom	hom	hom	Y
EGF	chr4	110902110	110902111	A	T	dbsnp.120:rs11569017	MISSENSE	2802	784	D	V	het	het	het	Y
EGF	chr4	110914426	110914427	A	T	dbsnp.111:rs4698803	MISSENSE	3084	878	E	V	hom	hom	hom	Y
EIF2AK3	chr2	88874890	88874891	C	A	dbsnp.89:rs1805165	MISSENSE	2411	704	A	S	het	hom	half called	Y
EIF2AK3	chr2	88895122	88895123	T	C	dbsnp.52:rs13045	MISSENSE	798	166	Q	R	het	het	het	Y
EIF2AK3	chr2	88913272	88913273	G	C	dbsnp.86:rs867529	MISSENSE	708	136	S	C	het	not called	not called	Y
EIF2B5	chr3	183861242	183861243	A	G	dbsnp.86:rs843358	MISSENSE	2122	587	I	V	het	het	half called	Y
EIF3H	chr8	117661126	117661127	C	G		MISSENSE	771	249	R	T	het	not called	not called	N
EIF4B	chr12	53427597	53427598	C	G		MISSENSE	1193	330	Q	E	het	not called	not called	Y
EIF4B	chr12	53431297	53431298	T	A	dbsnp.134:rs146008363	MISSENSE	1617	471	L	Q	het	het	not called	Y
EIF4G1	chr3	184037532	184037533	A	G	rs13319149	MISSENSE		168	T	A	not called	not called	not called	Y
EIF4G1	chr3	184039665	184039666	A	G	dbsnp.96:rs2178403	MISSENSE	1585	439	M	V	het	het	het	Y
ELN	chr7	73470713	73470714	G	A	COSMIC:mut:150313;dbsnp.96:rs2071307	MISSENSE	1354	422	G	S	hom	hom	hom	Y

EML4	chr2	42515436	42515437	A	G	dbsnp.125:rs28651764	MISSENSE	1280	340	K	R	het	het	not called	Y
EPHA1	chr7	143088866	143088867	T	C	dbsnp.116:rs6967117	MISSENSE	2784	900	M	V	hom	hom	hom	Y
EPHA1	chr7	143097099	143097100	A	G	dbsnp.111:rs4725617	MISSENSE	565	160	V	A	hom	hom	hom	Y
EPHA10	chr1	38186188	38186189	C	T	dbsnp.116:rs6671088	MISSENSE	2331	749	G	E	het	het	het	Y
EPHA10	chr1	38227085	38227086	A	T	dbsnp.111:rs4653328	MISSENSE	926	281	F	I	het	het	het	N
EPHA2	chr1	16456762	16456763	C	T	dbsnp.126:rs35903225	MISSENSE	2781	876	R	H	het	half called	het	Y
EPHA3	chr3	89521692	89521693	T	C	dbsnp.126:rs35124509	MISSENSE	2994	924	W	R	hom	hom	hom	Y
EPHA8	chr1	22915752	22915753	T	C	dbsnp.83:rs606002	MISSENSE	1493	457	S	P	het	not called	het	N
EPHA8	chr1	22923872	22923873	G	C	dbsnp.86:rs999765	MISSENSE	1958	612	E	Q	het	hom	half called	Y
EPS15	chr1	51871688	51871689	C	T		MISSENSE	787	208	G	E	het	het	het	Y
ERC1	chr12	1137216	1137217	A	G	dbsnp.126:rs35037408	MISSENSE	388	50	S	G	het	het	het	Y
ERCC4	chr16	14029032	14029033	G	A	dbsnp.89:rs1800067	MISSENSE	1252	415	R	Q	het	not called	het	Y
ERCC6	chr10	50732375	50732376	G	C		MISSENSE	1253	367	S	C	het	het	het	Y
ERN2	chr16	23711924	23711925	C	G	dbsnp.76:rs26764	MISSENSE	1772	535	S	T	het	het	het	Y
ESR1	chr6	152129062	152129063	C	T	dbsnp.134:rs139960913	MISSENSE	249	6	H	Y	het	not called	het	N
ETV1	chr7	13978808	13978809	T	C	dbsnp.119:rs9639168	MISSENSE	839	100	S	G	het	het	het	Y
F12	chr5	176831825	176831826	C	G	COSMIC:mut:449394;dbsnp.124:rs17876030	MISSENSE	667	207	A	P	hom	not called	not called	N
F8	chrX	154158284	154158285	G	C	dbsnp.89:rs1800291	MISSENSE	3950	1260	D	E	hom	not called	hom	Y
FANCA	chr16	89815074	89815075	A			FRAMESHIFT	3381	1114	S	L	het	not called	not called	N
FANCA	chr16	89849479	89849480	C	T	dbsnp.98:rs2239359	MISSENSE	1542	501	G	S	het	not called	not called	Y
FANCA	chr16	89883006	89883007	A	T	dbsnp.89:rs1800282	MISSENSE	58	6	V	D	het	not called	not called	N
FANCB	chrX	14877403	14877404	C	T	dbsnp.127:rs41309679	MISSENSE	1271	335	G	E	hom	hom	hom	Y
FANCD2	chr3	10106531	10106532	C	T	dbsnp.108:rs3864017	MISSENSE	2218	714	P	L	hom	hom	hom	Y
FANCD2	chr3	10107586	10107587	A	G		MISSENSE	2386	770	K	R	not called	het	not called	N
FANCD2	chr3	10114943	10114944	A	C	dbsnp.129:rs56041034;dbsnp.129:rs61744531	MISSENSE	2690	871	K	N	not called	half called	not called	N
FANCI	chr15	89804042	89804043	C	T	dbsnp.123:rs17803620	MISSENSE	346	86	A	V	hom	hom	hom	Y
FANCI	chr15	89836227	89836228	G	C	dbsnp.100:rs2283432	MISSENSE	2314	742	C	S	hom	half called	hom	Y
FBN1	chr15	48807636	48807637	C	T	rs4775765	MISSENSE		472	C	Y	not called	not called	not called	Y
FBN2	chr5	127685134	127685135	C	T	dbsnp.79:rs154001	MISSENSE	3331	965	V	I	het	het	het	Y
FCGR2B	chr1	161643797	161643798	T	C	dbsnp.86:rs1050501	MISSENSE	818	231	I	T	not called	het	not called	N
FGFR4	chr5	176517796	176517797	C	T	dbsnp.80:rs376618	MISSENSE	573	136	P	L	het	het	het	Y
FLNB	chr3	58110118	58110119	G	C	dbsnp.132:rs111330368	MISSENSE	3949	1262	G	A	hom	hom	hom	Y
FLT1	chr13	28971099	28971101	TG	AA		MISSENSE	1940	552	IT	IS	not called	not called	het	N
FLT3	chr13	28624293	28624294	G	A	dbsnp.92:rs1933437	MISSENSE	761	227	T	M	hom	hom	hom	Y
FLT3	chr13	28674627	28674628	T	C	dbsnp.121:rs12872889	MISSENSE	101	7	D	G	hom	hom	not called	N
FLT4	chr5	180046343	180046344	G	C	COSMIC:mut:449470;COSMIC:mut:1486680;dbsnp.80:rs448012	MISSENSE	2748	890	H	Q	het	not called	not called	N
FLT4	chr5	180048661	180048662	C	T		MISSENSE	1978	634	A	T	het	not called	not called	Y
FMN2	chr1	240255568	240255571	GGC		COSMIC:mut:244564;dbsnp.134:rs140531536;dbsnp.130:rs72215772;dbsnp.130:rs71929261	DELETE	384	58	G		not called	het	not called	N
FMN2	chr1	240371553	240371554	A	G	rs12732924	MISSENSE		1152	R	G	not called	not called	not called	Y
FMN2	chr1	240492733	240492734	G	A	dbsnp.107:rs3795677	MISSENSE	4627	1468	R	H	het	het	het	Y
FN1	chr2	216300479	216300482	ACT	ACA	dbsnp.87:rs1250259	MISSENSE	309	15	Q	L	not called	half called	not called	N
FN1	chr2	216300481	216300482	T	A	dbsnp.87:rs1250259	MISSENSE	309	15	Q	L	hom	not called	hom	Y
FNBP1	chr9	132662785	132662786	C	T	dbsnp.86:rs1023000	MISSENSE	1687	490	S	N	het	het	het	Y
FSCB	chr14	44974563	44974564	G	A		NONSENSE	1935	543	Q	*	het	not called	not called	Y
FSCB	chr14	44974921	44974922	A	T	dbsnp.107:rs3825632	MISSENSE	1577	423	D	E	het	het	not called	Y
FSCB	chr14	44974965	44974966	G	A	dbsnp.92:rs1959379	MISSENSE	1533	409	P	S	het	het	half called	Y
FSCB	chr14	44975051	44975052	A	G	COSMIC:mut:147770;dbsnp.107:rs3825630	MISSENSE	1447	380	L	P	het	het	het	Y
FSCB	chr14	44975605	44975606	G	T	COSMIC:mut:147771;dbsnp.107:rs3809429	MISSENSE	893	195	H	Q	het	het	het	Y
GAB1	chr4	144359489	144359490	C	T	dbsnp.125:rs28925904	MISSENSE	1290	311	P	L	het	het	het	Y
GAK	chr4	860922	860923	G	A	dbsnp.137:rs201137296	MISSENSE	2891	898	P	L	het	not called	not called	Y
GCNT2	chr6	10586726	10586727	G	A	dbsnp.129:rs56106312	MISSENSE	734	169	A	T	het	hom	half called	N
GCNT2	chr6	10587037	10587038	C	G	dbsnp.83:rs539351	MISSENSE	1045	272	D	E	hom	hom	hom	N
GHR	chr5	42718153	42718154	G	T		MISSENSE	918	292	R	S	het	not called	het	Y
GHR	chr5	42719238	42719239	A	C	dbsnp.52:rs6180	MISSENSE	1672	544	I	L	het	het	het	Y
GLI1	chr12	57865320	57865321	G	A	dbsnp.98:rs2228224	MISSENSE	2671	805	G	D	hom	hom	hom	Y
GLI1	chr12	57865820	57865821	G	C	dbsnp.98:rs2228226	MISSENSE	3171	972	E	Q	hom	hom	hom	Y
GLI2	chr2	121746955	121746956	G	T	dbsnp.107:rs3738880	MISSENSE	3495	1156	A	S	hom	hom	hom	Y
GLI2	chr2	121747405	121747406	G	A	dbsnp.121:rs12711538	MISSENSE	3945	1306	D	N	hom	hom	half called	Y

GLI3	chr7	42005677	42005678	G	A	dbsnp.86:rs929387	MISSENSE	3083	998	P	L	het	het	het	N
GLI3	chr7	42088221	42088222	T	C	dbsnp.86:rs846266	MISSENSE	637	183	T	A	het	het	het	N
GLTSCR1	chr19	48184473	48184474	C	T	rs3745762	MISSENSE		683	P	S	not called	not called	not called	Y
GLYAT	chr11	58478083	58478084	T	C	dbsnp.83:rs675815	MISSENSE	621	156	N	S	hom	hom	hom	Y
GLYAT	chr11	58491920	58491921	A	T	dbsnp.120:rs10896818	MISSENSE	203	17	S	T	het	het	het	Y
GLYATL2	chr11	58602308	58602309	C	T	COSMIC:mut:147251;dbsnp.120:rs11229651	MISSENSE	608	160	E	K	het	het	het	Y
GLYATL2	chr11	58605841	58605842	C	A		DISRUPT	208	26		TCTGTTGCATCGTAT	not called	not called	het	N
GOLGA5	chr14	93276653	93276654	T	C	dbsnp.86:rs1040835	MISSENSE	1229	350	F	L	het	het	het	Y
GPR177	chr1	68603585	68603586	C	T	dbsnp.86:rs983034	MISSENSE	1633	463	V	I	not called	het	het	Y
GRIA3	chrX	122318386	122318386		G	rs66632982	SPLICE_SITE_INS					not called	not called	not called	Y
GRK4	chr4	3039149	3039150	T	C	dbsnp.89:rs1801058	MISSENSE	1815	454	V	A	het	het	het	Y
GRK7	chr3	141535557	141535558	A	G	dbsnp.126:rs36009541	MISSENSE	1411	443	E	G	het	het	het	Y
GRK7	chr3	141535607	141535608	C	A	dbsnp.126:rs33928105	MISSENSE	1461	460	P	T	het	het	het	Y
GUCY2C	chr12	14829892	14829893	A	C	dbsnp.88:rs1420635	MISSENSE	979	281	F	L	hom	half called	hom	Y
GUCY2F	chrX	108708515	108708516	C	T	dbsnp.83:rs502209	MISSENSE	1162	296	R	Q	hom	hom	hom	Y
GUCY2F	chrX	108708551	108708552	A	G	dbsnp.120:rs12008095	MISSENSE	1126	284	L	P	hom	hom	hom	Y
HDLBP	chr2	242192847	242192848	T	C	dbsnp.116:rs7578199	MISSENSE	1624	418	N	S	het	half called	het	Y
HECW1	chr7	43483980	43483981	G	C	dbsnp.129:rs61756574	MISSENSE	1814	404	G	R	het	het	het	Y
HEPH	chrX	65382684	65382685	T	C	dbsnp.114:rs5919015	MISSENSE	252	39	V	A	hom	hom	hom	N
HERC2	chr15	28419618	28419619	C	T		MISSENSE		3327	V	M	not called	not called	not called	Y
HERC2	chr15	28441716	28441717	T	C	dbsnp.100:rs2428638	DISRUPT	8116	2671		TCTGTTCTGTCCTGG	not called	het	not called	N
HERC2	chr15	28443773	28443774	C	A		MISSENSE	7963	2620	V	F	not called	het	not called	N
HERC2	chr15	28446642	28446643	T	C	dbsnp.129:rs61756158	MISSENSE	7780	2559	S	G	not called	het	not called	N
HERC2	chr15	28446650	28446651	T	C		MISSENSE	7772	2556	D	G	not called	het	not called	N
HERC2	chr15	28446658	28446659	T	G	dbsnp.129:rs61756159	MISSENSE	7764	2553	K	N	not called	het	not called	N
HERC2	chr15	28447501	28447502	T	C	dbsnp.134:rs140027229	MISSENSE	7576	2491	S	G	not called	het	not called	N
HERC2	chr15	28456219	28456220	G	A	dbsnp.100:rs2525916	MISSENSE	7102	2333	L	F	not called	het	het	N
HERC2	chr15	28465642	28465644	CA	GG		MISSENSE	5907	1933	AA	AP	half called	not called	not called	N
HERC2	chr15	28483864	28483865	G	A		MISSENSE		1211	R	C	not called	not called	not called	Y
HERC2	chr15	28483902	28483903	C	A		MISSENSE		1198	G	V	not called	not called	not called	Y
HERC2	chr15	28501066	28501067	T	G		MISSENSE		941	D	A	not called	not called	not called	Y
HERC2	chr15	28501100	28501101	T	C		MISSENSE		930	M	V	not called	not called	not called	Y
HERC2	chr15	28501377	28501379	GT	CC		MISSENSE	2707	868	T	G	not called	not called	het	N
HERC2	chr15	28544553	28544554	T	C	dbsnp.100:rs2638727;dbsnp.137:rs200922469	MISSENSE	289	61	R	G	het	not called	not called	N
HIF1A	chr14	62207574	62207575	G	A	dbsnp.120:rs11549467	MISSENSE	2062	612	A	T	het	not called	not called	Y
HIPK2	chr7	139249947	139249947		A		UNKNOWN-TR					het	not called	not called	N
HIPK2	chr7	139252779	139252780	T	C	dbsnp.89:rs1638195	UNKNOWN-TR					hom	not called	not called	N
HIPK2	chr7	139415774	139415775	G	C	dbsnp.116:rs7456421	UNKNOWN-TR					hom	not called	not called	N
HNF1A	chr12	121435449	121435450	C	T		NONSENSE	1505	495	Q	*	het	het	het	Y
HNF1A	chr12	121437381	121437382	A	G	rs1169305	MISSENSE		581	S	G	not called	not called	not called	Y
HOXA13	chr7	27238042	27238043	G	A		MISSENSE	969	314	S	L	het	not called	not called	Y
HSP90AA1	chr14	102568366	102568367	T	A	dbsnp.116:rs8005905	MISSENSE	555	71	M	L	hom	hom	hom	Y
HSP90B1	chr12	104336552	104336553	G	A		MISSENSE	1727	541	M	I	het	het	not called	Y
HUNK	chr21	33371122	33371123	C	T	dbsnp.120:rs10775648	MISSENSE	2130	591	R	C	het	het	het	Y
IKBKE	chr1	206669464	206669465	C	T	dbsnp.107:rs3748022	MISSENSE	2596	628	P	L	het	het	het	Y
IL17RD	chr3	57136584	57136585	C	T	dbsnp.123:rs17057718	MISSENSE	989	301	V	M	hom	hom	hom	Y
IL3	chr5	131396477	131396478	C	T	dbsnp.76:rs40401	MISSENSE	131	27	P	S	het	het	not called	Y
ING1	chr13	111368163	111368164	T	G	dbsnp.116:rs7338333	MISSENSE	805	125	L	R	hom	hom	hom	N
INSR	chr19	7293897	7293898	G	C	dbsnp.116:rs7508518	MISSENSE	113	2	A	G	hom	hom	hom	N
IRAK1	chrX	153278828	153278829	G	A	dbsnp.86:rs1059703	MISSENSE	1436	453	S	L	hom	hom	hom	N
IRAK1	chrX	153284191	153284192	A	G	dbsnp.86:rs1059702	MISSENSE	665	196	F	S	hom	hom	half called	Y
IRAK2	chr3	10264479	10264480	C	G	dbsnp.108:rs3844283	MISSENSE	1249	392	L	V	hom	hom	half called	Y
IRAK2	chr3	10276162	10276163	T	A	dbsnp.86:rs708035	MISSENSE	1368	431	D	E	hom	hom	hom	Y
IRAK3	chr12	66605227	66605228	A	G	dbsnp.87:rs1152888	MISSENSE	357	86	I	V	hom	hom	half called	Y
IRAK4	chr12	44180294	44180295	G	A	dbsnp.111:rs4251545	MISSENSE	1411	428	A	T	het	het	het	Y

IRS1	chr2	227659925	227659926	G	A		MISSENSE	3580	1177	L	F	het	not called	not called	Y
IRS2	chr13	110435230	110435231	C	T	dbsnp.89:rs1805097	MISSENSE	3683	1057	G	D	hom	not called	not called	N
ISG15	chr1	949607	949608	G	A	dbsnp.36:rs1921	MISSENSE	354	83	S	N	hom	hom	hom	Y
ITGA7	chr12	56089356	56089357	C	T	dbsnp.89:rs1800974	MISSENSE	2183	655	R	H	het	het	het	Y
ITGA7	chr12	56106046	56106047	G	A		MISSENSE	42	3	P	S	not called	not called	het	N
ITGAV	chr2	187541984	187541985	C	T		MISSENSE		1038	P	L	not called	not called	not called	Y
ITGB4	chr17	73753502	73753503	T	C	dbsnp.86:rs871443	MISSENSE	5522	1779	L	P	hom	hom	not called	Y
ITPR2	chr12	26834804	26834804		ACTC	rs66696203;rs59199365	SPLICE_SITE_INS		470	R		not called	not called	not called	Y
ITPR2	chr12	26835595	26835596	C	T		MISSENSE	1575	387	V	I	het	het	het	Y
JAG2	chr14	105617041	105617042	C	T	dbsnp.86:rs1057744	MISSENSE	1904	501	E	K	hom	hom	hom	N
KALRN	chr3	124053271	124053272	T	G		MISSENSE	1697	524	I	S	het	not called	not called	N
KALRN	chr3	124114182	124114183	G	A		MISSENSE	2284	720	E	K	het	not called	not called	Y
KALRN	chr3	124376307	124376308	G	C		MISSENSE	5999	1958	R	T	het	not called	not called	Y
KALRN	chr3	124385379	124385380	A	C	dbsnp.134:rs138817318	MISSENSE	6553	2143	K	Q	het	het	het	Y
KALRN	chr3	124390721	124390722	G	A	dbsnp.126:rs35653635	MISSENSE	7042	2306	G	R	het	het	het	Y
KCNH2	chr7	150645533	150645534	T	G	dbsnp.89:rs1805123	MISSENSE	2702	897	K	T	het	not called	het	Y
KDM5A	chr12	427574	427575	A	G	dbsnp.120:rs11062385	MISSENSE	2956	865	M	T	het	het	het	Y
KDM6A	chrX	44929076	44929077	C	A	dbsnp.98:rs2230018	MISSENSE	2551	726	T	K	hom	hom	hom	Y
KDR	chr4	55972945	55972946	A	G	dbsnp.126:rs34231037	MISSENSE	1745	482	C	R	het	het	het	Y
KDR	chr4	55972973	55972974	T	A	COSMIC:mut:149673;dbsnp.92:rs1870377	MISSENSE	1717	472	Q	H	het	het	het	Y
KIAA0802	chr18	8783834	8783835	T	C	dbsnp.126:rs35739383	MISSENSE	866	242	M	T	not called	het	het	N
KIAA0802	chr18	8784611	8784612	A	G	dbsnp.92:rs1965665	MISSENSE	1643	501	Q	R	not called	hom	hom	N
KIAA1409	chr14	94088368	94088369	T	C	dbsnp.125:rs28670114	MISSENSE	4913	1420	V	A	not called	hom	hom	N
KIAA1549	chr7	138588409	138588410	T	C	dbsnp.134:rs144488000	MISSENSE	3641	1198	Q	R	het	het	het	Y
KLK7	chr19	51485621	51485622	A	G	dbsnp.100:rs2659067	MISSENSE	305	54	L	P	hom	not called	not called	N
KLK7	chr19	51487174	51487175	T	G	dbsnp.89:rs1624358	MISSTART	145	1	M	L	hom	not called	not called	N
LAMA1	chr18	6980522	6980523	T	C	dbsnp.83:rs607230	MISSENSE	6097	2002	K	E	het	het	het	Y
LAMA1	chr18	6997817	6997818	A	C	dbsnp.121:rs12961939	MISSENSE	4822	1577	S	A	het	het	het	Y
LAMA1	chr18	7008590	7008591	T	C	dbsnp.83:rs662471	MISSENSE	4111	1340	M	V	het	het	het	Y
LATS1	chr6	150023074	150023075	C	T		MISSENSE	735	63	R	Q	het	not called	het	Y
LATS2	chr13	21562831	21562832	C	T	dbsnp.100:rs2770928	MISSENSE	1528	363	G	S	hom	hom	hom	N
LATS2	chr13	21562947	21562948	G	A	dbsnp.83:rs558614	MISSENSE	1412	324	A	V	hom	hom	hom	N
LCP1	chr13	46708290	46708291	T	C	dbsnp.111:rs4941543	MISSENSE	1834	533	K	E	hom	hom	hom	Y
LCP1	chr13	46722554	46722556	CA	TG		MISSENSE	1146	303	LE	LK	not called	not called	het	N
LDLR	chr19	11218095	11218096	C	G		MISSENSE	1032	282	F	L	het	not called	not called	Y
LIMK2	chr22	31658204	31658205	C	T	dbsnp.126:rs34930775	MISSENSE	928	192	R	C	hom	hom	hom	Y
LMTK3	chr19	48994757	48994757		G		FRAMESHIFT	4218	1407	E	R	not called	het	not called	N
LRGUK	chr7	133848256	133848257	G	T	dbsnp.123:rs17167553	MISSENSE	919	302	D	Y	het	het	het	Y
LRGUK	chr7	133906668	133906669	C	T	dbsnp.126:rs35149449	MISSENSE	1997	661	A	V	het	het	het	Y
LRGUK	chr7	133932356	133932357	T	C	dbsnp.126:rs34904752	MISSENSE	2048	678	M	T	het	het	het	Y
LRP1	chr12	57589783	57589784	A	C	dbsnp.116:rs7397167	MISSENSE	9164	2900	Q	P	hom	hom	hom	Y
LRP1	chr12	57603939	57603939		C	COSMIC:mut:87951	FRAMESHIFT	13033	4192	PD	PR	not called	het	not called	N
LRP1B	chr2	141232770	141232772	TC	GT		MISSENSE	10531	3187	R	N	not called	het	not called	N
LRP1B	chr2	141777553	141777554	C	T	dbsnp.131:rs77234491	MISSENSE	2878	636	R	Q	het	not called	not called	Y
LRP2	chr2	169997024	169997024		G		FRAMESHIFT	13352	4380	PC	PM	not called	het	het	N
LRP2	chr2	170003431	170003432	T	G	dbsnp.111:rs4667591	MISSENSE	12840	4210	I	L	hom	hom	hom	Y
LRP2	chr2	170010984	170010985	T	C	dbsnp.96:rs2075252	MISSENSE	12492	4094	K	E	hom	hom	hom	Y
LRP2	chr2	170011040	170011041	G	A		MISSENSE	12436	4075	S	L	het	not called	het	Y
LRP6	chr12	12301897	12301898	C	T	dbsnp.100:rs2302685	MISSENSE	3325	1062	V	I	hom	hom	hom	Y
LRRC2	chr3	46580590	46580591	G	T	dbsnp.123:rs17078944	MISSENSE	797	145	A	E	hom	hom	hom	Y
LRRC2	chr3	46586621	46586622	T	C	dbsnp.123:rs17286758	MISSENSE	610	83	T	A	hom	hom	hom	Y
LRRC2	chr3	46592986	46592987	A	G	dbsnp.125:rs28687398	MISSENSE	458	32	V	A	hom	hom	hom	Y
LRRK2	chr12	40713900	40713901	T	A	dbsnp.120:rs11564148	MISSENSE	5059	1647	S	T	het	not called	not called	Y
LRRK2	chr12	40758651	40758652	T	C	dbsnp.107:rs3761863	MISSENSE	7310	2397	M	T	hom	hom	hom	Y
LSP1	chr11	1887805	1887806	T	A	dbsnp.116:rs7938342	MISSENSE	209	34	H	Q	hom	not called	not called	N
LSP1	chr11	1902767	1902768	G	A	dbsnp.83:rs621679	MISSENSE	558	38	A	T	hom	hom	hom	N
MAGEB16	chrX	35820424	35820425	C	T	dbsnp.88:rs1410961	MISSENSE	390	38	L	F	hom	het	hom	Y
MAGEB16	chrX	35820695	35820696	G	A	dbsnp.88:rs1410962	MISSENSE	661	128	C	Y	hom	hom	hom	Y
MAGEB16	chrX	35820794	35820795	A	G	COSMIC:mut:150777;dbsnp.114:rs5973488	MISSENSE	760	161	H	R	hom	hom	hom	Y

MAGEB16	chrX	35821054	35821056	AT	GA	dbsnp.111:rs4829390;dbsnp.111:rs4829391;dbsnp.131:rs78330639	MISSENSE	1020	248	M	E	hom	hom	hom	N
MAGEB16	chrX	35821055	35821056	AT	GA	rs4829390;rs78330639	MISSENSE		248	M	E	not called	not called	not called	Y
MAGEB16	chrX	35821126	35821127	C	T	dbsnp.111:rs4829392	NONSENSE	1092	272	R	*	hom	hom	hom	Y
MAK	chr6	10775599	10775600	G	A	dbsnp.83:rs567083	MISSENSE	1786	520	P	S	het	het	het	Y
MAML3	chr4	140640702	140640703	G	T	dbsnp.129:rs61747885	MISSENSE	3927	1059	P	H	het	not called	not called	Y
MAML3	chr4	140651584	140651587	CTG			SPLICE_SITE_DEL		767	Q		not called	not called	not called	Y
MAP2K3	chr17	21202190	21202191	C	A	dbsnp.126:rs33911218	MISSENSE	124	11	P	T	het	het	het	Y
MAP2K3	chr17	21202236	21202237	G	C	dbsnp.126:rs36047035	MISSENSE	170	26	R	T	het	het	het	Y
MAP2K3	chr17	21203892	21203893	T	C	dbsnp.126:rs34105301	MISSENSE	208	39	S	P	het	het	het	Y
MAP2K3	chr17	21203940	21203941	G	A	dbsnp.100:rs2305873	MISSENSE	256	55	A	T	het	het	het	N
MAP2K3	chr17	21204186	21204187	G	T	COSMIC:mut:288153;dbsnp.129:rs56067280	MISSENSE	287	65	R	L	het	het	het	N
MAP2K3	chr17	21204191	21204192	C	T	dbsnp.129:rs56216806	MISSENSE	292	67	R	W	het	het	het	N
MAP2K3	chr17	21204209	21204210	C	T	dbsnp.129:rs55796947	NONSENSE	310	73	Q	*	het	het	het	N
MAP2K3	chr17	21207812	21207813	T	G	dbsnp.131:rs74575904	MISSENSE	650	186	L	W	not called	het	not called	N
MAP2K3	chr17	21207833	21207834	C	T	dbsnp.129:rs58609466	MISSENSE	671	193	T	M	het	not called	het	N
MAP2K3	chr17	21207834	21207835	CG	TA	rs58609466;rs76111309	MISSENSE		222	T	I	not called	not called	not called	Y
MAP2K3	chr17	21215556	21215557	G	A	dbsnp.126:rs35206134	MISSENSE	884	264	R	H	het	het	not called	Y
MAP2K3	chr17	21217512	21217513	G	A	dbsnp.100:rs2363198	MISSENSE	1021	310	V	M	het	het	het	Y
MAP3K1	chr5	56177442	56177443	G	A	dbsnp.86:rs702689	MISSENSE	2916	806	D	N	hom	hom	hom	Y
MAP3K1	chr5	56177742	56177743	G	A	dbsnp.86:rs832582	MISSENSE	3216	906	V	I	hom	hom	hom	Y
MAP3K1	chr5	56177848	56177851	CAA			IN_FRAME_DEL		949	T		not called	not called	not called	Y
MAP3K11	chr11	65373549	65373550	C	G		MISSENSE		536	E	Q	not called	not called	not called	Y
MAP3K14	chr17	43364293	43364293		G	rs66766023	SPLICE_SITE_INS		218	R		not called	not called	not called	Y
MAP3K6	chr1	27688632	27688633	G	A	dbsnp.86:rs1138294	MISSENSE	1612	455	T	I	hom	half called	hom	Y
MAP3K8	chr10	30740611	30740612	T	A		MISSENSE	1507	271	F	Y	het	het	not called	Y
MAP4K3	chr2	39515383	39515384	G	A		MISSENSE	1442	451	T	I	het	not called	het	Y
MAPK1	chr22	22162071	22162072	G	C		MISSENSE	422	61	H	Q	het	het	het	Y
MAPK10	chr4	87028478	87028479	C	T		MISSENSE	484	88	R	K	het	het	not called	Y
MAPK13	chr6	36107086	36107087	G	C	dbsnp.132:rs111849509	MISSENSE	1132	345	E	D	het	het	het	Y
MAPK15	chr8	144801592	144801593	C	A	dbsnp.129:rs60732298	MISSENSE	702	221	T	K	het	het	het	Y
MAPK15	chr8	144803272	144803273	C	G		MISSENSE	1061	341	Q	E	het	het	not called	N
MAPKBP1	chr15	42105917	42105918	C	G	dbsnp.87:rs1201689	MISSENSE	1222	313	L	V	hom	hom	hom	Y
MARK3	chr14	103934479	103934489	AAGTGTTTTT	AAGTGTTTCT	dbsnp.119:rs10137161	MISSENSE	1886	410	F	S	not called	half called	not called	N
MARK3	chr14	103934487	103934488	T	C	dbsnp.119:rs10137161	MISSENSE	1894	410	F	S	hom	not called	hom	Y
MARK4	chr19	45790757	45790758	G	T		NONSENSE	1660	444	E	*	het	not called	not called	N
MAST2	chr1	46476586	46476587	T	G	dbsnp.120:rs11211247	MISSENSE	1446	388	D	E	hom	hom	hom	Y
MAST2	chr1	46493459	46493460	T	G	dbsnp.89:rs1707336	MISSENSE	2259	659	I	M	hom	hom	hom	Y
MAST2	chr1	46499525	46499526	A	G	dbsnp.86:rs1052607	MISSENSE	3872	1197	K	R	het	het	het	Y
MAST2	chr1	46500250	46500251	G	A	dbsnp.126:rs33931638	MISSENSE	4192	1304	V	M	het	het	het	Y
MAST2	chr1	46500992	46500993	A	G	dbsnp.86:rs1052610	MISSENSE	4934	1551	D	G	het	half called	not called	Y
MAST3	chr19	18255358	18255359	G	A	dbsnp.116:rs8108738	MISSENSE	2580	861	G	S	hom	hom	hom	Y
MAST4	chr5	65892767	65892767		GCC	dbsnp.137:rs200514960;dbsnp.137:rs201910335;dbsnp.137:rs200017963	INSERT	592	99		P	het	hom	not called	N
MAST4	chr5	66459877	66459878	G	C	dbsnp.89:rs1705399	MISSENSE	5178	1624	R	P	hom	hom	hom	Y
MBIP	chr14	36789728	36789729	T	G	dbsnp.105:rs3168891	MISSENSE	153	22	R	S	hom	hom	hom	Y
MBIP	chr14	36789774	36789775	A	T	dbsnp.101:rs2899849	MISSENSE	107	7	L	H	hom	hom	hom	Y
MCM3AP	chr21	47704895	47704896	G	A	dbsnp.119:rs9975588	MISSENSE	340	102	S	L	het	het	het	Y
MCM4	chr8	48885435	48885436	T	A	dbsnp.86:rs762679	MISSENSE	2158	650	L	M	not called	hom	hom	Y
MERTK	chr2	112686987	112686988	G	A	dbsnp.121:rs13027171	MISSENSE	474	118	S	N	het	not called	not called	Y
MERTK	chr2	112751927	112751928	G	A	dbsnp.116:rs7604639	MISSENSE	1518	466	R	K	hom	hom	hom	Y
MERTK	chr2	112755000	112755001	A	G	dbsnp.98:rs2230515	MISSENSE	1673	518	I	V	hom	hom	hom	Y
MFSD4	chr1	205554084	205554085	G	C	dbsnp.116:rs7526132	MISSENSE	1026	314	G	A	het	het	not called	Y
MGA	chr15	41991314	41991315	A	T	dbsnp.96:rs2178004	MISSENSE	2326	716	T	S	hom	hom	hom	Y
MGA	chr15	42041742	42041743	G	A		MISSENSE	5491	1771	E	K	het	het	het	Y
MINK1	chr17	4799545	4799546	A	T		MISSENSE	3707	1155	Q	L	hom	hom	hom	Y
MKNK2	chr19	2050822	2050823	G	T	dbsnp.107:rs3746101	MISSENSE	272	10	Q	K	hom	hom	half called	N
MLF1	chr3	158320702	158320703	C	A	dbsnp.52:rs15967;dbsnp.130:rs77911695	MISSENSE	856	201	P	T	het	not called	het	Y
MLL	chr11	118360517	118360519	GT	TA		MISSENSE	4513	1497	EC	DS	not called	het	not called	N

MLL	chr11	118365017	118365018	G	A		MISSENSE	5207	1729	D	N	het	not called	het	Y
MLL3	chr7	151927015	151927016	T	C		MISSENSE		990	S	G	not called	not called	not called	Y
MLL3	chr7	151927020	151927021	C	A	rs28522267	MISSENSE		988	C	F	not called	not called	not called	Y
MLL3	chr7	151927025	151927026	AT	GC	rs28439884	MISSENSE		987	Y	H	not called	not called	not called	Y
MLL3	chr7	151927350	151927351	T	G	dbsnp.131:rs75934016;dbsnp.137:rs201118650	MISSENSE	3043	942	N	T	not called	het	not called	N
MLL3	chr7	151945006	151945007	C	T		MISSENSE		838	G	S	not called	not called	not called	Y
MLL3	chr7	151945333	151945334	T	C	dbsnp.111:rs4639425;dbsnp.131:rs77255032	MISSENSE	2403	729	N	D	not called	het	not called	N
MLL3	chr7	151970855	151970856	T	A	rs10454320	MISSENSE		316	T	S	not called	not called	not called	Y
MLL3	chr7	151970930	151970931	G	A	dbsnp.129:rs56850341;dbsnp.131:rs74363400	MISSENSE	1089	291	L	F	het	het	het	Y
MMP20	chr11	102495997	102495998	T	G	dbsnp.100:rs2245803	MISSENSE	65	18	K	T	het	het	het	Y
MMP9	chr20	44640224	44640225	A	G	dbsnp.63:rs17576	MISSENSE	854	279	Q	R	het	het	not called	Y
MMP9	chr20	44642405	44642406	G	C	dbsnp.100:rs2250889	MISSENSE	1739	574	R	P	hom	hom	hom	N
MMP9	chr20	44643110	44643111	G	A	dbsnp.63:rs17577	MISSENSE	2021	668	R	Q	het	het	not called	Y
MNX1	chr7	156801699	156801700	G	T	dbsnp.131:rs78596384	MISSENSE	429	16	A	D	het	het	het	N
MSH3	chr5	79950780	79950781	A	G	dbsnp.89:rs1650697	MISSENSE	487	79	I	V	het	not called	half called	N
MSH3	chr5	79970927	79970929	AC	TT		MISSENSE	1406	385	N	I	not called	het	not called	N
MSH3	chr5	80149980	80149981	A	G	dbsnp.79:rs184967	MISSENSE	3098	949	Q	R	hom	hom	hom	N
MSH3	chr5	80168936	80168937	G	A	dbsnp.76:rs26279	MISSENSE	3385	1045	A	T	hom	hom	hom	Y
MSH6	chr2	48010487	48010488	G	A	dbsnp.86:rs1042821	MISSENSE	267	39	G	E	het	not called	not called	Y
MST1R	chr3	49924939	49924940	T	C	dbsnp.86:rs1062633	MISSENSE	4266	1335	R	G	hom	hom	hom	Y
MST1R	chr3	49936101	49936102	T	C	dbsnp.98:rs2230590	MISSENSE	1831	523	Q	R	hom	hom	hom	Y
MTMR11	chr1	149906166	149906167	T	G	dbsnp.127:rs41302101	MISSENSE	850	200	E	D	het	het	het	Y
MTMR11	chr1	149906412	149906413	T	C	COSMIC:mut:146598;dbsnp.120:rs11205303	MISSENSE	725	159	M	V	het	het	het	Y
MTUS2	chr13	29898767	29898768	A	C	rs928661	MISSENSE		952	Q	P	not called	not called	not called	Y
MTUS2	chr13	30054396	30054397	G	A		MISSENSE		1109	E	K	not called	not called	not called	Y
MUSK	chr9	113457798	113457799	A	G	dbsnp.126:rs35176182	MISSENSE	608	159	S	G	het	het	het	Y
MYCL1	chr1	40363053	40363054	G	C	dbsnp.103:rs3134614	MISSENSE	1665	362	T	S	hom	hom	hom	Y
MYH11	chr16	15797966	15797967	T	A	dbsnp.132:rs113667224	MISSENSE	5927	1941	T	S	het	het	het	Y
MYH9	chr22	36684353	36684354	T	C	dbsnp.100:rs2269529	MISSENSE	5106	1626	I	V	hom	hom	hom	Y
MYLK	chr3	123451772	123451773	G	C	dbsnp.119:rs9833275	MISSENSE	1767	496	L	V	hom	hom	hom	Y
MYLK	chr3	123457892	123457893	G	A	dbsnp.119:rs9840993	MISSENSE	720	147	P	S	hom	hom	hom	Y
MYO18B	chr22	26159288	26159289	G	A	dbsnp.78:rs133885	MISSENSE	380	44	G	E	hom	hom	hom	Y
MYO18B	chr22	26166899	26166900	G	C	dbsnp.108:rs3859866	MISSENSE	1890	547	W	C	hom	hom	hom	Y
MYO18B	chr22	26173660	26173661	T	C	dbsnp.114:rs5761170	MISSENSE	2230	661	W	R	hom	hom	hom	N
MYO18B	chr22	26239849	26239850	C	A	dbsnp.114:rs5761268	MISSENSE	3606	1119	H	Q	hom	hom	hom	Y
MYO18B	chr22	26422564	26422565	G			FRAMESHIFT	6874	2209	E	K	not called	not called	het	N
MYO3A	chr10	26355991	26355992	A	G	dbsnp.107:rs3824699	MISSENSE	1401	348	I	V	het	not called	het	Y
MYO3A	chr10	26357747	26357748	G	A	rs3817420	MISSENSE		369	V	I	not called	not called	not called	Y
MYO3A	chr10	26463129	26463130	C	A	dbsnp.92:rs1999240	MISSENSE	4296	1313	R	S	het	het	not called	Y
MYO3B	chr2	171225840	171225841	A	G	dbsnp.111:rs4668246	MISSENSE	1067	309	K	E	hom	hom	hom	Y
MYO3B	chr2	171260786	171260787	G	A	dbsnp.116:rs6736609	MISSENSE	2450	770	V	I	hom	hom	hom	Y
MYO3B	chr2	171260796	171260797	A	G	dbsnp.126:rs33962844	MISSENSE	2460	773	E	G	hom	hom	hom	Y
MYO3B	chr2	171356273	171356274	G	A	dbsnp.119:rs10185178	MISSENSE	3387	1082	R	K	hom	not called	hom	N
N4BP2	chr4	40104587	40104588	G	T		NONSENSE	1460	375	G	*	het	het	not called	Y
N4BP2	chr4	40121561	40121562	G	A	dbsnp.86:rs794001	MISSENSE	2168	611	D	N	hom	hom	hom	Y
NACA	chr12	57109791	57109792	A	G	dbsnp.101:rs2958149	MISSENSE	5803	1841	L	P	hom	hom	hom	Y
NACA	chr12	57109930	57109931	A	T	dbsnp.101:rs2926747	MISSENSE	5664	1795	S	T	het	het	het	Y
NACA	chr12	57114099	57114100	A	G	dbsnp.101:rs2926743	MISSENSE	1495	405	F	S	hom	hom	hom	Y
NACA	chr12	57114306	57114307	A	T	dbsnp.101:rs2958127	MISSENSE	1288	336	V	E	het	het	not called	Y
NAV3	chr12	78225373	78225374	A	G	dbsnp.120:rs10735309	MISSENSE	305	45	T	A	hom	hom	hom	Y
NAV3	chr12	78511987	78511988	C	A		NONSENSE		984	S	*	not called	not called	not called	Y
NBN	chr8	90990478	90990479	C	G	dbsnp.92:rs1805794	MISSENSE	662	185	E	Q	hom	hom	hom	Y
NCAPD3	chr11	134062763	134062764	C	T	dbsnp.120:rs12292394	MISSENSE	2470	622	R	Q	hom	hom	hom	Y
NCOA4	chr10	51568377	51568378	T	G	COSMIC:mut:146935;dbsnp.120:rs10761581	MISSENSE	273	8	F	V	hom	hom	hom	N
NEK1	chr4	170482696	170482698	TC	AT		MISSENSE	1690	400	R	N	not called	het	not called	N
NEK11	chr3	130947434	130947435	A	T	dbsnp.107:rs3738000	MISSENSE	1715	488	E	V	hom	hom	hom	Y
NEK9	chr14	75574086	75574087	C	T	dbsnp.119:rs10146482	MISSENSE	1439	429	R	H	hom	hom	hom	Y
NFATC1	chr18	77246405	77246406	T	G	dbsnp.86:rs754093	MISSENSE	2703	751	C	G	hom	not called	hom	Y
NFKB1	chr4	103500048	103500049	G	C		MISSENSE	1046	194	E	Q	het	het	het	Y
NFKB2	chr10	104162022	104162023	G	A		MISSENSE	2790	864	D	N	het	het	het	Y
NGF	chr1	115829312	115829313	G	A	dbsnp.52:rs6330	MISSENSE	272	35	A	V	hom	hom	hom	Y

NGFR	chr17	47587818	47587819	C	T	dbsnp.96:rs2072446	MISSENSE	738	205	S	L	het	het	het	Y
NIN	chr14	51223788	51223789	C	T	dbsnp.96:rs2073347	MISSENSE	4149	1320	E	G	het	het	het	N
NIN	chr14	51224100	51224101	C	T	dbsnp.135:rs192017781	MISSENSE	3837	1216	R	Q	het	het	het	Y
NIN	chr14	51224373	51224374	T	G	dbsnp.121:rs12882191	MISSENSE	3564	1125	P	Q	het	het	het	N
NIPBL	chr5	37006525	37006526	C	T	COSMIC:mut:449594;COSMIC:mut:449595	MISSENSE	4421	1308	A	V	het	not called	not called	Y
NLRP1	chr17	5425076	5425077	T	C	dbsnp.120:rs11651270	MISSENSE	4116	1188	M	V	hom	hom	hom	Y
NLRP1	chr17	5436262	5436263	C	T	dbsnp.100:rs2301582	MISSENSE	3741	1063	V	M	hom	hom	hom	Y
NLRP10	chr11	7981750	7981751	G	A		NONSENSE	1424	470	Q	*	not called	not called	het	N
NOS2	chr17	26094787	26094788	A	T		MISSENSE	2373	704	W	R	not called	het	not called	N
NOS2	chr17	26094804	26094805	T	G		MISSENSE	2356	698	Y	S	not called	het	not called	N
NOS2	chr17	26096596	26096597	G	A	dbsnp.100:rs2297518	MISSENSE	2086	608	S	L	het	not called	not called	Y
NOS3	chr7	150696110	150696111	T	G	dbsnp.89:rs1799983	MISSENSE	1189	298	D	E	het	not called	not called	Y
NOTCH2	chr1	120539741	120539742	G	A	dbsnp.100:rs2258139;dbsnp.130:rs71246344	MISSENSE	884	210	P	L	het	het	het	N
NOTCH2	chr1	120572546	120572547	T	C	dbsnp.129:rs61788900;dbsnp.134:rs141882865	MISSENSE	392	46	N	S	not called	not called	het	N
NOTCH2	chr1	120572611	120572612	T	C	dbsnp.108:rs3872062	DISRUPT	328	25		TGTTTTTATTTTTGG	not called	not called	het	N
NOTCH2	chr1	120611959	120611960	C	T	dbsnp.100:rs2603926;dbsnp.134:rs139076095	MISSENSE	316	21	A	T	not called	not called	het	N
NOTCH2	chr1	120611963	120611964	G	C	COSMIC:mut:132738;dbsnp.120:rs11810554	MISSENSE	312	19	C	W	het	het	het	N
NOTCH2	chr1	120612002	120612004	GG		COSMIC:mut:132830	FRAMESHIFT	272	6	PA	RS	not called	het	het	N
NOTCH3	chr19	15271770	15271771	G	A	dbsnp.86:rs1044009	MISSENSE	6743	2223	A	V	hom	hom	hom	N
NOTCH4	chr6	32188822	32188823	G	A	dbsnp.117:rs8192585	MISSENSE	869	244	S	L	het	het	not called	Y
NOV	chr8	120431505	120431506	G	A	dbsnp.120:rs11538929	MISSENSE	918	233	R	H	het	het	het	Y
NPAT	chr11	108044090	108044091	T	A	dbsnp.110:rs4144901	MISSENSE	1721	540	L	F	hom	hom	hom	Y
NR1H2	chr19	50881820	50881820		AAC	rs66783852;rs112359270	IN_FRAME_INS		176177		Q	not called	not called	not called	Y
NR4A3	chr9	102591041	102591042	A	G	dbsnp.120:rs12344570	MISSENSE	1446	240	S	G	het	het	not called	N
NSD1	chr5	176684146	176684147	C	G		MISSENSE	5098	1654	S	C	het	het	het	Y
NSD1	chr5	176715854	176715855	C	G		MISSENSE	6324	2063	L	V	het	het	het	Y
NUAK1	chr12	106460937	106460938	G	C	COSMIC:mut:147600;COSMIC:mut:147601;dbsnp.107:rs3741883	MISSENSE	3007	543	P	R	het	het	not called	Y
NUMA1	chr11	71724113	71724114	C	G	dbsnp.132:rs111576201	MISSENSE	4606	1479	E	Q	hom	hom	hom	Y
NUP153	chr6	17633060	17633061	C	T	dbsnp.100:rs2274136	MISSENSE	2678	827	A	T	hom	hom	hom	Y
NUP153	chr6	17665478	17665479	G	C	dbsnp.116:rs6906499	MISSENSE	1405	402	N	K	hom	hom	hom	Y
NUP153	chr6	17675245	17675246	T	C	dbsnp.98:rs2228375	MISSENSE	941	248	I	V	hom	hom	hom	Y
NUP210	chr3	13395578	13395579	C	A	dbsnp.100:rs2280084	MISSENSE	2439	786	R	L	hom	hom	hom	Y
NUP210	chr3	13399785	13399786	G	A	dbsnp.116:rs6795271	MISSENSE	2346	755	A	V	hom	hom	hom	Y
NUP210	chr3	13407555	13407556	T	C	dbsnp.107:rs3732671	MISSENSE	1904	608	I	V	hom	hom	hom	Y
NUP210	chr3	13421149	13421150	C	T	dbsnp.116:rs7628051	MISSENSE	971	297	A	T	hom	hom	hom	Y
NUP214	chr9	134020091	134020092	C	T	dbsnp.79:rs103612	MISSENSE	1830	574	P	S	het	het	het	Y
NYNRIN	chr14	24883886	24883887	G	A	rs8017377	MISSENSE		978	A	T	not called	not called	not called	Y
OBSCN	chr1	228402120	228402121	A	G	dbsnp.89:rs1771487	MISSENSE	1548	502	Q	R	het	het	het	N
OBSCN	chr1	228412226	228412228	TG	CA	dbsnp.89:rs1771480;dbsnp.89:rs1757153	MISSENSE	2764	907	SA	ST	het	het	het	N
OBSCN	chr1	228412227	228412228	TG	CA	rs1771480;rs1757153	MISSENSE		908	A	T	not called	not called	not called	Y
OBSCN	chr1	228444564	228444565	T	A	dbsnp.116:rs7532342	MISSENSE	4566	1508	V	D	hom	hom	hom	Y
OBSCN	chr1	228461128	228461129	A	G	dbsnp.87:rs1188724	MISSENSE	6216	2048	H	R	het	not called	not called	N
OBSCN	chr1	228464247	228464248	T	G	dbsnp.87:rs1188721	MISSENSE	6361	2106	D	E	het	not called	het	N
OBSCN	chr1	228464275	228464276	T	C	dbsnp.87:rs1188722	MISSENSE	6389	2116	F	L	het	not called	het	N
OBSCN	chr1	228465345	228465346	A	G	dbsnp.83:rs493945	MISSENSE	8093	2674	N	D	het	not called	not called	N
OBSCN	chr1	228468457	228468458	G	A	dbsnp.87:rs1188697	MISSENSE	8201	2720	V	M	het	not called	het	Y
OBSCN	chr1	228475847	228475848	G	A	dbsnp.80:rs437129	MISSENSE	9941	3300	A	T	het	het	het	Y
OBSCN	chr1	228494103	228494104	G	C		MISSENSE	11764	3897	Q	H	het	not called	not called	Y
OBSCN	chr1	228494789	228494790	G	A	dbsnp.80:rs435776	MISSENSE	12158	4039	G	R	hom	hom	hom	Y
OBSCN	chr1	228503676	228503677	A	G	dbsnp.87:rs1150912	MISSENSE	13185	4381	H	R	hom	not called	hom	Y
OBSCN	chr1	228504471	228504472	T	C	dbsnp.87:rs1188732	MISSENSE	13391	4450	C	R	hom	hom	hom	N
OBSCN	chr1	228504669	228504670	C	T	dbsnp.120:rs11810627	MISSENSE	13589	4516	R	W	hom	hom	hom	N
OBSCN	chr1	228505667	228505668	C	G	dbsnp.87:rs1188729	MISSENSE	13968	4642	S	C	hom	hom	hom	Y
OBSCN	chr1	228509426	228509427	A	G	dbsnp.80:rs373610	MISSENSE	14928	4962	D	G	hom	hom	half called	Y
OBSCN	chr1	228520972	228520973	C	G	dbsnp.80:rs369909	MISSENSE	15848	5269	L	V	hom	hom	hom	Y
OBSCN	chr1	228528562	228528563	C	G	dbsnp.87:rs1188710	MISSENSE	17714	5891	Q	E	hom	not called	hom	Y
P2RX7	chr12	121600252	121600253	T	C	dbsnp.79:rs208294	MISSENSE	605	155	Y	H	het	het	het	Y
P2RX7	chr12	121615102	121615103	G	A	dbsnp.89:rs1718119	MISSENSE	1184	348	A	T	het	het	het	Y
P2RX7	chr12	121622303	121622304	A	C	dbsnp.107:rs3751143	MISSENSE	1629	496	E	A	het	het	het	Y
P2RX7	chr12	121622519	121622520	A	T	rs1653624	MISSENSE		568	N	I	not called	not called	not called	Y
PAFAH1B2	chr11	117042376	117042377	G	A	dbsnp.111:rs4936367	MISSENSE	592	151	V	M	hom	hom	hom	N

PAK2	chr3	196509576	196509577	C	G	rs76714248	MISSENSE		20	S	R	not called	not called	not called	Y
PAK4	chr19	39668400	39668401	G	C	dbsnp.132:rs111768373	MISSENSE	2032	524	M	I	het	het	not called	Y
PAK7	chr20	9543621	9543622	C	T	dbsnp.100:rs2297345	MISSENSE	2076	511	S	N	hom	hom	not called	Y
PAK7	chr20	9547017	9547018	C	G	dbsnp.120:rs11700112	MISSENSE	1548	335	R	P	hom	hom	hom	Y
PAPPA	chr9	119106880	119106881	C	A	dbsnp.116:rs7020782	MISSENSE	4051	1224	S	Y	hom	hom	hom	Y
PARP1	chr1	226595554	226595555	C	G		MISSENSE	246	26	E	Q	het	het	het	Y
PARP9	chr3	122259605	122259606	T	C	dbsnp.119:rs9851180	MISSENSE	1727	528	Y	C	het	het	het	Y
PASK	chr2	242077495	242077496	C	T	COSMIC:mut:149119;COSMIC:mut:149120;dbsnp.88:rs1470414	MISSENSE	880	250	V	I	het	not called	not called	N
PASK	chr2	242082261	242082262	T	C	dbsnp.116:rs6709462	MISSENSE	317	62	T	M	het	not called	not called	N
PASK	chr2	242082261	242082263	TG	CA	dbsnp.116:rs6709462;dbsnp.134:rs144572631	MISSENSE	276	62	T	M	het	hom	hom	Y
PAX3	chr2	223085954	223085955	G	T	dbsnp.98:rs2234675	MISSENSE	1321	314	T	K	het	het	het	Y
PBX1	chr1	164529119	164529120	G	A	dbsnp.100:rs2275558	MISSENSE	318	21	G	S	het	het	het	N
PCDH15	chr10	55566405	55566406	T	G	dbsnp.123:rs17704703	MISSENSE	5376	1661	Q	P	het	het	not called	N
PCDH15	chr10	55583014	55583015	C	T		MISSENSE	4886	1498	E	K	het	het	het	Y
PCDH15	chr10	55755490	55755491	C	T	dbsnp.96:rs2135720	MISSENSE	3195	934	R	Q	het	het	het	Y
PCDH15	chr10	55955443	55955444	T	G	dbsnp.111:rs4935502	MISSENSE	1713	440	D	A	not called	not called	het	Y
PCM1	chr8	17814914	17814915	A	G	dbsnp.79:rs208753	MISSENSE	2210	597	M	V	hom	not called	hom	Y
PDE4DIP	chr1	144854597	144854598	TC	CT	rs3851873;rs3863691	MISSENSE		2291	R	Q	not called	not called	not called	Y
PDE4DIP	chr1	144865849	144865850	G	T	dbsnp.89:rs1613780	MISSENSE	6020	1910	D	E	het	het	het	Y
PDE4DIP	chr1	144866642	144866643	G	A	dbsnp.89:rs1620560	MISSENSE	5889	1867	R	C	het	het	het	Y
PDE4DIP	chr1	144868169	144868170	C	T	dbsnp.89:rs1628310	MISSENSE	5559	1757	A	T	het	het	het	Y
PDE4DIP	chr1	144871737	144871738	C	A	rs1698605	MISSENSE		1742	A	S	not called	not called	not called	Y
PDE4DIP	chr1	144871754	144871755	A	T	dbsnp.89:rs1778159	MISSENSE	5497	1736	V	E	not called	half called	not called	Y
PDE4DIP	chr1	144871781	144871782	A	G	dbsnp.89:rs1778158	MISSENSE	5470	1727	L	P	not called	not called	het	N
PDE4DIP	chr1	144871782	144871782		GGG		INSERT	5470	1727		P	not called	het	not called	N
PDE4DIP	chr1	144873962	144873963	T		dbsnp.120:rs11341221	FRAMESHIFT	5284	1665	Q	R	het	het	het	Y
PDE4DIP	chr1	144877175	144877176	C	T	dbsnp.100:rs2762875	MISSENSE	4801	1504	R	Q	het	het	het	Y
PDE4DIP	chr1	144879263	144879264	A	G	dbsnp.100:rs2798901	MISSENSE	4476	1396	W	R	het	het	het	Y
PDE4DIP	chr1	144879374	144879375	T	C	dbsnp.89:rs1747958	MISSENSE	4365	1359	K	E	half called	half called	not called	Y
PDE4DIP	chr1	144882822	144882823	C	T	dbsnp.89:rs1698647	MISSENSE	3486	1066	A	T	het	het	het	Y
PDE4DIP	chr1	144886196	144886197	A	T	dbsnp.89:rs1698624	MISSENSE	3327	1013	F	I	het	het	het	Y
PDE4DIP	chr1	144906120	144906121	G	A		MISSENSE	3324	1001	R	W	not called	het	not called	N
PDE4DIP	chr1	144912152	144912153	A	G	dbsnp.89:rs1628172	MISSENSE	2934	871	C	R	het	het	het	Y
PDE4DIP	chr1	144912232	144912233	C	T	dbsnp.89:rs1629011	MISSENSE	2854	844	R	H	het	het	het	Y
PDE4DIP	chr1	144915560	144915561	G	A	dbsnp.89:rs1778111	NONSENSE	2676	785	R	*	half called	het	half called	Y
PDE4DIP	chr1	144916675	144916676	C	T	dbsnp.89:rs1698683	NONSENSE	2491	723	W	*	het	het	het	Y
PDE4DIP	chr1	144916747	144916748	C	G	dbsnp.89:rs1747930	MISSENSE	2419	699	S	T	het	het	het	Y
PDE4DIP	chr1	144917827	144917828	A		dbsnp.120:rs11295415	FRAMESHIFT	2270	649	VA	VL	not called	half called	half called	Y
PDE4DIP	chr1	144917840	144917841	T	C	dbsnp.89:rs1698681;dbsnp.131:rs79453142	MISSENSE	2257	645	H	R	not called	half called	not called	Y
PDE4DIP	chr1	144918956	144918957	T	A	dbsnp.86:rs1061308	MISSENSE	2041	573	E	V	not called	het	het	Y
PDE4DIP	chr1	144921917	144921918	C	T		MISSENSE	1923	534	D	N	het	not called	het	Y
PDE4DIP	chr1	144922522	144922523	C	T	dbsnp.100:rs2455994	MISSENSE	1696	458	R	H	het	het	het	Y
PDE4DIP	chr1	144922582	144922583	G	A	dbsnp.88:rs1359300	MISSENSE	1636	438	S	L	het	het	het	Y
PDE4DIP	chr1	144930939	144930940	T	C	dbsnp.96:rs2147326	MISSENSE	1092	257	K	E	het	het	het	N
PDE4DIP	chr1	144931329	144931330	C	T	dbsnp.100:rs2762745	MISSENSE	702	127	A	T	het	het	het	N
PDE4DIP	chr1	144931391	144931392	G	A	dbsnp.100:rs2798893	MISSENSE	640	106	A	V	het	het	het	N
PDE4DIP	chr1	144952206	144952207	C	T	rs3121544	MISSENSE		171	R	K	not called	not called	not called	Y
PDE4DIP	chr1	144952219	144952220	C	T		MISSENSE		167	A	T	not called	not called	not called	Y
PDE4DIP	chr1	144994657	144994658	C	A	dbsnp.89:rs1664022	MISSENSE	364	25	R	L	het	het	het	Y
PDE4DIP	chr1	145015876	145015877	G	T	rs77741369	MISSENSE		71	L	I	not called	not called	not called	Y
PDE4DIP	chr1	145039593	145039594	T	C	COSMIC:mut:1333549;dbsnp.132:rs111958438	MISSENSE	398	6	T	A	het	not called	not called	N
PDE4DIP	chr1	145075682	145075683	C	T	dbsnp.100:rs2762779	NONSENSE	396	60	W	*	het	not called	het	Y
PDGFA	chr7	540740	540741	G	A	rs1800814	MISSENSE		212	P	S	not called	not called	not called	Y
PDZRN4	chr12	41582602	41582603	G	C	dbsnp.120:rs10879831	MISSENSE	353	116	G	R	het	not called	not called	N
PER1	chr17	8046771	8046772	C	G	dbsnp.100:rs2585405	MISSENSE	3121	962	A	P	hom	half called	hom	Y
PER1	chr17	8046988	8046989	G	C		MISSENSE	2904	889	F	L	het	not called	not called	Y
PEX5L	chr3	179593235	179593236	C	T		MISSENSE		179	D	N	not called	not called	not called	Y
PGAP3	chr17	37842242	37842243	C	T		MISSENSE		71	E	K	not called	not called	not called	Y
PGR	chr11	100933411	100933412	C	A	dbsnp.86:rs1042838	MISSENSE	2720	660	V	L	het	het	het	Y
PGR	chr11	100998770	100998771	C	G	dbsnp.107:rs3740753	MISSENSE	1773	344	S	T	het	het	het	Y
PHIP	chr6	79675700	79675701	A	G	dbsnp.119:rs9350797	MISSENSE	3503	1093	L	P	hom	hom	hom	Y

PIK3C2A	chr11	17118686	17118687	T	C	dbsnp.120:rs11604561	MISSENSE	4308	1415	T	A	het	het	het	Y
PIK3CA	chr3	178916945	178916946	G	C	COSMIC:mut:12580	MISSENSE	489	111	K	N	het	het	het	Y
PIK3CG	chr7	106509330	106509331	C	A	dbsnp.123:rs17847825	MISSENSE	1634	442	S	Y	het	het	het	Y
PIKFYVE	chr2	209179938	209179939	A	G	dbsnp.123:rs16840913	MISSENSE	2006	617	M	V	het	hom	hom	Y
PIKFYVE	chr2	209184979	209184980	G	A	dbsnp.120:rs10932258	MISSENSE	2244	696	S	N	hom	hom	hom	Y
PIKFYVE	chr2	209190329	209190330	T	C	dbsnp.100:rs2363468	MISSENSE	2952	932	L	S	hom	hom	hom	Y
PIKFYVE	chr2	209190518	209190519	A	T	dbsnp.86:rs893254	MISSENSE	3141	995	Q	L	hom	hom	hom	Y
PIKFYVE	chr2	209190527	209190528	C	G	dbsnp.86:rs893253	MISSENSE	3150	998	T	S	hom	hom	hom	Y
PIKFYVE	chr2	209190631	209190632	T	G	dbsnp.86:rs999890	MISSENSE	3254	1033	S	A	het	hom	het	Y
PIKFYVE	chr2	209191081	209191082	C	A	dbsnp.88:rs1529979	MISSENSE	3704	1183	Q	K	hom	hom	hom	Y
PIM1	chr6	37138219	37138220	C	G	dbsnp.79:rs262936	MISSENSE	298	48	H	D	het	not called	not called	N
PIM3	chr22	50356692	50356693	T	C	dbsnp.108:rs4077129	MISSENSE	1351	300	V	A	hom	half called	not called	Y
PINK1	chr1	20976999	20977000	A	C	dbsnp.86:rs1043424	MISSENSE	1655	521	N	T	het	het	het	Y
PIP4K2A	chr10	22839627	22839628	T	C	dbsnp.98:rs2230469;dbsnp.120:rs10828317	MISSENSE	999	251	N	S	het	het	het	Y
PKHD1	chr6	51875249	51875250	A	C	dbsnp.100:rs2435322	MISSENSE	5883	1870	L	V	hom	hom	hom	Y
PKHD1	chr6	51890822	51890823	G	A	dbsnp.119:rs9296669	MISSENSE	4060	1262	A	V	het	het	not called	Y
PKHD1	chr6	51910904	51910905	T	C	COSMIC:mut:150104;dbsnp.129:rs62406032	MISSENSE	2764	830	N	S	het	het	hom	Y
PKHD1	chr6	51914955	51914956	G	A	dbsnp.119:rs9370096	MISSENSE	2553	760	R	C	het	het	not called	N
PKHD1	chr6	51923290	51923291	C	G	dbsnp.134:rs149781976	MISSENSE	1617	448	G	R	not called	het	not called	Y
PKMYT1	chr16	3023024	3023025	G	A	dbsnp.132:rs113683372	MISSENSE	1871	477	R	W	het	not called	het	Y
PKN1	chr19	14568934	14568935	A	T		MISSENSE	1425	420	T	S	het	not called	not called	Y
PKN1	chr19	14580327	14580328	A	G	dbsnp.98:rs2230539	MISSENSE	2319	718	I	V	het	het	het	Y
PLAG1	chr8	57080788	57080789	C	G		MISSENSE	435	14	D	H	het	not called	not called	Y
PLD1	chr3	171404477	171404478	C	A	COSMIC:mut:1162698;dbsnp.100:rs2290480	MISSENSE	1990	622	A	S	het	not called	not called	Y
PLD1	chr3	171417548	171417549	G	A		MISSENSE	1328	405	L	F	het	not called	het	Y
PLEKHA5	chr12	19500062	19500063	G	A		MISSENSE	2952	949	E	K	het	not called	not called	N
PLK4	chr4	128807218	128807219	T	A	dbsnp.107:rs3811740	MISSENSE	871	200	S	T	het	het	not called	Y
PLK4	chr4	128814963	128814964	G	T	dbsnp.123:rs17012739	MISSENSE	2667	798	E	D	het	het	het	Y
PML	chr15	74328115	74328116	A	G	dbsnp.86:rs743580	MISSENSE	2453	772	S	G	hom	hom	hom	N
PML	chr15	74328140	74328141	G	T	dbsnp.86:rs743581	MISSENSE	2478	780	G	V	het	het	het	N
PML	chr15	74337305	74337306	T	G		MISSENSE	2745	869	L	R	not called	not called	het	Y
PMS1	chr2	190682790	190682791	A	G		MISSENSE		156	K	R	not called	not called	not called	Y
PMS2	chr7	6026864	6026865	T	C	dbsnp.98:rs2228007	MISSENSE	1617	511	T	A	not called	not called	het	Y
PMS2	chr7	6045626	6045627	C	T	dbsnp.119:rs10254120	MISSENSE	145	20	R	Q	not called	het	not called	Y
POU5F1B	chr8	128428637	128428638	G	A	dbsnp.116:rs6998061;dbsnp.131:rs75641460	MISSENSE	781	176	G	E	het	het	het	Y
POU5F1B	chr8	128428822	128428823	G	C	dbsnp.116:rs7002225;dbsnp.129:rs60346131	MISSENSE	966	238	E	Q	half called	half called	not called	Y
PPARGC1A	chr4	23815661	23815662	C	T	dbsnp.117:rs8192678	MISSENSE	1563	482	G	S	hom	hom	hom	Y
PPM1E	chr17	56833457	56833457		GAACCC	rs74256772	IN_FRAME_INS		4344		P	not called	not called	not called	Y
PPP1R3A	chr7	113518501	113518502	A	T	dbsnp.101:rs2974938	MISSENSE	2675	882	L	H	hom	hom	hom	Y
PPP1R3A	chr7	113519718	113519719	A	T	dbsnp.101:rs2974944	MISSENSE	1458	476	N	K	hom	hom	hom	Y
PPP1R3A	chr7	113519795	113519796	C	T	dbsnp.101:rs2974942	MISSENSE	1381	451	V	M	hom	hom	hom	Y
PRAME	chr22	22892448	22892449	T	C	dbsnp.127:rs41277507	MISSENSE	900	218	M	V	hom	hom	hom	Y
PRAME	chr22	22899233	22899234	A	G	COSMIC:mut:85675;dbsnp.86:rs1129172	MISSENSE	267	7	W	R	hom	not called	not called	Y
PRDM16	chr1	3328357	3328358	T	C	dbsnp.86:rs870124	MISSENSE	1678	533	S	P	het	not called	half called	Y
PRKACG	chr9	71628206	71628207	G	C	dbsnp.107:rs3730386	MISSENSE	832	268	H	D	het	not called	het	Y
PRKCQ	chr10	6527142	6527143	G	A	COSMIC:mut:146874;dbsnp.98:rs2236379	MISSENSE	1063	330	P	L	het	het	het	Y
PRKCZ	chr1	2066741	2066742	T	G	rs75260030	MISSENSE		126	Y	D	not called	not called	not called	Y
PRKD2	chr19	47177912	47177913	A	G	rs314665	MISSENSE		845	V	A	not called	not called	not called	Y
PRKDC	chr8	48805816	48805816		G	rs67588121	FRAME_SHIFT_INS		1244	L		not called	not called	not called	Y
PRKX	chrX	3631166	3631167	A	G	dbsnp.107:rs3752362	MISSENSE	508	43	V	A	het	half called	het	N
PRRX1	chr1	170633501	170633504	TGG			DELETE	189	49	V		not called	het	not called	N
PSKH1	chr16	67942751	67942752	G	A		MISSENSE		34	V	M	not called	not called	not called	Y
PSKH2	chr8	87076651	87076652	G	C		MISSENSE	393	132	Q	E	het	het	het	Y
PSKH2	chr8	87076809	87076810	C	T	dbsnp.126:rs35315725	MISSENSE	235	79	R	K	het	het	het	Y
PTCH1	chr9	98209593	98209594	G	A	dbsnp.79:rs357564	MISSENSE	4131	1315	P	L	hom	hom	hom	N
PTK2B	chr8	27290970	27290971	C	T		MISSENSE	1654	336	S	L	not called	hom	het	N
PTPRT	chr20	41818288	41818289	C	G	dbsnp.101:rs2867655	MISSENSE	268	29	A	P	not called	hom	half called	N
PTPRU	chr1	29631908	29631909	A	G	COSMIC:mut:146456;dbsnp.98:rs2235937	MISSENSE	2917	930	N	S	hom	hom	hom	Y

RAD21	chr8	117864885	117864886	C	A		MISSENSE	1510	408	G	V	het	het	not called	Y
RAG1	chr11	36595599	36595600	A	G	dbsnp.107:rs3740955	MISSENSE	869	249	R	H	het	not called	not called	N
RAP1GDS1	chr4	99273751	99273752	C	T		MISSENSE	553	122	H	Y	het	het	not called	Y
RARG	chr12	53605544	53605545	G	A	dbsnp.98:rs2229774	MISSENSE	1764	427	S	L	het	het	het	Y
RBBP8	chr18	20573317	20573318	G	C		MISSENSE	1858	510	E	Q	het	het	het	Y
RBL1	chr20	35695460	35695461	T	A		MISSENSE	698	207	D	V	not called	het	het	N
RBL2	chr16	53481009	53481010	A	G	dbsnp.123:rs17800727	MISSENSE	746	210	Y	C	het	het	het	Y
RBL2	chr16	53488748	53488749	G	C		MISSENSE	1291	392	D	H	het	het	het	Y
RBM15	chr1	110888947	110888948	G	A	dbsnp.129:rs61787373	MISSENSE	2964	961	R	Q	het	not called	not called	N
RECQL4	chr8	145737372	145737373	C	T	dbsnp.126:rs36078464	MISSENSE	3355	1105	G	D	het	not called	not called	Y
RECQL4	chr8	145737815	145737816	C	T	COSMIC:mut:1569133;dbsnp.111:rs4251691	MISSENSE	3055	1005	R	Q	het	not called	not called	Y
RECQL4	chr8	145737863	145737864	A	C		MISSENSE	3007	989	M	R	het	not called	not called	Y
RECQL4	chr8	145738767	145738768	G			FRAME_SHIFT_DEL		766	R		not called	not called	not called	Y
RECQL4	chr8	145741701	145741702	C	G	dbsnp.111:rs4244612	MISSENSE	842	267	E	D	het	half called	not called	Y
RECQL4	chr8	145742513	145742514	A	G	rs2721190	MISSENSE		92	S	P	not called	not called	not called	Y
REM1	chr20	30064330	30064331	A	G	dbsnp.86:rs1006459	MISSENSE	366	28	H	R	hom	hom	half called	Y
RGL2	chr6	33264453	33264454	C	T		MISSENSE	702	32	V	M	het	not called	not called	Y
RGL3	chr19	11505115	11505116	C	G	dbsnp.132:rs113801365	MISSENSE	2158	699	E	Q	het	not called	het	N
RGL3	chr19	11526758	11526759	G	A	dbsnp.79:rs160838	MISSENSE	554	164	A	V	het	het	half called	Y
RGL3	chr19	11526764	11526765	G	T	dbsnp.79:rs167479	MISSENSE	548	162	P	H	hom	hom	half called	Y
RGL4	chr22	24034287	24034288	A	G	dbsnp.86:rs738786	MISSENSE	1240	24	Q	R	hom	hom	hom	Y
RGL4	chr22	24035969	24035970	C	T	dbsnp.96:rs2070446	MISSENSE	1890	241	H	Y	hom	hom	hom	Y
RGL4	chr22	24038846	24038847	T	C	dbsnp.86:rs1007298	MISSENSE	2302	378	V	A	hom	hom	hom	N
RHOA	chr3	49412972	49412973	C	T	dbsnp.120:rs11552761	MISSENSE	325	17	G	E	het	het	het	Y
RHPN2	chr19	33490584	33490585	G	A	rs78615454	NONSENSE		378	Q	*	not called	not called	not called	Y
RHPN2	chr19	33493766	33493767	G	C		MISSENSE		300	I	M	not called	not called	not called	Y
RHPN2	chr19	33512484	33512485	C	T		MISSENSE	447	128	V	I	het	het	not called	N
RHPN2	chr19	33512490	33512491	C	A		MISSENSE	441	126	A	S	het	het	not called	N
RHPN2	chr19	33512499	33512500	C	T		MISSENSE	432	123	V	I	het	het	not called	N
RHPN2	chr19	33512538	33512539	T	C		MISSENSE	393	110	I	V	het	het	het	N
RICTOR	chr5	38955795	38955796	G	A	dbsnp.94:rs2043112	MISSENSE	2533	837	S	F	het	het	het	Y
RIF1	chr2	152311569	152311570	G	A	dbsnp.100:rs2444263	MISSENSE	2666	836	G	S	het	het	hom	Y
RIF1	chr2	152320117	152320118	G	A	dbsnp.96:rs2123465	MISSENSE	4244	1362	V	M	het	het	hom	Y
RIF1	chr2	152322094	152322095	A	T	dbsnp.100:rs2444257	MISSENSE	6221	2021	N	Y	het	het	hom	Y
RIF1	chr2	152331417	152331418	C	G	dbsnp.86:rs1065177	MISSENSE	7334	2392	L	V	het	het	hom	Y
RIMS2	chr8	104778751	104778752	A	G	dbsnp.135:rs188770151	MISSENSE	823	229	I	V	het	het	het	Y
RIOK1	chr6	7405507	7405508	G	A	dbsnp.129:rs56067778	MISSENSE	1296	375	V	I	het	hom	het	Y
RIOK2	chr5	96503417	96503418	C	G		MISSENSE	1259	384	E	Q	het	hom	half called	Y
RIOK2	chr5	96503522	96503523	C	T	dbsnp.79:rs160632	MISSENSE	1154	349	G	R	hom	hom	hom	Y
RIOK2	chr5	96513470	96513471	G	C	dbsnp.100:rs2544773	MISSENSE	396	96	S	C	hom	hom	hom	Y
RIPK4	chr21	43161356	43161357	T	C	dbsnp.107:rs3746891	MISSENSE	2043	666	M	V	hom	hom	hom	Y
RNASEL	chr1	182551336	182551337	A	C	dbsnp.83:rs627928	MISSENSE	1789	541	D	E	hom	hom	hom	Y
RNASEL	chr1	182554556	182554557	C	T	dbsnp.83:rs486907	MISSENSE	1551	462	R	Q	hom	hom	hom	Y
ROCK1	chr18	18533611	18533612	G	A	dbsnp.86:rs1045144	NONSENSE	4928	1330	R	*	not called	not called	het	N
ROR2	chr9	94486320	94486321	C	T	dbsnp.120:rs10761129	MISSENSE	2653	819	V	I	hom	hom	hom	Y
ROR2	chr9	94495607	94495608	T	C	dbsnp.120:rs10820900	MISSENSE	931	245	T	A	hom	hom	hom	N
ROS1	chr6	117642452	117642453	C	A		MISSENSE	5944	1916	V	L	het	het	het	Y
RPGRIP1	chr14	21770729	21770730	A	G	dbsnp.116:rs6571751	MISSENSE	573	192	K	E	het	not called	not called	Y
RPGRIP1	chr14	21796783	21796784	G	C	COSMIC:mut:147738;dbsnp.107:rs3748361	MISSENSE	3096	1033	E	Q	het	not called	het	Y
RPS6KA2	chr6	167271710	167271711	T	C	dbsnp.86:rs943687	MISSENSE	212	34	T	A	hom	hom	hom	N
RPS6KA2	chr6	167271715	167271716	T	C	dbsnp.119:rs9347162	MISSENSE	207	32	E	G	het	het	het	Y
RPS6KA3	chrX	20181136	20181137	C	T		MISSENSE	1785	596	D	N	het	not called	het	Y
RPS6KA5	chr14	91366459	91366460	G	C		MISSENSE	1585	457	I	M	het	not called	not called	Y
RPS6KA6	chrX	83320016	83320017	C	T	dbsnp.116:rs6616890	MISSENSE	2109	692	D	N	hom	half called	hom	Y
RPS6KB2	chr11	67198886	67198887	C	T	dbsnp.132:rs113335412	MISSENSE	439	120	R	W	het	het	het	Y
RPS6KB2	chr11	67202155	67202156	C	T	dbsnp.52:rs13859	MISSENSE	1340	420	A	V	hom	hom	hom	Y
RPS6KL1	chr14	75386575	75386576	G	A	dbsnp.100:rs2286913	MISSENSE	846	121	P	L	hom	hom	hom	Y
RPS6KL1	chr14	75388182	75388183	C	T	dbsnp.116:rs7156590	MISSENSE	546	21	R	Q	hom	hom	hom	Y

RRM2	chr2	10262919	10262920	T	G	COSMIC:mut:440905;dbsnp.86:rs1130609	MISSENSE	225	59	S	A	het	not called	not called	N
RUNX1T1	chr8	93026880	93026881	T	C		MISSENSE	723	105	T	A	het	not called	het	Y
RYK	chr3	133941319	133941320	C	T	COSMIC:mut:149476;dbsnp.86:rs1131262	MISSENSE	385	99	S	N	het	het	het	N
SACS	chr13	23928670	23928671	C	T	dbsnp.123:rs17325713	MISSENSE	2668	694	A	T	hom	hom	half called	Y
SBDS	chr7	66453469	66453470	G	A		MISSENSE	824	214	P	L	not called	het	not called	N
SBK2	chr19	56041254	56041255	C	G	dbsnp.83:rs620251	MISSENSE	906	298	A	P	hom	half called	hom	N
SBK2	chr19	56047447	56047448	A	G	dbsnp.79:rs310453	MISSENSE	228	72	C	R	hom	not called	hom	N
SCD	chr10	102116310	102116311	A	C	dbsnp.98:rs2234970	MISSENSE	1159	224	M	L	hom	hom	hom	Y
SCD5	chr4	83582063	83582064	G	T	dbsnp.107:rs3733228	MISSENSE	1055	246	L	M	het	not called	het	N
SCD5	chr4	83582210	83582211	C	G	dbsnp.107:rs3733227	MISSENSE	908	197	E	Q	hom	hom	hom	N
SCRIB	chr8	144873838	144873839	G	C		MISSENSE	4773	1589	I	M	het	not called	not called	N
SCRIB	chr8	144893083	144893084	G	A	dbsnp.116:rs6558394	MISSENSE	1271	422	P	L	hom	not called	not called	N
SCYL2	chr12	100732684	100732688	ATCT	TTAA		MISSENSE	2574	842	NL	IN	not called	not called	het	N
SCYL3	chr1	169823717	169823718	T	C	COSMIC:mut:146679;dbsnp.111:rs4656197	MISSENSE	1897	567	Q	R	het	het	hom	Y
SEMA5B	chr3	122630345	122630346	T	C	dbsnp.100:rs2303983	MISSENSE	3386	1028	D	G	hom	hom	hom	N
SEMA5B	chr3	122631895	122631896	A	T	dbsnp.100:rs2276782	MISSENSE	2822	840	V	D	hom	half called	hom	N
SEMA5B	chr3	122647883	122647884	C	T	dbsnp.137:rs200299701	MISSENSE	799	166	E	K	het	not called	het	N
SEPT9	chr17	75398497	75398498	C	T	dbsnp.126:rs34587622	MISSENSE	559	145	P	L	hom	hom	hom	Y
SEPT9	chr17	75494704	75494705	A	G	dbsnp.100:rs2627223	MISSENSE	1851	576	M	V	hom	not called	not called	Y
SGK2	chr20	42196340	42196342	CG	GA		MISSENSE	521	101	IG	MS	not called	not called	het	N
SGK223	chr8	8176387	8176387		GGGGCG	dbsnp.134:rs143409664;dbsnp.130:rs71217287	INSERT	3497	1166		PA	half called	not called	not called	N
SGK223	chr8	8176553	8176554	C	T	dbsnp.120:rs12549973	MISSENSE	3330	1111	A	T	hom	not called	not called	N
SGK269	chr15	77407113	77407114	C	G	dbsnp.92:rs1867780	MISSENSE	4932	1542	S	T	not called	not called	het	N
SHC1	chr1	154942975	154942975		CTTGGG		INSERT	248	10		PK	not called	het	not called	N
SIK1	chr21	44837554	44837555	G	A	dbsnp.80:rs430554	MISSENSE	1970	615	A	V	hom	not called	hom	N
SIX4	chr14	61180656	61180657	T	G	dbsnp.107:rs3742636	MISSENSE	1873	605	H	P	het	not called	het	Y
SKIL	chr3	170078231	170078232	C	T	dbsnp.107:rs3772173	MISSENSE	821	38	A	V	hom	half called	hom	Y
SLC14A1	chr18	43310414	43310415	G	A	dbsnp.100:rs2298720	MISSENSE	519	100	E	K	het	het	het	Y
SLK	chr10	105763025	105763026	C	T	dbsnp.107:rs3740469	MISSENSE	2123	697	T	I	hom	hom	hom	Y
SMAD7	chr18	46468945	46468946	G	A	dbsnp.107:rs3764482	MISSENSE	231	28	S	F	het	het	het	N
SMG1	chr16	18840948	18840949	C	T		MISSENSE	9624	3088	D	N	not called	het	not called	N
SMG1	chr16	18879947	18879949	TT	CC		MISSENSE	3288	976	N	G	not called	het	not called	N
SNX19	chr11	130750641	130750642	A	C	dbsnp.100:rs2298566	MISSENSE	3180	878	L	R	hom	hom	hom	Y
SNX19	chr11	130776523	130776524	T	C	dbsnp.111:rs4414223	MISSENSE	2805	753	N	S	hom	hom	hom	Y
SNX19	chr11	130784753	130784754	C	G	dbsnp.107:rs3751037	MISSENSE	1628	361	V	L	hom	hom	hom	Y
SORL1	chr11	121458814	121458815	C	T		NONSENSE	4029	1301	Q	*	het	not called	not called	Y
SP110	chr2	231036859	231036860	C	T	dbsnp.108:rs3948463	MISSENSE	1976	579	M	I	hom	hom	hom	Y
SP110	chr2	231042275	231042276	A	G	dbsnp.86:rs1135791	MISSENSE	1843	529	M	T	hom	hom	hom	Y
SP110	chr2	231050714	231050715	A	G	dbsnp.108:rs3948464	MISSENSE	1549	431	L	S	hom	hom	hom	N
SP110	chr2	231072708	231072709	C	T	dbsnp.88:rs1365776	MISSENSE	1170	305	G	R	hom	hom	hom	Y
SP110	chr2	231077109	231077110	C	T	COSMIC:mut:149088;dbsnp.52:rs9061	MISSENSE	894	213	E	K	hom	hom	hom	Y
SP110	chr2	231077675	231077676	G	A	COSMIC:mut:149089;dbsnp.120:rs11556887	MISSENSE	658	134	A	V	hom	hom	hom	N
SP110	chr2	231077724	231077725	A	G	dbsnp.86:rs1129411	MISSENSE	609	118	W	R	hom	hom	hom	N
SP140	chr2	231149107	231149108	G	A	dbsnp.111:rs4972946	MISSENSE	1660	516	E	K	hom	hom	hom	Y
SPEG	chr2	220309684	220309685	G	A	dbsnp.129:rs55821435	MISSENSE	616	206	R	H	hom	hom	hom	N
SPEG	chr2	220348948	220348949	C	G		NONSENSE		2255	S	*	not called	not called	not called	Y
SPEG	chr2	220353531	220353532	C	A	dbsnp.121:rs13026308	MISSENSE	8058	2687	P	T	hom	hom	half called	N
SPP1	chr4	88898940	88898941	C	T	dbsnp.120:rs11728697	MISSENSE	385	24	A	V	het	not called	not called	N
SS18	chr18	23618521	23618522	C	T		MISSENSE	954	293	D	N	het	not called	not called	Y
SSX1	chrX	48123331	48123332	A	G		MISSENSE	536	149	E	G	het	het	het	Y
STAT3	chr17	40490776	40490777	G	C		MISSENSE	739	174	F	L	het	het	het	Y
STK17A	chr7	43664279	43664280	A	G	dbsnp.86:rs1044141	MISSENSE	1234	362	K	E	hom	hom	hom	Y
STK31	chr7	23757161	23757162	G	C	dbsnp.116:rs6945306	MISSENSE	271	48	Q	H	het	het	het	Y
STK31	chr7	23794028	23794029	G	A	dbsnp.111:rs4722266	MISSENSE	1287	387	G	E	het	het	het	Y
STK33	chr11	8435076	8435078	CA	TC	dbsnp.107:rs3751095;dbsnp.107:rs3751096	MISSENSE	1830	436	DA	ET	het	het	het	N
STK33	chr11	8435077	8435078	CA	TC	rs3751095;rs3751096	MISSENSE		436437		T	not called	not called	not called	Y

STK35	chr20	2082731	2082732	C	G	dbsnp.114:rs6112857	MISSENSE	204	69	R	G	hom	hom	hom	N
STK35	chr20	2083576	2083577	G	A		MISSENSE	457	153	R	Q	het	not called	not called	N
STK36	chr2	219555261	219555262	G	A	dbsnp.88:rs1344642	MISSENSE	2013	583	R	Q	het	not called	het	Y
STK36	chr2	219562674	219562675	G	A	dbsnp.92:rs1863704	MISSENSE	3273	1003	G	D	het	not called	het	Y
STK40	chr1	36807480	36807481	C	T	dbsnp.107:rs3795498	MISSENSE	1589	395	A	T	het	het	het	Y
STYK1	chr12	10782114	10782115	T	C	dbsnp.107:rs3759259	MISSENSE	1130	204	S	G	hom	hom	hom	Y
TAF15	chr17	34171977	34171977		GGAGGAGACCGAGGTGGGGGCTAC		INSERT	1751	569		GGYGGDRG	not called	hom	not called	N
TAF1L	chr9	32632531	32632532	G	A	dbsnp.126:rs35905429	MISSENSE	3135	1016	R	C	het	not called	het	Y
TAOK3	chr12	118682750	118682751	C	T	dbsnp.80:rs428073	MISSENSE	630	47	S	N	het	not called	het	Y
TBX18	chr6	85473757	85473758	C	T	dbsnp.79:rs172562	MISSENSE	141	48	G	R	not called	het	het	N
TCERG1L	chr10	132896608	132896609	C	G		MISSENSE	1649	522	E	Q	not called	not called	hom	N
TEC	chr4	48178100	48178101	G	C		MISSENSE	331	81	Q	E	het	het	not called	Y
TESK1	chr9	35609577	35609578	G	A	dbsnp.129:rs55673450	MISSENSE	2055	574	G	S	het	het	het	Y
TET1	chr10	70332671	70332672	T	A	dbsnp.121:rs12773594	MISSENSE	1081	193	S	T	het	het	het	Y
TET1	chr10	70332861	70332862	C	T	dbsnp.120:rs12221107	MISSENSE	1271	256	A	V	het	het	het	Y
TET1	chr10	70333509	70333510	G	A		MISSENSE	1919	472	G	D	not called	het	het	Y
TET1	chr10	70404532	70404533	C	G	dbsnp.134:rs139785845	MISSENSE	2551	683	Q	E	het	het	half called	Y
TET1	chr10	70405538	70405539	A	G	dbsnp.123:rs16925541	MISSENSE	3557	1018	N	S	het	not called	not called	Y
TET1	chr10	70405854	70405855	A	G	dbsnp.108:rs3998860	MISSENSE	3873	1123	I	M	het	half called	half called	Y
TET2	chr4	106156186	106156187	C	T	dbsnp.123:rs17253672	MISSENSE	1473	363	P	L	het	het	het	Y
TET2	chr4	106196828	106196829	T	G	dbsnp.126:rs34402524	MISSENSE	5547	1721	L	W	het	het	het	N
TEX14	chr17	56659017	56659018	C	T	dbsnp.116:rs6503870	MISSENSE	3362	1082	G	D	hom	hom	hom	Y
TEX14	chr17	56665373	56665374	C	G		MISSENSE	2702	862	R	T	het	not called	het	Y
TEX14	chr17	56676367	56676368	T	C	dbsnp.80:rs389389	MISSENSE	2455	780	N	D	het	het	het	Y
TEX14	chr17	56679810	56679811	G	A		NONSENSE		499	R	*	not called	not called	not called	Y
TFRC	chr3	195800810	195800811	C	T	dbsnp.107:rs3817672	MISSENSE	565	142	G	S	het	het	het	Y
TG	chr8	133899183	133899184	T	C	dbsnp.132:rs116062097	MISSENSE	1607	523	S	P	het	hom	het	Y
TG	chr8	133900251	133900252	T	G	dbsnp.79:rs180223	MISSENSE	2240	734	S	A	het	not called	het	Y
TG	chr8	133909973	133909974	A	G	dbsnp.86:rs853326	MISSENSE	3122	1028	M	V	het	not called	het	Y
TG	chr8	133920517	133920518	A	G	dbsnp.96:rs2069556;dbsnp.130:rs75865560	MISSENSE	3975	1312	D	G	hom	hom	hom	Y
TG	chr8	133975282	133975283	G	A	COSMIC:mut:150550;dbsnp.96:rs2069561	MISSENSE	5552	1838	D	N	het	het	het	Y
TG	chr8	133984057	133984058	C	T	dbsnp.96:rs2076740	MISSENSE	6035	1999	R	W	hom	hom	hom	Y
TGFB1	chr19	41858920	41858921	G	A	dbsnp.89:rs1800470	MISSENSE	910	10	P	L	het	not called	not called	N
TGFBR2	chr3	30686251	30686252	G	C		MISSENSE	564	61	M	I	het	het	hom	Y
TGS1	chr8	56686223	56686224	T	C	dbsnp.36:rs1818	MISSENSE	433	16	I	T	hom	hom	hom	Y
THRAP3	chr1	36752432	36752433	C	T	dbsnp.116:rs6425977	MISSENSE	825	201	A	V	hom	hom	hom	Y
TIAM1	chr21	32639196	32639197	C	T	dbsnp.134:rs141720377	MISSENSE	563	31	R	H	het	not called	het	Y
TLK2	chr17	60601612	60601613	C	G	dbsnp.100:rs2598147	MISSENSE	554	95	A	G	not called	het	not called	N
TLR4	chr9	120475301	120475302	A	G	dbsnp.113:rs4986790	MISSENSE	1186	299	D	G	het	het	het	Y
TLR4	chr9	120475601	120475602	C	T	dbsnp.118:rs4986791	MISSENSE	1486	399	T	I	het	het	het	Y
TMPO	chr12	98921738	98921739	G	T	dbsnp.132:rs113967157	MISSENSE	649	119	D	Y	het	het	not called	Y
TMPRSS2	chr21	42879908	42879909	C	A	dbsnp.132:rs75603675	MISSENSE	83	8	G	V	hom	not called	half called	N
TNFRSF10B	chr8	22886019	22886020	A	G	dbsnp.121:rs13265018	MISSENSE	864	191	V	A	hom	hom	hom	Y
TNFRSF11B	chr8	119964051	119964052	G	C	dbsnp.96:rs2073618	MISSENSE	331	3	N	K	het	het	het	Y
TNFRSF13C	chr22	42322701	42322702	C	G		MISSENSE	119	26	D	H	het	not called	not called	N
TNFRSF17	chr16	12060162	12060163	A	G	dbsnp.80:rs373496	MISSENSE	459	81	N	S	hom	hom	hom	Y
TNFRSF8	chr1	12169700	12169701	A	G		MISSENSE	721	167	K	R	het	hom	half called	Y
TNFRSF8	chr1	12175728	12175729	C	T	dbsnp.89:rs1763642	MISSENSE	1110	297	R	C	hom	hom	hom	Y
TNK1	chr17	7292106	7292107	G	A	dbsnp.116:rs6503018	MISSENSE	1936	593	V	M	hom	hom	hom	Y
TNK2	chr3	195591055	195591056	C	T	dbsnp.121:rs13433937	MISSENSE	3401	1086	R	H	het	het	not called	Y
TNN	chr1	175046788	175046789	A	G	dbsnp.96:rs2072032	MISSENSE	347	79	R	G	het	het	het	Y
TNN	chr1	175087728	175087729	T	C	dbsnp.116:rs6696455	MISSENSE	2531	807	W	R	het	het	het	Y
TNN	chr1	175092673	175092674	C	T	COSMIC:mut:146692;dbsnp.100:rs2285215	MISSENSE	2901	930	P	L	hom	hom	hom	Y
TNN	chr1	175092706	175092707	C	T	dbsnp.120:rs10798333	MISSENSE	2934	941	T	M	het	het	het	Y
TNNI3K	chr1	74701144	74701145	T	G		MISSENSE	60	4	Y	D	het	het	het	N
TOP2A	chr17	38552686	38552687	G	T		MISSENSE	3726	1190	Q	K	not called	het	not called	N
TOP2A	chr17	38555188	38555189	C	G		MISSENSE	3447	1097	V	L	not called	het	het	Y
TOPBP1	chr3	133368361	133368362	T	G	dbsnp.105:rs3192149	MISSENSE	1500	457	K	Q	het	het	het	Y
TOX	chr8	59851906	59851907	T	C	dbsnp.129:rs61753688	MISSENSE	585	122	N	S	het	het	het	Y
TP53	chr17	7577084	7577085	C	T	COSMIC:mut:10722;COSMIC:mut:137087;dbsnp.132:rs112431538	MISSENSE	1049	285	E	K	hom	hom	hom	Y

TP53	chr17	7579471	7579472	G	C	dbsnp.86:rs1042522	MISSENSE	411	72	P	R	hom	hom	hom	N
TP53RK	chr20	45315785	45315786	C	T	dbsnp.126:rs34983477	MISSENSE	590	123	R	Q	het	het	het	Y
TPH1	chr11	18051104	18051105	G	A	dbsnp.134:rs145479597	MISSENSE	449	142	R	C	het	het	not called	Y
TPO	chr2	1481154	1481155	G	T	dbsnp.100:rs2280132	MISSENSE	1207	373	A	S	hom	hom	hom	N
TPO	chr2	1481230	1481231	G	C	dbsnp.96:rs2175977	MISSENSE	1283	398	S	T	hom	hom	hom	N
TPO	chr2	1520675	1520676	T	C	COSMIC:mut:148793;dbsnp.86:rs1126799	MISSENSE	2630	847	V	A	het	not called	not called	Y
TPR	chr1	186321241	186321242	C	T	COSMIC:mut:146711;dbsnp.129:rs61744267	MISSENSE	2631	779	V	I	het	het	not called	Y
TRIB2	chr2	12858443	12858444	A	G	dbsnp.129:rs55813198	MISSENSE	1446	4	H	R	het	het	not called	Y
TRIM33	chr1	114948280	114948281	A	G	dbsnp.116:rs6537825	MISSENSE	2602	840	I	T	hom	hom	hom	Y
TRIM36	chr5	114462354	114462355	C	T	dbsnp.101:rs2974617	MISSENSE	2540	678	D	N	het	het	half called	Y
TRIM42	chr3	140401692	140401693	A	G	dbsnp.86:rs698673	MISSENSE	936	244	K	R	hom	hom	hom	N
TRIM42	chr3	140406946	140406947	G	A	dbsnp.125:rs28594654	MISSENSE	1628	475	V	M	hom	hom	hom	Y
TRIM42	chr3	140407259	140407260	C	A	dbsnp.119:rs9876490	MISSENSE	1941	579	A	E	hom	half called	hom	Y
TRIM67	chr1	231344907	231344908	A	G	COSMIC:mut:24030;dbsnp.132:rs114415891	MISSENSE	2076	679	M	V	het	het	not called	Y
TRIP11	chr14	92465748	92465749	C	T	dbsnp.126:rs35007347	MISSENSE	5100	1576	R	H	het	het	het	Y
TRIP11	chr14	92480757	92480759	TC	AA		MISSENSE	1359	329	R	I	not called	not called	het	N
TRPM6	chr9	77376646	77376647	T	C	COSMIC:mut:150618;dbsnp.100:rs2274924	MISSENSE	4806	1579	K	E	het	het	het	Y
TRPM6	chr9	77377409	77377410	C	T	dbsnp.107:rs3750425	MISSENSE	4233	1388	V	I	het	het	het	Y
TRPM6	chr9	77502159	77502160	G	A	dbsnp.88:rs1333342	MISSENSE	103	2	T	I	het	het	het	N
TRPM7	chr15	50878629	50878630	G	A	dbsnp.116:rs8042919	MISSENSE	4726	1482	T	I	hom	hom	half called	Y
TSHR	chr14	81610582	81610583	G	C	dbsnp.92:rs1991517	MISSENSE	2336	727	E	D	het	not called	het	Y
TTBK1	chr6	43230969	43230970	G	C	dbsnp.107:rs3800294	MISSENSE	2146	623	G	A	het	het	half called	N
TTBK1	chr6	43231047	43231048	C	G	dbsnp.126:rs35175743	MISSENSE	2224	649	P	R	het	het	half called	N
TTL	chr2	113258845	113258846	A	C		MISSENSE	711	178	Q	P	not called	het	not called	N
TTN	chr2	179432184	179432185	A	G	dbsnp.120:rs12463674	MISSENSE	51703	17160	I	T	het	het	het	Y
TTN	chr2	179441931	179441932	G	A	dbsnp.129:rs55980498	MISSENSE	64431	21403	P	S	het	not called	not called	Y
TTN	chr2	179444767	179444768	C	G	dbsnp.110:rs4145333	MISSENSE	40275	13351	A	P	hom	hom	hom	Y
TTN	chr2	179545858	179545859	C	T	dbsnp.126:rs36051007	MISSENSE	29779	9852	R	H	het	hom	het	Y
TTN	chr2	179554304	179554305	C	T	dbsnp.100:rs2244492	MISSENSE	28356	9378	G	R	het	hom	half called	Y
TTN	chr2	179582852	179582853	T	C	dbsnp.130:rs72648982	MISSENSE	21372	7050	R	G	het	not called	het	Y
TTN	chr2	179590158	179590160	CT	GA		MISSENSE	17263	5680	K	I	not called	not called	het	N
TTN	chr2	179598522	179598523	G	C		MISSENSE	12085	3954	S	C	het	het	het	Y
TTN	chr2	179612649	179612650	A	T		MISSENSE	14701	4826	L	H	het	het	het	N
TTN	chr2	179615886	179615887	T	C	dbsnp.86:rs922984	MISSENSE	11464	3747	D	G	hom	hom	hom	N
TTN	chr2	179615930	179615931	C	G	dbsnp.86:rs922985	MISSENSE	11420	3732	L	F	hom	hom	hom	N
TTN	chr2	179620950	179620951	C	T	dbsnp.116:rs7585334	MISSENSE	10963	3580	G	D	hom	hom	hom	N
TTN	chr2	179621476	179621477	C	T	dbsnp.116:rs6433728	MISSENSE	10437	3405	A	T	hom	hom	hom	N
TTN	chr2	179623757	179623758	C	T	dbsnp.100:rs2291310	MISSENSE	10342	3373	S	N	hom	hom	hom	Y
TTN	chr2	179629460	179629461	C	T	dbsnp.100:rs2291311	MISSENSE	9867	3215	V	M	hom	hom	hom	Y
TTN	chr2	179644034	179644035	G	A	dbsnp.88:rs1552280	MISSENSE	3970	1249	S	L	hom	hom	half called	Y
TTN	chr2	179644854	179644855	T	C	dbsnp.119:rs10497520	MISSENSE	3687	1155	K	E	hom	hom	hom	Y
TTN	chr2	179650407	179650408	G	A	dbsnp.126:rs35813871	MISSENSE	2518	765	T	I	het	het	het	Y
TUFT1	chr1	151512894	151512895	A	G	dbsnp.107:rs3828054	MISSENSE	114	18	Q	R	het	het	het	N
TYK2	chr19	10469974	10469975	A	C	dbsnp.121:rs12720356	MISSENSE	2428	684	I	S	het	not called	het	Y
TYK2	chr19	10475651	10475652	C	A	dbsnp.100:rs2304256	MISSENSE	1461	362	V	F	het	not called	het	Y
ULK1	chr12	132401565	132401566	C	T	COSMIC:mut:330256;dbsnp.120:rs11546871	MISSENSE	2408	714	P	L	het	not called	not called	Y
ULK2	chr17	19713739	19713740	C	T	dbsnp.79:rs150122	MISSENSE	1616	370	V	M	hom	hom	hom	Y
ULK3	chr15	75130092	75130093	T	C	dbsnp.121:rs12898397	MISSENSE	1439	445	K	R	hom	hom	hom	Y
ULK4	chr3	41841715	41841716	A	C	dbsnp.111:rs4973986	MISSENSE	2118	640	S	A	hom	hom	hom	Y
ULK4	chr3	41925397	41925398	C	T	dbsnp.86:rs1052501	MISSENSE	1824	542	A	T	hom	half called	not called	Y
ULK4	chr3	41960005	41960006	T	C	dbsnp.89:rs1716975	MISSENSE	870	224	I	V	hom	hom	hom	Y
ULK4	chr3	41996135	41996136	T	C	dbsnp.100:rs2272007	MISSENSE	316	39	K	R	hom	hom	not called	Y
UNC13C	chr15	54306924	54306925	G	A	dbsnp.120:rs12437941	MISSENSE	1824	609	G	S	hom	hom	hom	Y
URB2	chr1	229770672	229770673	G	A		MISSENSE	448	105	E	K	het	not called	het	Y
URB2	chr1	229772692	229772693	T	G	dbsnp.107:rs3811473	MISSENSE	2468	778	V	G	het	het	het	Y
USP6	chr17	5036273	5036274	C	A	COSMIC:mut:132780;COSMIC:mut:132781;dbsnp.131:rs78465432	MISSENSE	1963	89	H	N	not called	het	het	Y
USP6	chr17	5036280	5036281	G	C	dbsnp.131:rs76236903	MISSENSE	1970	91	S	T	het	het	het	Y
USP6	chr17	5036747	5036748	G	T	dbsnp.132:rs113754955	MISSENSE	1985	96	R	L	not called	not called	het	N
USP6	chr17	5042714	5042715	G	A	rs61740308	MISSENSE		415	C	Y	not called	not called	not called	Y
USP6	chr17	5042836	5042837	C	T	rs61745111	MISSENSE		456	R	W	not called	not called	not called	Y
USP6	chr17	5042893	5042894	T	C	rs8073787	MISSENSE		475	W	R	not called	not called	not called	Y

USP6	chr17	5045774	5045775	G	A		MISSENSE	3494	599	S	N	het	not called	not called	N
USP6	chr17	5058848	5058849	T	C		MISSENSE	4474	926	W	R	not called	not called	het	N
USP6	chr17	5058859	5058860	A	C		MISSENSE	4485	929	R	S	het	not called	het	N
USP6	chr17	5058885	5058886	T	A		MISSENSE	4511	938	I	N	het	not called	not called	N
USP6	chr17	5058899	5058900	C	T		MISSENSE	4525	943	R	C	het	not called	het	N
USP6	chr17	5064856	5064857	C	G		MISSENSE	4561	955	L	V	het	het	not called	Y
USP6	chr17	5072332	5072333	T	G	rs3816913	MISSENSE		1167	I	S	not called	not called	not called	Y
VDR	chr12	48272894	48272895	A	G	dbsnp.98:rs2228570	MISSTART	161	1	M	T	hom	hom	hom	Y
VEGFC	chr4	177605081	177605084	TCA			SPLICE_SITE_DEL		419	S		not called	not called	not called	Y
VEGFC	chr4	177650865	177650866	C	T	dbsnp.127:rs41278571	MISSENSE	611	61	R	Q	het	het	not called	Y
VPS13B	chr8	100133705	100133706	T	G	dbsnp.116:rs7460625	NONSENSE	1349	413	Y	*	hom	hom	hom	N
VPS13B	chr8	100779199	100779200	T	C		DISRUPT	7433	2441		GCAATT	het	het	half called	Y
VRK2	chr2	58316813	58316814	A	G	dbsnp.86:rs1051061	MISSENSE	683	167	I	V	het	hom	het	Y
WNK1	chr12	971290	971291	C	T	dbsnp.100:rs2286007	MISSENSE	2636	665	T	I	het	het	het	Y
WNK1	chr12	974308	974308		C	dbsnp.134:rs141823469;dbsnp.134:rs145230824;dbsnp.126:rs35706572	FRAMESHIFT	2815	726	I	H	het	het	het	N
WNK1	chr12	974355	974355		C	dbsnp.126:rs34967262	FRAMESHIFT	2862	741	F	L	hom	hom	hom	N
WNK1	chr12	990911	990912	A	C	dbsnp.86:rs956868	MISSENSE	4588	1316	T	P	hom	hom	hom	Y
WNK1	chr12	994486	994487	G	C	dbsnp.116:rs7955371	MISSENSE	5939	1766	C	S	hom	hom	hom	N
WNK1	chr12	998364	998365	G	T	dbsnp.121:rs12828016	MISSENSE	6846	2068	M	I	hom	hom	hom	Y
WNK2	chr9	95997205	95997206	G	C		MISSENSE	1191	398	E	Q	het	het	het	Y
WNK2	chr9	96021311	96021312	G	A	dbsnp.120:rs10761203	MISSENSE	2481	828	V	M	hom	hom	hom	Y
WNK3	chrX	54265338	54265349	TGCCGTAGCCT	AGGCTACGGCA		MISSENSE	4273	1279	RLRQ	CRSL	not called	het	not called	N
WRN	chr8	30921934	30921935	G	A	dbsnp.98:rs2230009	MISSENSE	1127	114	V	I	hom	hom	hom	Y
WRN	chr8	30948413	30948415	CT	TG		MISSENSE	2572	595	IS	IA	not called	not called	het	N
WRN	chr8	30973886	30973887	A	G		MISSENSE	3078	764	E	G	het	het	het	Y
WRN	chr8	30999196	30999198	GG	AA		MISSENSE	3926	1047	G	N	not called	not called	het	N
XPC	chr3	14187448	14187449	G	T	dbsnp.98:rs2228001	MISSENSE	2807	902	Q	K	hom	hom	hom	Y
XPC	chr3	14199886	14199887	G	A	dbsnp.98:rs2228000	MISSENSE	1488	462	A	V	hom	hom	hom	Y
XPC	chr3	14199907	14199908	C	T	dbsnp.98:rs2227999	MISSENSE	1467	455	R	H	hom	hom	hom	Y
ZAK	chr2	174128512	174128513	C	T	dbsnp.107:rs3769148	MISSENSE	1791	531	S	L	het	hom	hom	N
ZNF217	chr20	52192511	52192512	G	C	dbsnp.127:rs41274706	MISSENSE	3061	931	Q	E	het	half called	hom	Y
ZNF423	chr16	49671084	49671085	G	A		MISSENSE	2275	660	R	W	het	not called	het	Y
ZNF608	chr5	123980163	123980164	T	C	dbsnp.132:rs113873110	MISSENSE	4018	1299	K	R	het	not called	het	Y
ZNF608	chr5	123983914	123983915	G	T	dbsnp.116:rs6862252	MISSENSE	2284	721	T	N	het	not called	het	Y
ZNF643	chr1	40923018	40923019	G	A	dbsnp.100:rs2272994	MISSENSE	640	115	C	Y	het	het	het	Y
