# Supplementary material for: Whole genome sequence analysis of BT-474 using complete Genomics’ standard and long fragment read technologies
Source: Gigascience. 2016 Feb 9;5:8. doi: 10.1186/s13742-016-0113-x (PMC4748558; doi:10.1186/s13742-016-0113-x)
Supplement: Additional file 3: — Standard Sequencing Service Data File Formats v2. (PDF 6212 kb) [file 13742_2016_113_MOESM3_ESM.pdf]

Standard Sequencing Service
Data File Formats

File format v2.5

Software v2.5

October 2013

CGA Tools, cPAL, and DNB are trademarks of Complete Genomics, Inc. in the US and certain other countries. All other trademarks are the property of their respective owners.

Disclaimer of Warranties. COMPLETE GENOMICS, INC. PROVIDES THESE DATA IN GOOD FAITH TO THE RECIPIENT “AS IS.” COMPLETE GENOMICS, INC. MAKES NO REPRESENTATION OR WARRANTY, EXPRESS OR IMPLIED, INCLUDING WITHOUT LIMITATION ANY IMPLIED WARRANTY OF MERCHANTABILITY OR FITNESS FOR A PARTICULAR PURPOSE OR USE, OR ANY OTHER STATUTORY WARRANTY. COMPLETE GENOMICS, INC. ASSUMES NO LEGAL LIABILITY OR RESPONSIBILITY FOR ANY PURPOSE FOR WHICH THE DATA ARE USED.

Any permitted redistribution of the data should carry the Disclaimer of Warranties provided above.

Data file formats are expected to evolve over time. Backward compatibility of any new file format is not guaranteed.

Complete Genomics data is for Research Use Only and not for use in the treatment or diagnosis of any human subject. Information, descriptions and specifications in this publication are subject to change without notice.

Copyright © 2011-2013 Complete Genomics Incorporated. All rights reserved.

RM_DFFSS_2.5-01

Table of Contents

Preface **6**
Conventions 6
Analysis Tools 6
References 6
Introduction **9**
Sequencing Approach 9
Mapping Reads and Calling Variations 9
Read Data Format 9
Data File Formats and Conventions **11**
Data File Structure 11
Header Format 11
Sequence Coordinate System 15
Data File Content and Organization 15
ASM Results **17**
Small Variations and Annotations Files 17
Variations 20
ASM/var-[ASM-ID].tsv.bz2 20
Master Variations 27
ASM/masterVarBeta-[ASM-ID].tsv.bz2 27
Individual Genomes' Small Variations, CNVs, SVs, and MEIs in VCF Format 35
ASM/vcfBeta-[ASM-ID].vcf.bz2 35
Annotated Variants within Genes 54
ASM/gene-[ASM-ID].tsv.bz2 54
Annotated Variants within Non-coding RNAs 59
ASM/ncRNA-[ASM-ID].tsv.bz2 59
Count of Variations by Gene 61
ASM/geneVarSummary-[ASM-ID].tsv 61
Variations at Known dbSNP Loci 63
ASM/dbSNPAnnotated-[ASM-ID].tsv.bz2 63
Sequencing Metrics and Variations Summary 67
ASM/summary-[ASM-ID].tsv 67
Copy Number Variation Files 71
Copy Number Segmentation 73
ASM/CNV/cnvSegmentsDiploidBeta-[ASM-ID].tsv 73
Detailed Ploidy and Coverage Information 76
ASM/CNV/cnvDetailsDiploidBeta-[ASM-ID].tsv.bz2 76
Genomic Copy Number Analysis of Non-Diploid Samples Files 79
Non-diploid CNV Segments 80
ASM/CNV/cnvSegmentsNondiploidBeta-[ASM-ID].tsv.bz2 80
Detailed Non-Diploid Coverage Level Information 83
ASM/CNV/cnvDetailsNondiploidBeta-[ASM-ID].tsv.bz2 83

Depth of Coverage Report 86
ASM/CNV/depthOfCoverage_100000-[ASM-ID].tsv 86
Structural Variation Files....89
Detected Junctions and Associated Annotations....91
ASM/SV/allJunctionsBeta-[ASM-ID].tsv 91
High-confidence Junctions and Associated Annotations 96
ASM/SV/highConfidenceJunctionsBeta-[ASM-ID].tsv....96
Alignments of DNBs in Junction Cluster....97
ASM/SV/evidenceJunctionDnbBeta-[ASM-ID].tsv.bz2....97
Evidence Junctions and Annotations 100
ASM/SV/evidenceJunctionClustersBeta-[ASM-ID].tsv....100
Structural Rearrangement Events 103
ASM/SV/allSvEventsBeta-[ASM-ID].tsv and ASM/SV/highConfidenceSvEventsBeta-[ASM-ID].tsv....103
Mobile Element Insertion Files 108
Mobile Element Insertion Sites 110
ASM/MEI/mobileElementInsertionsBeta-[ASM-ID].tsv....110
Mobile Element Insertion ROC Graph 113
ASM/MEI/mobileElementInsertionsROCBeta-[ASM-ID].png....113
Mobile Element Insertion Reference Counts Graph 114
ASM/MEI/mobileElementInsertionsRefCountsBeta-[ASM-ID].png....114
Assemblies Underlying Called Variants Files....115
Results from Assembled Intervals....117
ASM/EVIDENCE/evidenceIntervals-[CHROMOSOME-ID]-[ASM-ID].tsv.bz2 117
Individual Reads Aligned to Assembled Sequences....120
ASM/EVIDENCE/evidenceDnbs-[CHROMOSOME-ID]-[ASM-ID].tsv.bz2 120
Correlation of Evidence between Assemblies 123
ASM/EVIDENCE/correlation.tsv.bz 123
Coverage and Reference Scores Files....125
Coverage and Reference Scores....126
ASM/REF/coverageRefScore-[CHROMOSOME ID]-[ASM ID].tsv.bz2....126
Quality and Characteristics of Sequenced Genome Files....128
Coverage Distribution Report File....129
ASM/REPORTS/coverage-[ASM-ID].tsv and ASM/REPORTS/coverageCoding-[ASM-ID].tsv....129
Coverage-by-GC-Content Report File 131
ASM/REPORTS/coverageByGcContent-[ASM-ID].tsv and ASM/REPORTS/coverageByGcContentCoding-[ASM-ID].tsv 131
Indel Net Length Report File....133
ASM/REPORTS/IndelLength-[ASM-ID].tsv 133
Indel Net Length in Coding Region Report File....134
ASM/REPORTS/IndelLengthCoding-[ASM-ID].tsv....134
Substitution Net Length File Report File 136
ASM/REPORTS/substitutionLength-[ASM-ID].tsv....136
Substitution Net Length in Coding Region Report File....137
ASM/REPORTS/substitutionLengthCoding-[ASM-ID].tsv 137

Circos Visualization of Small Variations, CNVs, SVs, and Associated Data: Non-Tumor Sample 139
ASM/REPORTS/circos-[ASM-ID].html and ASM/REPORTS/circos-[ASM-ID].png 139
Tumor Genome Circos Visualization of Small Variations, CNVs, SVs, and Associated Data 141
ASM/REPORTS/circos-[ASM-ID].html and ASM/REPORTS/circos-[ASM-ID].png 141
Library Information 143
Architecture of Reads and Gaps 144
LIB/lib_DNB_[LIBRARY-NAME].tsv 144
Empirically Observed Mate Gap Distribution 146
LIB/lib_gaps_M_[LIBRARY-NAME].tsv 146
Empirical Intraread Gap Distribution 147
LIB/lib_gaps_rollup_[ARM]_[LIBRARY-NAME].tsv 147
Sequence-dependent Empirical Intraread Gap Distribution 148
LIB/lib_gaps_[ARM][ID]_[LIBRARY-NAME].tsv 148
Reads and Mapping Data 150
Reads and Quality Scores 151
MAP/reads_[SLIDE-LANE]_00X.tsv.bz2 151
Initial Mappings 153
MAP/mapping_[SLIDE-LANE]_00X.tsv.bz2 153
Association between Initial Mappings and Reads Data 155
Glossary 156

List of Tables

Table 1: Header Metadata Present in all Data Files 12
Table 2: Header Metadata Present in Specific Files 12
Table 3: Sequence Coordinate System (Build 37) 15
Table 4: Content Description: Small Variant Data in *vcfBeta* Files 41
Table 5: Content Description of CNV Data in *vcfBeta* Files 46
Table 6: Content Description: Structural Variation Data in *VcfBeta* Files 48
Table 7: Content Description: MEI Data in *vcfBeta* Files 51
Table 8: Mobile Element Sequence Database 109
Table 9: Alignment CIGAR Format Modifiers in *evidenceDnbs-[CHROMOSOME-ID]-[ASM-ID].tsv.bz2* 116
Table 10: Alignment CIGAR Format Modifiers in *evidenceIntervals-[CHROMOSOME-ID]-[ASM-ID].tsv.bz2* 116
Table 11: Initial Mapping File Flags Field Values 155

List of Figures

Figure 1: Gapped Read Structure 10
Figure 2: Genome Data File Structure 16
Figure 3: ASM Directory Structure 18
Figure 4: *vcfBeta-[ASM-ID].vcf.bz2* File: Meta Information 35
Figure 5: *vcfBeta-[ASM-ID].vcf.bz2* File: Header with Seven of Eight Mandatory Data Columns 38
Figure 5 continued: *vcfBeta-[ASM-ID].vcf.bz2* File: Header with Eighth Mandatory Data Column (INFO) and Two Additional Columns (FORMAT and Sample) 38
Figure 6: CNV Directory Contents 71
Figure 7: Plot of GC Bias-Corrected Coverage across Chromosome 1 87
Figure 8: SV Directory Contents 89
Figure 9: Example of a Typical Junction 90

Figure 10: MEI Directory Contents 108
Figure 11: Mobile Element Insertion ROC Graph 113
Figure 12: Mobile Element Insertion Reference Counts Graph 114
Figure 13: EVIDENCE Directory Contents 115
Figure 14: REF Directory Contents 125
Figure 15: REPORTS Directory Contents 128
Figure 16: Plot of Genome-wide Coverage Distribution Generated from File *coverage-[ASM-ID].tsv* 129
Figure 17: Unique Sequence Coverage by GC Content 131
Figure 18: Indel Net Length in Coding Region 134
Figure 19: Distribution of Substitution Net Length in Coding Regions 137
Figure 20: Non-Tumor Circos Visualization 139
Figure 21: Non-Tumor Circos Visualization Legend 140
Figure 22: Tumor Circos Visualization 141
Figure 23: Tumor Circos Visualization Legend 142
Figure 24: LIB Directory Contents 143
Figure 25: MAP Directory Contents 150
Figure 26: Allowed flag Field Values 151
Figure 27: Example Initial Mapping File *mapping_[SLIDE-LANE]_00X.tsv.bz2* 153

Preface

This document describes the organization and content of the format for complete genome sequencing data delivered by Complete Genomics, Inc. to customers and collaborators. The data include sequence reads, their mappings to a reference human genome, and variations detected against the reference human genome.

Conventions

This document uses the following notational conventions:

Notation	Description
italic	A field name from a data file. For example, the *varType* field in the variations data file indicates the type of variation identified between the assembled genome and the reference genome.
bold_italic	A file name from the data package. For example, each package contains the file ***manifest.all***.
[BOLD-ITALIC]	An identifier that indicates how to form a specific data file name. For example, a gene annotation file format includes the assembly ID for this genome assembly in the file name. This document represents the file name as ***gene-[ASM-ID].tsv.bz2*** where ***[ASM-ID]*** is the assemble ID.

Analysis Tools

Complete Genomics has developed several tools for use with your Complete Genomics data set. CGA™ Tools is an open source product to provide tools for downstream analysis of Complete Genomics data. For more information on CGA Tools, see www.completegenomics.com/sequence-data/cgatools.

References

You can find the following documents on the Complete Genomics web site:

www.completegenomics.com/customer-support/documentation

- Release Notes — indicates new features and enhancements by release.
- *Complete Genomics Variation FAQ* — Answers to frequently asked questions about Complete Genomics variation data.
- *Complete Genomics Service FAQ* — Answers to frequently asked questions about Complete Genomics products and services.
- *Complete Genomics Managing Data FAQ* — Answers to questions about preparing to receive the hard drives of data.
- *Complete Genomics CNV Methods* — Describes the processing steps and algorithmic details of the Complete Genomics CNV pipeline that is used to identify and score regions of genomic copy number variation.
- *Complete Genomics Small Variations Methods* — Describes the algorithmic details of the Complete Genomics Small Variant Caller that is used to identify and score small variants (SNPs, insertions, deletions, and block substitutions).
- *Complete Genomics Calibration Methods* — Methods used to calibrate Complete Genomics small variant quality scores to absolute error rate. Error calibration is based on replicate experiments conducted by Complete Genomics at various levels of coverage.

Also available from Complete Genomics:

- Complete Genomics *Science* Article — An article describing the methodology and performance of the Complete Genomics sequencing platform. (*Science* 327 (5961), 78. [DOI: 10.1126/science.1181498]) This document is available on the *Science* web site:

  www.sciencemag.org/cgi/content/abstract/1181498?ijkey=2cSK/YvTtuDSU&keytype=ref&siteid=sci

  We recommend you read the *Complete Genomics Service FAQ* as background for this document.

- "Computational Techniques for Human Genome Resequencing Using Mated Gapped Reads" — An article describing the original Complete Genomics computational methods for small variant detection. These methods have evolved over the development of further Analysis Pipeline versions. (*Journal of Computational Biology*, Volume: 19 Issue 3: March 8, 2012) This document is available on the Liebert web site:

  online.liebertpub.com/doi/full/10.1089/cmb.2011.0201

- *CGA Tools User Guide* — Complete Genomics Analysis Tools (CGA™ Tools) is an open source project to provide tools for downstream analysis of Complete Genomics data. This document describes how to install and use the tools and provides information on the underlying algorithms.

  cgatools.sourceforge.net

- Baseline Genome Set — The data used to generate the baseline genome set is comprised of 52 unrelated genomes from the Complete Genomics Diversity Panel. The following summaries are available of this data:
  - CNV Baseline Genome Dataset: Summary of the underlying data and normalization constants for each of the CNV baseline genomes. The accompanying *Data Format Description* document provides the identifiers for each genome in the CNV baseline set and describes the data file format for the CNV baseline genome composite file. Available from the Complete Genomics FTP site. [ftp://ftp2.completegenomics.com/Baseline_Genome_Set/CNVBaseline]
  - SV Baseline Genome Dataset: Summary of the detected junctions and their frequencies across the SV baseline set. The accompanying *Data Format Description* document provides the identifiers for each genome in the SV baseline set and describes the data file format for the SV baseline genome composite file. Available from the Complete Genomics FTP site. [ftp://ftp2.completegenomics.com/Baseline_Genome_Set/SVBaseline]

The following references appear in this *Data File Formats* document:

- bzip2 — The open-source application with which much of the Complete Genomics data is compressed. [www.bzip.org]
- SAM— The Sequence Alignment/Map format is a generic format for storing large nucleotide sequence alignments. Where possible, the Complete Genomics data conforms to this standard. [www.samtools.sourceforge.net]
- Reference human genome assembly — All Complete Genomics genomic coordinates are reported with respect to the NCBI Build indicated in the header of each file. [www.ncbi.nlm.nih.gov/projects/mapview/map_search.cgi?taxid=9606&build=previous]
- ASCII-33 — The encoding used to represent quality scores and probabilities. [maq.sourceforge.net/fastq.shtml]
- Quality scores — Phred-like scores used to characterize the confidence in mapping quality, base call, and variant call. [en.wikipedia.org/wiki/Phred_quality_score]
- Sha256 and sha256sum — Checksum format and utility used to check the integrity of the Complete Genomics data files. [en.wikipedia.org/wiki/Sha1sum]

- Reference Sequence (RefSeq) Information — Functional impact of variants in the coding regions of genes is determined using RefSeq annotation data. Refer to the following sources:
  - RefSeq — Database of reference sequences annotations of DNA. [www.ncbi.nlm.nih.gov/refseq/]
  - Release Notes — Information on a given annotation build. [www.ncbi.nlm.nih.gov/genome/guide/human/release_notes.html]
  - RefSeq Alignment Data — Reference assembly and alignment data. This data is available here:

    ftp://ftp.ncbi.nih.gov/genomes/H_sapiens/ARCHIVE/

    Select the appropriate release, such as Annotation Release 104, then navigate to:

    ```
    mapview/seq_gene.md.gz
    ```
- Catalogue Of Somatic Mutations In Cancer (COSMIC) — Database designed to store and display somatic mutation information and related details. [www.sanger.ac.uk/genetics/CGP/cosmic]
- Database of Genomic Variants (DGV) — Database describing structural variation in the human genome, including copy number variation (CNV). The information in this database is used to annotate called CNV segments that overlap with previously identified CNVs. [http://dgv.tcag.ca/dgv/app/home]
- Database of Single Nucleotide Polymorphism (dbSNP) — Database maintained by the National Center for Biotechnology Information to serve as a central repository for both single base nucleotide substitutions and short deletion and insertion polymorphisms. [www.ncbi.nlm.nih.gov/projects/SNP/index.html]
- RepeatMasker (from UCSC Genome Browser track) — Database of DNA sequences for interspersed repeats and low complexity DNA sequences. [genome.ucsc.edu/cgi-bin/hgTrackUi?hgsid=194787809&c=chr21&g=rmsk]
- Variant Call Format (VCF) (from the 1000 Genomes Project) — A standard for encoding structural variations in a text format. [www.1000genomes.org/wiki/Analysis/Variant%20Call%20Format/vcf-variant-call-format-version-41]

Introduction

This document describes the directory structure and file formats for complete genome sequences delivered by Complete Genomics, Inc. to customers. The data include sequence reads, their mappings to a reference human genome, and variations detected against the reference human genome.

Sequencing Approach

Complete Genomics sequencing platform employs high-density DNA nanoarrays that are populated with DNA nanoballs (DNBs™) and base identification is performed using a non-sequential, unchained read technology, known as combinatorial probe-anchor ligation (cPAL™).

Complete Genomics sequencing technology, including the general library construction process and ligation-based assay approach, is described in the Complete Genomics *Science* Article. Note that methods have evolved since the writing of this paper.

Mapping Reads and Calling Variations

Complete Genomics reads are initially mapped to the reference genome using a fast algorithm. These initial mappings are both expanded and refined by a form of local *de novo* assembly in all regions of the genome that appear to contain variation (SNPs, indels, and block substitutions) based on these initial mappings. The *de novo* assembly fully leverages mate-pair information, allowing reads to be recruited into variant calling with higher sensitivity than genome-wide mapping methods alone typically provide. Assemblies are diploid, and Complete Genomics produces two separate result sequences for each locus in diploid regions (exception: for males, the non-pseudo-autosomal regions are assembled as haploid). Variants are called by independently comparing each of the diploid assemblies to the reference.

Complete Genomics uses initial mappings only to identify regions of potential variation and to identify informative reads for each such region. Because of the division of labor between our mapping and assembly processes, our initial mappings have a somewhat different character than mappings often produced for other platforms. For example, calling SNPs directly from these initial alignments tends to produce suboptimal results compared to those provided from Complete Genomics local *de novo* assembly approach.

The original Complete Genomics computational methods for small variant detection are described in "Computational Techniques for Human Genome Resequencing Using Mated Gapped Reads", in the *Journal of Computational Biology*. These methods have evolved over the development of further Analysis Pipeline versions.

Read Data Format

Each slide containing an ultra-high density DNA nanoarray is partitioned into several lanes. Each region within a lane imaged at one time is a "field"; each field covers a two-dimensional array of spots on the slide, the vast majority of which are occupied by 0 or 1 DNB. The DNB is a head-to-tail concatamer consisting of more than 200 copies of a circular DNA template comprised of genomic DNA and two synthetic adaptors. A library is a collection of these paired-end constructs processed together from genomic DNA and the known adaptors. Figure 1 depicts the architecture of the circular template and of the reads generated from a single DNB.

Figure 1: Gapped Read Structure

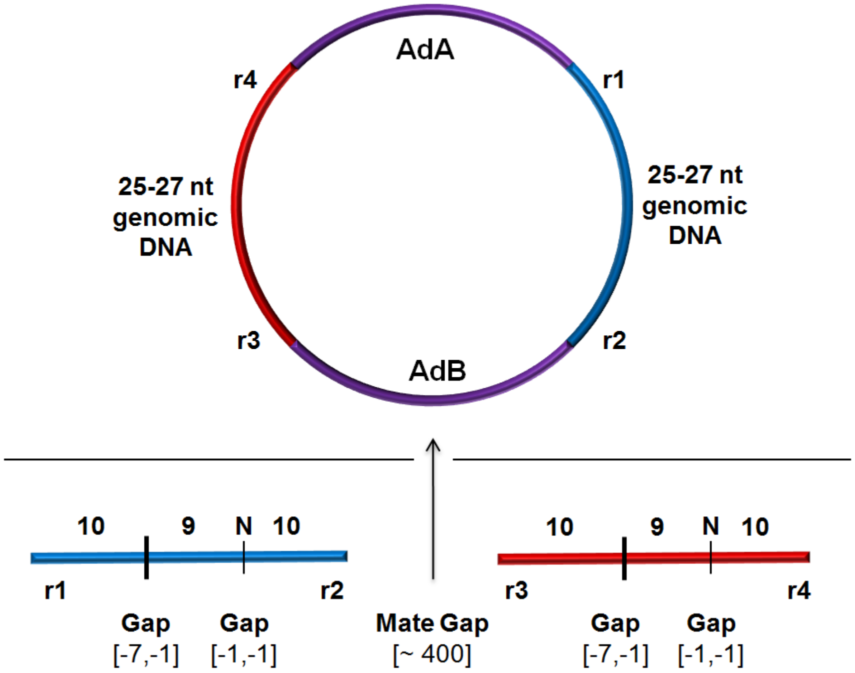

Each DNB consists of two paired reads, called half-DNBs, separated by a physical distance referred to as the "mate gap." Within each half-DNB (blue and red bands), sub-reads of genomic DNA are obtained from the ends of each adaptor. Sub-reads r1 and r2 correspond to one half-DNB, reads r3 and r4 correspond to a second half-DNB. The two half-DNBs are separated by two adapters (purple bands), which are not read during sequencing. Sub-reads within a half-DNB may overlap one another in genomic coordinates. The range of overlaps are indicated at the bottom of the figure as negative gap values, in bases. Note that one artifical unknown base (N) is inserted into each half-DNB to meet Complete Genomics mapping algorithm requirements, but this base is removed during read mapping and assembly through the use of a one base negative gap. Actual gap distributions are empirically estimated from sampled data and are provided in the data package. DNB positions in output files refer to positions within an aggregation of the sub-reads obtained from each DNB. In Figure 1, these are positions within the 60 bases (10 + 9 + N + 10 + 10 + 9 + N + 10) constructed by aggregating reads r1 – r4 in order of genomic position. Note that because proximal sub-reads (such as r1 and r2 in Figure 1) can overlap, two read positions may correspond to a single genomic location.

Data File Formats and Conventions

Data File Structure

Each data file corresponding to a single genome includes the following sections:

- Header: describes the file content and contains associated metadata in the form of key-value pairs. The header indicates the type of the data in the file, for example, "reads" data or "mapping" data. See "Header Format."
- Column headers: single row of tab-separated column headers that begins with the "greater than" character (>). The column headers reflect the data content in the file and are illustrated for each file type in "Data File Content and Organization."
- Data: ASCII data in a tab-separated format. The data content in each type of file is described in "Data File Content and Organization."

The following example shows a gene variation summary file:

```
#ASSEMBLY_ID GS19240-ASM
#BUILD 1.7
#DBSNP_BUILD dbSNP build 129
#GENERATED_AT 2010-Jan-21 13:42:57.076648
#GENERATED_BY callannotate
#GENE_ANNOTATIONS NCBI build 37.2
#GENOME_REFERENCE NCBI build 37
#TYPE GENE-VAR-SUMMARY-REPORT
#VERSION 0.6

>column-headers
Data
```

Complete Genomics enforces a 5 GB limit on the size of any data file when generating the package. If a data file becomes too large, it will be split into multiple files. The resulting collection of files is known as a "batch." Each file in the batch has a copy of the original header and additional header fields that are specific to a batch, such as a BATCH_FILE_NUMBER. A batch file repeats the structure of the original file but contains a contiguous subset of the original file data.

The original file can be restored by concatenating the batch files, without their headers, in their BATCH_FILE_NUMBER order. Some data files from the export package refer to the other files in the split format and use the keys FILE_ID, BATCH_FILE_NUMBER, and RECORD_NUMBER to refer the data. The files that are split include reads and mappings files.

Data files from some software versions are signed using S/MIME technology to ensure data integrity, using the PKCS #7 secure message format specification (Public Key Cryptography Standards #7, published by RSA Security). Contact our Technical Support for more information (support@completegenomics.com).

Header Format

Each data file in the directory structure contains a header section that describes the contents of the file and provides associated metadata. Each header row begins with the hash character (#) followed by a tab-separated, key-value pair. Table 1 describes header items that are present in all data files including the header keys and their possible values. Table 2 provides this information for file-specific header items. Not all files have all header values; refer to the individual file description for details on headers specific to that file.

Table 1: Header Metadata Present in all Data Files

Key	Description	Allowed Values
#CUSTOMER_SAMPLE_ID	Sample ID provided by customer.	Arbitrary text value.
#SAMPLE_SOURCE	Sample source provided on Sample Manifest.	Arbitrary text value.
#REPORTED_GENDER	Gender reported by customer.	`Male`, `Female`, `Unknown`
#CALLED_GENDER	Gender called by Analysis Pipeline.	`Male`, `Female`, `Unknown`
#TUMOR_STATUS	Tumor status reported by customer on Sample Manifest.	`Yes`, `No`
#LIBRARY_TYPE	Distinguishes between libraries generated using Complete Genomics' standard process or Long-Fragment Read Technology (LFR).	▪ `PureLFR`: Data from sample was sequenced only using LFR.<br>▪ `Mixed-LFR`: LFR data from sample was sequenced independently using both LFR and standard libraries.<br>▪ `Mixed-STD`: Non-LFR data from sample was seqenced independently using both LFR and standard libraries.<br>▪ `Standard`: Data from sample was sequenced only using Complete Genomics' standard process.
#LIBRARY_SOURCE	Library generation process used to create genomic clones for sequencing.	▪ `V1`: 4-adaptor based library.<br>▪ `V2`: 2-adaptor based library.

Table 2: Header Metadata Present in Specific Files

Key	Description	Allowed Values
#TYPE	Indicates the type of data contained in the file.	▪ `READS`: reads file.<br>▪ `MAPPINGS`: alignments of reads to the reference genome.<br>▪ `LIB-DNB`: description of the architecture of reads within DNBs in a library.<br>▪ `LIB-MATE-GAPS`: description of the empirically observed mate gap distribution for the library LIB-SMALL-GAPS-ROLLUP: description of the frequency of observation of gap tuples for the given arm for the library<br>▪ `LIB-SEQDEP-GAPS`: description of the frequency of observation of small gap values depending on nearby genomic sequence for the given arm for the library<br>▪ `REFMETRICS`: reference scores (scores indicating the likelihood of the assembled genome being identical to the reference at each genomic position) and coverage information.<br>▪ `DBSNP-TO-CGI`: information on loci annotated in dbSNP.<br>▪ `GENE-ANNOTATION`: variations annotated with impact on RefSeq genes.<br>▪ `SUMMARY-REPORT`: summary information on the assembled genome.<br>▪ `VAR-ANNOTATION`: information on the assembled genome, expressed relative to the reference genome.<br>▪ `GENE-VAR-SUMMARY-REPORT`: summary of genic variations in coding regions of genes.<br>▪ `EVIDENCE-CORRELATION`: information on

Key	Description	Allowed Values
		correlations in supporting data between pairs of genomic intervals.<br>▪ EVIDENCE-DNBS: DNB alignments supporting the called alleles in a genomic interval.<br>▪ EVIDENCE-INTERVALS: genomic intervals over which supporting evidence is provided for the called sequence.<br>▪ COVERAGE-DISTRIBUTION: count of bases sequenced at a given coverage depth.<br>▪ COVERAGE-BY-GC: normalized coverage by cumulative base GC percentage.<br>▪ DEPTH-OF-COVERAGE: coverage for each 100 kb non-overlapping window along the genome.<br>▪ INDEL-LENGTH-CODING: length of called indels in the coding region of the genome.<br>▪ INDEL-LENGTH: length of called indels in genome.<br>▪ SUBSTITUTION-LENGTH-CODING: length of called substitutions in the coding region of the genome.<br>▪ SUBSTITUTION-LENGTH: length of called substitutions in the genome.<br>▪ CNV-SEGMENTS: segmentation of the reference genome into regions of distinct ploidy.<br>▪ TUMOR-CNV-SEGMENTS: segmentation of the reference genome into regions of distinct coverage level.<br>▪ CNV-DETAILS-SCORES: estimated ploidy for every 2 kb non-overlapping window along the genome.<br>▪ TUMOR-DETAILS-SCORES: estimated coverage level for every 100 kb non-overlapping window along the genome.<br>▪ JUNCTIONS: information on detected junctions, expressed relative to the reference genome.<br>▪ JUNCTION-DNBS: DNB alignments supporting the called junctions in a genomic interval.<br>▪ SV-EVENTS: junctions composed into structural variation events.<br>▪ VAR-OLPL: information on the assembled genome, expressed relative to the reference genome in a one-line-per-locus format.<br>▪ MEI: information on detected mobile element insertion events, expressed relative to the reference genome.
#FORMAT_VERSION	Version number of the file format.	Two or more digits separated by periods. For example, “0.6”.
#LIBRARY	Identifier of the library from which the DNBs were generated.	
#SAMPLE	Identifier of the sample from which the library was created.	
#SLIDE	Flow slide identification code.	
#LANE	Identifier of the slide lane from which the reads were extracted.	

Key	Description	Allowed Values
#CHROMOSOME	Identifier of the chromosome that the reference score and coverage data apply to. Data for the pseudo-autosomal regions on chromosome Y are reported at their coordinates on chromosome X.	`chr1-chr22`, `chrM`, `chrX`, `chrY`
#ASSEMBLY_ID	Name of the assembly.	"`<assembly-name>-ASM`". For example, "GS000000474-ASM".
#SOFTWARE_VERSION	Assembly pipeline build number.	Two or more digits separated by periods.
#DBSNP_BUILD	dbSNP version used for annotation.	"`dbSNP build XXX`" where X's are digits.
#COSMIC	COSMIC version used for annotation.	"`COSMIC vXX`", where X's are digits. For example "COSMIC v48".
#PFAM_DATE	Date on which Pfam information was downloaded from NCBI Conserved Domain Database.	Day-Month-Year. For example "13-Aug-10".
#MIRBASE_VERSION	miRBase version used for annotation.	"`miRBase build XX`" where X's are digits.
#DGV_VERSION	DGV version used for annotation.	"`XX`", where X's are digits.
#GENERATED_AT	Date and time of the assembly.	Year-Month-Day Time.<br>For example "2010-Sep-08 20:27:52.457773".
#GENERATED_BY	Assembly pipeline component that generated the output.	Alpha-numeric string.
#GENE_ANNOTATIONS	NCBI annotation build.	"`NCBI build XX.X`" where X's are digits.
#GENOME_REFERENCE	Human genome build used for assembly.	"`NCBI build XX`" where X's are digits.
#BATCH_FILE_NUMBER	Number of the batch of a split data file.	Positive 1-based integer.
#BATCH_OFFSET	Offset of the first record in a batch to the position of the record in a non-split file.	Positive 0-based integer.
#FIELD_SIZE	Size of the lane fields.	Positive integer.
#MAX_PLOIDY	Maximum allowed copy number estimate.	Positive integer.
#WINDOW_SHIFT	Shift, in bases, between consecutive windows in which smoothed coverage is calculated for copy number estimation.	Positive integer.
#WINDOW_WIDTH	Width, in bases, of windows in which smoothed coverage is calculated for copy number estimation.	Positive integer.
#NUMBER_LEVELS	Number of coverage levels used for tumor CNV calling.	Positive integer.
#MEAN_LEVEL_X	Average relative coverage of level X, used for tumor CNV calling. X takes values from 0 to NUMBER_LEVELS-1, inclusive.	Positive floating point value.
#REPMASK_GENERATED_AT	Date and time on which repeat masker information was downloaded from the UCSC genome browser website	Year-Month-Day Time.<br>For example "2010-Sep-08 20:27:52.457773".

Key	Description	Allowed Values
#SEGDUP_GENERATED_AT	Date and time on which segmental duplication information was downloaded from the UCSC genome browser website.	Year-Month-Day Time. For example "2010-Sep-08 20:27:52.457773".
#MEI_1000G_ANNOTATIONS	Version of the 1000 genomes data set used for annotations.	INITIAL-DATA-RELEASE

Sequence Coordinate System

Sequence positions in the mapping and variations files are represented in half-open, zero-based coordinates, which denote locations between successive reference base positions. A substitution or deletion of the second base (T) in the sequence of length 8 below would have a start position of 1 and an end position of 2. An insertion following the same second base would have both a start and end position of 2.

```
0       1       2       3       4       5       6       7       8
|   A   |   T   |   A   |   G   |   G   |   C   |   T   |   A   |
```

Complete Genomics reports variants relative to the GRCh37 human reference genome. The version we use, "build 37," consists of the assembled nuclear chromosomes from GRCh37 (not unplaced or alternate loci), plus the Cambridge Reference Sequence for the mitochondrion (NC_012920.1). This assembly (though with an alternate mitochondrial sequence) is also known as UCSC hg19.

The FASTA sequence for build 37 is available at
ftp://ftp.completegenomics.com/ReferenceFiles/build37.fa.bz2

All genomic coordinates are reported with respect to the build indicated in the header of each file. All data for the pseudo-autosomal regions on the Y chromosome in males are reported at their coordinates on the X chromosome. The ranges of the two pseudo-autosomal regions on the sex chromosomes are listed in Table 3.

Table 3: Sequence Coordinate System (Build 37)

Pseudo-autosomal Region	Coordinates on Chromosome X	Coordinates on Chromosome Y
1	60000 – 2,699,519	10000 – 2,649,519
2	154,931,043 – 155,260,559	59,034,049 – 59,363,565

Data File Content and Organization

The data corresponding to a single genome is organized into three main directories:

- ASM – Assembly of the complete genome: variations called, coverage, and annotations
- LIB – DNB structure for the library used in the sequencing assay.
- MAP – Reads, quality scores, and alignments to the reference genome.

The representation of reads, quality scores, and alignments has been designed as a transfer format, dominated by considerations of simplicity and compactness.

© Complete Genomics, Inc.

The data is stored in the directory structure shown in Figure 2.

Figure 2: Genome Data File Structure

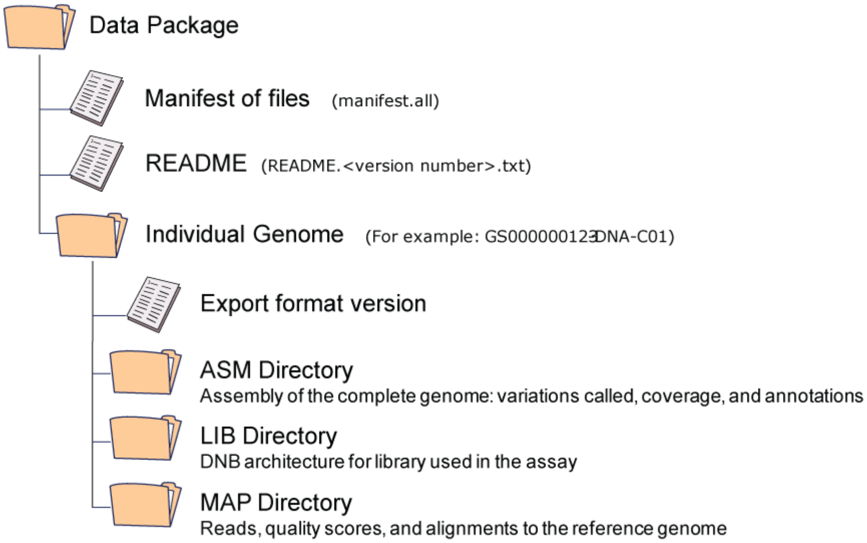

The files at the top-level of the organization apply to the package as a whole:

- ***README.<version number>.txt*** — Contains important information regarding the data delivered for each complete human genome sequenced by Complete Genomics Inc, organized by release version.
- ***manifest.all*** — a file containing the sha256-checksums for all files written to the disk.

In addition, the following file resides inside each individual genome directory:

- ***version*** — the version of the data file formats in this package.

ASM Results

The files in the ASM directory describe and annotate the genome assembly with respect to the reference genome. The ASM directory contains the primary results of the assembly within the following files. Each file includes a description of all loci where the assembled genome differs from the reference genome, but the files differ in format:

- The “variations” file: ***var-[ASM-ID].tsv.bz2***
- The “master variations” file: ***masterVarBeta-[ASM-ID].tsv.bz2***
- The “vcf” file: ***vcfBeta-[ASM-ID].vcf.bz2***.

The following file naming convention is enforced for files in the ASM directory:

`<filetype>-<ASM-ID>`

where `<filetype>` denotes the type of data included in the file and `<ASM-ID>` denotes the assembly ID, including only upper or lowercase letters, numbers, underscores, or hyphens; for example:

`geneVarSummary-GS000000474-ASM.tsv`

Renaming of Complete Genomics ASM files or writing code to process these files should take this convention into consideration. CGA Tools also considers this convention when handling ASM files.

Small Variations and Annotations Files

The files in the ASM directory describe and annotate the sample’s genome assembly with respect to the reference genome, including:

- Variations: The primary results of the assembly describing variant and non-variant alleles found.
- Master Variations: Results of the assembly describing variant and non-variant alleles found, with annotation information in a one-line-per-locus format.
- vcfBeta: Results of small variant, CNV, MEI, and SV detection with scores and annotations in VCF format.
- Genes: Annotated variants within known protein coding genes.
- ncRNAs: Annotated variants within non-coding RNAs
- Gene Variation Summary: Count of variants in known genes.
- DB SNP: Variations in known dbSNP loci.
- Variations and Annotations Summary: Statistics of sequence data to assess genome quality.

The ASM directory has the structure illustrated in Figure 3.

Figure 3: ASM Directory Structure

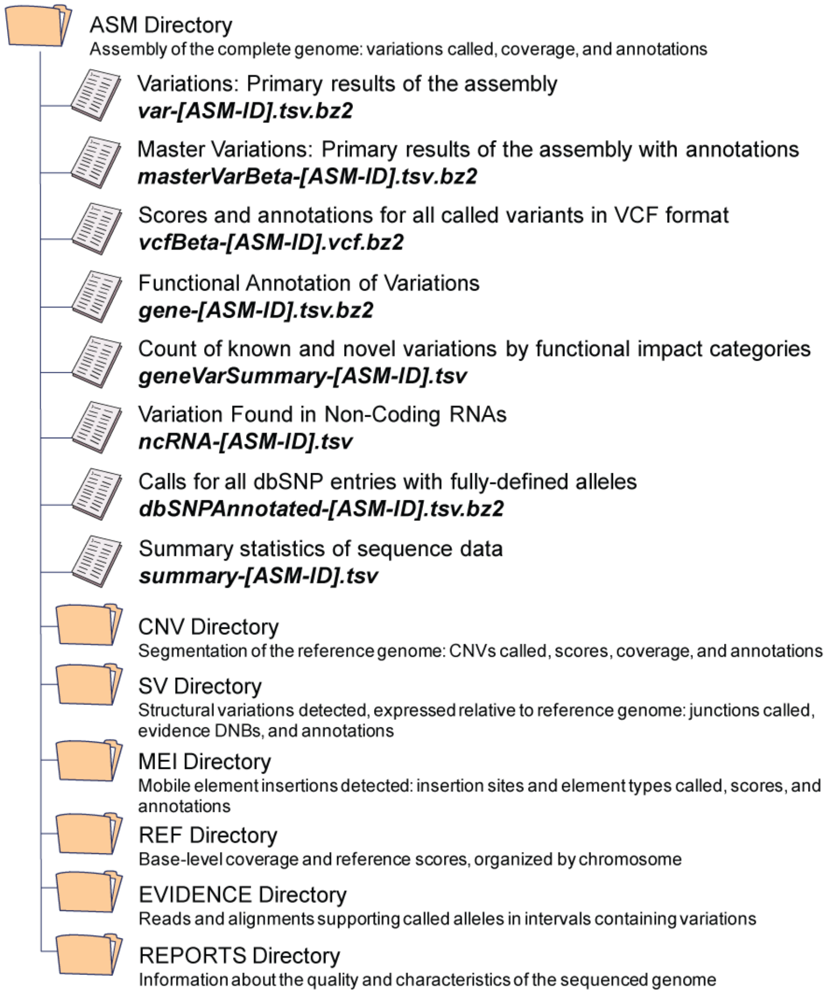

In addition to the variations file, the ASM directory includes annotations of the assembled sequence with respect to the SNP database (dbSNP), RefSeq transcripts, and protein sequences. The ASM directory includes the following subdirectories:

- CNV — Files containing the segmentation of reference genome into regions of distinct ploidy. For normal genomes, ploidy, coverage, confidence scores, and annotations are reported for each segment. For tumor genomes, called level, coverage, and confidence scores are reported for each segment.
- SV — Files containing detected junctions, supporting evidence DNB mappings, and associated annotations, including coordinates of breakpoint, putative structural variation size, confidence scores, and overlap with genomic elements.
- MEI — Files containing detected mobile element insertions and associated annotations, including event type, count of DNBs supporting event versus reference, confidence score for called event type, and overlap with genomic elements.
- REF — Files containing the sequence coverage at each reference genomic position determined from the initial mappings only and a score indicating the likelihood of the genome being homozygous and identical to the reference at each position.

- EVIDENCE — Results from the final *de novo* assemblies provides supporting information for intervals in the reference sequence where there is substantial evidence for variations from reference sequence.
- REPORTS — Files containing information that can be used to assess quality and characteristics of the sequenced genome, including distribution of coverage, coverage by GC content, and size of called indels and substitutions, genome-wide and in coding region.

The following sections describe the ASM results files.

Variations

ASM/var-[ASM-ID].tsv.bz2

Called variants in this file are cross-referenced with entries in dbSNP and the Catalogue of Somatic Mutation in Cancer (COSMIC). The versions of dbSNP and COSMIC used for the annotation can be found in the #DBSNP_BUILD and #COSMIC fields of the header section of this file.

The variation file contains records for each position in the reference genome, describing whether the corresponding position was called in the Complete Genomics data, and if so, whether it is called as reference (its sequence is same as the reference genome) or variant. This is done independently for each of the two diploid alleles of the sequenced genome.

Variations File Content

For all base positions in the reference genome that are presumed diploid, the variations file can have two records, one describing each of the two diploid alleles. In presumed haploid regions one should see only a single record for each base. Allele numbers 1 and 2 are assigned arbitrarily and one should not use these designations to infer phase (phase however will be indicated by the *haplink* field where it is known). For convenience, each range of positions is grouped into a "locus" based on the regions of variation on one or both alleles. The criteria for defining locus boundaries are standardized and applied evenly, but are also arbitrary: no notion of genetic inheritance (for example) is applied. See *Complete Genomics Variation FAQ* for more information on criteria used for defining locus boundaries.

Variations Type Description

For any record in the variations file describing a range of base(s) for an allele, the following designations may be used in the *varType* column:

- SNPs: "snp" in the *varType* column indicates a single base position that is called and was determined to be different than the reference sequence (technically, this is an "SNV", although we use the more common acronym "SNP" for convenience).
- Deletion events: "del" in the *varType* column indicates a region in which the reference genome includes one or more bases where the assembled allele sequence has no corresponding bases.
- Insertion events: "ins" in the *varType* column indicates a region where the allele sequence includes one or more bases where the reference sequence has no corresponding region. Insertion events have the same start and end positions indicating the inter-base position of the inserted sequence (using zero-based, half-open coordinates).
- Substitution events: "sub" in the *varType* column indicates that one or more bases in the reference are replaced by one or more bases in this sample. Substitutions can be length-conserving (the same number of bases as the corresponding reference sequence region) or length-altering (a different number). Standard rules are used to define when nearby variant bases are considered to be a larger substitution rather than a set of individual SNPs. See *Complete Genomics Variation FAQ* for more information.
- No-call events: "no-call" in the *varType* column indicates that an allele is either unresolved or is not completely resolved over reference sequence range. When some bases are resolved but others are not, an incomplete allele sequence is produced: In this case "no-call-rc" indicates that the called bases are consistent with the reference sequence. "no-call-ri" indicates that one or more of the called bases are inconsistent with (different than) the reference sequence. If the *allele* column is "all", the "no-call" indicates that neither allele is called.

  In some cases, one allele may have a "no-call" *varType* while the other allele has a called sequence (reference or variant). One cause of this is regions of lower coverage where the algorithms cannot distinguish a homozygote and an under-sampled heterozygote.

Occasionally one will see a zero-length no-call that has the same start and end position and a "?" for the allele sequence. This is an allele in the genome where we cannot rule out the possibility that there is an insertion present.

- Reference: "ref" in the *varType* column indicates that the corresponding allele sequence is the same as reference. If the *allele* column is "all" this means that both alleles are called reference and is shorthand for indicating that the region is called homozygous.
- Unspecified: "no-ref" in the *varType* column indicates that the reference sequence is unspecified over this region.
- Y chromosome: "PAR-called-in-X" in the *varType* column is used to indicate the pseudo-autosomal region of the Y chromosome in males. The called sequence for the PAR is reported as diploid sequence on the X chromosome.

Each of the two alleles is called separately by comparing the assembled allele sequence to the reference. For this reason, it is possible (and indeed happens) that some loci are *asymmetric*: the type of a variant on one allele (for example, a SNP) or the sequence of that variant may be quite different than that on the other allele. We call these "complex" variants.

Variants in this file are matched with entries in dbSNP, and those that match are annotated with the corresponding rs-ID. The version of dbSNP used for the annotation can be found in the #DBSNP_BUILD field of the header section of this file.

Ambiguous Calls

Ambiguous calls are made when there is strong evidence that an allele is not reference, but insufficient evidence to make a single high-confidence call. For these variants:

- The top scoring hypothesis is selected as the primary call.
- The allele is designated "AMBIGUOUS" in the *varFilter* column.
- A list of up to 200 additional supported variant calls and their relative scores is provided in the *alternativeCalls* column. These alternate calls must be non-reference and score within 10 dB of the primary call for homozygous calls and within 20 dB of the primary call for heterozygous alleles.
- Variant annotations are made relative to the primary call.

Example *ASM/var-[ASM-ID].tsv.bz2*

This example shows the kinds of variations identified in the variations file. Look for the following typical variations:

- Locus `974` is a "no-call" extending from position `5099` to `5126`, where both alleles are indeterminate in length and composition. The *allele* value of "all" is shorthand to indicate that both alleles are unresolved over this sequence range.
- Loci `975`, `977`, and `979` identify regions that are confirmed to be homozygous and identical to the reference sequence. In these cases, *varType* is "`ref`" and both the *reference* and *alleleSeq* fields are reported as "=", which is shorthand for the reference sequence over the specified sequence range.
- The first set of variations (locus ID=`976`) is an example of a homozygous SNP call, where the reference sequence is a "`G`" and the assembled genome has two copies of the "`T`" allele.

  The confidence score for the existence of at least one "`T`" allele is `87` under the equal allele fraction scoring model (*varScoreEAF*) and `97` under the maximum likelihood allele fraction scoring model (*varScoreVAF*).

  The confidence score for the existence of two "`T`" alleles is `58` under the equal allele fraction scoring model (*varScoreEAF*) and `19` under the maximum likelihood allele fraction scoring model (*varScoreVAF*).

The confidence flag is VQLOW for the second allele, because its *varScoreVAF* falls below the homozygous score threshold for high-quality homozygous loci, which is 20. (For loci that are not homozygous, the VQLOW confidence flag is applied if the *varScoreVAF* falls below 40.)

This variation has the dbSNP identifier "rs806".

- Locus 980 is an example of an insertion event in one of the alleles. An insertion of a "G" is seen at position 5363 in allele 1, while allele 2 has the reference sequence, with a *varType* of "ref". Note that for this locus, there is strong support for the insertion, but extreme allele imbalance, as indicated by the fact that the *varScoreVAF* is high but *varScoreEAF* is negative. For most variants in diploid regions of normal genomes, the *varScoreEAF* and *varScoreVAF* more nearly match.
- A homozygous deletion of a "T" is found in locus 982 at position 6464, indicated by the calling of a "del" variation in both alleles.
- A heterozygous SNP "C/T" call is found in locus 984, where reference shows a "C" and the assembled genome has a "C" allele in one allele and a "T" in the other.
- Locus 978 shows an example where only one of the two alleles is called. The assembled genome is identical to the reference (in this case, the bases "GTC") on one allele, while the other allele could not be fully called due to competing alternate hypotheses that could not be adequately discriminated. The *alleleSeq* column shows "?T?" in this case. The type of allele is "no-call".
- Locus 986 depicts a more complex situation, where there are three calls for one allele (1) and a "no-call" unresolved call for the other allele. There is only one variation call on allele 1 (a SNP at position 9564), but neither the length nor the composition of the sequence on the other allele could be reliably determined over this locus. The variant on allele 1 overlaps two known records in dbSNP (dbsnp.127:rs991 and dbsnp.137:rs994) as indicated in the *xRef* column. The second dbSNP record (but not the first) has a 1000 Genomes Project-measured minor-allele frequency of 0.03 as indicated in the *alleleFreq* column. Allele 2 has a value in the *haplink* column (780), which links this variation to the variation in locus 988 on allele 2. This indicates that these variations are in phase with one another.
- Locus 988 illustrates an "AMBIGUOUS" call (see the *varFilter* column), wherein a variation with respect to the reference is called but there is uncertainty regarding the exact sequence of the variation. The *alleleSeq* column and the *varType* show that the best reconstruction is a T->C SNP, but a T->CC substitution is indicated as an alternative in the *alternativeCalls* column, with a score of 13 dB separating the best from this alternative.

>locus	ploidy	allele	chromosome	begin	end	varType	reference	alleleSeq	varScoreVAF	varScoreEAF	varFilter	hapLink	xRef	alleleFreq	alternativeCalls
974	2	all	chr1	5099	5126	no-call	=	?							
975	2	all	chr1	5126	5145	ref	=	=							
976	2	1	chr1	5145	5146	snp	G	T	97	87	PASS		dbsnp.129:rs806		
976	2	2	chr1	5145	5146	snp	G	T	19	58	VQLOW		dbsnp.129:rs806		
977	2	all	chr1	5146	5212	ref	=	=							
978	2	1	chr1	5212	5215	ref	GTC	GTC	36	36	VQLOW				
978	2	2	chr1	5212	5215	no-call	GTC	?							
979	2	all	chr1	5215	5363	ref	=	=							
980	2	1	chr1	5363	5363	ins		G	123	-10	PASS				
980	2	2	chr1	5363	5363	ref			55	55	PASS				
981	2	all	chr1	5363	6464	ref	=	=							
982	2	1	chr1	6464	6465	del	T		57	57	PASS				
982	2	2	chr1	6464	6465	del	T		65	65	PASS				
983	2	all	chr1	6465	8600	ref	=	=							
984	2	1	chr1	8600	8601	ref	C	C	120	120	PASS				
984	2	2	chr1	8600	8601	snp	C	T	495	479	PASS				
985	2	all	chr1	8601	9559	ref	=	=							
986	2	1	chr1	9559	9563	ref	ACGG	ACGG	65	65	PASS	779			
986	2	1	chr1	9563	9564	snp	C	G	47	47	PASS	779	dbsnp.127:rs991; dbsnp.137:rs994	;dbnp:0.03	
986	2	1	chr1	9564	9566	ref	GT	GT	69	69	PASS	779			
986	2	2	chr1	9559	9566	no-call	ACGGCGT	?				780			
987	2	all	chr1	9566	9569	ref	=	=							
988	2	1	chr1	9569	9570	ref	T	T	46	43	PASS				
988	2	2	chr1	9569	9570	snp	T	C	21	22	AMBIGUOUS; VQLOW	780			CC:-13

File-Specific Header Description ASM/var-[ASM-ID].tsv.bz2

Key	Description	Allowed Values
#ASSEMBLY_ID	Name of the assembly.	"`<assembly-name>-ASM`". For example, "GS000000474-ASM".
#DBSNP_BUILD	dbSNP version used for annotation.	"`dbSNP build XXX`" where X's are digits. For example, "dbSNP build 130".
#COSMIC	COSMIC version used for annotation.	"`COSMIC vXX`", where X's are digits. For example "COSMIC v48".
#FORMAT_VERSION	Version number of the file format.	Two or more digits separated by periods. For example, "0.6".
#GENERATED_AT	Date and time of the assembly.	Year-Month-Day Time. For example "2010-Sep-08 20:27:52.457773".
#GENERATED_BY	Assembly pipeline component that generated the output.	Alpha-numeric string
#GENOME_REFERENCE	Human genome build used for assembly.	"`NCBI build XX`" where X's are digits.
#SAMPLE	Complete Genomics identifier of the sample from which the library was created.	"`GSXXXXX-DNA_YZZ`" where<br>▪ X's are digits<br>▪ `-DNA_` is literal<br>▪ YZZ is the location of the sample in a 96-well plate with Y as one of "`A`" through "`H`" and ZZ is one of "`01`" through "`12`"<br>For example "GS12345-DNA_A01"
#SOFTWARE_VERSION	Assembly pipeline version number.	Two or more digits separated by periods
#TYPE	Indicates the type of data contained in the file.	"`VAR-ANNOTATION`": information on the assembled genome, expressed relative to the reference genome.

Content Description ASM/var-[ASM-ID].tsv.bz2

	Column Name	Description
1	locus	Identifier of a particular genomic locus
2	ploidy	The *ploidy* of the reference genome at the locus (= 2 for autosomes, 2 for pseudo-autosomal regions on the sex chromosomes, 1 for males on the non-pseudo-autosomal parts of the sex chromosomes, 2 for mitochondrion, 2 if *varType* is `no-ref` or `PAR-called-in-X`). The reported ploidy is fully determined by gender, chromosome and location, and is not inferred from the sequence data.
3	allele	Identifier for each allele at the variation locus. For diploid genomes, 1 or 2. Shorthand of `all` is allowed where the *varType* field is one of `ref`, `no-call`, `no-ref`, or `PAR-called-in-X`. Allele numbering does not imply phasing; allele 1 in locus 1 is not necessarily in phase with allele 1 in locus 2. See *hapLink* for phasing information.
4	chromosome	Chromosome name in text: `chr1`, `chr2`, ..., `chr22`, `chrX`, `chrY`. The mitochondrial genome is represented as `chrM`. The pseudo-autosomal regions within the sex chromosomes X and Y are reported at their coordinates on chromosome X.
5	begin	Reference coordinate specifying the start of the variation (not the locus) using the half-open, zero-based coordinate system. See "Sequence Coordinate System" for more information.
6	end	Reference coordinate specifying the end of the variation (not the locus using the half-open, zero-based coordinate system. See "Sequence Coordinate System" for more information.

	Column Name	Description
7	varType	Type of variation, if any, for the range of bases. Currently must be one of `snp`, `ins`, `del`, `sub`, `ref`, `no-call-rc`, `no-call-ri`, `no-call`, `No-ref`, or `PAR-called-in-X`. See "Variations Type Description" for a description of the flags.
8	reference	The reference sequence for the locus of variation. Empty when *varType* is "`ins`". A value of "`=`" indicates that you must consult the reference for the sequence; this shorthand is only used in regions where no allele deviates from the reference sequence.
9	alleleSeq	The observed sequence at the locus of variation. Empty when *varType* is `del`. Question mark (`?`) indicates zero or more unknown bases within the sequence. "`N`" indicates exactly one unknown base within the sequence. Equal sign (`=`) is used as shorthand to indicate identity to the reference sequence for non-variant sequence, such as when *varType* is `ref`.
10	varScoreVAF	Positive integer representing confidence in the call. It is derived from the probability estimates under maximum likelihood variable allele fraction. Specifically, it is equal to $$10 * \log 10\left(\frac{\text{P(Call is true)}}{\text{P(Call is false)}}\right)$$ This field is empty for reference calls or no-calls.
11	varScoreEAF	Positive or negative integer representing confidence in the call. It is derived from the probability estimates under equal allele fraction model. Specifically, it is equal to $$10 * \log 10\left(\frac{\text{P(Call is true)}}{\text{P(Call is false)}}\right)$$ This field is empty for reference calls or no-calls.
12	varFilter	List of indicators of low-quality or incomplete resolution of the sequence of this call. If "PASS", then the call passes all relevant quality tests. Otherwise, the list includes one or more semicolon-separated values from the following possible filters:<br>▪ `VQLOW` — indicates the call is homozygous and *varScoreVAF* is less than 20 dB, or the call is not homozygous and *varScoreVAF* is less than 40 dB.<br>▪ `AMBIGUOUS` — for homozygous calls, indicates that there was another non-reference hypothesized sequence that scored within 10 dB of the primary non-reference call; for heterozygous calls, indicates that there was another non-reference hypothesized sequence that scored within 20 dB of the primary non-reference call.
13	hapLink	Identifier that links an allele at one locus to alleles at other loci. Currently this field is only populated for very proximate variations that were either assembled together or were determined to be in phase using a correlation-based analysis between two variation intervals one mate pair away. Calls that share a hapLink identifier are expected to be on the same haplotype. Calls with haplinks appearing only once in the file and calls with no haplinks can be interpreted similarly: there is no phasing information with any other loci.
14	xRef	Semicolon-separated list of external variation identifiers, populated for variations corroborated directly by external sources. Currently used sources are dbSNP and COSMIC. Multiple entries for the same source (e.g., `dbsnp`) correspond to multiple matches between the variant and records from the source. Supported types of matches include one call to one record, one call to a combination of overlapping records, and a combination of several consecutive calls matching exactly one record.<br><br>Format for dbSNP: `dbsnp.<build>:<rsID>` where `<build>` indicates the version of dbSNP where the record first appeared. For example, "`dbsnp.129:rs12345`".<br><br>Format for COSMIC: `COSMIC.<type>:identifier` where `<type>` indicates COSMIC classification of somatic variants. For example, for a non-coding variant, *xRef* would contain "`COSMIC:ncv_id:139111`".

<table>
<tr><th></th><th>Column Name</th><th>Description</th></tr>
<tr><td>15</td><td>alleleFreq</td><td>
Allele frequency value(s) for the entire call or for parts of the call that are corroborated directly by external sources. The source is 1000 Genomes Project minor allele frequency information in dbSNP.
Format is <code><source>:<frequency></code>, with multiple pairs separated by a semicolon. Precision of <code>frequency</code> is three decimal places.
Format for dbSNP becomes <code>dbsnp:<frequency></code>. Multiple entries for the same type of source mirror the multiple entries for this source appearing in xRef.
If an allele frequency value is not available for a dbSNP record, the corresponding position in the alleleFreq column is left empty.
<table>
<tr><th>When the call matches...</th><th>The alleleFreq field shows...</th></tr>
<tr><td>1 rsID with known frequency.</td><td><code>dbsnp:0.234</code></td></tr>
<tr><td>1 rsID with unknown frequency.</td><td>(empty string)</td></tr>
<tr><td>2 rsIDs (independently or as a combination) with both known frequencies.</td><td><code>dbsnp:0.234;dbsnp:0.123</code></td></tr>
<tr><td>2 rsIDs with both unknown frequencies.</td><td><code>;</code> (a single semicolon)</td></tr>
<tr><td>3 rsIDs with 1 known and 2 unknown frequencies.</td><td>Depending on which of the frequencies are unknown, one of the following:
<code>dbsnp:0.234;;</code>
<code>;dbsnp:0.234;</code>
<code>;;dbsnp:0.234</code></td></tr>
</table>
</td></tr>
<tr><td>16</td><td>alternativeCalls</td><td>Contains alternate calls for alleles designated “<code>AMBIGUOUS</code>”. Formatted as a semicolon-separated list of <code><sequence>:<score></code> pairs, where <code><sequence></code> is a hypothesized nucleotide sequence, and <code><score></code> is the score of that hypothesized sequence, relative to the called sequence. For example, if alternativeCalls is “<code>AG:-1;G:-8</code>”, then sequence AG scored 1 dB less than the called sequence and G scored 8 dB less than the called sequence.</td></tr>
</table>

Master Variations

ASM/masterVarBeta-[ASM-ID].tsv.bz2

The master variations file is a simple, integrated report of the variant calls and annotation information produced by the Complete Genomics assembly process. The file format is derived heavily from the variations file format and has the following important features:

- The format includes one line for any given locus of the genome. The allele sequence is a concatenation of all calls from the variations file for the given allele. As a result, in some complex loci, the information about the exact alignment of the called sequence to the reference may be lost.

  Also note that allele IDs are not conserved between the ***var*** and ***masterVar*** files. Thus at a given locus allele 1 in the var file can be called allele 1 or allele 2 in ***masterVar***.

- Just as the information about alignment of call sequence is lost when concatenating calls of the ***var*** file to produce the ***masterVarBeta*** file, so also is call-specific annotation information. For example, functional impact information from the gene file that relates to a call is combined to produce the annotation within the ***masterVar*** file. However, in most cases, the association is obvious.
- Certain simple variant calls embedded in more complex loci may not be as easy to identify in the ***masterVarBeta*** file format compared to the variations file. For example, a locus that contains a SNP opposite a two-base substitution will be classified as “complex” after the conversion.
- The format integrates annotation information from other Complete Genomics data files. For example, loci are annotated with read counts from the evidence files and with copy number calls from the CNV result files.
- For every locus line, the *zygosity* field can be used to quickly determine if the locus is fully called on one, both, or none of the alleles. Fully called loci are further classified into haploid, homozygous, heterozygous reference (where one of the alleles is equal to the reference), and heterozygous alternate (where neither of the alleles is equal to the reference).
- Loci that contain simple isolated variations (SNP, INS, DEL or SUB) can be easily identified using the *varType* field.
- The format provides a structured content not found in the ***Var*** file that can easily be converted into other standard variation file formats.

Example — *ASM/masterVarBeta-[ASM-ID].tsv.bz2*

The data is broken into three sections to show all the columns. The second and third sections of data repeat the *locus* column at the left edge to more easily match the data between sections; the *locus* column is not repeated in the actual data.

© Complete Genomics, Inc.

>locus	ploidy	chromosome	begin	end	zygosity	varType	reference	allele1Seq	allele2Seq	allele1VarScoreVAF	allele2VarScoreVAF	allele1VarScoreEAF	allele2VarScoreEAF
15143	2	chr1	857653	857727	hom	ref	=	=	=				
15144	2	chr1	857727	857728	hom	snp	T	G	G	71	588	71	588
15145	2	chr1	857728	857829	hom	ref	=	=	=				
15146	2	chr1	857829	857829	het-ref	ins		GAGA		314	563	327	563
15147	2	chr1	857829	858691	hom	ref	=	=	=				
15148	2	chr1	858691	858692	hom	del	G			644	62	644	62
15149	2	chr1	858692	858800	hom	ref	=	=	=				
15150	2	chr1	858800	858801	hom	snp	A	G	G	28	186	28	186

>locus	allele1VarFilter	allele2VarFilter	allele1HapLink	allele2HapLink	allele1XRef	allele2XRef	allele1Freq	allele2Freq	allele1AlternativeCalls	allele2AlternativeCalls	evidenceIntervalId	allele1ReadCount	allele2ReadCount	referenceAlleleReadCount	totalReadCount
15143															
15144	PASS	PASS			dbsnp.116:rs6689107	dbsnp.116:rs6689107					5921	29	29	0	29
15145															
15146	PASS	PASS									5922	23	26	26	49
15147															
15148	PASS	PASS			dbsnp.126:rs34628185; dbsnp.132:rs112938754; dbsnp.137:rs200483463	dbsnp.126:rs34628185; dbsnp.132:rs112938754; dbsnp.137:rs200483463	;;	;;			5923	36	36	1	37
15149															
15150	PASS	PASS			dbsnp.116:rs7418179	dbsnp.116:rs7418179					5924	12	12	0	12

>locus	allele1Gene	allele2Gene	pfam	miRBaseId	repeatMasker	segDupOverlap	relativeCoverageDiploid	calledPloidy	relativeCoverageNondiploid	calledLevel	bestLAFsingle	lowLAFsingle	highLAFsingle
15143							1.01	2	1	1.001	0.42	0.41	0.45
15144	100130417:NR_026874.1: FLJ39609:TSS-UPSTREAM: UNKNOWN-INC;…	100130417:NR_026874.1: FLJ39609:TSS-UPSTREAM: UNKNOWN-INC;…					1.01	2	1	1.001	0.42	0.41	0.45
15145							1.01	2	1	1.001	0.42	0.41	0.45
15146	100130417:NR_026874.1: FLJ39609:TSS-UPSTREAM: UNKNOWN-INC;…	100130417:NR_026874.1: FLJ39609:TSS-UPSTREAM:UNKNOWN-INC;…					1.01	2	1	1.001	0.42	0.41	0.45
15147							1.01	2	1	1.001	0.42	0.41	0.45
15148	100130417:NR_026874.1: FLJ39609:TSS-UPSTREAM: UNKNOWN-INC;…	100130417:NR_026874.1: FLJ39609:TSS-UPSTREAM:UNKNOWN-INC;…					1.01	2	1	1.001	0.42	0.41	0.45
15149							1.01	2	1	1.001	0.42	0.41	0.45
15150	100130417:NR_026874.1: FLJ39609:TSS-UPSTREAM: UNKNOWN-INC;…	100130417:NR_026874.1: FLJ39609:TSS-UPSTREAM:UNKNOWN-INC;…			MIR:MIR:30.2		1.01	2	1	1.001	0.42	0.41	0.45

File-Specific Header Description — *ASM/masterVarBeta-[ASM-ID].tsv.bz2*

Key	Description	Allowed Values
#ASSEMBLY_ID	Name of the assembly	"<assembly-name>-ASM". For example, "GS000000474-ASM".
#CNV_DIPLOID_WINDOW_WIDTH	Width, in bases, of windows in which smoothed coverage is calculated for copy number estimation	Positive integer. For example, 2000.
#CNV_NONDIPLOID_WINDOW_WIDTH	Width, in bases, of windows in which smoothed coverage is calculated for coverage level estimation	Positive integer. For example, 10000.
#COSMIC	COSMIC version used for annotation	"COSMIC vXX", where X's are digits. For example "COSMIC v48".
#DBSNP_BUILD	dbSNP version used for annotation	"dbSNP build XXX" where X's are digits. For example, "dbSNP build 130".
#FORMAT_VERSION	Version number of the file format	Two or more digits separated by periods. For example, "0.6".
#GENERATED_AT	Date and time of the assembly	Year-Month-Day Time. For example "2010-Sep-08 20:27:52.457773".
#GENERATED_BY	Assembly pipeline component that generated the output	Alpha-numeric string.
#GENE_ANNOTATIONS	NCBI annotation build	"NCBI build XX.X" where X's are digits.

Key	Description	Allowed Values
#GENOME_REFERENCE	Human genome build used for assembly	"NCBI build XX" where X's are digits.
#MIRBASE_VERSION	miRBase version used for annotation	"miRBase build XX" where X's are digits.
#PFAM_DATE	Date on which Pfam information was downloaded from NCBI Conserved Domain Database	Day-Month-Year. For example "13-Aug-10".
#REPMASK_GENERATED_AT	Date and time on which repeat masker information was downloaded from the UCSC genome browser website	Year-Month-Day Time. For example "2010-Sep-08 20:27:52.457773".
#SAMPLE	Complete Genomics identifier of the sample from which the library was created	"GSXXXXX-DNA_YZZ" where<br>▪ X's are digits<br>▪ -DNA_ is literal<br>▪ YZZ is the location of the sample in a 96-well plate with Y as one of "A" through "H" and ZZ is one of "01" through "12"<br>For example "GS12345-DNA_A01".
#SEGDUP_GENERATED_AT	Date and time on which segmental duplication information was downloaded from the UCSC genome browser website	Year-Month-Day Time. For example "2010-Sep-08 20:27:52.457773".
#SOFTWARE_VERSION	Assembly pipeline build number	Two or more digits separated by periods.
#TYPE	Indicates the type of data contained in the file.	"VAR-OLPL"

Content Description *ASM/masterVarBeta-[ASM-ID].tsv.bz2*

	Column Name	Description
1	locus	Integer ID of the locus. When converting a Complete Genomics variant file, all loci will retain the original IDs. When processing filtered files where regions have been removed, the loci that correspond to the removed regions are recreated with a locus ID 0 and are considered fully no-called.
2	ploidy	Number of alleles (same as in the Complete Genomics variations file).
3	chromosome	Chromosome name (same as in the Complete Genomics variations file).
4	begin	Locus start. Zero-based offset of the first base in the locus, the same as in the Complete Genomics variations file.
5	end	Locus end. Zero-based offset of the first base downstream of the locus, same as in the Complete Genomics variations file.
6	zygosity	Call completeness and zygosity information. *zygosity* is assigned one of the following values:<br>▪ `no-call`: All alleles are partially or fully no-called.<br>▪ `hap`: Haploid, fully called locus.<br>▪ `half`: Diploid locus where one of the alleles is fully called and the other contains no-calls.<br>▪ `hom`: Diploid, homozygous, fully called locus.<br>▪ `het-ref`: Diploid, heterozygous, fully called locus where one of the alleles is identical to the reference.<br>▪ `het-alt`: Diploid, heterozygous, fully called locus where both alleles differ from the reference.

	Column Name	Description
7	varType	Variation type for simple, isolated variations. *varType* is assigned one of the following values:<br>▪ `snp`, `ins`, `del`, or `sub`: Fully called or half-called locus that contains only a single isolated variation.<br>▪ `ref`: Fully called or half-called locus that contains only reference calls and no calls and at least one allele is fully called.<br>▪ `complex`: Locus that contains multiple variations or has no-calls in all alleles. This is also the value for all loci where the reference itself is ambiguous.<br>▪ `no-ref`: Locus where the reference genome is N.<br>▪ `PAR-called-in-X`: Locus on the pseudo-autosomal region of the Y chromosomes in males.
8	reference	Reference sequence. Loci called as homozygous reference and loci that are fully no-called on all alleles will contain "=" instead of the literal reference sequence.
9	allele1Seq	Sequence of the first allele. May contain N and ? characters that represent one-base no-calls and unknown length no-calls, respectively, with the same semantics as used for "*alleleSeq*" in the Complete Genomics variant file.<br>The field is empty when the called variant is a deletion of all bases in the locus. For a given locus, if the allele in the variation file spans multiple lines, then the sequences for each call corresponding that the allele are concatenated.
10	allele2Seq	Sequence of the second allele. The value of *allele2Seq* follows the same rules as *allele1Seq*. This field is always blank for haploid loci (whenever the ploidy field contains `1`).<br>The values of *allele1Seq* and *allele2Seq* are assigned such that a variation allele always precedes a pure reference allele, and a fully called allele always precedes any allele that contains no-calls. As a result, the allele order may differ from the order in the corresponding source variations file.
11	allele1VarScoreVAF	Positive integer representing confidence in the call for the first allele. It is derived from the probability estimates under maximum likelihood variable allele fraction. Specifically, it is equal to<br>$$10 * \log 10 \left(\frac{\text{P(Call is true)}}{\text{P(Call is false)}} \right)$$<br>This field is empty for reference calls or no-calls.
12	allele2VarScoreVAF	Positive integer representing confidence in the call for the second allele. It is derived from the probability estimates under maximum likelihood variable allele fraction. Specifically, it is equal to<br>$$10 * \log 10 \left(\frac{\text{P(Call is true)}}{\text{P(Call is false)}} \right)$$<br>This field is empty for reference calls or no-calls.
13	allele1VarScoreEAF	Positive or negative integer representing confidence in the call for the first allele. It is derived from the probability estimates under equal allele fraction model. Specifically, it is equal to<br>$$10 * \log 10 \left(\frac{\text{P(Call is true)}}{\text{P(Call is false)}} \right)$$<br>This field is empty for reference calls or no-calls.
14	allele2VarScoreEAF	Positive or negative integer representing confidence in the call for the second allele. It is derived from the probability estimates under equal allele fraction model. Specifically, it is equal to<br>$$10 * \log 10 \left(\frac{\text{P(Call is true)}}{\text{P(Call is false)}} \right)$$<br>This field is empty for reference calls or no-calls.

	Column Name	Description
15	allele1VarFilter	List of indicators of low-quality or incomplete resolution of the sequence of allele 1. If "PASS", then the allele passes all relevant quality tests. Otherwise the list includes one or more semicolon-separated values, from the following possible filters:<br>▪ `VQLOW` — indicates the call is homozygous and *allele1VarScoreVAF* is less than 20 dB, or the call is not homozygous and *allele1VarScoreVAF* is less than 40 dB.<br>▪ `AMBIGUOUS` — for homozygous non-reference alleles, indicates there was another non-reference hypothesized sequence that scored within 10 dB of the call; for heterozygous non-reference alleles, indicates there was another non-reference hypothesized sequence that scored within 20 dB of the call.
16	allele2VarFilter	List of indicators of low-quality or incomplete resolution of the sequence of allele 2. Values as described for *allele1VarFilter*.
17	allele1HapLink	Integer ID that links the first allele to the alleles of other loci that are known to reside on the same haplotype.
18	allele2HapLink	Integer ID that links the second allele to the alleles of other loci that are known to reside on the same haplotype.
19	allele1XRef	Semicolon-separated list of external variation identifiers for allele 1, populated for variations corroborated directly by external sources. Currently used sources are dbSNP and COSMIC. Multiple entries for the same source (e.g., `dbsnp`) correspond to multiple matches between the variant and records from the source. Supported types of matches include one call to one record, one call to a combination of overlapping records, and a combination of several consecutive calls matching exactly one record.<br><br>Format for dbSNP: `dbsnp.<build>:<rsID>` where `<build>` indicates the version of dbSNP where the record first appeared. For example, "`dbsnp.129:rs12345`".<br><br>Format for COSMIC: `COSMIC.<type>:identifier` where `<type>` indicates COSMIC classification of somatic variants. For example for a non-coding variant, *allele1XRef* would contain "`COSMIC:ncv_id:139111`".
20	allele2XRef	Semicolon-separated list of external variation identifiers for allele 2. Values and formatting as described for *allele1XRef*.

<table>
<tr><th></th><th>Column Name</th><th>Description</th></tr>
<tr><td>21</td><td>allele1Freq</td><td>Allele 1 frequency value(s) for the entire call or for parts of the call that are corroborated directly by external sources. The source is 1000 Genomes Project minor allele frequency information in dbSNP.

Format is <source>:<frequency>, with multiple entries separated by the semicolon (;). Precision of frequency is three decimal places.

Format for dbSNP becomes dbsnp:<frequency>. Multiple entries for the same source mirror the multiple entries for this source appearing in *allele1xRef*. If an allele frequency value is not available for a dbSNP record, the corresponding position in the *allele1Freq* column is left empty.

<table>
<tr><th>When the call matches...</th><th>The *alleleFreq* field shows...</th></tr>
<tr><td>1 rsID with known frequency.</td><td>dbsnp:0.234</td></tr>
<tr><td>1 rsID with unknown frequency.</td><td>(empty string)</td></tr>
<tr><td>2 rsIDs (independently or as a combination) with both known frequencies.</td><td>dbsnp:0.234;dbsnp:0.123</td></tr>
<tr><td>2 rsIDs with both unknown frequencies.</td><td>; (a single semicolon)</td></tr>
<tr><td>3 rsIDs with 1 known and 2 unknown frequencies.</td><td>Depending on which of the frequencies are unknown, one of the following:

dbsnp:0.234;;
;dbsnp:0.234;
;;dbsnp:0.234</td></tr>
</table>
</td></tr>
<tr><td>22</td><td>allele2Freq</td><td>Allele 2 frequency value(s) for the entire call or for parts of the call that are corroborated directly by external sources. Values and formatting as described for *allele1Freq*.</td></tr>
<tr><td>23</td><td>allele1AlternativeCalls</td><td>Alternate calls for allele 1 if the primary call is designated “AMBIGUOUS”. Formatted as a semicolon-separated list of <sequence>:<score> pairs, where <sequence> is a hypothesized nucleotide sequence, and <score> is the score of that hypothesized sequence, relative to the called sequence. For example, if *allele1AlternativeCalls* is “AG:-1;G:-8”, then sequence AG scored 1 dB less than the called sequence, and G scored 8 dB less than the called sequence.</td></tr>
<tr><td>24</td><td>allele2AlternativeCalls</td><td>Alternate calls for allele 2 if the primary call is designated “AMBIGUOUS”. Values and formatting as described for *allele1AlternativeCalls*.</td></tr>
<tr><td>25</td><td>evidenceIntervalId</td><td>Integer ID of the interval in the evidence file. Multiple loci may share the same evidence interval.</td></tr>
<tr><td>26</td><td>allele1ReadCount</td><td>Number of reads that support the first allele. A read is included in the count if it overlaps the locus interval and supports the allele by at least 3 dB more than the other allele or the reference.

For length-preserving variations, at least one base in the read must overlap the interval to be included in the read count.</td></tr>
<tr><td>27</td><td>allele2ReadCount</td><td>Number of reads that support the second allele. For homozygous loci, this number is identical to *allele1ReadCount*.</td></tr>
<tr><td>28</td><td>referenceAlleleReadCount</td><td>Number of reads that support the reference sequence. For loci where one of the alleles is reference, this number is identical to the read count of that allele.</td></tr>
</table>

	Column Name	Description
29	totalReadCount	Total number of reads in the evidence file that overlap the interval. Note that this count also includes reads that do not strongly support one allele over the other and consequently are not accounted for in *allele1ReadCount* or *allele2ReadCount*.<br>For loci where one of the alleles contains a no-call, the *totalReadCount* also includes the reads that support that no-called allele. The *totalReadCount* does not include reads that do not overlap the locus, even if they do overlap the evidence interval, and, hence, are present in the evidence file.
30	allele1Gene	Semicolon-separated list of all gene annotations for the first allele of the locus. For every gene annotation, the following fields from the ***gene*** file are concatenated together using colon as separator: *geneId*, *mrnaAcc*, *symbol*, *component*, and *impact*.<br>For example:<br>`100130417:NR_026874.1:FLJ39609:TSS-UPSTREAM:UNKNOWN-INC;148398:NM_152486.2:SAMD11:TSS-UPSTREAM:UNKNOWN-INC`
31	allele2Gene	Gene annotation list for the second allele formatted in the same way as *allele1Gene*.
32	pfam	Pfam domain information that overlap with the locus.
33	miRBaseId	Semicolon-separated list of all ncRNA annotations for this locus.
34	repeatMasker	Semicolon-separated list of all RepeatMasker records that overlap this locus. Within each record, the following data is concatenated together using colon as the separator:<br>▪ repeat name<br>▪ repeat family<br>▪ overall divergence percentage (number of bases changed, deleted, or inserted relative to the repeat consensus sequence per hundred bases)<br>Mitochondrion loci are not annotated.<br>See RepeatMasker in "References" for more information.
35	segDupOverlap	Number of distinct segmental duplications that overlap this locus.
36	relativeCoverageDiploid	Normalized coverage level, under a diploid model, for the segment that overlaps the current locus (for loci that overlap two segments, the data from the ***cnvSegmentsDiploidBeta*** file with the longer overlap are chosen). This column corresponds to the *relativeCvg* field in the ***cnvSegmentsDiploidBeta*** file.
37	calledPloidy	Ploidy of the segment, as called using a diploid model. Only present if the ploidy calls were made during the assembly (only when the *calledPloidy* column is present in the source ***cnvSegmentsDiploidBeta*** file). This column corresponds to the *calledPloidy* field in the ***cnvSegmentsDiploidBeta*** file.
38	relativeCoverageNondiploid	Normalized coverage level, under a nondiploid model, for the segment that overlaps the current locus (for loci that overlap two segments, the data from the ***cnvSegmentsNondiploidBeta*** file with the longer overlap are chosen). This column corresponds to the *relativeCvg* field in the ***cnvSegmentsNondiploidBeta*** file.
39	calledLevel	Coverage level of the segment, as called using a non-diploid model. Only present if the ploidy coverage levels were made during the assembly (only when the *calledLevel* column is present in the source ***cnvSegmentsNondiploidBeta*** file). This column corresponds to the *calledLevel* field in the ***cnvSegmentsNondiploidBeta*** file.
40	bestLAFsingle	Single-sample maximum likelihood estimate of Lesser Allele Fraction (LAF) of the overlapping 100 kb CNV analysis window spanning the locus. This column corresponds to the *bestLAFsingle* column in the ***cnvDetailsNondiploidBeta**** file.
41	lowLAFsingle	Minimum value of the approximate 99% confidence interval around the *bestLAFsingle*. This column corresponds to the *lowLAFsingle* column in the ***cnvDetailsNondiploidBeta**** file.
42	highLAFsingle	Maximum value of the approximate 99% confidence interval around the *bestLAFsingle*. This column corresponds to the *highLAFsingle* column in the ***cnvDetailsNondiploidBeta**** file.

Individual Genomes' Small Variations, CNVs, SVs, and MEIs in VCF Format

ASM/vcfBeta-[ASM-ID].vcf.bz2

The ***vcfBeta-[ASM-ID].vcf.bz2*** file contains the small variant, copy number variation (CNV), structural variation (SV), and mobile element insertion (MEI) calls made by the Complete Genomics Assembly Pipeline for a single genome (either a normal genome or a tumor). It conforms to the VCF 4.1 specification. Characteristics of the file to note:

- The file integrates information such as scores, annotations, and coverage for all called variants including small variants (SNPs, indels and substitutions), CNVs, SVs, and MEIs.
- The sample column represents the genome with the sample header ID corresponding to the assembly ID of the genome.
- Non-reserved words in the ALT, INFO, and FORMAT fields of the VCF use a "CGA_" prefix to ensure there is no conflict in future usage of standard sub-field names or other non-standard sub-field names. The FILTER field does not use the "CGA_" prefix.

The explanation of the data format for the ***vcfBeta*** file is broken into the following sections:

- Example
- Meta-Information Description
- Header Line Description
- Content Description, which is further divided into:
  - Small Variant Data in VCF
  - CNVs in VCF
  - SVs in VCF
  - MEIs in VCF

Example — *ASM/vcfBeta-[ASM-ID].vcf.bz2*

This example shows the contents of the file in sections, including:

- Figure 4: Meta-information: each line starts with a ## string, and often includes "key=value" statements.
- Figure 5: Header with the seven of the eight mandatory columns (#CHROM, POS, ID, REF, ALT, QUAL, FILTER).
- Figure 7 continued: Header with the remaining mandatory columns followed by the format and the sample column (INFO, FORMAT, genome's ASM-ID).

Data removed for brevity is marked with ellipses.

Figure 4: *vcfBeta-[ASM-ID].vcf.bz2* File: Meta Information

```
##fileformat=VCFv4.1
##fileDate=20120620
##center=Complete Genomics
##source=CGAPipeline_2.2.0.16
##source_GENOME_REFERENCE=NCBI build 37
##source_MAX_PLOIDY=10
##source_NUMBER_LEVELS=GS00028-DNA_C01:8
##source_NONDIPLOID_WINDOW_WIDTH=100000
##source_MEAN_GC_CORRECTED_CVG=GS00028-DNA_C01:45.50
```

```
##source_GENE_ANNOTATIONS=NCBI build 37.2
##source_DBSNP_BUILD=dbSNP build 135
##source_MEI_1000G_ANNOTATIONS=INITIAL-DATA-RELEASE
##source_COSMIC=COSMIC v59
##source_MIRBASE_VERSION=mirBase version 18
##source_PFAM_DATE=April 21, 2011
##source_REPMASK_GENERATED_AT=2011-Feb-15 10:08
##source_SEGDUP_GENERATED_AT=2010-Dec-01 13:40
##phasing=partial
##reference=ftp://ftp.completegenomics.com/ReferenceFiles/build37.fa.bz2
##contig=<ID=1,length=249250621,assembly=B37,md5=1b22b98cdeb4a9304cb5d48026a85128,species="Homo sapiens">
##contig=<ID=2,length=243199373,assembly=B37,md5=a0d9851da00400dec1098a9255ac712e,species="Homo sapiens">
. . .
##ALT=<ID=CGA_CNVWIN,Description="Copy number analysis window">
##ALT=<ID=INS:ME:ALU,Description="Insertion of ALU element">
##ALT=<ID=INS:ME:L1,Description="Insertion of L1 element">
##ALT=<ID=INS:ME:SVA,Description="Insertion of SVA element">
##ALT=<ID=INS:ME:MER,Description="Insertion of MER element">
##ALT=<ID=INS:ME:LTR,Description="Insertion of LTR element">
##ALT=<ID=INS:ME:PolyA,Description="Insertion of PolyA element">
##ALT=<ID=INS:ME:HERV,Description="Insertion of HERV element">
##ALT=<ID=CGA_NOCALL,Description="No-called record">
##FILTER=<ID=URR,Description="Too close to an underrepresented repeat">
##FILTER=<ID=MPCBT,Description="Mate pair count below 10">
##FILTER=<ID=SHORT,Description="Junction side length below 70">
##FILTER=<ID=TSNR,Description="Transition sequence not resolved">
##FILTER=<ID=INTERBL,Description="Interchromosomal junction in baseline">
##FILTER=<ID=sns75,Description="Sensitivity to known MEI calls in range (.75,.95] i.e. medium FDR">
##FILTER=<ID=sns95,Description="Sensitivity to known MEI calls in range (.95,1.00] i.e. high to very high FDR">
##FILTER=<ID=VQLOW,Description="Indicates the call is homozygous and the varScoreVAF is less than 20 dB, or the call is not
homozygous and the varScoreVAF is less than 40 dB">
##FILTER=<ID=SQLOW,Description="Indicates somaticScore < -10 for somatic variant">
##FILTER=<ID=FET30,Description="Indicates fisherSomatic < 30 for somatic variant">
##FILTER=<ID=AMBIGUOUS,Description="Indicates that read evidence does not strongly distinguish multiple non-reference
candidate alleles">
##INFO=<ID=NS,Number=1,Type=Integer,Description="Number of Samples With Data">
##INFO=<ID=CGA_WINEND,Number=1,Type=Integer,Description="End of coverage window">
##INFO=<ID=CGA_BF,Number=1,Type=Float,Description="Frequency in baseline">
##INFO=<ID=CGA_MEDEL,Number=4,Type=String,Description="Consistent with deletion of mobile element;
type,chromosome,start,end">
##INFO=<ID=CGA_XR,Number=A,Type=String,Description="Per-ALT external database reference (dbSNP, COSMIC, etc)">
##INFO=<ID=MATEID,Number=1,Type=String,Description="ID of mate breakend">
##INFO=<ID=SVTYPE,Number=1,Type=String,Description="Type of structural variant">
##INFO=<ID=CGA_BNDG,Number=A,Type=String,Description="Transcript name and strand of genes containing breakend">
##INFO=<ID=CGA_BNDGO,Number=A,Type=String,Description="Transcript name and strand of genes containing mate breakend">
##INFO=<ID=CIPOS,Number=2,Type=Integer,Description="Confidence interval around POS for imprecise variants">
##INFO=<ID=END,Number=1,Type=Integer,Description="End position of the variant described in this record">
##INFO=<ID=IMPRECISE,Number=0,Type=Flag,Description="Imprecise structural variation">
##INFO=<ID=MEINFO,Number=4,Type=String,Description="Mobile element info of the form NAME,START,END,POLARITY">
##INFO=<ID=SVLEN,Number=.,Type=Integer,Description="Difference in length between REF and ALT alleles">
##INFO=<ID=AN,Number=1,Type=Integer,Description="Total number of alleles in called genotypes">
##INFO=<ID=AC,Number=A,Type=Integer,Description="Allele count in genotypes, for each ALT allele">
##INFO=<ID=AF,Number=A,Type=String,Description="Allele frequency, or &-separated frequencies for complex variants (in
latter, '?' designates unknown parts)">
##INFO=<ID=CGA_FI,Number=A,Type=String,Description="Functional impact annotation">
##INFO=<ID=CGA_PFAM,Number=.,Type=String,Description="PFAM Domain">
```

```
##INFO=<ID=CGA_MIRB,Number=.,Type=String,Description="miRBaseId">
##INFO=<ID=CGA_RPT,Number=.,Type=String,Description="repeatMasker overlap information">
##INFO=<ID=CGA_SDO,Number=1,Type=Integer,Description="Number of distinct segmental duplications that overlap this locus">
##FORMAT=<ID=GT,Number=1,Type=String,Description="Genotype">
##FORMAT=<ID=CGA_GP,Number=1,Type=Float,Description="Depth of coverage for 2k window GC normalized to mean">
##FORMAT=<ID=CGA_NP,Number=1,Type=Float,Description="Coverage for 2k window, GC-corrected and normalized relative to
copy-number-corrected multi-sample baseline">
##FORMAT=<ID=CGA_CL,Number=1,Type=Float,Description="Nondiploid-model called level">
##FORMAT=<ID=CGA_LS,Number=1,Type=Integer,Description="Nondiploid-model called level score">
##FORMAT=<ID=CGA_CP,Number=1,Type=Integer,Description="Diploid-model called ploidy">
##FORMAT=<ID=CGA_PS,Number=1,Type=Integer,Description="Diploid-model called ploidy score">
##FORMAT=<ID=CGA_CT,Number=1,Type=String,Description="Diploid-model CNV type">
##FORMAT=<ID=CGA_TS,Number=1,Type=Integer,Description="Diploid-model CNV type score">
##FORMAT=<ID=CGA_LAFS,Number=1,Type=Float,Description="Lesser allele fraction estimate, single-sample analysis, 100000bp
window">
##FORMAT=<ID=CGA_LLAFS,Number=1,Type=Float,Description="Lesser allele fraction lower bound, single-sample analysis,
100000bp window">
##FORMAT=<ID=CGA_ULAFS,Number=1,Type=Float,Description="Lesser allele fraction upper bound, single-sample analysis,
100000bp window">
##FORMAT=<ID=FT,Number=1,Type=String,Description="Genotype filters">
##FORMAT=<ID=CGA_BNDMPC,Number=1,Type=Integer,Description="Mate pair count supporting breakend">
##FORMAT=<ID=CGA_BNDPOS,Number=1,Type=Integer,Description="Breakend position">
##FORMAT=<ID=CGA_BNDDEF,Number=1,Type=String,Description="Breakend definition">
##FORMAT=<ID=CGA_BNDP,Number=1,Type=String,Description="Precision of breakend">
##FORMAT=<ID=CGA_IS,Number=1,Type=Float,Description="MEI InsertionScore: confidence in occurrence of an insertion">
##FORMAT=<ID=CGA_IDC,Number=1,Type=Float,Description="MEI InsertionDnbCount: count of paired ends supporting insertion">
##FORMAT=<ID=CGA_IDCL,Number=1,Type=Float,Description="MEI InsertionLeftDnbCount: count of paired ends supporting
insertion on 5' end of insertion point">
##FORMAT=<ID=CGA_IDCR,Number=1,Type=Float,Description="MEI InsertionRightDnbCount: count of paired ends supporting
insertion on 3' end of insertion point">
##FORMAT=<ID=CGA_RDC,Number=1,Type=Integer,Description="MEI ReferenceDnbCount: count of paired ends supporting
reference allele">
##FORMAT=<ID=CGA_NBET,Number=1,Type=String,Description="MEI NextBestElementType: (sub)type of second-most-likely
inserted mobile element">
##FORMAT=<ID=CGA_ETS,Number=1,Type=Float,Description="MEI ElementTypeScore: confidence that insertion is of type
indicated by CGA_ET/ElementType">
##FORMAT=<ID=CGA_KES,Number=1,Type=Float,Description="MEI KnownEventSensitivityForInsertionScore: fraction of known
MEI insertion polymorphisms called for this sample with CGA_IS at least as high as for the current call">
##FORMAT=<ID=PS,Number=1,Type=Integer,Description="Phase Set">
##FORMAT=<ID=SS,Number=1,Type=String,Description="Somatic Status: Germline, Somatic, LOH, or . (Unknown)">
##FORMAT=<ID=CGA_ALTCALLS,Number=2,Type=String,Description="Alternative call sequences and scores">
##FORMAT=<ID=GQ,Number=1,Type=Integer,Description="Genotype Quality">
##FORMAT=<ID=HQ,Number=2,Type=Integer,Description="Haplotype Quality">
##FORMAT=<ID=EHQ,Number=2,Type=Integer,Description="Haplotype Quality, Equal Allele Fraction Assumption">
##FORMAT=<ID=CGA_CEHQ,Number=2,Type=Integer,Description="Calibrated Haplotype Quality, Equal Allele Fraction
Assumption">
##FORMAT=<ID=GL,Number=.,Type=Integer,Description="Genotype Likelihood">
##FORMAT=<ID=CGA_CEGL,Number=.,Type=Integer,Description="Calibrated Genotype Likelihood, Equal Allele Fraction
Asssumption">
##FORMAT=<ID=DP,Number=1,Type=Integer,Description="Total Read Depth">
##FORMAT=<ID=AD,Number=2,Type=Integer,Description="Allelic depths (number of reads in each observed allele)">
##FORMAT=<ID=CGA_RDP,Number=1,Type=Integer,Description="Number of reads observed supporting the reference allele">
```

Figure 5: *vcfBeta-[ASM-ID].vcf.bz2* File: Header with Seven of Eight Mandatory Data Columns

#CHROM	POS	ID	REF	ALT	QUAL	FILTER
1	1032964	.	A	.	.	.
…	…	…	…	…	…	…
1	1034001	.	T	<CGA_CNVWIN>	.	.
1	1034137	.	G	<INS:ME:ALU>	.	.
1	1034141	.	G	A	.	.
…	…	…	…	…	…	…
1	1046746	.	AAAA	A	.	.
…	…	…	…	…	…	…
1	1050858	.	C	<CGA_NOCALL>	.	.
…	…	…	…	…	…	…
1	1070131	NA19240female37_4994_L	G	]1:1070188]G	.	.
1	1070188	NA19240female37_4994_R	T	T[1:1070131[	.	.

Figure 5 continued: *vcfBeta-[ASM-ID].vcf.bz2* File: Header with Eighth Mandatory Data Column (INFO) and Two Additional Columns (FORMAT and Sample)

INFO	FORMAT	NA19240-37-240-ASM
NS=1;AN=0	GT:PS	./.:.
…	…	…
NS=1;CGA_WINEND=1036000	GT:CGA_GP:CGA_NP:CGA_CP:CGA_PS:CGA_CT:CGA_LAFS:CGA_LLAFS:CGA_ULAFS:CGA_TS:CGA_CL:CGA_LS	.:1.70:1.06:2:56:=:0.46:0.44:0.5:56:1.004:879
IMPRECISE;SVTYPE=INS;END=1034137;SVLEN=39;CIPOS=-15,15;MEINFO=AluSq,248,286,-;NS=1	GT:FT:CGA_IS:CGA_IDC:CGA_IDCL:CGA_IDCR:CGA_RDC:CGA_NBET:CGA_ETS:CGA_KES	.:sns95:76:2:2:0:150:AluSp:0:0.995
NS=1;AN=2;AC=1;CGA_XR=dbsnp.135|rs190723968;AF=0.002;CGA_FI=54991|NM_017891.4|C1orf159|INTRON|UNKNOWN-INC;CGA_RPT=AluSq|Alu|17.4	GT:PS:FT:GQ:HQ:EHQ:CGA_CEHQ:GL:CGA_CEGL:DP:AD:CGA_RDP	1/0:.:PASS:242:242,242:242,242:52,50:-242,0,-242:-52,0,-50:28:13,15:15
…	…	…
NS=1;AN=1;AC=1;CGA_XR=dbsnp.126|rs35082223&dbsnp.134|rs138588344;AF=?&?;CGA_FI=54991|NM_017891.4|C1orf159|INTRON|UNKNOWN-INC;CGA_RPT=AluJb|Alu|20.9	GT:PS:FT:CGA_ALTCALLS:HQ:EHQ:CGA_CEHQ:GL:CGA_CEGL:DP:AD:CGA_RDP	1/.:.:AMBIGUOUS;VQLOW:|-12&AA|-19,.:32,.:64,.:19,.:-32,0,0:-19,0,0:4:2,.:0
…	…	…
END=1051148;NS=1;AN=0	GT:PS	./.:.
…	…	…
NS=1;SVTYPE=BND;MATEID=NA19240-37-240-ASM_4994_R;CGA_BF=0.02	GT:FT:CGA_BNDMPC:CGA_BNDPOS:CGA_BNDDEF:CGA_BNDP	1:SHORT:10:1070131:]1070188]G:PRECISE
NS=1;SVTYPE=BND;MATEID=NA19240-37-240-ASM_4994_L;CGA_BF=0.02	GT:FT:CGA_BNDMPC:CGA_BNDPOS:CGA_BNDDEF:CGA_BNDP	1:SHORT:10:1070188:T[1070131[:PRECISE

Meta-Information Description ASM/vcfBeta-[ASM-ID].vcf.bz2

The following meta-information is included in ***vcfBeta-[ASM-ID].vcf.bz2*** file:

Metadata Tags	Values	Example(s)
##fileformat	Always set to "VCF4.1".	VCFv4.1
##fileDate	Date the file was generated, in YYYYMMDD format.	20120620
##center	Always set to "Complete Genomics".	Complete Genomics
##source	The version of the Complete Genomics software pipeline used to create this VCF file.	CGAPipeline_2.2.0.16
##source_GENOME_REFERENCE	#GENOME_REFERENCE header value from the ***masterVarBeta*** file.	NCBI build 37
##source_MAX_PLOIDY	#MAX_PLOIDY header value from the ***cnvDetailsDiploidBeta*** file.	10
##source_NUMBER_LEVELS	Sample ID:number of levels pair, where "number of levels" is the #NUMBER_LEVELS header value from the sample's ***cnvDetailsNondiploidBeta*** file.	GS00028-DNA_C01:8
##source_NONDIPLOID_WINDOW_WIDTH	#WINDOW_WIDTH header value from the ***cnvDetailsNondiploidBeta*** file(s).	100000
##source_MEAN_GC_CORRECTED_CVG	Sample Id:mean coverage pair, where "mean coverage" is the mean of gcCorrectedCvg values from the sample's ***cnvDetailsDiploidBeta*** file.	GS00028-DNA_C01:45.50
##source_GENE_ANNOTATIONS	#GENE_ANNOTATIONS header value from the ***masterVarBeta*** file.	NCBI build 37.2
##source_DBSNP_BUILD	#DBSNP_BUILD header value from the ***masterVarBeta*** file.	dbSNP build 135
##source_MEI_1000G_ANNOTATIONS	Version of 1000 Genomes MEI call set used to determine whether or not a call corresponds to a known mobile element insertion.	INITIAL-DATA-RELEASE
##source_COSMIC	#COSMIC header value from the ***masterVarBeta*** file.	COSMIC v59
##source_MIRBASE_VERSION	#MIRBASE_VERSION header value from the ***masterVarBeta*** file.	mirBase version 18
##source_PFAM_DATE	#PFAM_DATE header value from the ***masterVarBeta*** file.	April 21, 2011
##source_REPMASK_GENERATED_AT	#REPMASK_GENERATED_AT header value from the ***masterVarBeta*** file.	2011-Feb-15 10:08
##source_SEGDUP_GENERATED_AT	#SEGDUP_GENERATED_AT header value from the ***masterVarBeta*** file.	2010-Dec-01 13:40
##phasing	Always set to "partial".	partial
##reference	The FTP location of the FASTA sequence pointed to by CGA Tools documentation.	ftp://ftp.completegenomics.com/ReferenceFiles/build37.fa.bz2
##contig	Lists the ID, length, assembly, md5, and species of this chromosome.	<ID=1,length=249250621,assembly=B37,md5=1b22b98cdeb4a9304cb5d48026a85128,species="Homo sapiens">

Header Line Description ASM/vcfBeta-[ASM-ID].vcf.bz2

The following description of the mandatory header line columns is from the public VCF specification.

Column Name	Description	Allowed Values
#CHROM	An identifier from the reference genome or an angle-bracketed ID String ("<ID>") pointing to a contig in the assembly file. All entries for a specific CHROM should form a contiguous block within the VCF file. The colon symbol (:) must be absent from all chromosome names to avoid parsing errors when dealing with breakends.<br><br>(Required)	String, no white-space permitted.
POS	The reference position, with the first base having position 1. Positions are sorted numerically, in increasing order, within each reference sequence CHROM. It is permitted to have multiple records with the same POS. Telomeres are indicated by using positions 0 or N+1, where N is the length of the corresponding chromosome or contig.<br><br>(Required)	Integer.
ID	Semicolon-separated list of unique identifiers where available. If this is a dbSNP variant it is encouraged to use the rs number(s). No identifier should be present in more than one data record. If there is no identifier available, then the missing value should be used.	String, no white-space or semi-colons permitted.
REF	Reference base(s). Each base must be one of A,C,G,T,N (case insensitive). Multiple bases are permitted. The value in the POS field refers to the position of the first base in the String. For InDels or larger structural variants, the reference String must include the base before the event (which must be reflected in the POS field).<br><br>(Required)	String.
ALT	Comma separated list of alternate non-reference alleles called on at least one of the samples. Options are base Strings made up of the bases A,C,G,T,N, (case insensitive) or an angle-bracketed ID String ("<ID>") or a breakend replacement string as described in the section on breakends. If there are no alternative alleles, then the missing value should be used.	String; no whitespace, commas, or angle-brackets are permitted in the ID String itself.
QUAL	Phred-scaled quality score for the assertion made in ALT.	
FILTER	PASS if this position has passed all filters, that is, a call is made at this position. Otherwise, if the site has not passed all filters, a semicolon-separated list of codes for filters that fail.<br>For example, "q10;s50" might indicate that at this site the quality is below 10 and the number of samples with data is below 50% of the total number of samples.<br>"0" is reserved and should not be used as a filter String. If filters have not been applied, then this field should be set to the missing value.	String, no white-space or semi-colons permitted.
INFO	Additional information described in the meta-information lines in the file header. INFO fields are encoded as a semicolon-separated series of short keys with optional values in the format:<br><br>`<key>=<data>[,data]`	String, no white-space, semi-colons, or equals-signs permitted.
FORMAT	Data types and order as described in the meta-information lines in the file header.<br>This is followed by one field corresponding to the genome, with the colon-separated data in this field corresponding to the types specified in the format. The first sub-field must always be the genotype (GT) if it is present. There are no required sub-fields.	Colon-separated alphanumeric string.
Genome ASM-ID	Column labeled with the assembly ID of the genome.	

Content Description ASM/vcfBeta-[ASM-ID].vcf.bz2

The content rows in the ***vcfBeta-[ASM-ID].vcf.bz2*** file contain the small variant, CNV, SV, and MEI information. The data is captured in the ALT, INFO, and FORMAT fields as described below.

- Small Variant Data in VCF
- CNVs in VCF
- SVs in VCF
- MEIs in VCF

Small Variant Data in VCF

The small variant — SNP, indels and substitutions — data in the VCF file is derived from the Complete Genomics ***masterVarBeta-[ASM-ID].tsv.bz2*** file. The general approach of performing a conversion from ***masterVarBeta*** file format to VCF is to perform a one-to-one mapping, to the extent possible. Sometimes, however multiple loci from the ***masterVarBeta*** file need to be merged to construct a single VCF locus.

In cases where a one-to-one mapping is not possible, the following conversion rules have been applied:

- Character changes. The ***masterVarBeta*** file uses semicolons to separate multiple records within a column and a colon to separate multiple parts of a record. These characters are used as separators with other annotations in VCF. The following conversions have been applied:

masterVarBeta **Character**	**VCF Character**
Semicolon (;)	Ampersand (&)
Colon (:)	Vertical bar (|)
Period (.)	Omitted

- Long no-called regions. Some very long regions of the genome are no-called, and to fit this information into VCF would normally require outputting very long strings in the <REF> field. As a workaround for such loci, we only output the first base of the locus in the <REF> field , we add the END tag in the <INFO> field, we output <CGA_NOCALL> for the <ALT> column, and the GT sub-field value for each no-called allele is period (.).
- Locus overlap. If a ***masterVarBeta*** locus only partially overlaps the VCF record (only possible for ref-called or no-called loci), the annotations are not transferred to the VCF.
- Period as second value. The per-allele, per-genome annotations of the FORMAT field are always listed with two values. In haploid regions, the second value is always a period (.). This convention is required by *HQ* field, and has been followed for other fields for consistency.
- CNV information omitted. Some of the annotation columns of the ***masterVarBeta*** include CNV information. Because this information is represented by distinct records in the VCF, it is not repeated for each small variant record.

The following content table lists the fields represented in the <ALT>, <INFO>, and <FORMAT> columns of the ***vcfBeta*** files for small variant analysis.

Table 4: Content Description: Small Variant Data in *vcfBeta* Files

Field	Tag	Description / Source	Possible Values / Example
<ALT>	<CGA_NOCALL>	Special <ALT> tag that indicates this record describes a long no-called region of the genome.<br>**Source:** No-called records in ***masterVar***.	<CGA_NOCALL>
<INFO>	NS	Number of samples represented in the VCF file.<br>**Source:** The count of sample columns is used, regardless whether a call is made at this locus.	Integer, always 2 in somatic VCF.<br>**Example:** 2

Field	Tag	Description / Source	Possible Values / Example
<INFO>	AN	Total number of alleles in called genotypes.<br>**Source:** The count of calls (not ".") in the GT tag for this locus, for all samples in the VCF, is used.	Nonnegative integer.<br>**Example:** 4
<INFO>	AC	Allele count in genotypes, for each ALT allele, in the same order as listed.<br>**Source:** For each ALT allele, we count the number of times that ALT allele occurs in the GT tag, for all samples in the VCF.	Comma-separated list of non-negative integers, one for each ALT allele.<br>**Example:** 40,35
<INFO>	AF	Allele frequency value(s) for calls that are corroborated directly by external sources.<br>**Source:** The source is 1000 Genomes Project minor allele frequency information in dbSNP. Multiple entries for the same source mirror the multiple entries for this source appearing in *CGA_XR.*	Ampersand-separated list of `source|<frequency>` values. Precision of `frequency` is three decimal places. Unknown parts of a compound frequency are represented by a question mark (?).<br>**Example:** `dbsnp|0.234&?&dbsnp|0.010`
<INFO>	CGA_XR	External database reference. This is a per ALT-allele annotation, where the rsIDs associated with the variants and the dbSNP version these rsIDs were 'born in'. ALT alleles with no CGA_XR annotations are novel.<br>**Source:** This annotation is taken from the *allele1XRef* and *allele2XRef* columns of the ***masterVarBeta*** file. Colons are replaced with vertical bars, and multiple values are concatenated with ampersands.	Comma-separated list of annotations, one for each ALT allele. For each ALT allele, an ampersand-separated list of external database references is given.<br>**Example:** COSMIC|mut|146371&dbsnp.100|rs2803287,dbsnp.100|rs2803287&dbsnp.111|rs4648738
<INFO>	CGA_FI	Functional impact details.<br>**Source:** This is taken from the *allele1Gene* and *allele2Gene* columns of the ***masterVarBeta*** file. For every annotation, the following columns from the ***gene-[ASM-ID].tsv.bz2*** file are concatenated together using a vertical bar (|) as separator: *geneId*, *mrnaAcc*, *symbol*, *component*, and *impact*. Multiple values for a single ALT allele are concatenated with ampersands.	Comma-separated list of annotations, one for each ALT allele. For each ALT allele, an ampersand-separated list of functional impact annotations is given.<br>**Example:** 728642|NM_024011.2|CDK11A|INTRON|UNKNOWN-INC&728642|NM_033529.2|CDK11A|INTRON|UNKNOWN-INC
<INFO>	CGA_PFAM	PFAM domain that variant overlaps.<br>**Source:** This value is taken from the pfam column of the ***masterVarBeta*** file, replacing colons with vertical bars. Multiple values are concatenated with a comma.	A comma-separated list of PFAM domains is given.<br>**Example:** PFAM|PF00781|DAGK_cat,PFAM|PF01513|NAD_kinase
<INFO>	CGA_MIRB	miRBaseId<br>**Source:** This is taken from the *miRBaseId* column of the ***masterVarBeta*** file, replacing colons with vertical bars. Multiple values are concatenated with a comma.	Comma-separated list of ncRNA annotations for this locus.<br>**Example:** hsa-miR-663b|MIMAT0005867
<INFO>	CGA_SDO	Depth of overlapping segmental duplications.<br>**Source:** The value reported is the maximum of all the *segDupOverlap* values from the ***masterVarBeta*** file for each call contributing to this locus .	Positive integer.<br>**Example:** 9

Field	Tag	Description / Source	Possible Values / Example
<INFO>	CGA_RPT	Overlapping repeatMasker annotations.<br><br>**Source:** This is taken from the *repeatMasker* column of the ***masterVarBeta*** file, replacing colons with vertical bars. Multiple values are concatenated with a comma.	Comma-separated list of repeat masker annotations. Each annotation provides repeat name, repeat family and % divergence from the repeat consensus sequence, separated by a vertical bar (|). Mitochondrial loci are not annotated.<br><br>**Example:** MLT1E1A-int|ERVL-MaLR|38.5
<INFO>	END	End position of the variant described in this record. Used for records that describe very large loci that are no-called. For these loci, the record is formatted as follows: the first base of the locus is provided in REF, the END tag in the INFO column designates the position of the last base of the locus, and the ALT column contains the value "CGA_NOCALL".	Positive integer.<br><br>**Example:** 726099
<FORMAT>	GT	Genotype. Alleles of the genotype are separated by slash if they are unphased. Otherwise, they are separated by a vertical bar, and the PS tag indicates which phase set this locus is a part of.<br><br>**Source:** When calls must be merged to produce the genotype, the alleles of the calls are concatenated. NOTE: If the resulting allele contains "?" or "N", the entire allele is replaced with a period (.) for the GT sub-field.	Slash (/) or vertical bar (|) separated list of allele indexes or period (.).<br><br>**Example:** 0/1
<FORMAT>	PS	Phase set.<br><br>**Source:** For loci whose genotype field is phased (indicated by the vertical bar separator character), this field is filled with the position of the first locus in the phase set. Loci with the same phase set position are phased with each other. Loci that do not have the same phase set position have not been phased with each other, even if the genotype field contains a vertical bar. This field is derived from the *allele1HapLink* and *allele2HapLink* fields of the ***masterVarBeta*** file.	Positive integer, corresponding to chromosome coordinate.<br><br>**Example:** 734042

Field	Tag	Description / Source	Possible Values / Example
<FORMAT>	FT	Sample genotype filters. This is a per-sample sub-field. Note that "FT" is a VCF reserved word.<br><br>**Source:** Derived from the *allele1VarFilter* and *allele2VarFilter* columns of the ***masterVarBeta*** file, if available.	PASS, period (.) or a semicolon-separated list of failed filters. Period appears if none of the calls contributing to this allele have a *varFilter* value. Failed filters include:<br>▪ `VQLOW` — indicates the variant was homozygous with score < 20 or heterogeneous with score < 40. If any call contributing to this allele is marked as `VQLOW`, this field fails the VQLOW filter.<br>▪ `AMBIGUOUS` — for homozygous alleles, indicates there was another non-reference hypothesized sequence that scored within 10 dB of the call; for heterozygous alleles, indicates there was another non-reference hypothesized sequence that scored within 20 dB of the call.<br><br>**Example:** PASS
<FORMAT>	CGA_ALTCALLS	Alternative call sequences and scores; an alternative call is a possible sequence that cannot be confidently rejected in favor of the most likely seqeuence.<br><br>**Source**: *allele1AlternativeCalls* and *allele2AlternativeCalls* columns of the ***masterVarBeta*** file.	An ampersand (&) separated list of `<variant>|<score>` pairs, where `<variant>` is a sequence, and `<score>` is the score of that sequence relative to the score of the called sequence.<br><br>**Example:** if the *CGA_ALTCALLS* value is "`AG|-1&G|-8`", then sequence AG scored 1 dB less than the called sequence and G scored 8 dB less than the called sequence.
<FORMAT>	GL	Genotype likelihoods, as described in the VCF 4.1 specification.<br><br>**Source:** These genotype likelihoods are conservatively inferred from the haplotype quality (HQ) tags. For example, for a heterozygous call of A, C with scores 40, 50, the GL of genotype AA is -50, the GL of AC is 0, and the GL of CC is -40. The GL of TT is also -50, as it can be inferred from the HQ that any genotype containing no C's is at least 50dB worse than the called genotype.	A comma-separated list of integers or period (.), one for each possible genotype.<br><br>**Example:** -100,0,-40
<FORMAT>	CGA_CEHQ	Calibrated haplotype quality based on equal allele fraction assumption.<br><br>**Source:** These qualities are derived by looking up the calibrated score using the varScoreEAF and totalReadCount of this locus in the Complete Genomics calibration. The false positive, undercall, and overcall calibrations are used. For more information, see *Complete Genomics Calibration Methods.*	A comma-separated list of integers or period (.) one for each haplotype.<br><br>**Example:** 40100

Field	Tag	Description / Source	Possible Values / Example
<FORMAT>	CGA_CEGL	Genotype likelihoods, as in the GL tag, except based on equal allele fraction calibrated haplotype quality tags (CEHQ) instead of HQ.<br>**Source:** These genotype likelihoods are conservatively inferred from the calibrated equal allele fraction haplotype quality (CGA_CEHQ) tags. For example, for a heterozygous call of A, C with calibrated scores 40, 50, the CGA_CEGL of genotype AA is -50, the GL of AC is 0, and the GL of CC is -40. The CGA_CEGL of TT is also -50, as it can be inferred from the CGA_CEHQ that any genotype containing no C's is at least 50dB worse than the called genotype.	A comma-separated list of integers or period character, one for each possible genotype.<br>**Example:** -30,0,-40
<FORMAT>	HQ	Haplotype quality.<br>**Source:** Maximum *varScoreVAF*, for all scored calls contributing to the GT. If the GT for a haplotype is period (.), the HQ is also period.	Integer or period (.).<br>**Example:** 150
<FORMAT>	EHQ	Haplotype quality based on Equal Allele Fraction assumption.<br>**Source:** Maximum *varScoreEAF*, for all scored calls contributing to the GT. If the GT for a haplotype is period (.), the EHQ is also period.	Integer or period (.).<br>**Example:** 40
<FORMAT>	GQ	Genotype quality.<br>**Source:** Minimum of the *HQ*, for all alleles of this locus. Period (.) if *HQ* is "." for all alleles of this locus.	Integer or period (.).<br>**Example:** 100
<FORMAT>	DP	Total read depth.<br>**Source:** Maximum of the *totalReadCount* column of the ***masterVarBeta*** file, for all input loci that overlap this VCF locus.	Nonnegative integer.<br>**Example:** 50
<FORMAT>	AD	Allelic depths. Number of reads in each observed allele.<br>**Source:** Maximum of the *allele1ReadCount* and *allele2ReadCount* columns of the ***masterVarBeta*** file that overlap this VCF locus.	Comma-separated list of nonnegative integers.<br>**Example:** 30,20
<FORMAT>	CGA_RDP	Read depth in reference. Number of alleles observed supporting the reference allele.<br>**Source:** Maximum of *referenceAlleleReadCount* column of the ***masterVarBeta*** file for all input loci overlapping this VCF locus.	Nonnegative integer.<br>**Example:** 20
<FORMAT>	SS	Somatic status.<br>Not currently used in ***vcfBeta***.	

CNVs in VCF

The CNV data represented here closely corresponds to the CNV details files: ***cnvDetailsDiploidBeta*** and ***cnvDetailsNondiploidBeta***. Based on this representation, multiple rows will need to be processed to determine CNV boundaries and generate an output analogous to ***cnvSegmentsDiploidBeta*** and ***cnvSegmentsNondiploidBeta*** files.

CNV records are indicated using the special CGA_CNVWIN tag in the <ALT> field. Data are reported for every 2 kb segment of the genome, with some exceptional windows at the very start/end of contigs. This facilitates comparison across multiple samples. Each position therefore does NOT represent the bounds of a CNV. Consequently, there is no genotype value associated with each row. Also as a 2 kb sequence is represented per row, the REF field will include only the first base (for the 2 kb sequence) as denoted by the POS coordinate. Information about samples and their processing for the purposes of CNV calling is recorded in `##source` lines in the header of the ***vcfBeta*** file, specifically the `source_MAX_PLOIDY`, `source_NUMBER_LEVELS`, `source_NONDIPLOID_WINDOW_WIDTH` and `source_MEAN_GC_CORRECTED_CVG` lines described in the "Header Line Description".

The following content table lists the fields represented in the <ALT>, <INFO>, and <FORMAT> fields of the ***vcfBeta*** files for CNV analysis.

Table 5: Content Description of CNV Data in *vcfBeta* Files

Field	Tag	Description / Source	Possible Values / Example
<ALT>	<CGA_CNVWIN>	Special <ALT> tag that indicates this record describes coverage information for a window of the genome.	<CGA_CNVWIN>
<INFO>	NS	Number of samples with data.<br><br>**Source:** Always 2 in somatic VCF.	2<br><br>**Example:** 2
<INFO>	CGA_WINEND	End of coverage window: CNV lines are provided for windows of approximately 2K; this value specifies the ending position for the window described by a specific line.<br><br>**Source:** This is taken from the end field in ***cnvDetailsDiploidBeta-[ASM-ID].tsv*** file.	Positive integer<br><br>**Example:** 102000
<FORMAT>	GT	Genotype.<br><br>**Source:** Always set to "." for CNV-related records.	Period (.).<br><br>**Example:** .
<FORMAT>	CGA_GP	Normalized mean GC corrected coverage for the window described by this record.<br><br>**Source:** This is based on the *gcCorrectedCvg* field from the ***cnvDetailsDiploidBeta-[ASM-ID].tsv*** file. To obtain the value given here, the *gcCorrectedCvg* value is divided by the mean for the genome, which is given in the `##source_MEAN_GC_CORRECTED_CVG` header record. When *gcCorrectedCvg* is 'N' (where estimated coverage is unreliable), CGA_GP is given as a period (.)	Nonnegative floating point value or period (.). The average value is approximately 1.<br><br>**Example:** 0.8
<FORMAT>	GCA_NP	Normalized mean coverage for 2k window: GC-corrected and corrected relative to a multi-sample baseline.<br><br>**Source:** This is taken from the *relativeCvg* field from the ***cnvDetailsDiploidBeta-[ASM-ID].tsv*** File, except for replacing 'N' with period (.).	Nonnegative floating point value or dot (.). The average value is approximately 1.<br><br>**Example:** 1.75

Field	Tag	Description / Source	Possible Values / Example
<FORMAT>	CGA_CP	Diploid-model ploidy call for segment including this interval.<br>**Source:** This is taken from the *calledPloidy* field from the ***cnvDetailsDiploidBeta-[ASM-ID].tsv*** file, except for replacing N with period (.).	Integer between 0 and #source_MAX_PLOIDY, inclusive, or period (.) in no-called regions.<br>**Example:** 3
<FORMAT>	CGA_PS	Diploid-model called ploidy score: Phred-like confidence that the CNV type reported in CGA_CT is correct.<br>**Source:** This is taken from the *ploidyScore* field from the ***cnvDetailsDiploidBeta-[ASM-ID].tsv*** file.	Nonnegative integer.<br>**Example:** 10
<FORMAT>	CGA_CT	Diploid-model CNV type: Classification of called ploidy.<br>**Source:** This is taken from the *calledCNVType* field from the ***cnvDetailsDiploidBeta-[ASM-ID].tsv*** file, except for replacing hypervariable or invariant with period (.), indicating no-called regions.	String:<br>▪ = indicates coverage consistent with reference copy number.<br>▪ + indicates an amplification.<br>▪ - indicates a deletion.<br>▪ . (period) indicates unresolved copy number.<br>**Example:** +
<FORMAT>	CGA_TS	Diploid-model CNV type score.<br>**Source:** This is taken from the *CNVTypeScore* field from the ***cnvDetailsDiploidBeta-[ASM-ID].tsv*** file.	Nonnegative integer.<br>**Example:** 25
<FORMAT>	CGA_CL	Nondiploid-model called level: Called coverage level for segment containing this interval.<br>**Source:** This is taken from the *calledLevel* field from the overlapping 100k window in the ***cnvDetailsNondiploidBeta-[ASM-ID].tsv*** file, except for replacing N with period (.) in regions where relative coverage is highly variable.	Nonnegative floating point value or period (.).<br>**Example:** 1.361
<FORMAT>	CGA_LS	Nondiploid-model called level score: Phred-like confidence that the interval has the called level, as compared to the alternative levels included in the model.<br>**Source:** This is taken from the *levelScore* field from the overlapping 100k window in the ***cnvDetailsNondiploidBeta-[ASM-ID].tsv*** file.	Nonnegative integer.<br>**Example:** 601
<FORMAT>	CGA_LAFS	Maximum likelihood estimate of Lesser Allele Fraction (LAF) for the CNV analysis window.<br>**Source:** This is taken from the *bestLAFsingle* column from the overlapping 100k window in the ***cnvDetailsNondiploidBeta-[ASM-ID].tsv*** file.	Floating point value between 0 and 0.5.<br>**Example**: 0.48
<FORMAT>	CGA_LLAFS	Minimum value of the approximate 99% confidence interval around *CGA_LAFS*.<br>**Source:** This is taken from the *lowLAFsingle* column from the overlapping 100k window in the ***cnvDetailsNondiploidBeta-[ASM-ID].tsv*** file.	Floating point value between 0 and 0.5.<br>**Example**: 0.47
<FORMAT>	CGA_ULAFS	Maximum value of the approximate 99% confidence interval around *CGA_LAFS*.<br>**Source:** This is taken from the *highLAFsingle* column from the overlapping 100k window in the ***cnvDetailsNondiploidBeta-[ASM-ID].tsv*** file.	Floating point value between 0 and 0.5.<br>**Example**: 0.49

SVs in VCF

The "Specifying Complex Rearrangements with Breakends" section of the VCF specification describes the representation of complex structural variations as:

> An arbitrary rearrangement event can be summarized as a set of novel **adjacencies.** Each adjacency ties together 2 **breakends.** The two breakends at either end of a novel adjacency are called **mates.**

The specifications for structural variants in the ***vcfBeta-[ASM-ID].vcf.bz2*** file follows the definitions introduced in the revision of VCF 4.1. The concept of "junctions" in Complete Genomics data is translated to the concept of "adjacency" in the VCF format. Each adjacency ties together 2 breakends. The two breakends at either end of a novel adjacency are called mates. By extension, the left and right sections of a junction are analogous to mates:

- Adjacency: Analogous to Complete Genomics term "junction"
- Breakend: Analogous to Complete Genomics term '"LeftPosition" or "RightPosition"
- Mate: Analogous to Complete Genomics term "LeftSection" or "RightSection"

The following content table lists the tags represented in the <INFO> and <FORMAT> fields of the ***vcfBeta*** files for SV analysis.

Table 6: Content Description: Structural Variation Data in *VcfBeta* Files

Field	Tag	Description / Source	Possible Values / Example
<INFO>	NS	Number of samples. As of now only two samples are supported.<br><br>**Source:** Usually it is one normal sample and one tumor sample.	Integer; always 1 in single-genome VCF.<br><br>**Example:** NS=1
<INFO>	SVTYPE	Type of structural variation.<br><br>**Source:** BND (breakend) will be used to denote each half of a junction, as suggested in the VCF 4.1 specification.	Always BND.<br><br>**Example:** SVTYPE=BND
<INFO>	CGA_BF	Frequency at which breakend is detected in set of baseline genomes publicly released by Complete Genomics.<br><br>**Source:** Corresponds to the value from *FrequencyInBaselineGenomeSet* column in the respective junctions file.	Floating point value between 0 and 1.<br><br>**Example:** 0.24
<INFO>	CGA_MEDEL	Mobile element deletion: If the detected structural variant is consistent with deletion of a mobile element, the specific class (e.g., AluYa5) and the boundaries of the mobile element (chromosome, start and end) will be provided.<br><br>**Source:** Based on the value from *DeletedTransposableElement* column in the respective junctions file.	Comma separated list of four elements: Mobile element type, chromosome, start, end.<br><br>**Example:** AluYb8,1,43008205,43008523
<INFO>	CGA_XR	If a structural variation is consistent with a deletion in dbSNP, the corresponding rsID will be provided.<br><br>**Source:** Based on the value from *XRef* column in the respective junctions file.	rsID.<br><br>**Example:** rs70949527

Field	Tag	Description / Source	Possible Values / Example
<INFO>	MATEID	ID of mate breakend. For a left mate, as designated by *_L in ID, the MATEID will be a pointer to a mate whose position is denoted by *RightChr*, *RightPosition*, *RightStrand*, and *RightLength*. For a right mate, as designated by *_R the converse is true.<br><br>**Source:** Based on the values from *LeftChr*, *LeftPosition*, *LeftStrand*, *LeftLength* columns (for a left mate), or *RightChr*, *RightPosition*, *RightStrand*, *RightLength* (for the right mate), in the respective junctions file.	GSXXXXX-<junction ID>-Y where X's are digits and Y is either 'L' or 'R'.<br><br>**Example:** GS00059_4567_L
<INFO>	CGA_BNDG	Transcript name and strand of genes containing breakend<br><br>**Source:** Based on the value from *RightGenes* column (for a right mate), or *LeftGenes* column (for a left mate), in the respective junctions file.	Ampersand-delimited list of one or more values of the following format: <mRNA>|<strand> where <mRNA> is the mRNA accession number and <strand> is the strand.<br><br>**Example:** NM_024011|-
<INFO>	CGA_BNDGO	Transcript name and strand of genes containing mate breakend<br><br>**Source:** Based on the value from *RightGenes* column (for a left mate), or *LeftGenes* column (for a right mate), in the respective junctions file.	Ampersand-delimited list of one or more values of the following format: <mRNA>|<strand> where <mRNA> is the mRNA accession number and <strand> is the strand.<br><br>**Example:** NM_024011|-
<FORMAT>	CGA_BNDP	Precision of breakend: If precise breakpoint of the structural variation junction ("breakend" in VCF notation) is not known, the BNDP field within FORMAT is set to IMPRECISE.<br><br>**Source:** Based on the value from *JunctionSequenceResolved* column in the respective junctions file: PRECISE for Y, IMRPECISE for N.	PRECISE or IMPRECISE.<br><br>**Example:** PRECISE
<FORMAT>	CGA_BNDMPC	Mate pair count supporting a breakend.<br><br>**Source:** Based on the value from *DiscordantMatePairAlignments* column in the respective junctions file.	Positive integer.<br><br>**Example:** 10
<FORMAT>	CGA_BNDPOS	Position of breakend as detected in individual genome: When breakends are detected in multiple genomes at proximate locations (within 200 bases of POS), the precise position detected within each genome is indicated.<br><br>**Source:** Based on the value from *RightPosition* column (for a right mate), or *LeftPosition* column (for a left mate), in the respective junctions file.	Positive integer.<br><br>**Example:** 815189

Field	Tag	Description / Source	Possible Values / Example
<FORMAT>	CGA_BNDDEF	Breakend definition in individual genome: When breakends are detected in multiple genomes at proximal locations (within 200 bases of POS), the precise definition in VCF 4.1 syntax for the breakend detected in an individual genome.<br><br>Source: Based on the value from *RightPosition* column (for a right mate), or *LeftPosition* column (for a left mate), as well as *LeftStrand*, *RightStrand*, and *TransitionSequence* column in the respective junctions file.	<Base><TS>]<POS>]<br>or<br>[<POS>[<TS><BASE><br>where <Base> is the base at the breakend position, <TS> is the transition sequence and <POS> is the mate breakend position. The orientation of the square brackets indicates the direction to the mate gap.<br><br>**Example:** CG]227760836]
<FORMAT>	FT	Filter tags, one for each of the filters that the junction did't pass, or PASS if all filters passed:<br>▪ URR: Proximity to a known underrepresented repeat in the human genome, indicating that an apparent translocation is likely to be spurious.<br>▪ MPCBT: Mate pair count filter. Fewer than 10 mate-paired reads support a breakend.<br>▪ SHORT: Junction side length flag – shorter than 70 bases. The sequence span over which mate pair support exists for the breakend.<br>▪ TSNR: Transition sequence resolution filter. Transition sequence is not resolved: as indicated by N in the transition sequence.<br>▪ INTERBL: Interchromosomal junction with non-zero baseline frequency.<br>▪ PASS: all filters passed. None of the above conditions are true. Indicates that this junction is a high confidence one.<br><br>Source:<br>▪ URR: based on the value from *KnownUnderrepresentedRepeat* column in the junctions file:<br>▪ MPCBT: based on the value from *DiscordantMatePairAlignments* column in the junctions file<br>▪ SHORT: based on the value from *LeftLength* and *RightLengthcolumns* in the junctions file<br>▪ TSNR: based on the value from *JunctionSequenceResolved* column in the junctions file<br>▪ INTERBL: based on the value from *Interchromosomal* column in the junctions file and on the Complete Genomics' SV baseline.	Semicolon-delimited list including one or more of the following values: URR, MPCBT, SHORT, TSNR, INTERBL, or PASS.<br><br>**Example:** MPCBT;INTERBL

MEIs in VCF

The mobile element insertion (MEI) data represented here closely corresponds to the file ***mobileElementInsertionsBeta***, with a 1-to-1 relationship between data rows.

The following content table lists the tags represented in <ALT>, <INFO>, and <FORMAT> fields of the ***vcfBeta*** files for MEI analysis.

Table 7: Content Description: MEI Data in *vcfBeta* Files

Field	Tag	Description / Source	Possible Values / Example
<ALT>	INS:ME:ALU	Indication that the current row describes insertion of an ALU element.<br><br>**Source:** Derived from *ElementType* column of ***mobileElementInsertionsBeta-[ASM-ID].tsv*** file.	**Example:** INS:ME:ALU
<ALT>	INS:ME:L1	Indication that the current row describes insertion of an L1 element,<br><br>**Source:** Derived from *ElementType* column of ***mobileElementInsertionsBeta-[ASM-ID].tsv*** file.	**Example:** INS:ME:L1
<ALT>	INS:ME:LTR	Indication that the current row describes insertion of an LTR element.<br><br>**Source:** Derived from *ElementType* column of ***mobileElementInsertionsBeta-[ASM-ID].tsv*** file.	**Example:** INS:ME:LTR
<ALT>	INS:ME:SVA	Indication that the current row describes insertion of an SVA element.<br><br>**Source:** Derived from *ElementType* column of ***mobileElementInsertionsBeta-[ASM-ID].tsv*** file.	**Example:** INS:ME:SVA
<ALT>	INS:ME:MER	Indication that the current row describes insertion of a MER element.<br><br>**Source:** Derived from *ElementType* column of ***mobileElementInsertionsBeta-[ASM-ID].tsv*** file.	**Example:** INS:ME:MER
<ALT>	INS:ME:HERV	Indication that the current row describes insertion of an HERV element.<br><br>**Source:** Derived from *ElementType* column of ***mobileElementInsertionsBeta-[ASM-ID].tsv*** file.	**Example:** INS:ME:HERV
<ALT>	INS:ME:POLYA	Indication that the current row describes insertion of a poly-A sequence.<br><br>**Source:** Derived from *ElementType* column of ***mobileElementInsertionsBeta-[ASM-ID].tsv*** file.	**Example:** INS:ME:POLYA
<INFO>	NS	Number of samples with data<br><br>**Source:** Always 1	1<br><br>**Example:** 1
<INFO>	IMPRECISE	Imprecise structural variation<br><br>**Source:** Always imprecise	**Example:** IMPRECISE
<INFO>	SVTYPE	Type of structural variant. Always INS.<br><br>**Source:** Always INS	INS<br><br>**Example:** INS
<INFO>	END	End position of the variant described in this record.<br><br>**Source:** Value is midpoint of *InsertRangeBegin* and *InsertRangeEnd* columns from ***mobileElementInsertionsBeta-[ASM-ID].tsv*** file.	Positive integer.<br><br>**Example:** 724293

Field	Tag	Description / Source	Possible Values / Example
<INFO>	SVLEN	Difference in length between REF and ALT alleles.<br><br>**Source:** Value is computed as difference between *ElementSequenceBegin* and *ElementSequenceEnd* columns in ***mobileElementInsertionsBeta-[ASM-ID].tsv*** file.	Integer.<br><br>**Example:** 39
<INFO>	CIPOS	Confidence interval around POS for imprecise variants.<br><br>**Source:** Value is computed as change in coordinates from POS to *InsertRangeBegin* and *InsertRangeEnd* column in ***mobileElementInsertionsBeta-[ASM-ID].tsv*** file.	Two comma-separated integers, the first nonpositive and the second nonnegative.<br><br>**Example:** -202202
<INFO>	MEINFO	Mobile element info of the form NAME,START,END,POLARITY.<br><br>**Source:** Values correspond to *ElementType*, *ElementSequenceBegin*, *ElementSequenceEnd*, and *Strand* columns in ***mobileElementInsertionsBeta-[ASM-ID].tsv*** file.	Four comma-separated values as follows: String identifying mobile element type, e.g. AluYb8, L1HS, etc; nonnegative integer; nonnegative integer; + or -.<br><br>**Example:** L1HS,3173,3211,+
<FORMAT>	GT	Genotype; always missing.<br><br>**Source:** Always period (.)	Period (.).<br><br>**Example:** .
<FORMAT>	FT	Filter status:<br><br>**Source:** PASS if CGA_KES ≤0.75<br>sns75 if 0.75 < CGA_KES ≤ 0.95<br>sns95 if CGA_KES > 0.95	PASS, sns95, or sns75.<br><br>**Example:** sns95
<FORMAT>	CGA_IS	Measure of confidence that there is a mobile element insertion (higher == more confidence).<br><br>**Source:** Value is taken from *InsertionScore* column in ***mobileElementInsertionsBeta-[ASM-ID].tsv*** file.	Positive floating point value.<br><br>**Example:** 18
<FORMAT>	CGA_IDC	Count of paired ends consistently indicating a mobile element insertion.<br><br>**Source:** Value is taken from *InsertionDnbCount* column in ***mobileElementInsertionsBeta-[ASM-ID].tsv*** file.	Positive integer.<br><br>**Example:** 1
<FORMAT>	CGA_IDCL	Count of paired ends consistently indicating a mobile element insertion and anchored 5' of the insertion point on the reference.<br><br>**Source:** Value is taken from *InsertionLeftDnbCount* column in ***mobileElementInsertionsBeta-[ASM-ID].tsv*** file.	Nonnegative integer.<br><br>**Example:** 0
<FORMAT>	CGA_IDCR	Count of paired ends consistently indicating a mobile element insertion and anchored 3' of the insertion point on the reference.<br><br>**Source:** Value is taken from *InsertionRightDnbCount* column in ***mobileElementInsertionsBeta-[ASM-ID].tsv*** file.	Nonnegative integer.<br><br>**Example:** 1
<FORMAT>	CGA_RDC	Count of paired ends supporting the presence of a reference allele.<br><br>**Source:** Value is taken from *ReferenceDnbCount* column in ***mobileElementInsertionsBeta-[ASM-ID].tsv*** file.	Nonnegative integer, or '.' when not readily ascertained.<br><br>**Example:** .

Field	Tag	Description / Source	Possible Values / Example
<FORMAT>	CGA_NBET	Next (i.e. second)-best estimate of the (sub)type of inserted mobile element.<br>**Source:** Value is taken from *NextBestElementType* column in ***mobileElementInsertionsBeta-[ASM-ID].tsv*** file.	String identifying mobile element type, e.g. AluYb8, L1HS, etc.<br>**Example:** L1PREC2
<FORMAT>	CGA_ETS	Measure of confidence that the ElementType (MEINFO:NAME) is correct.<br>**Source:** Value is taken from *ElementTypeScore* column in ***mobileElementInsertionsBeta-[ASM-ID].tsv*** file.	Nonnegative floating point value.<br>**Example:** 16
<FORMAT>	CGA_KES	Fraction of known MEI insertion polymorphisms called for this sample that receive at least as good an InsertionScore (CGA_IS) as the current call.<br>**Source:** Value is taken from *KnownEventSensitivityForInsertionScore* column in ***mobileElementInsertionsBeta-[ASM-ID].tsv*** file.	Nonnegative floating point value.<br>**Example:** 0.999

Annotated Variants within Genes

ASM/gene-[ASM-ID].tsv.bz2

The tab-separated text file ***gene-[ASM-ID].tsv.bz2*** contains annotations of variations that fall within RefSeq mRNAs. Each variation is annotated with its effect on the transcript, such as frameshift, silent, or nonsense mutations. The collection of RefSeq transcripts used for annotation is taken from a specific NCBI genome annotation build, the identity of which is in the #GENE_ANNOTATIONS field of the header of this file. Alignment data for the transcripts can be downloaded from NCBI as described in "References."

Example — *ASM/gene-[ASM-ID].tsv.bz2*

The first section shows the first 12 columns; the remaining 13 columns appear in the lower section. The second section of data repeats the *index* column at the left edge to more easily match the data with the previous section of data; the *index* column is not repeated in the actual data.

>index	locus	allele	chromosome	begin	end	varType	reference	call	xRef	geneId	mrnaAcc
97	1268	2	chr1	58608	58615	no-call		?		79501	NM_001005484.1
98	1270	1	chr1	58758	58759	snp	G	A	dbsnp.100:rs2854683	79501	NM_001005484.1
98	1270	2	chr1	58758	58759	snp	G	A	dbsnp.100:rs2854683	79501	NM_001005484.1
99	1272	1	chr1	58804	58811	no-call		?		79501	NM_001005484.1
99	1272	2	chr1	58804	58811	no-call		?		79501	NM_001005484.1
100	1274	2	chr1	58996	58997	no-call-rc	A	N		79501	NM_001005484.1
100	1274	1	chr1	58996	58997	ref	A	A		79501	NM_001005484.1
101	1276	1	chr1	59143	59150	no-call		?		79501	NM_001005484.1
101	1276	2	chr1	59143	59150	no-call		?		79501	NM_001005484.1
102	1278	1	chr1	59315	59316	snp	G	A	dbsnp.100:rs2854682	79501	NM_001005484.1
102	1278	2	chr1	59315	59316	ref	G	G		79501	NM_001005484.1
103	1280	1	chr1	59373	59374	snp	A	G	dbsnp.100:rs2691305	79501	NM_001005484.1
103	1280	2	chr1	59373	59374	snp	A	G	dbsnp.100:rs2691305	79501	NM_001005484.1
104	1282	1	chr1	59414	59415	snp	G	C	dbsnp.100:rs2531266; dbsnp.129:rs55874132	79501	NM_001005484.1
104	1282	2	chr1	59414	59415	ref	G	G		79501	NM_001005484.1
105	1284	1	chr1	59431	59432	snp	T	C	dbsnp.100:rs2531267	79501	NM_001005484.1

>index	proteinAcc	symbol	orientation	component	componentIndex	hasCodingRegion	impact	nucleotidePos	proteinPos	annotationRefSequence	sampleSequence	genomeRefSequence	pfam
97	NP_001005484.1	OR4F5	+	TSS-UPSTREAM		Y	UNKNOWN-VNC						
98	NP_001005484.1	OR4F5	+	TSS-UPSTREAM		Y	UNKNOWN-INC						
98	NP_001005484.1	OR4F5	+	TSS-UPSTREAM		Y	UNKNOWN-INC						
99	NP_001005484.1	OR4F5	+	TSS-UPSTREAM		Y	UNKNOWN-VNC						
99	NP_001005484.1	OR4F5	+	TSS-UPSTREAM		Y	UNKNOWN-VNC						
100	NP_001005484.1	OR4F5	+	CDS	0	Y	UNKNOWN-VNC	43	14	E	?	E	
100	NP_001005484.1	OR4F5	+	CDS	0	Y	NO-CHANGE	43	14	E	E	E	
101	NP_001005484.1	OR4F5	+	CDS	0	Y	UNKNOWN-VNC	190	63	LSL	?	RLQ	PFAM:PF00001:7tm_1
101	NP_001005484.1	OR4F5	+	CDS	0	Y	UNKNOWN-VNC	190	63	LSL	?	RLQ	PFAM:PF00001:7tm_1
102	NP_001005484.1	OR4F5	+	CDS	0	Y	SYNONYMOUS	362	120	K	K	K	PFAM:PF00001:7tm_1
102	NP_001005484.1	OR4F5	+	CDS	0	Y	NO-CHANGE	362	120	K	K	K	PFAM:PF00001:7tm_1
103	NP_001005484.1	OR4F5	+	CDS	0	Y	MISSENSE	420	140	T	A	T	PFAM:PF00001:7tm_1
103	NP_001005484.1	OR4F5	+	CDS	0	Y	MISSENSE	420	140	T	A	T	PFAM:PF00001:7tm_1
104	NP_001005484.1	OR4F5	+	CDS	0	Y	SYNONYMOUS	461	153	A	A	A	PFAM:PF00001:7tm_1
104	NP_001005484.1	OR4F5	+	CDS	0	Y	NO-CHANGE	461	153	A	A	A	PFAM:PF00001:7tm_1
105	NP_001005484.1	OR4F5	+	CDS	0	Y	MISSENSE	478	159	L	P	L	PFAM:PF00001:7tm_1

File-Specific Header Description *ASM/gene-[ASM-ID].tsv.bz2*

Key	Description	Allowed Values
#ASSEMBLY_ID	Name of the assembly	"<assembly-name>-ASM". For example, "GS000000474-ASM".
#COSMIC	COSMIC version used for annotation	"COSMIC vXX", where X's are digits. For example "COSMIC v48".
#DBSNP_BUILD	dbSNP version used for annotation	"dbSNP build XXX" where X's are digits. For example, "dbSNP build 130".
#FORMAT_VERSION	Version number of the file format, for example, "0.6"	Two or more digits separated by periods.
#GENERATED_AT	Date and time of the assembly	Year-Month-Day Time. For example "2010-Sep-08 20:27:52.457773".
#GENERATED_BY	Assembly pipeline component that generated the output.	Alpha-numeric string.
#GENE_ANNOTATIONS	NCBI annotation build	"NCBI build XX.X" where X's are digits.
#GENOME_REFERENCE	Human genome build used for assembly	"NCBI build XX" where X's are digits.
#SAMPLE	Complete Genomics identifier of the sample from which the library was created	"GSXXXXX-DNA_YZZ" where<br>▪ X's are digits<br>▪ -DNA_ is literal<br>▪ YZZ is the location of the sample in a 96-well plate with Y as one of "A" through "H" and ZZ is one of "01" through "12"<br>For example "GS12345-DNA_A01"

Key	Description	Allowed Values
#SOFTWARE_VERSION	Assembly pipeline build number	Two or more digits separated by periods.
#TYPE	Indicates the type of data contained in the file.	`GENE-ANNOTATION`: variations annotated with impact on RefSeq genes.
#PFAM_DATE	Date on which Pfam information was downloaded from NCBI Conserved Domain Database	Day-Month-Year. For example "13-Aug-10".

Content Description ASM/gene-[ASM-ID].tsv.bz2

	Column Name	Description
1	index	Identifier for this annotation.
2	locus	Identifier for the locus. Identifier is the identifier from the ***var-[ASM-ID].tsv*** file.
3	allele	Identifier for each allele at the variation locus. For diploid chromosomes, 1 or 2.
4	chromosome	Chromosome name in text: `chr1`, `chr2`, ..., `chr22`, `chrX`, `chrY`. The mitochondrion is represented as `chrM`. The pseudo-autosomal regions within the sex chromosomes X and Y are reported at their coordinates on chromosome X.
5	begin	Reference coordinates specifying the start of the variation (not the locus). Uses the half-open zero-based coordinate system. See "Sequence Coordinate System" for more information.
6	end	Reference coordinates specifying the end of the variation (not the locus). Uses the half-open zero-based coordinate system. See "Sequence Coordinate System" for more information.
7	varType	Type of variation, as reported in the ***var-[ASM-ID].tsv*** file. See "Variations Type Description."
8	reference	The reference sequence at the locus of the variation. Empty when *varType* is `ins`.
9	call	The observed sequence at the locus of the variation. Empty when *varType* is `del`. Question mark (`?`) indicates 0 or more unknown bases within the sequence; `N` indicates exactly one unknown base within the sequence.
10	xRef	Cross-reference to external identifier for variation. Currently populated for variations reported in dbSNP and COSMIC.<br>Format for dbSNP: `dbsnp.<build>:<rsID>`, with multiple entries separated by the semicolon (;). `build` indicates in which build of dbSNP this entry first appeared. For example, `dbsnp.129:rs12345`.<br>Format for COSMIC: `COSMIC.<type>:identifier`, with multiple entries separated by the semicolon (;). `<type>` indicates COSMIC classification of somatic variants. For example for a non-coding variant, *xRef* would contain "COSMIC:ncv_id:139111", where type indicates non-coding variant.
11	geneId	Entrez Gene identifier of the locus in which this variation falls.
12	mrnaAcc	RefSeq mRNA accession number (versioned), for example "NM_152486.2".
13	proteinAcc	RefSeq protein accession number (versioned), for example "NP_689699.2".
14	symbol	NCBI Gene Symbol. For example, "GAPDH".
15	orientation	Orientation of the transcript with respect to the reference genome, "+" for positive strand, "-" for negative strand.

	Column Name	Description
16	component	Category of the region of the gene where this variation is located. Indicates the area of the locus this variation falls in. Can be one of the following:<br>▪ CDS: Region of nucleotides that encodes the sequence of amino acids in the translated protein.<br>▪ INTRON: Region of nucleotides within a gene that is removed before translation of mRNA.<br>▪ DONOR or ACCEPTOR: Indicates that the variation falls inside the 6 bases of the splice donor site or the 15 bases of the splice acceptor site.<br>▪ TSS-UPSTREAM: Indicates that the variation falls within the 7.5 kb region upstream of 5' transcription start site of a gene.<br>▪ SPAN5, SPAN3, or SPAN: SPAN5 and SPAN3 indicate that the variation overlaps an exon and another component, such as, ACCEPTOR and CDS, or TSS-UPSTREAM and UTR5. SPAN5 indicates that the 5' end of the exon is one of the components. SPAN3 indicates that the 3' end of the exon is one of the components. SPAN indicates that the variation overlaps an entire exon.<br>▪ UTR5, UTR3, or UTR: Indicates that the variation falls inside the 5' untranslated region (UTR5) or 3' untranslated region (UTR3) of protein coding genes, or genes with no known coding region (UTR)
17	componentIndex	Number indicating which exon or intron is affected by this variation (0-based, from 5' to 3' on the annotation mRNA).
18	hasCodingRegion	Indicates if transcript has coding region. Can be Y or N.
19	impact	Indicates the type of effect this variation has on the protein sequence. Currently empty or one of:<br>▪ NO-CHANGE: The sequence of this allele is identical to the canonical transcript sequence (which may or may not be identical to the reference sequence used in the assembly). Also, non-GT/AG conserved splice site sequences or AT/AC rare splice site sequences become canonical sequences.<br>▪ SYNONYMOUS: The DNA sequence for this transcript has changed, but there is no change in the protein sequence: the altered codon codes for the same amino acid<br>▪ MISSENSE: The DNA sequence for this transcript has changed and there is a change in the protein sequence as well, since the codon codes for a different amino acid. There is no change in size of the protein<br>▪ NONSENSE: The DNA sequence for this transcript has changed and has resulted in a STOP codon (TGA, TAG or TAA), resulting in an early termination of the protein translation.<br>▪ NONSTOP: The DNA sequence for this transcript has changed and has resulted in the change of a STOP codon (TGA, TAG or TAA) into a codon that codes for an amino acid, likely resulting in the continuation of the translation for this protein.<br>▪ DELETE: The DNA sequence for this transcript has changed and the length of the deletion is a multiple of 3, resulting in deletion of amino acids in the sequence in-frame, with no neighboring amino acids modified<br>▪ INSERT: The DNA sequence for this transcript has changed and the length of the insertion is a multiple of 3, resulting in the insertion of amino acids in the sequence in-frame, with no neighboring amino acids modified.<br>▪ DELETE+: The DNA sequence for this transcript has changed and the length of the deletion is a multiple of 3, occurs out of frame, and results in the deletion of amino acid(s) with possible modification of one or both of the neighboring codons.<br>▪ INSERT+: The DNA sequence for this transcript has changed and the length of the insertion is a multiple of 3, occurs out of frame, and results in the insertion of amino acid(s) with possible modification of one or both of the neighboring codons.<br>▪ FRAMESHIFT: The DNA sequence for this transcript has changed and has resulted in a frameshift for this protein.<br>▪ MISSTART: The DNA sequence for this transcript has changed and resulted in the change of a START codon into a codon that codes for something other than a start codon, likely resulting in a non-functional gene.<br>▪ DISRUPT: GT or AG conserved donor and acceptor splice site sequence has changed to something that is incompatible. Also used if rare AT/AC sequence has

	Column Name	Description
		changed to something that is incompatible.<br>▪ UNKNOWN-VNC: Impact unknown due to the fact that one or both alleles have no-calls (N or ?).<br>▪ UNKNOWN-INC: Impact unknown due to lack of biological information. For example, impact of variation in introns (possible enhancer location) or events spanning splice and coding sequence (is splicing broken and the exon not included?)<br>▪ UNKNOWN-TR: Impact unknown due to the transcript being rejected by annotation pipeline. Conditions for transcript rejection include: 1) transcript contains unknown ("X") amino acid, 2) transcript start and/or stop coding positions are unknown, 3) transcript contains unspecified nucleotides, and 4) transcript maps to unknown location/chromosome.
20	nucleotidePos	Start position of the variation in the mRNA. Counted from the start of the mRNA sequence (0 based). If *component* = DONOR or ACCEPTOR, *nucleotidePos* represents the boundary between exons where the splice site is mapped to nucleotide sequence.
21	proteinPos	Start position of the variation in the protein sequence. (0 based). If *component* = DONOR or ACCEPTOR, *proteinPos* represents the boundary between exons where the splice site is mapped to protein sequence.
22	annotationRefSequence	This value represents the amino acid sequence for this allele before modification. Stop codons are represented using character '*' and unknown codons are represented using '?' character Amino acid sequence is derived directly from the transcript sequence. It is NOT derived from the reference genome sequence used in the assembly since that may be different. If *component* = DONOR or ACCEPTOR, then this field is empty.
23	sampleSequence	For variants within coding region, this value represents the amino acid sequence for this allele after modification. Stop codons are represented using character '*' and no-called amino acids are represented using the '?' character. This amino acid sequence is derived directly from the transcript sequence and modified. It is NOT derived from the reference genome sequence used in the assembly. For variants within splice site donor or acceptor regions, this value represents the nucleotide sequence of splice site donor or splice site acceptor region for this allele after modification and may contain N and ? characters that represent one-base no-calls and unknown length no-calls, respectively.
24	genomeRefSequence	This amino acid sequence IS derived from the reference genome sequence used in the assembly and may be different than *annotationRefSequence*. Stop codons are represented using character '*' and unknown codons are represented using '?' character. For variants within splice site donor or acceptor regions, this value represents the sequence of splice site donor or splice site acceptor region for this allele before modification.
25	pfam	Pfam identifier and domain name of the locus in which this variation falls. Format:<br>PFAM:<identifier>:<domain name><br>For example, "PF00069:Pkinase".

Annotated Variants within Non-coding RNAs

ASM/ncRNA-[ASM-ID].tsv.bz2

The tab-separated text file ***ncRNA-[ASM-ID].tsv.bz2*** contains annotations of variations that fall within a non-coding RNAs. This file contains variants found in known microRNA in miRBase. Each variation is annotated with a miRBase identifier and accession of the mature or pre-miRNA that it falls within. The version of miRBase used for annotation is indicated in #MIRBASE_VERSION field of the header of this file.

Example *ASM/ncRNA-[ASM-ID].tsv.bz2*

The first section shows the first 8 columns; the remaining 3 columns appear in the lower section. The second section of data repeats the *index* column at the left edge to more easily match the data with the previous section of data; the *index* column is not repeated in the actual data.

>index	locus	allele	chromosome	begin	end	varType	reference	call
262	15899359	1	chr12	61283749	61283761	ref	GCAATTTTCTAA	GCAATTTTCTAA
263	15899359	2	chr12	61283749	61283761	no-call	GCAATTTTCTAA	?
264	15899361	1	chr12	61283773	61283773	ins		A
265	15899361	2	chr12	61283773	61283773	no-call		?
266	15899363	1	chr12	61283776	61283777	ref	T	T
266	15899363	2	chr12	61283776	61283777	no-call-rc	T	N

>index	xRef	miRBaseId
262		hsa-let-7i:MI0000434;hsa-let-7i:MIMAT0000415
263		hsa-let-7i:MI0000434;hsa-let-7i:MIMAT0000415
264	dbsnp.120:rs11400719	hsa-let-7i:MI0000434
265		hsa-let-7i:MI0000434
266		hsa-let-7i:MI0000434
266		hsa-let-7i:MI0000434

File-Specific Header Description *ASM/ncRNA-[ASM-ID].tsv.bz2*

Key	Description	Allowed Values
#ASSEMBLY_ID	Name of the assembly	"<assembly-name>-ASM". For example, "GS000000474-ASM".
#COSMIC	COSMIC version used for annotation	"COSMIC vXX", where X's are digits. For example, "COSMIC v48".
#DBSNP_BUILD	dbSNP version used for annotation	"dbSNP build XXX" where X's are digits. For example, "dbSNP build 130".
#FORMAT_VERSION	Version number of the file format	Two or more digits separated by periods. For example, "0.6".
#GENERATED_AT	Date and time of the assembly	Year-Month-Day Time. For example "2010-Sep-08 20:27:52.457773".

Key	Description	Allowed Values
#GENERATED_BY	Assembly pipeline component that generated the output.	Alpha-numeric string.
#GENOME_REFERENCE	Human genome build used for assembly	"NCBI build XX" where X's are digits.
#SAMPLE	Complete Genomics identifier of the sample from which the library was created	"GSXXXXX-DNA_YZZ" where<br>▪ X's are digits<br>▪ -DNA_ is literal<br>▪ YZZ is the location of the sample in a 96-well plate with Y as one of "A" through "H" and ZZ is one of "01" through "12"<br>For example "GS12345-DNA_A01"
#SOFTWARE_VERSION	Assembly pipeline build number	Two or more digits separated by periods.
#TYPE	Indicates the type of data contained in the file.	GENE-ANNOTATION: variations annotated with impact on non-coding RNAs.
#MIRBASE_VERSION	miRBase version used for annotation	"miRBase build XX" where X's are digits.

Content Description ASM/ncRNA-[ASM-ID].tsv.bz2

	Column Name	Description
1	index	Identifier for this annotation.
2	locus	Identifier for the locus. This identifier is the identifier from the ***var-[ASM-ID].tsv*** file. See locus in the content description for "Variations."
3	allele	Identifier for each allele at the variation locus. For diploid chromosomes, 1 or 2.
4	chr	Chromosome name in text: chr1, chr2, ..., chr22, chrX, chrY. The mitochondrion is represented as chrM. The pseudo-autosomal regions within the sex chromosomes X and Y are reported at their coordinates on chromosome X.
5	begin	Reference coordinates specifying the start of the variation (not the locus). Uses the half-open zero-based coordinate system. See "Sequence Coordinate System" for more information.
6	end	Reference coordinates specifying the end of the variation (not the locus). Uses the half-open zero-based coordinate system. See "Sequence Coordinate System" for more information.
7	varType	Type of variation, as reported in the ***var-[ASM-ID].tsv*** file. See "Variations Type Description."
8	reference	The reference sequence at the locus of the variation. Empty when *varType* is ins.
9	call	The observed sequence at the locus of the variation. Empty when *varType* is del. Question mark (?) indicates 0 or more unknown bases within the sequence; N indicates exactly one unknown base within the sequence.
10	xRef	Cross-reference to external identifier for variation. Currently populated for variations reported in dbSNP and COSMIC.<br>Format for dbSNP: dbsnp.<build>:<rsID>, with multiple entries separated by the semicolon (;). build indicates in which build of dbSNP this entry first appeared. For example, dbsnp.129:rs12345.<br>Format for COSMIC: COSMIC.<type>:identifier, with multiple entries separated by the semicolon (;). <type> indicates COSMIC classification of somatic variants. For example for a non-coding variant, *xRef* would contain "COSMIC:ncv_id:139111".
11	miRBaseId	miRBase Identifier and corresponding unique miRBase accession number for mature and pre-miRNA in which the variant was found. If the variant is found in mature miRNA, identifiers and accessions for both mature and pre-miRNA are listed. If the variant is found in a pre-miRNA location that does not include a mature miRNA sequence, only the pre-miRNA identifier and accession are listed.

Count of Variations by Gene

ASM/geneVarSummary-[ASM-ID].tsv

The gene variation summary file ***geneVarSummary-[ASM-ID].tsv*** is a tab-separated text file that contains counts of variations that fall within a RefSeq transcript and information regarding copy number in the transcript and coverage in the transcript, relative to the genome average coverage. For genes with multiple isoforms the variations are counted for each isoform. Note that variations are categorized according to their presence or absence in dbSNP. Novel variants are those not in dbSNP. The version of dbSNP used for annotation can be found in the header of the file on the line which begins with `#DBSNP_BUILD`. The version of RefSeq used can be found in the `#GENE_ANNOTATIONS` field of the header of this file. For more information on the annotation of a given reference genome build, refer to the Release Notes for the Reference Sequence build. Functional impact of variants in the coding regions of genes is determined using RefSeq alignment data, which can be downloaded from NCBI as described in "References."

Example — *ASM/geneVarSummary-[ASM-ID].tsv*

>geneId	mrnaAcc	symbol	chromosome	begin	end	missense	nonsense	nonStop	misStart	frameshift	inframe	disrupt	total	missenseNovel	nonsenseNovel	nonStopNovel	misStartNovel	frameshiftNovel	inframeNovel	disruptNovel	totalNovel	relativeCvg	calledPloidy
6891	NM_018833.2	TAP2	chr6	32789609	32806547	2	0	0	0	0	0	0	2	0	0	0	0	0	0	0	0	1.03	2
6891	NM_000544.3	TAP2	chr6	32793186	32806547	0	0	0	0	0	0	0	0	0	0	0	0	0	0	0	0	1.03	2
5696	NM_148919.3	PSMB8	chr6	32808493	32811816	0	0	0	0	0	0	0	0	0	0	0	0	0	0	0	0	1.03	2

File-Specific Header Description — *ASM/geneVarSummary-[ASM-ID].tsv*

Key	Description	Allowed Values
#ASSEMBLY_ID	Name of the assembly	"`<assembly-name>-ASM`". For example, "GS000000474-ASM".
#DBSNP_BUILD	dbSNP version used for annotation	"`dbSNP build XXX`" where X's are digits. For example, "dbSNP build 130".
#FORMAT_VERSION	Version number of the file format	Two or more digits separated by periods. For example, "0.6".
#GENERATED_AT	Date and time of the assembly	Year-Month-Day Time. For example "2010-Sep-08 20:27:52.457773".
#GENERATED_BY	Assembly pipeline component that generated the output.	Alpha-numeric string.
#GENE_ANNOTATIONS	NCBI annotation build	NCBI build XX.X where X are digits.
#GENOME_REFERENCE	Human genome build used for assembly	NCBI build XX where X are digits.
#SAMPLE	Complete Genomics identifier of the sample from which the library was created	"`GSXXXXX-DNA_YZZ`" where<br>▪ X's are digits<br>▪ `-DNA_` is literal<br>▪ YZZ is the location of the sample in a 96-well plate with Y as one of "A" through "H" and ZZ is one of "`01`" through "`12`"<br>For example "GS12345-DNA_A01".
#SOFTWARE_VERSION	Assembly pipeline build number	Two or more digits separated by periods.

Key	Description	Allowed Values
#TYPE	Indicates the type of data contained in the file.	`GENE-VAR-SUMMARY-REPORT`: summary of genic variations in coding regions of genes.

Content Description *ASM/geneVarSummary-[ASM-ID].tsv*

	Column Name	Description
1	geneId	Entrez Gene Identifier. For example "2597".
2	mrnaAcc	RefSeq mRNA accession number (versioned). For example "NM_002046.3".
3	symbol	NCBI Gene Symbol. For example, "GAPDH".
4	chromosome	Chromosome name in text: `chr1`, `chr2`,…,`chr22`, `chrX`, `chrY`. The mitochondrion is represented as `chrM`. The pseudo-autosomal regions within the sex chromosomes X and Y are reported at their coordinates on chromosome X.
5	begin	Genomic start position of the gene (not the variation).
6	end	Genomic end position of the gene (not the variation).
7	missense	Number of MISSENSE records in the ***gene-[ASM-ID].tsv.bz2*** file for this transcript.
8	nonsense	Number of NONSENSE records in the ***gene-[ASM-ID].tsv.bz2*** file for this transcript.
9	nonStop	Number of NONSTOP records in the ***gene-[ASM-ID].tsv.bz2*** file for this transcript.
10	misStart	Number of MISSTART records in the ***gene-[ASM-ID].tsv.bz2*** file for this transcript.
11	frameshift	Number of FRAMESHIFT records in the ***gene-[ASM-ID].tsv.bz2*** file for this transcript.
12	inframe	Number of INSERT, INSERT+, DELETE, or DELETE+ records in the ***gene-[ASM-ID].tsv.bz2*** file for this transcript.
13	disrupt	Number of DISRUPT records in the ***gene-[ASM-ID].tsv.bz2*** file for this transcript.
14	total	Sum of the *missense*, *nonsense*, *nonstop*, *misStart*, *frameshift*, *inframe*, and *disrupt* columns.
15	missenseNovel	Number of MISSENSE records in the ***gene-[ASM-ID].tsv.bz2*** file for this transcript that are not present in dbSNP.
16	nonsenseNovel	Number of NONSENSE records in the ***gene-[ASM-ID].tsv.bz2*** file for this transcript that are not present in dbSNP.
17	nonStopNovel	Number of NONSTOP records in the ***gene-[ASM-ID].tsv.bz2*** file for this transcript that are not present in dbSNP.
18	misStartNovel	Number of MISSTART records in the ***gene-[ASM-ID].tsv.bz2*** file for this transcript that are not present in dbSNP.
19	frameshiftNovel	Number of FRAMESHIFT records in the ***gene-[ASM-ID].tsv.bz2*** file for this transcript that are not present in dbSNP.
20	inframeNovel	Number of INSERT, INSERT+, DELETE, or DELETE+ records in the ***gene-[ASM-ID].tsv.bz2*** file for this transcript that are not present in dbSNP.
21	disruptNovel	Number of DISRUPT records in the ***gene-[ASM-ID].tsv.bz2*** file for this transcript that are not present in dbSNP.
22	totalNovel	The sum of the *missenseNovel*, *nonsenseNovel*, *nonstopNovel*, *misStartNovel*, *frameshiftNovel*, *inframeNovel*, and *disruptNovel* columns.
23	relativeCvg	Relative coverage (as reported in CNV results) of the region spanned by the gene. If gene spans more than a single CNV segment, relative coverage will be listed for each segment, separated by semicolons (;). Relative coverage entries are sorted by value, rather than the order of the values in the original segments file. Relative coverage is floating-point valued or 'N' if *avgNormalizedCvg* in the original segments file is 'N'.
24	calledPloidy	Copy number (as reported in CNV results) of the region spanned by the gene. If gene spans more than a single CNV segment, called ploidy will be listed for each segments, separated by semicolons (;). Ploidy entries are sorted by value, rather than the order of the values in the original segments file. For normal samples, ploidy is integer valued, with regions of uncertain ploidy labeled 'N'. For tumor samples, this column will be empty, as ploidy is currently not called.

Variations at Known dbSNP Loci

ASM/dbSNPAnnotated-[ASM-ID].tsv.bz2

The ***dbSNPAnnotated-[ASM-ID].tsv.bz2*** file contains all dbSNP entries with fully-defined alleles (i.e., coordinates and exact allele sequence is defined) and the calls that were made for each of the locations in the genome being sequenced. For dbSNP entries that were detected in the 1000 Genomes Project dataset, minor allele and minor allele frequency reported by 1000 Genomes Project is also provided. This information is only included for files annotated with dbSNP version 132. Note "A" and "B" are used to indicate that allele information is present for both chromosomes but does not indicate the origin of the chromosome.

Example *ASM/dbSNPAnnotated-[ASM-ID].tsv.bz2*

The first section shows the first 10 columns; the remaining 16 columns appear in the lower section. The second section of data repeats the *dbSnpId* column at the left edge to more easily match the data with the previous section of data; the *dbSnpId* column is not repeated in the actual data.

>dbSnpId	alleles	chromosome	begin	end	reference	alleleAGenotype	alleleBGenotype	loci	zygosity
dbsnp.132:rs114201130	C/T	chr1	54585	54586	T	T	T	977	hom
dbsnp.100:rs2462492	C/T	chr1	54675	54676	C	C	C	977	hom
dbsnp.132:rs115797567	C/G	chr1	54707	54708	G	G	G	977	hom
dbsnp.130:rs71270700	-/TCTT	chr1	54766	54767	T	NO-MATCH	NO-CALL	977;978	unknown
dbsnp.129:rs59861892	-/CT	chr1	54788	54789	C	NO-MATCH	NO-MATCH	979	unknown
dbsnp.129:rs58014817	A/T	chr1	54794	54795	T	T	T	979	hom
dbsnp.89:rs1645795	G/C	chr1	55037	55038	C	C	C	979	hom
dbsnp.130:rs71258961	A/T	chr1	55084	55085	T	T	T	979	hom
dbsnp.103:rs3091275	A/G	chr1	55130	55131	A	A	G	980	het-ref
dbsnp.103:rs3091274	A/C	chr1	55163	55164	C	A	A	982	hom
dbsnp.119:rs10399749	C/T	chr1	55298	55299	C	C	C	983	hom

© Complete Genomics, Inc.

>dbSnpId	varTypeA	hapA	varScoreVAFA	varScoreEAFA	chromosomeA	beginA	endA	varTypeB	hapB	varScoreVAFB	varScoreEAFB	chromosomeB	beginB	endB	1000GenomesProjectMinorAllele	1000GenomesProjectMAF
dbsnp.132	ref	T			chr1	54585	54586	ref	T			chr1	54585	54586	C	0.056
dbsnp.100	ref	C			chr1	54675	54676	ref	C			chr1	54675	54676	T	0.088
dbsnp.132	ref	G			chr1	54707	54708	ref	G			chr1	54707	54708	C	0.125
dbsnp.130	ref	T			chr1	54766	54767	no-call	?			chr1	54770	54774		
dbsnp.129	ref	C			chr1	54788	54789	ref	C			chr1	54788	54789		
dbsnp.129	ref	T			chr1	54794	54795	ref	T			chr1	54794	54795		
dbsnp.89:	ref	C			chr1	55037	55038	ref	C			chr1	55037	55038		
dbsnp.130	ref	T			chr1	55084	55085	ref	T			chr1	55084	55085		
dbsnp.103	ref	A	88	88	chr1	55130	55131	snp	G	88	88	chr1	55130	55131		
dbsnp.103	snp	A	88	88	chr1	55163	55164	snp	A	88	88	chr1	55163	55164	C	0.125
dbsnp.119	ref	C			chr1	55298	55299	ref	C			chr1	55298	55299	T	0.223

File-Specific Header Description *ASM/dbSNPAnnotated-[ASM-ID].tsv.bz2*

Key	Description	Allowed Values
#ASSEMBLY_ID	Name of the assembly	"<assembly-name>-ASM". For example, "GS000000474-ASM".
#COSMIC	COSMIC version used for annotation	"COSMIC vXX", where X's are digits. For example, "COSMIC v48".
#DBSNP_BUILD	dbSNP version used for annotation	"dbSNP build XXX" where X's are digits. For example, "dbSNP build 130".
#GENOME_REFERENCE	Human genome build used for assembly	NCBI build XX where X are digits.
#SAMPLE	Complete Genomics identifier of the sample from which the library was created	"GSXXXXX-DNA_YZZ" where<br>▪ X's are digits<br>▪ -DNA_ is literal<br>▪ YZZ is the location of the sample in a 96-well plate with Y as one of "A" through "H" and ZZ is one of "01" through "12"<br>For example "GS12345-DNA_A01".
#GENERATED_BY	Assembly pipeline component that generated the output.	Alpha-numeric string.
#GENERATED_AT	Date and time of the assembly	Year-Month-Day Time. For example "2010-Sep-08 20:27:52.457773".
#SOFTWARE_VERSION	Assembly pipeline build number	Two or more digits separated by periods
#FORMAT_VERSION	Version number of the file format	Two or more digits separated by periods. For example, "0.6".
#TYPE	The type of data contained in the file	DBSNP-TO-CGI: information on loci annotated in dbSNP.

Content Description — ASM/dbSNPAnnotated-[ASM-ID].tsv.bz2

	Column Name	Description
1	dbSnpId	Identifier for this dbSNP entry. The format is `[DBNAME].[BUILD]:[ACC#]`, where `DBNAME` currently is `dbsnp` only; `BUILD` indicates the DBNAME build in which this entry first appeared; and `ACC#` is the dbSNP identifier. For example: `dbsnp.129:rs1167318`.
2	alleles	Alleles for the dbSNP entry. For example, "C/T" or "C/-".
3	chromosome	Chromosome name in text: `chr1`, `chr2`,…,`chr22`, `chrX`, `chrY`. The mitochondrion is represented as `chrM`. The pseudo-autosomal regions within the sex chromosomes X and Y are reported at their coordinates on chromosome X.
4	begin	Reference coordinate specifying the start of the dbSNP entry. Uses the half-open zero-based coordinate system. See "Sequence Coordinate System" for more information.
5	end	Reference coordinate specifying the end of the dbSNP entry. Uses the half-open zero-based coordinate system. See "Sequence Coordinate System" for more information.
6	reference	The reference sequence at the locus of the variation.
7	alleleAGenotype	The dbSNP allele (from the *alleles* column) matched to allele 1 of the variations file. The special value `NO-CALL` is used to denote a no-call in the variations file, and `NO-MATCH` is given if the locus was called but did not match any of the dbSNP alleles.
8	alleleBGenotype	The dbSNP allele (from the *alleles* column) matched to allele 2 of the variations file. The special value `NO-CALL` is used to denote a no-call in the variations file, and `NO-MATCH` is given if the locus was called but did not match any of the dbSNP alleles. Additionally, for haploid regions, *alleleBGenotype* is `NO-ALLELE`.
9	loci	A semicolon-separated list of locus IDs from the variations file loci used to determine the *alleleAGenotype* and *alleleBGenotype*. This field corresponds to the first column (*locus*) of the variation file ***var-[ASM-ID].tsv.bz2***.
10	zygosity	The zygosity of the alleleAGenotype and alleleBGenotype. The following values are possible:<br>▪ `unknown`: Either *alleleAGenotype* or *alleleBGenotype* is NO-MATCH.<br>▪ `no-call`: Either both allele genotypes are NO-CALL, or *alleleAGenotype* is NO-CALL and *alleleBGenotype* is NO-ALLELE.<br>▪ `hap`: The *alleleAGenotype* is called, this region is haploid so that *alleleBGenotype* is NO-ALLELE.<br>▪ `half`: One allele has a NO-CALL genotype, but the other allele has a called genotype.<br>▪ `het-ref`: Both alleles have a called genotype, the two genotypes are different, and one genotype is equal to the reference genotype.<br>▪ `het-alt`: Both alleles have a called genotype, the two genotypes are different, and neither genotype is equal to the reference genotype.<br>▪ `hom`: Both alleles have a called genotype, and the genotype is the same for both alleles.
11	varTypeA	A semicolon-separated list of *varType* values from the ***var-[ASM-ID].tsv.bz2*** file, for each call used to determine *alleleAGenotype*. If the list includes more than one element, the prefix "multiple:" is added to the list. See "Variations Type Description."
12	hapA	Sequence of the "A" allele , based on the calls in the variations file. The sequence of reference calls is truncated to match the range of the dbSNP entry.

	Column Name	Description
13	varScoreVAFA	A semicolon-separated list of varScoreVAF values from the ***var-[ASM-ID].tsv.bz2*** file, for each call used to determine *alleleAGenotype*.
14	varScoreEAFA	A semicolon-separated list of *varScoreEAF* values from the ***var-[ASM-ID].tsv.bz2*** file, for each call used to determine *alleleAGenotype*.
15	chromosomeA	Chromosome number where the "A" allele is found.
16	beginA	The *begin* position of the "A" allele, from the ***var-[ASM-ID].tsv.bz2*** file. The ranges of reference calls are truncated to match the range of the dbSNP entry. Uses the half-open zero-based coordinate system. See "Sequence Coordinate System" for more information.<br>The pseudoautosomal regions for the sex chromosomes X and Y are represented by their coordinates on chromosome X
17	endA	The *end* position of the "A" allele, from the ***var-[ASM-ID].tsv.bz2*** file. The ranges of reference calls are truncated to match the range of the dbSNP entry. See "Sequence Coordinate System" for more information.<br>The pseudoautosomal regions for the sex chromosomes X and Y are represented by their coordinates on chromosome X.
18	varTypeB	A semicolon-separated list of *varType* values from the ***var-[ASM-ID].tsv.bz2*** file, for each call used to determine *alleleBGenotype*. If the list includes more than one element, the prefix "multiple:" is added to the list.
19	hapB	Sequence of the "B" allele , based on the calls in the variations file. The sequence of reference calls is truncated to match the range of the dbSNP entry.
20	varScoreVAFB	A semi-colon-separated list of *varScoreVAF* values from the ***var-[ASM-ID].tsv.bz2*** file, for each call used to determine *alleleBGenotype*.
21	varScoreEAFB	A semi-colon-separated list of *varScoreEAF* values from ***the var-[ASM-ID].tsv.bz2*** file, for each call used to determine *alleleBGenotype*.
22	chromosomeB	Chromosome number where the "B" allele is found.
23	beginB	The *begin* position of the "B" allele, from the ***var-[ASM-ID].tsv.bz2*** file. The ranges of reference calls are truncated to match the range of the dbSNP entry. Uses the half-open zero-based coordinate system. See "Sequence Coordinate System" for more information.<br>The pseudoautosomal regions for the sex chromosomes X and Y are represented by their coordinates on chromosome X.
24	endB	The *end* position of the "B" allele, from the ***var-[ASM-ID].tsv.bz2*** file. The ranges of reference calls are truncated to match the range of the dbSNP entry. Uses the half-open zero-based coordinate system. See "Sequence Coordinate System" for more information.<br>The pseudoautosomal regions for the sex chromosomes X and Y are represented by their coordinates on chromosome X.
25	1000GenomesProjectMinorAllele	Minor allele reported by 1000 Genomes Project. This field is empty if the dbSNP entry was not found by 1000 Genomes Project; 'NA' if the dbSNPAnnotated file was annotated by dbSNP v131 or earlier.
26	1000GenomesProjectMAF	Minor allele frequency reported by 1000 Genomes Project. This field is empty if the dbSNP entry was not found by 1000 Genomes Project; 'NA' if the dbSNPAnnotated file was annotated by dbSNP v131 or earlier.

Sequencing Metrics and Variations Summary

ASM/summary-[ASM-ID].tsv

The summary file ***summary-[ASM-ID].tsv*** contains a variety of metrics that may be helpful in assessing the quality of the delivered genome, such as the gross mapping yield and fraction of genome that was fully called. This file also enables the comparison of metrics such as total SNP count, SNP het/hom ratio, and nonsynonomous/synonomous SNP ratio across individuals of the same ethnicity, and determination of whether these metrics are roughly consistent across individuals.

The file contains a header followed by a number of data lines describing the metrics, which are grouped by category. For metrics based on variant count (e.g., total SNP count, missense loci, SNP het/hom ratio, and junction count), two values for each metric are provided.

File-Specific Header Description — *ASM/summary-[ASM-ID].tsv*

Key	Description	Allowed Values
#ASSEMBLY_ID	Name of the assembly	"<assembly-name>-ASM". For example, "GS000000474-ASM".
#SOFTWARE_VERSION	Assembly pipeline build number	Two or more digits separated by periods.
#GENERATED_BY	Assembly pipeline component that generated the output.	Alpha-numeric string.
#GENERATED_AT	Date and time of the assembly	Year-Month-Day Time. For example "2010-Sep-08 20:27:52.457773".
#FORMAT_VERSION	Version number of the file format	Two or more digits separated by periods. For example, "0.6".
#GENOME_REFERENCE	Human genome build used for assembly	"NCBI build XX" where X's are digits.
#SAMPLE	Complete Genomics identifier of the sample from which the library was created	"GSXXXXX-DNA_YZZ" where<br>▪ X's are digits<br>▪ -DNA_ is literal<br>▪ YZZ is the location of the sample in a 96-well plate with Y as one of "A" through "H" and ZZ is one of "01" through "12"<br>For example "GS12345-DNA_A01".
#DBSNP_BUILD	dbSNP version used for annotation	"dbSNP build XXX" where X's are digits. For example, "dbSNP build 130".
#GENE_ANNOTATIONS	NCBI annotation build	"NCBI build XX.X" where X's are digits.

Content Description ASM/summary-[ASM-ID].tsv

The columns in the data section are described here, followed by a table that describes each metric.

Column	Description
Category	Metric category. For example, Genome coverage or Library.
Metric	Metric. Each metric is described in the following table.
Value	Value pulled from more detailed data source.
CallConfidence	Variants used to calculate the metric.<br>▪ ALL: Metric is calculated using all variants detected.<br>▪ HIGH: Metric is calculated using high-confidence variants. For small variant calls, *CallConfidence* includes all variants that are not designated VQLOW in the *varFilter* column of the ***var-[ASM-ID].tsv.bz2*** file. High confidence junctions are those reported in the ***highConfidenceJunctionsBeta-[ASM-ID].tsv*** file.<br>▪ NA: Metric is not based on variant count. For example gross mapping yield and mate distribution mean.

Metric Name	Description
**Category: Miscellaneous**	
▪ Gender	Gender of the sample as determined by presence or absence of Y chromosome.
**Category: Genome coverage**	
▪ Fully called genome fraction	Fraction of the reference bases where all alleles were called.
▪ Partially called genome fraction	Fraction of the reference bases where one allele out of two was called.
▪ No-called genome fraction	Fraction of the reference bases where all alleles were no-called.
▪ Gross mapping yield (Gb)	Count of called bases within DNB arms with at least one initial mapping to the reference genome. This excludes reads marked as overflow (large number of mappings to the reference genome indicative of highly repetitive sequence). In the case of a DNB with only one arm mapped to the reference, only the mapped bases contribute to this statistic. This is the sum of the *grossWeightSumSequenceCoverage* counter in the "Coverage and Reference Scores" files.
▪ Both mates mapped yield (Gb)	Count of called bases within DNBs where both arms mapped to the reference genome on the correct strand and orientation, and within the expected distance. This is the sum of the *uniqueSequenceCoverage* counter in the "Coverage and Reference Scores" files.
▪ 100k normalized coverage variability	A measure of noise in the normalized coverage data for 100K windows. To compute this metric, the 100K normalized coverage is split into 3 Mb buckets. For each bucket, the expected-coverage value is computed as the mean of the coverage values for the bucket. Then 20% of the buckets with lowest mean value are rejected and 40% of the remaining buckets with highest standard deviation in coverage/expected coverage are rejected. Based on the remaining buckets, the standard deviation of coverage/expected coverage is returned.<br>**Note**: Genomes with 100K normalized coverage variability > 0.04 have no-called CNVs.
▪ Genome fraction where *weightSumSequenceCoverage* >= 5x	Fraction of the reference bases where the corresponding coverage column in the "Coverage and Reference Scores" files is greater than 5. There are also metrics for cutoff values of 10, 20, 30 and 40.
**Category: Exome coverage**	
▪ Fully called exome fraction	Fraction of the reference bases of the exome where all alleles were called.
▪ Partially called exome fraction	Fraction of the reference bases of the exome where one allele out of two was called.
▪ No-called exome fraction	Fraction of the reference bases of the exome where all alleles were no-called.

Metric Name	Description
**Category: Exome coverage (continued)**	
▪ Exome fraction where *weightSumSequenceCoverage* >= 5x	Fraction of the reference bases of the exome where the corresponding coverage column in the "Coverage and Reference Scores" files is greater than 5. There are also metrics for cutoff values of 10, 20, 30 and 40.
**Category: Library**	
▪ Mate distribution mean	Mean mate gap estimated for the library.
▪ Mate distribution range (.95 confidence interval) min	Lower boundary of the range of mate gap that captures 95% of the data.
▪ Mate distribution range (.95 confidence interval) max	Upper boundary of the range of mate gap that captures 95% of the data.
**Category: Genome variations**	
▪ SNP total count	Number of fully or partially called SNP loci.
▪ Homozygous SNP count	Number of fully called homozygous SNP loci.
▪ Heterozygous SNP count	Number of fully called heterozygous SNP loci.
▪ SNP novel fraction	Fraction of SNPs not found in version of dbSNP indicated in header.
▪ Homozygous SNP novel fraction	Fraction of homozygous SNPs not found in version of dbSNP indicated in header.
▪ Heterozygous SNP novel fraction	Fraction of heterozygous SNPs not found in version of dbSNP indicated in header.
▪ SNP heterozygous/ homozygous ratio	Ratio of fully called heterozygous to homozygous SNP loci.
▪ SNP transitions/ transversions ratio	Ratio of transition to transversion SNP allele count.
▪ INS total count	Number of fully or partially called insertion loci.
▪ INS novel fraction	Fraction of insertions not found in version of dbSNP indicated in header.
▪ INS heterozygous/ homozygous ratio	Ratio of fully called heterozygous to homozygous insertion loci.
▪ DEL total count	Number of fully or partially called deletion loci.
▪ DEL novel fraction	Fraction of deletions not found in version of dbSNP indicated in header.
▪ DEL heterozygous/ homozygous ratio	Ratio of fully called heterozygous to homozygous deletion loci.
▪ SUB total count	Number of fully or partially called substitution loci.
▪ SUB novel fraction	Fraction of substitutions not found in version of dbSNP indicated in header.
▪ SUB heterozygous/ homozygous ratio	Ratio of fully called heterozygous to homozygous substitution loci.
**Category: Exome variations**	
Multiple metrics	Same as the corresponding metric from the "Genome variations" category, restricted to loci within the exome.
**Category: Functional impact**	
▪ Synonymous SNP loci	Number of loci where the single nucleotide change in coding sequence did not result in protein sequence change.
▪ Non-synonymous SNP loci	Number of loci where the single nucleotide change in coding sequence did result in protein sequence change. Non-synonymous SNP loci is the sum of missense, nonsense, nonstop, and misstart SNP loci.
▪ Missense SNP loci	Number of loci where the single nucleotide change in coding sequence resulted in protein sequence change, with no change in size of protein.

Metric Name	Description
**Category: Functional impact (continued)**	
▪ Nonsense SNP loci	Number of loci where the single nucleotide change in coding sequence resulted in a STOP codon (TGA, TAG, or TAA), causing an early termination of protein translation.
▪ Nonstop SNP loci	Number of loci where the single nucleotide change in coding sequence resulted in the change of a STOP codon (TGA, TAG, or TAA) into a codon that codes for an amino acid, resulting in the continuation of the translation for this protein.
▪ Misstart SNP loci	Number of loci where the single nucleotide change in coding sequence resulted in the change of a START codon into a codon for something other than a start codon, likely resulting in a non-functional gene.
▪ Disrupt SNP loci	Number of loci where the single nucleotide change in the GT or AG conserved donor and acceptor splice site (or rare AT/AC) sequence resulted in a change to something that is incompatible.
▪ Frame-shifting INS loci	Number of insertion loci where the change in coding sequence resulted in a frameshift for the encoded protein.
▪ Frame-shifting DEL loci	Number of deletion loci where the change in coding sequence resulted in a frameshift for the encoded protein.
▪ Frame-shifting SUB loci	Number of substitution loci where the change in coding sequence resulted in a frameshift for the encoded protein.
▪ Frame-preserving INS loci	Number loci where there is a change in coding sequence and the length of the insertion is a multiple of 3, resulting in the insertion of amino acids in the encoded protein in-frame.
▪ Frame-preserving DEL loci	Number loci where there is a change in coding sequence and the length of the deletion is a multiple of 3, resulting in the deletion of amino acids in these encoded protein in-frame.
▪ Frame-preserving SUB loci	Number loci where there is a change in coding sequence and the length of the substitution is a multiple of 3, resulting in the substitution of amino acids in the encoded protein in-frame.
**Category: CNV**	
▪ Total CNV segment count	For normal genomes, number of contiguous segments of the reference that were called with copy number different from the expected (that is, the number of gain or loss segments in the genome) using the diploid model for CNV analysis. For tumor genomes, the number of contiguous segments reported for the genome regardless of coverage level, using the non-diploid model for CNV analysis.
▪ Total number of bases in CNV segments	For normal genomes, the number of bases in the segments called as gain or loss in normal genomes, using the diploid model for CNV analysis. For tumor genomes, the number is not calculated and value for this field is NA.
▪ Fraction of novel CNV (by segment count)	For normal genomes, the fraction of gain or loss CNV segments that do not overlap any known events in DGV database. For tumor genomes, this number is not calculated and value for this field is 'NA'.
▪ Fraction of novel CNV (by base count)	For normal genomes, the fraction of bases in gain or loss CNV segments that don't overlap any known events in DGV database to the total number of bases in the gain or loss CNV segments. For tumor genomes, this number is not calculated and value for this field is 'NA'.
**Category: SV**	
▪ Total junction count	Total number of junction events (as listed in *allJunctionsBeta* file).
**Category: MEI**	
▪ Mobile element insertion count	Total number of mobile element insertion events (as listed in the ***mobileElementInsertionsBeta*** file).
▪ Fraction of novel MEI	The fraction of mobile element insertions that do no overlap known events detected by 1000 Genomes Project.

Copy Number Variation Files

The CNV Directory contains information regarding copy number variation along the reference genome, based on depth of coverage. CNV reporting is provided in CNV segmentation files and CNV details files. For each genome, results from both diploid and non-diploid CNV models are provided. Additional supporting evidence consists of mappings and coverage data, as described in "Reads and Mapping Data" and "Coverage and Reference Scores").

Figure 6: CNV Directory Contents

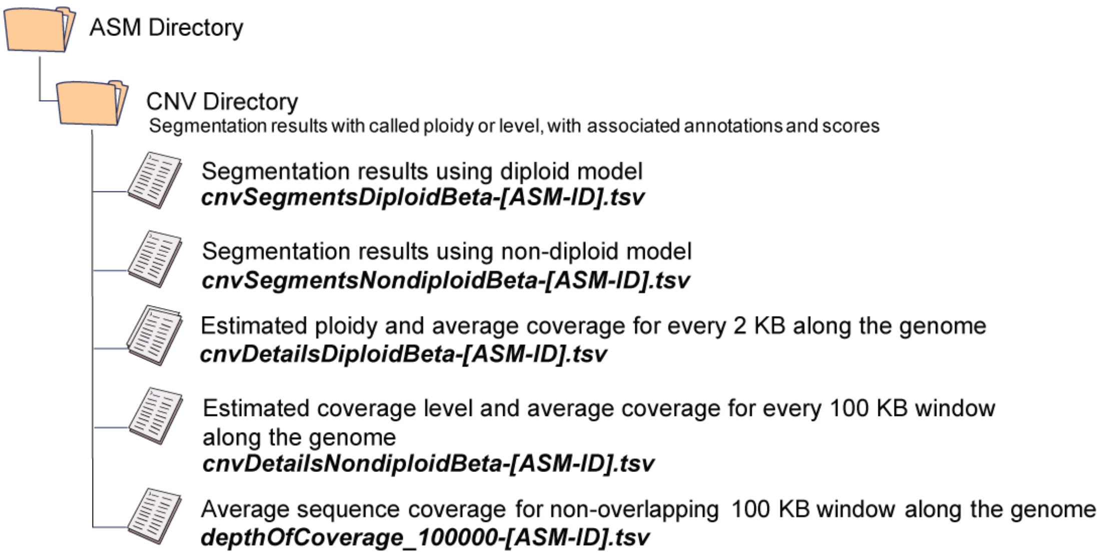

The CNV calls are based on an analysis of coverage that can be described in seven steps:

1. Computation of sequence coverage
2. Modeling of GC bias
3. GC bias correction
4. Coverage smoothing
5. Normalization of coverage by comparison to baseline values
6. Segmentation and scoring with a hidden Markov model (HMM) model
7. Annotation of called segments by CNV type (including 'no-calling'), overlapping genes, repeats, and known CNV in the Database of Genomic Variants (DGV).

The CNV analysis does not at this time take into account allele balance or mate pair-based evidence of structural variations.

The coverage computation used for CNV estimation takes into account the uniqueness and confidence of each mapping; mappings are given fractional weights corresponding to the confidence that a mapping correctly identifies the source of the mapped DNB.

Sequence coverage is averaged and corrected for GC bias over sliding windows across the genome, and normalized relative to a set of standard genomes. The files containing coverage information and normalization factors for the baseline genome set, along with the document listing the genomes used for the baseline set and the processing steps applied are available for download. See CNV Baseline Genome Dataset. The window width and window shift used for coverage smoothing can be found in the header of the file on the lines which begin with `#WINDOW_WIDTH` and `#WINDOW_SHIFT`, respectively. Changes in copy number shorter than the length of the smoothing window may either be missed or be interpreted as a

change to a longer segment than is actually present. In the latter case, the called copy number may be less extreme than the true change. Boundaries of segments are approximate, with uncertainty on the order of the length of the window shift.

The remainder of this section describes methods and file formats for CNV calling under the assumption that a sample is diploid (i.e., the diploid model pipeline assumes that most of the genome is diploid). See "Genomic Copy Number Analysis of Non-Diploid Samples" for description of modifications made for CNV calling, assuming samples are non-diploid.

The HMM model classifies segments of the genome as having 0 copies, 1 copy, 2 copies, 3 copies, … up to a maximum value. Segments with true ploidy higher than the maximum reportable value are assigned the maximum value. The maximum reported ploidy can be found in the header of the CNV files on the line which begins with `#MAX_PLOIDY`.

The coverage model employed by the HMM makes the assumptions that

- copy number is integer-valued
- changes in copy number can be attributed to a single location in the genome
- the sample is homogeneous

These assumptions may be incorrect in repeat or segmental duplication regions of the genome, where, for example, a heterozygous increase of one copy in a region present as two-copy in the reference may appear as a half-copy increase on each of the reference copies. They may also be incorrect in a tumor sample with normal tissue contamination or copy-number heterogeneity within the tumor. In either situation, the resulting copy number calls may not be optimal. Regions where coverage is not well-behaved in a set of standard genomes are assigned *ploidy*='N'; among such regions, we distinguish 'hypervariable' segments where coverage of the sequenced and baseline genomes varies considerably without clear clustering into distinct copy number categories, and 'invariant' segments where coverage of the sequenced and baseline genomes is not consistent with the reference (such as two copies in autosomal regions) but is highly consistent across the set of standard genomes. Because assignment of 'hypervariable' and 'invariant' segments is done based in part on the coverage of the sample of interest, the portion of the genome labeled 'hypervariable' or 'invariant' can differ from sample to sample.

Copy Number Segmentation

ASM/CNV/cnvSegmentsDiploidBeta-[ASM-ID].tsv

The copy number segments file, ***cnvSegmentsDiploidBeta-[ASM-ID].tsv***, provides a segmentation of the complete reference genome into regions of distinct ploidy levels, giving the estimated ploidy, the average and relative adjusted coverage for each segment, and measures of confidence in the called segments.

Example — *ASM/CNV/cnvSegmentsDiploidBeta-[ASM-ID].tsv*

The following example shows the kinds of variations identified in the variations file:

- The first segment, starting at position `17083000` of `chr1`, is called tetraploid (*calledPloidy* is `4`); *calledCNVType* is '+' because this is more than the nominal expectation of 2 copies in the autosome. Average coverage is over 100x, compared to approximately 50x in most of the genome (data not shown). The *ploidyScore*, `11`, is less than the *CNVTypeScore*, `35`, indicating that confidence is considerably higher that this is a region of copy number increase than that there are exactly 4 copies.
- The second segment, starting at position `17153000` of `chr1`, is called diploid and average coverage is slightly over 50. Because this is the nominally expected coverage for autosomal segments, the segment has *calledCNVType* '='. The *ploidyScore* matches the *CNVTypeScore* because only ploidy 2 corresponds to this *calledCNVType*.
- The third segment, starting at position `56597000` of `chr1`, is called "haploid"; *calledCNVType* is `'-'` because this is less than the nominally expected value. The *ploidyScore*, `4`, indicates quite low confidence, and the *CNVTypeScore* is the same. These scores reflect the fact that average coverage in the segment is ~33x, considerably below the ~50x seen over most of the genome for the genome but also considerably higher than the ~25x that might be expected of an obviously haploid region, and the probability of homozygous loss is negligible, so we are scarcely more confident that the region has either 0 or 1 copies (the hypothesis evaluated by the *CNVTypeScore*) than that it has exactly 1 copy.
- The fourth segment, starting at position `56613000`, is called "invariant". Coverage is low, approximately as expected of a haploid segment. The `'invariant'` designation indicates that the sequenced genome and all of a standard set of genomes had coverage similar to one another but implied a copy number different than what would be expected based on the reference genome.
- The last segment, starting at position `58610000`, is called "hypervariable". Coverage is near the expected level for a diploid segment (non-CNV), but coverage in the sequenced genome and a set of standard genomes was more variable than is typical of normal regions, without resolving into clear clusters of distinct ploidy. Such regions typically overlap segmental duplications, satellite regions, or short tandem repeats; in such regions, we do not call a discrete copy number.
- The *ploidyScore* and *CNVTypeScore* values are Phred-like scores. A score of `0` means effectively zero confidence; larger values mean more confidence. They are computed as $-10*\log_{10}$ of the probability of the assigned call being wrong, though due to differences between reality and the model, they may not give quantitatively reliable measures of probabilities. Scores for segments are computed such that they are the average of the scores for the constituent detail positions.

>chr	begin	end	avgNormalizedCvg	relativeCvg	calledPloidy	calledCNVType	ploidyScore	CNVTypeScore	overlappingGene	knownCNV	repeats
chr1	17083000	17153000	103.1	2.1	4	+	11	35	KIAA0445;MSTP9	dgv.9:Variation_34489;dgv.9:Variation_3284…	DNA:1;LINE:19;LTR:3…
chr1	17153000	56597000	51.5	1	2	=	29	29			
chr1	56597000	56613000	33.2	0.7	1	-	4	4			DNA:1;LINE:12;LTR:22…
chr1	56613000	56617000	19.7	0.4	N	invariant	0	0			
chr1	56617000	58610000	48.4	1	2	=	28	28			
chr1	58610000	58620000	44.5	0.9	N	hypervariable	0	0			

File-Specific Header Description *ASM/CNV/cnvSegmentsDiploidBeta-[ASM-ID].tsv*

Key	Description	Allowed Values
#ASSEMBLY_ID	Name of the assembly	"<assembly-name>-ASM". For example, "GS000000474-ASM".
#FORMAT_VERSION	Version number of the file format	Two or more digits separated by periods. For example, "0.6".
#GENERATED_AT	Date and time of the assembly	Year-Month-Day Time. For example "2010-Sep-08 20:27:52.457773".
#GENERATED_BY	Assembly pipeline component that generated the output.	Alpha-numeric string.
#GENOME_REFERENCE	Human genome build used for assembly	"NCBI build XX" where X's are digits.
#MAX_PLOIDY	Maximum allowed copy number estimate.	Positive integer.
#SAMPLE	Complete Genomics identifier of the sample from which the library was created	"GSXXXXX-DNA_YZZ" where<br>▪ X's are digits<br>▪ -DNA_ is literal<br>▪ YZZ is the location of the sample in a 96-well plate with Y as one of "A" through "H" and ZZ is one of "01" through "12"<br>For example "GS12345-DNA_A01".
#SOFTWARE_VERSION	Assembly pipeline build number	Two or more digits separated by periods.
#TYPE	Indicates the type of data contained in the file.	CNV-SEGMENTS: segmentation of the reference genome into regions of distinct ploidy.
#WINDOW_SHIFT	Shift, in bases, between consecutive windows in which smoothed coverage is calculated for copy number estimation.	Positive integer.
#WINDOW_WIDTH	Width, in bases, of windows in which smoothed coverage is calculated for copy number estimation.	Positive integer.
#GENE_ANNOTATIONS	NCBI annotation build	"NCBI build XX.X" where X's are digits.
#DGV_VERSION	DGV version used for annotation	"X", where X is a digit.

Content Description ASM/CNV/cnvSegmentsDiploidBeta-[ASM-ID].tsv

	Column Name	Description
1	chr	Chromosome name in text: `chr1`, `chr2`,…, `chr22`, `chrX`, `chrY`. The mitochondrion is represented as `chrM`, though this may be absent from CNV analyses. The pseudoautosomal regions within the sex chromosomes X and Y are reported at their coordinates on chromosome X.
2	begin	Beginning of segment.
3	end	End of segment.
4	avgNormalizedCvg	Baseline-normalized average coverage over the interval from *begin* to *end*. *avgNormalizedCvg* is no-called ('N') in regions of the genome with very high or very low GC content as well as in regions with very low average coverage among the baseline samples. See further description in "Detailed Ploidy and Coverage Information."
5	relativeCvg	*avgNormalizedCvg* divided by estimate of diploid median average adjusted coverage. Value is 'N' if *avgNormalizedCvg* is 'N'.
6	calledPloidy	Called ploidy for the segment. Typically an integer in the range [`0`,`1`,…,`MAX_PLOIDY`]; 'N' when *calledCNVType* is 'invariant', 'hypervariable', or 'N'.
7	calledCNVType	Classification of called ploidy to one of six categories:<br>▪ `-` (hyphen): a reduction in copy number relative to the nominal expectation (diploid for autosomes, sex-appropriate for sex chromosomes).<br>▪ `=` (equal): a match to the nominal expectation.<br>▪ `+` (plus): an increase relative to the nominal expectation.<br>▪ `invariant`: a change relative to the nominal expectation but in a fashion observed to be present in the sequenced genome and all of a collection of 'standard' genomes, indicating that the reference genome represents a rare alternative in this region or is simply wrong.<br>▪ `hypervariable`: coverage not interpretable as a discrete ploidy due to high diversity of coverage levels in the sequenced genome and a set of 'standard' genomes.<br>▪ `N`: whole genome coverage has been 'no-called'; see 100k normalized coverage variability in the content description at "Sequencing Metrics and Variations Summary".
8	ploidyScore	Phred-like confidence that the segment has the called ploidy.
9	CNVTypeScore	Phred-like confidence that the *calledCNVType* is correct.
10	overlappingGene	Gene(s) overlapping called segment, with minimum overlap of a single base pair.
11	knownCNV	Known CNVs in the Database for Genomic Variants that overlap called segment. Overlap requires that the CNV segment in DGV covers at least 80% of Complete Genomics called CNV segment, allowing a single-window error in the boundary on each side of the called segment. Format:<br>`dgv.<version>:Variation_XXX`<br>with multiple entries separated by the semicolon (;). `version` indicates in which version of DGV this entry first appeared.
12	repeats	Percent of called CNV segment that overlaps with each category of genomic repeats. Categories include: DNA, LINE, Low_Complexity, SINE, Satellite, SegDup, Self-chain, Simple_Repeats, scRNA, tRNA, and snRNA. If the amount of overlap for a category is less than 1%, category is not reported. Format:<br>`Repeat category:XX`<br>With multiple entries separated by the semicolon (`;`). XX represents percent of called CNV segment that overlaps with indicated repeat category.

© Complete Genomics, Inc.

Detailed Ploidy and Coverage Information

ASM/CNV/cnvDetailsDiploidBeta-[ASM-ID].tsv.bz2

The ***cnvDetailsDiploidBeta-[ASM-ID].tsv.bz2*** file provides information on estimated ploidy and average coverage for every 2 kb along the genome.

Example *ASM/CNV/cnvDetailsDiploidBeta-[ASM-ID].tsv.bz2*

The example shows information typical of the ***cnvDetailsDiploidBeta-[ASM-ID].tsv.bz2*** file:

- The first row indicates that the region of length `WINDOW_WIDTH` with begin and end position of `150000` and `152000` of `chr1` has average corrected coverage of ~39X; this region is called diploid, with a ploidyScore of `23`, which indicates reasonably high confidence in the called ploidy.
- There is a larger difference between the positions in the ninth and tenth rows (with begin positions of `166000` and `217280`) than between other pairs of adjacent rows. This reflects the presence of a gap between contigs in the NCBI human reference (build 37), and the impact of this gap on sliding window coverage smoothing. Similar gaps between segments in the ***cnvSegments*** file can occur.
- Rows reporting windows with begin positions `158000` to `217280` have *calledPloidy*=`3`. The called ploidy is given a low score, and the *CNVTypeScore* is the same as the *ploidyScore*, despite the fact that the average coverage for several of these rows is in the range of 75-80x, right around what would be expected of a triploid region in a genome where diploid regions are typically ~50x. This is because there is an alternative explanation according to which the entire region is diploid and the elevated coverage is noise or bias in sequencing rather than a true copy number variation; the HMM model indicates that this alternative is not the most likely interpretation, but it is likely enough to give reduced confidence in the called copy number increase.
- The second-to-last row has ploidy '`N`' and *calledCNVType* '`hypervariable`'. Coverage for this interval is highly diverse in the sequenced genome and a set of 'standard' genomes, so no discrete assignment of copy number is made. The *ploidyScore* and *CNVTypeScore* are `0` to signal the unspecified copy number assignment.
- The last row has *ploidy* '`N`' and *calledCNVType* '`invariant`'. Coverage for this interval is consistently different from the nominally expected (diploid) value across a set of 'standard' genomes, so no discrete assignment of copy number is made. The *ploidyScore* and *CNVTypeScore* are `0` to signal the unspecified copy number assignment.
- The *ploidyScore* and *CNVTypeScore* values are Phred-like scores. A score of `0` means effectively zero confidence, and larger values mean more confidence. They are computed as $-10*\log_{10}$ of the probability of the assigned call being wrong, though due to differences between reality and the model, they may not give quantitatively reliable measures of probabilities. Scores for segments are computed such that they are the average of the scores for the constituent detail positions.

© Complete Genomics, Inc.

>chr	begin	end	avgNormalizedCvg	gcCorrectedCvg	fractionUnique	relativeCvg	calledPloidy	calledCNVType	ploidyScore	CNVTypeScore
chr1	150000	152000	38.7	34.1	0.31	0.85	2	=	23	23
chr1	152000	154000	39	37.8	0.19	0.81	2	=	21	21
chr1	154000	156000	38.5	42.1	0.16	0.87	2	=	19	19
chr1	156000	158000	38.4	35.2	0.09	0.85	2	=	14	14
chr1	158000	160000	80.6	69	0.17	1.69	3	+	5	5
chr1	160000	162000	80	61	0.05	1.63	3	+	7	7
chr1	162000	164000	79.9	66.5	0.11	1.61	3	+	7	7
chr1	164000	166000	75.2	54.3	0.08	1.51	3	+	7	8
chr1	166000	167280	70.6	53.8	0.11	1.44	3	+	5	5
chr1	217280	220000	63.9	41.4	0.11	1.37	3	+	3	3
chr1	220000	222000	60.1	50	0.13	1.28	2	=	5	5
chr1	222000	224000	57.9	48.7	0.31	1.23	2	=	9	9
chr1	224000	226000	55.6	59.5	0.19	1.19	2	=	13	13
chr1	226000	228000	54.8	35.7	0.16	1.13	N	hypervariable	0	0
chr1	228000	230000	27.3	16.1	0.25	0.66	N	invariant	0	0

File-Specific Header Description *ASM/CNV/cnvDetailsDiploidBeta-[ASM-ID].tsv.bz2*

Key	Description	Allowed Values
#ASSEMBLY_ID	Name of the assembly	"<assembly-name>-ASM". For example, "GS000000474-ASM"
#FORMAT_VERSION	Version number of the file format	Two or more digits separated by periods. For example, "0.6"
#GENERATED_AT	Date and time of the assembly	Year-Month-Day Time. For example "2010-Sep-08 20:27:52.457773"
#GENERATED_BY	Assembly pipeline component that generated the output	Alpha-numeric string
#GENOME_REFERENCE	Human genome build used for assembly	"NCBI build XX" where X's are digits
#MAX_PLOIDY	Maximum allowed copy number estimate	Positive integer
#SAMPLE	Complete Genomics identifier of the sample from which the library was created	"GSXXXXX-DNA_YZZ" where<br>▪ X's are digits<br>▪ -DNA_ is literal<br>▪ YZZ is the location of the sample in a 96-well plate with Y as one of "A" through "H" and ZZ is one of "01" through "12"<br>For example "GS12345-DNA_A01"
#SOFTWARE_VERSION	Assembly pipeline build number	Two or more digits separated by periods
#TYPE	Indicates the type of data contained in the file	CNV-DETAIL-SCORES: estimated ploidy for every WINDOW_WIDTH non-overlapping window along the genome
#WINDOW_SHIFT	Shift, in bases, between consecutive windows in which smoothed coverage is calculated for copy number estimation	Positive integer
#WINDOW_WIDTH	Width, in bases, of windows in which smoothed coverage is calculated for copy number estimation	Positive integer

Content Description ASM/CNV/cnvDetailsDiploidBeta-[ASM-ID].tsv.bz2

	Column Name	Description
1	chr	Chromosome name in text: `chr1`, `chr2`,…, `chr22`, `chrX`, `chrY`. The mitochondrion is represented as `chrM`, though this may be absent from CNV analyses. The pseudoautosomal regions within the sex chromosomes X and Y are reported at their coordinates on chromosome X.
2	begin	Beginning of the window being described. For most of the genome, chromosome coordinates are even multiples of window length (for example, for 2K windows, window boundaries will end with "x000", where x is an even digit). Exceptions to this are windows at the ends of contigs. Windows will never span bases taken from more than one contig, even if the gap between contigs is small enough to permit this. Bases outside the outermost full default windows for each contig will either be added to the first full window towards the center of the contig or be placed in their own window, depending on whether the number of bases is larger than half the window width or not.
3	end	End of the window being described. See also information for *begin* column.
4	avgNormalizedCvg	Baseline-normalized average coverage of a window of width `WINDOW_WIDTH`. This is the value that is ultimately used to estimate ploidy; *avgNormalizedCvg* is derived from *gcCorrectedCvg* by normalization against other genomes. *avgNormalizedCvg* is no-called ('N') in regions of the genome with very high or very low GC content, as well as in regions with very low average coverage among the baseline samples.
5	gcCorrectedCvg	GC-corrected average coverage of a window of width `WINDOW_WIDTH`. The *gcCorrectedCvg* is no-called ('N') in regions of the genome with very high or very low GC content.
6	fractionUnique	Fraction of coverage due to unique mappings.
7	relativeCvg	*avgNormalizedCvg* divided by estimate of diploid median normalized adjusted coverage. Value is 'N' if *avgNormalizedCvg* is 'N'.
8	calledPloidy	Called ploidy for segment. Typically an integer in [`0`,`1`,…,`MAX_PLOIDY`]; 'N' when *calledCNVType* is 'invariant', 'hypervariable', or 'N'.
9	calledCNVType	Classification of called ploidy to one of six categories:<br>▪ `-` (hyphen): a reduction in copy number relative to the nominal expectation (diploid for autosomes, sex-appropriate for sex chromosomes).<br>▪ = (equal): a match to the nominal expectation.<br>▪ + (plus): an increase relative to the nominal expectation.<br>▪ `invariant`: a change relative to the nominal expectation in a fashion observed to be present in the sequenced genome and all of a collection of 'standard' genomes, indicating that the reference genome represents a rare alternative in this region or is simply wrong.<br>▪ `hypervariable`: coverage not interpretable as a discrete ploidy due to high diversity of coverage levels in the sequenced genome and a set of 'standard' genomes.<br>▪ N: whole genome coverage has been 'no-called'; see "100k normalized coverage variability" in the content description at "Sequencing Metrics and Variations Summary".
10	ploidyScore	Phred-like confidence that the segment has the called ploidy.
11	CNVTypeScore	Phred-like confidence that the *calldCNVType* is correct.

Genomic Copy Number Analysis of Non-Diploid Samples Files

Files described:

- Non-diploid CNV Segments: ***ASM/CNV/cnvSegmentsNondiploidBeta-[ASM-ID].tsv.bz2***
- Detailed Non-Diploid Coverage Level Information: ***ASM/CNV/cnvDetailsNondiploidBeta-[ASM-ID].tsv.bz2***

For non-diploid samples, CNV calling is modified from what is described for diploid samples in "Copy Number Variation."

The non-diploid model provides more accurate segmentation of samples with a large number of genomic copy number aberrations, as in the case of many tumors. The current non-diploid segmentation algorithm allows for normal contamination (presence of DNA from normal cells in the "tumor" sample) and/or tumor heterogeneity. Concretely, the inferred coverage levels are unconstrained, i.e., not forced to correspond to integer ploidy levels. Instead, a preliminary analysis of the data is done to identify discrete coverage levels: an initial set of levels is chosen based on the distribution of observed normalized coverage values. The initial set of levels is refined by a model selection process which tests alternative models by scoring the genome with an HMM, iteratively removing and adding levels to the model. Once the final set of levels is determined, the resulting HMM is used to segment the genome into regions assigned to the identified levels.

The called levels are identified by their coverage relative to the median of the portion of the genome nominally expected to be diploid (autosomes for male, autosomes+X for female). Thus, the results describe segments of the genome as floating-point values, with values > 1 being amplified relative to the sample median and values < 1 being reduced relative to the sample median. This method contrasts to the reporting for normal samples, for which segments are attributed a specific ploidy. Further, called levels are not identified as "amplified" or "reduced", as we cannot be sure what level corresponds to an unmodified state without further interpretation.

Sufficient heterogeneity in a tumor may make it difficult to correctly identify all the relevant coverage levels, and excessive normal contamination may make differences in ploidy within the tumor portion of a sample lead to differences in coverage that are too small to be reconstructed, even if the tumor is itself homogeneous. To provide reasonable results for most tumors in light of the possibility of narrowly separated coverage levels (normal contamination) and the lack of constraints on the spacing of allowed coverage levels (tumor heterogeneity), longer windows are used for coverage smoothing of tumors as compared to calling of normal samples.

Changes in coverage level shorter than the length of the smoothing window may either be missed or be interpreted as a change to a longer segment than is actually present. In the latter case, the called level may be less extreme than the true change. Boundaries of segments are approximate, with uncertainty on the order of the length of the window shift.

Lesser allele fraction (LAF), the fraction of copies of the less abundant haplotype in a specific region, provides information regarding allelic ratios. It may help identify regions of Loss of Heterozygosity (LOH) or provide another dimension on which to assess copy number change or tumor heterogeneity. For single samples, LAF is estimated based on allele-specific read counts at all fully-called variant loci. Estimates require information from several truly heterozygous loci, or from the absence of heterozygous signal for many consecutive variant loci, in order to be robust; for this reason, single-sample LAF estimates are only provided for the non-diploid CNV files, which provide information for longer windows than the diploid CNV files.

Non-diploid CNV Segments

ASM/CNV/cnvSegmentsNondiploidBeta-[ASM-ID].tsv.bz2

The non-diploid CNV segments file provides a segmentation of the complete reference genome into regions of distinct coverage levels, the average and relative adjusted coverage for each segment, and measures of confidence in the called segments.

Example *ASM/CNV/cnvSegmentsNondiploidBeta-[ASM-ID].tsv.bz2*

The example shows information typical of the ***cnvSegmentsNondiploidBeta-[ASM-ID].tsv.bz2*** file:

- The header rows indicate that the sample was modeled with five coverage levels, with means 0.029, 0.515, 1.004, etc.
- The first example segment, starting at position 89830436 of chr2, has average coverage of 68.4. The relative coverage is 1.01 (calculated as average coverage of this segment divided by the average coverage for the genome (data not shown)). The segment is called at coverage level 1.004, in close agreement with the observed relative coverage. The *levelScore* of 862 indicates a high degree of confidence in the called level for this segment. *calledCNVType* and *CNVTypeScore* are always set to "NA" in this file.
- The second row, starting at position 90371525 of chr2, is called at level 3.476; average coverage is 278.1.
- The fourth row, starting at position 91900000 of chr2, is called at level 1.854, with average coverage of 112.9.
- The difference in levels among the segments (1.004 vs 3.476 vs 1.854) is not a statement of the difference in absolute ploidy.
- The *levelScore* values are Phred-like scores. A score of 0 means effectively zero confidence; larger values mean more confidence. They are computed as -10*$\log_{10}$ of the probability of the assigned call being wrong, though due to differences between reality and the model, they may not give quantitatively reliable measures of probabilities. Scores for segments are computed such that they are the average of the scores for the constituent detail positions.

The data is broken into sections to show all the columns. The second section repeats the *chr*, *begin*, and *end* columns at the left edge to more easily match the data with the previous section of data; these columns are not repeated in the actual data.

#MEAN_LEVEL_0		0.029					
#MEAN_LEVEL_1		0.515					
#MEAN_LEVEL_2		1.004					
#MEAN_LEVEL_3		1.854					
#MEAN_LEVEL_4		3.476					
#NUMBER_LEVELS		5					
>chr	begin	end	avgNormalizedCvg	relativeCvg	calledLevel	calledCNVType	levelScore
chr2	89830436	90321525	68.4	1.01	1.004	NA	862
chr2	90371525	90545103	278.1	4.09	3.476	NA	1000
chr2	91595103	91900000	278.1	4.09	3.476	NA	1000
chr2	91900000	92200000	112.9	1.66	1.854	NA	381
chr2	92200000	92326171	68.5	1.01	1.004	NA	862
chr2	95326171	110109337	68.5	1.01	1.004	NA	862

>chr	begin	end	CNVTypeScore	bestLAFsingle	lowLAFsingle	highLAFsingle
chr2	89830436	90321525	NA	0.38	0.36	0.39
chr2	90371525	90545103	NA	0.3	0.26	0.34
chr2	91595103	91900000	NA	0.38	0.37	0.38
chr2	91900000	92200000	NA	0.41	0.4	0.42
chr2	92200000	92326171	NA	0.22	0.19	0.25
chr2	95326171	110109337	NA	0.49	0.48	0.49

File-Specific Header Description ASM/CNV/cnvSegmentsNondiploidBeta-[ASM-ID].tsv.bz2

Key	Description	Allowed Values
#ASSEMBLY_ID	Name of the assembly.	"<assembly-name>-ASM". For example, "GS000000474-ASM".
#FORMAT_VERSION	Version number of the file format.	Two or more digits separated by periods. For example, "0.6".
#GENERATED_AT	Date and time of the assembly.	Year-Month-Day Time. For example "2010-Sep-08 20:27:52.457773".
#GENERATED_BY	Assembly pipeline component that generated the output.	Alpha-numeric string.
#GENOME_REFERENCE	Human genome build used for assembly.	"NCBI build XX" where X's are digits.
#MEAN_LEVEL_N	Ratio of mean coverage of level to genome-wide mean coverage for "level N", N an integer from 0 to NUMBER_LEVELS-1.	Positive floating points.
#NUMBER_LEVELS	Number of distinct coverage levels	Positive integer.
#SAMPLE	Complete Genomics identifier of the sample from which the library was created.	"GSXXXXX-DNA_YZZ" where<br>▪ X's are digits<br>▪ -DNA_ is literal<br>▪ YZZ is the location of the sample in a 96-well plate with Y as one of "A" through "H" and ZZ is one of "01" through "12"<br>For example "GS12345-DNA_A01".
#SOFTWARE_VERSION	Assembly pipeline build number.	Two or more digits separated by periods
#TYPE	Indicates the type of data contained in the file.	TUMOR-CNV-SEGMENTS: segmentation of the reference genome into regions of distinct coverage level.
#WINDOW_SHIFT	Shift, in bases, between consecutive windows in which smoothed coverage is calculated for copy number estimation.	Positive integer.
#WINDOW_WIDTH	Width, in bases, of windows in which smoothed coverage is calculated for copy number estimation.	Positive integer.

Content Description *ASM/CNV/cnvSegmentsNondiploidBeta-[ASM-ID].tsv.bz2*

	Column Name	Description
1	chr	Chromosome name in text: `chr1`, `chr2`,..., `chr22`, `chrX`, `chrY`. The mitochondrion is represented as `chrM`, though this may be absent from CNV analyses. The pseudoautosomal regions within the sex chromosomes X and Y are reported at their coordinates on chromosome X.
2	begin	Beginning of segment.
3	end	End of segment.
4	avgNormalizedCvg	Baseline-normalized average coverage over the interval from *begin* to *end*. *avgNormalizedCvg* is no-called (`N`) in regions of the genome with very high or very low GC content, as well as in regions with very low average coverage among the baseline samples. See further description in "Detailed Non-Diploid Coverage Level Information."
5	relativeCvg	*avgNormalizedCvg* divided by estimate of diploid median average adjusted coverage. Value is `N` if *avgNormalizedCvg* is `N`.
6	calledLevel	Called coverage level for segment. Values give floating point relative coverage for the assigned level. A value of 'N' indicates that whole genome coverage has been 'no-called'; see "100k normalized coverage variability" in the content description.
7	calledCNVType	`NA`. *CNVType* is not called for non-diploid samples at this time. This field is present to maintain the same format as the diploid sample CNV Segments file and as a placeholder for future developments.
8	levelScore	Phred-like confidence that the segment belongs to the called level, as compared to the alternative levels included in the model.
9	CNVTypeScore	`NA`. *CNVType* is not called for non-diploid samples at this time. This field is present to maintain the same format as the diploid sample CNV Segments file and as a placeholder for future developments.
10	bestLAFsingle	Maximum likelihood estimate of Lesser Allele Fraction (LAF) of the segment based on counts of reads supporting the two alleles at fully called loci with variations. Floating point value between 0 and 0.5.
11	lowLAFsingle	Minimum value within interval that approximates the 99% confidence interval on the Bayesian posterior estimate under a uniform prior. Floating point value between 0 and 0.5.
12	highLAFsingle	Maximum value within interval that approximates the 99% confidence interval on the Bayesian posterior estimate under a uniform prior. Floating point value between 0 and 0.5.

Detailed Non-Diploid Coverage Level Information

ASM/CNV/cnvDetailsNondiploidBeta-[ASM-ID].tsv.bz2

The non-diploid coverage level details file provides information on estimated coverage level every 100kb along the genome, giving average coverage, the coverage level of the segment to which the window is assigned and a confidence score for that assignment.

Example *ASM/CNV/cnvDetailsNondiploidBeta-[ASM-ID].tsv.bz2*

The example shows information typical of the ***cnvDetailsNondiploidBeta-[ASM-ID].tsv.bz2*** file:

- The first record indicates that the region of length `WINDOW_WIDTH` with begin and end positions of `84000000` and `84100000` of chr1 has average normalized coverage of `121.4` times the median value; this region is assigned to coverage level `1.879` with a *levelScore* of `190`, which indicates high confidence in the called level (considered against the alternatives among the modeled levels).
- Records reporting begin positions `84100000` to `84400000` also have called coverage level = `1.879`. The *levelScore* decreases on the last of these rows, reflecting decreased (though still substantial) confidence in the level in the vicinity of the transition to another level.
- The remaining records show the beginning of a region called coverage level `0.966`, with similarly increasing confidence further from the transition reflected in *levelScore*.
- The *levelScore* values are Phred-like scores. A score of 0 means effectively zero confidence, and larger values mean more confidence. They are computed as -10*$\log_{10}$ of the probability of the assigned call being wrong, though due to differences between reality and the model, they may not give quantitatively reliable measures of probabilities. Scores for segments are computed such that they are the average of the scores for the constituent detail positions.
- The LAFsingle columns provide the maximum likelihood lesser allele fraction (LAF) measurement for the 100 kb window as well as the 99% confidence interval around the most likely value. In the example, the upper region has LAF measurements near 0.5, consistent with a normal heterozygous diploid region. The lower regon (beginning at 84500000) has LAF measurements approaching 0, consistent with a region of homozygosity.

The data is broken into sections to show all the columns. The second section repeats the *chr*, *begin*, and *end* columns at the left edge to more easily match the data with the previous section of data; these columns are not repeated in the actual data.

>chr	begin	end	avgNormalizedCvg	gcCorrectedCvg	fractionUnique	relativeCvg	calledLevel	calledCNVType	levelScore
chr1	84000000	84100000	121.4	128.4	1	1.9	1.879	NA	190
chr1	84100000	84200000	123.1	124.8	0.99	1.93	1.879	NA	148
chr1	84200000	84300000	124.4	130.4	0.99	1.95	1.879	NA	112
chr1	84300000	84400000	151.9	150.4	0.99	2.38	1.879	NA	81
chr1	84400000	84500000	119.4	121.8	1	1.87	1.879	NA	81
chr1	84500000	84600000	63.3	61.8	0.99	0.99	0.966	NA	232
chr1	84600000	84700000	61.9	64.6	0.97	0.97	0.966	NA	348
chr1	84700000	84800000	63.1	64.9	1	0.99	0.966	NA	334
chr1	84800000	84900000	62.9	65.5	0.99	0.99	0.966	NA	337

>chr	begin	end	CNVTypeScore	bestLAFsingle	lowLAFsingle	highLAFsingle
chr1	84000000	84100000	NA	0.5	0.47	0.5
chr1	84100000	84200000	NA	0.5	0.45	0.5
chr1	84200000	84300000	NA	0.02	0.02	0.03
chr1	84300000	84400000	NA	0.43	0.41	0.46
chr1	84400000	84500000	NA	0.41	0.39	0.44
chr1	84500000	84600000	NA	0.02	0.02	0.04
chr1	84600000	84700000	NA	0.01	0.01	0.03
chr1	84700000	84800000	NA	0.02	0.02	0.02
chr1	84800000	84900000	NA	0.02	0.02	0.02

File-Specific Header Description ASM/CNV/cnvDetailsNondiploidBeta-[ASM-ID].tsv.bz2

Key	Description	Allowed Values
#ASSEMBLY_ID	Name of the assembly	“<assembly-name>-ASM”. For example, “GS000000474-ASM”.
#FORMAT_VERSION	Version number of the file format	Two or more digits separated by periods. For example, “0.6”.
#GENERATED_AT	Date and time of the assembly	Year-Month-Day Time. For example “2010-Sep-08 20:27:52.457773”.
#GENERATED_BY	Assembly pipeline component that generated the output	Alpha-numeric string.
#GENOME_REFERENCE	Human genome build used for assembly	“NCBI build XX” where X’s are digits.
#MEAN_LEVEL_N	Ratio of mean coverage to genome-wide mean coverage for each level N from 0 to NUMBER_LEVELS-1	Positive floating points.
#NUMBER_LEVELS	Number of distinct coverage levels	Positive integer.
#SAMPLE	Complete Genomics identifier of the sample from which the library was created	“GSXXXXX-DNA_YZZ” where<br>▪ X’s are digits<br>▪ -DNA_ is literal<br>▪ YZZ is the location of the sample in a 96-well plate with Y as one of “A” through “H” and ZZ is one of “01” through “12”<br>For example “GS12345-DNA_A01”.
#SOFTWARE_VERSION	Assembly pipeline build number	Two or more digits separated by periods.
#TYPE	The type of data contained in the file	TUMOR-CNV-DETAILS: estimated coverage level for every 100 kb non-overlapping window along the genome.
#WINDOW_SHIFT	Shift, in bases, between consecutive windows in which smoothed coverage is calculated for copy number estimation.	Positive integer.
#WINDOW_WIDTH	Width, in bases, of windows in which smoothed coverage is calculated for copy number estimation.	Positive integer.

Content Description *ASM/CNV/cnvDetailsNondiploidBeta-[ASM-ID].tsv.bz2*

	Column Name	Description
1	chr	Chromosome name in text: chr1, chr2,..., chr22, chrX, chrY. The mitochondrion is represented as chrM, though this may be absent from CNV analyses. The pseudoautosomal regions within the sex chromosomes X and Y are reported at their coordinates on chromosome X.
2	begin	Beginning of window being described. For most of the genome, chromosome coordinates are even multiples of window length (for example, for 100K windows, window boundaries will end with "x000", where x is an even digit). Exceptions to this are windows at the ends of contigs. Windows will never span bases taken from more than one contig, even if the gap between contigs is small enough to permit this. Bases outside the outermost full default windows for each contig will either be added to the first full window towards the center of the contig or be placed in their own window, depending on whether the number of bases is larger than half the window width or not.
3	end	End of window being described. See also information for *begin* column.
4	avgNormalizedCvg	Baseline-normalized average coverage of a window of width WINDOW_WIDTH. This is the value that is ultimately used to estimate ploidy; *avgNormalizedCvg* is derived from *gcCorrectedCvg* by normalization against other genomes. *avgNormalizedCvg* is no-called (N) in regions of the genome with very high or very low GC content, as well as in regions with very low average coverage among the baseline samples.
5	gcCorrectedCvg	GC-corrected average coverage of a window of width WINDOW_WIDTH. The *gcCorrectedCvg* is no-called (N) in regions of the genome with very high or very low GC content.
6	fractionUnique	Fraction of coverage due to unique mappings.
7	relativeCvg	*avgNormalizedCvg* divided by estimate of diploid median normalized adjusted coverage. Value is N if *avgNormalizedCvg* is N.
8	calledLevel	Called coverage level for segment containing this detail interval. Values give floating point relative coverage for the assigned level. A value of 'N' indicates that whole genome coverage has been 'no-called'; see "100k normalized coverage variability" in the content description.
9	calledCNVType	NA. CNVType is not called for non-diploid samples at this time. This field is present to maintain the same format as the diploid sample CNV Detail file and as a placeholder for future developments.
10	levelScore	Phred-like confidence that the position has the called level, as compared to the alternative levels included in the model.
11	CNVTypeScore	NA. CNVType is not called for non-diploid samples at this time. This field is present to maintain the same format as the diploid sample CNV Detail file and as a placeholder for future developments.
12	bestLAFsingle	Maximum likelihood estimate of Lesser Allele Fraction (LAF) of the window based on counts of reads supporting the two alleles at fully called loci with variations. Floating point value between 0 and 0.5.
13	lowLAFsingle	Minimum value within interval that approximates the 99% confidence interval on the Bayesian posterior estimate under a uniform prior. Floating point value between 0 and 0.5.
14	highLAFsingle	Maximum value within interval that approximates the 99% confidence interval on the Bayesian posterior estimate under a uniform prior. Floating point value between 0 and 0.5.

Depth of Coverage Report

ASM/CNV/depthOfCoverage_100000-[ASM-ID].tsv

The ***depthOfCoverage_100000-[ASM-ID].tsv*** file reports unique and weight-sum sequence coverage, along with GC bias-corrected weight-sum coverage and baseline normalized coverage for every non-overlapping 100 kb window along the genome, facilitating the presentation of whole-genome coverage.

Example ***ASM/CNV/depthOfCoverage_100000-[ASM-ID].tsv***

>chromosome	begin	end	uniqueSequenceCoverage	weightSumSequenceCoverage	gcCorrectedCvg	avgNormalizedCoverage
chr1	10000	100000	10.983	100.154	105.584	88.5
chr1	100000	177417	4.233	74.921	76.313	91.9
chr1	227417	267719	11.443	99.353	95.888	90.3
chr1	317719	400000	0.129	57.173	59.302	82.9
chr1	400000	471368	0.095	69.652	68.811	86.1
chr1	521368	600000	55.614	624.928	597.642	69.5
chr1	600000	700000	3.817	66.198	66.529	82.8
chr1	700000	800000	37.893	79.457	77.735	68.6
chr1	800000	900000	63.27	72.956	89.724	67.6
chr1	900000	1000000	40.54	43.097	70.225	67.1
chr1	1000000	1100000	44.854	47.877	70.077	67.9

- The example data has `#WINDOW_WIDTH` of 100 kb. For most of the genome, chromosome coordinates are even multiples of window length. For example, for 100K windows, window boundaries will end with “x000”, where x is an even digit. Exceptions to this are windows at the ends of contigs. Windows will never span bases taken from more than one contig, even if the gap between contigs is small enough to permit this. Bases outside the outermost full default windows for each contig will either be added to the first full window towards the center of the contig or be placed in their own window, depending on whether the number of bases is larger than half the window width or not.
- The first row of the example data represents coverage data for the first 100 kb window of the contig. As shown in row 2, a consecutive full 100 kb window cannot be constructed because there is not 100 kb left in the contig, hence it ends at position `177417`.
- Note that regularly spaced consecutive 100 kb windows begin on chr1 at position `600000`.

Figure 7 shows GC-bias-corrected coverage for chromosome 1 generated from information contained in the ***depthOfCoverage_100000-[ASM-ID].tsv*** file. The content of Figure 7 does not match the data provided in the example.

Figure 7: Plot of GC Bias-Corrected Coverage across Chromosome 1

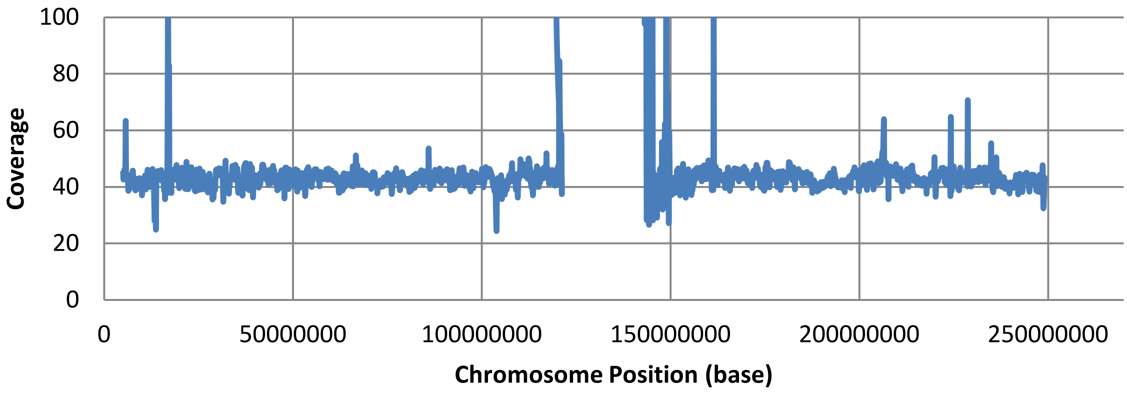

File-Specific Header Description *ASM/CNV/depthOfCoverage_100000-[ASM-ID].tsv*

Key	Description	Allowed Values
#ASSEMBLY_ID	Name of the assembly.	"<assembly-name>-ASM". For example, "GS000000474-ASM".
#SOFTWARE_VERSION	Assembly pipeline build number.	Two or more digits separated by periods.
#GENERATED_BY	Assembly pipeline component that generated the output.	Alpha-numeric string.
#GENERATED_AT	Date and time of the assembly.	Year-Month-Day Time. For example "2010-Sep-08 20:27:52.457773".
#FORMAT_VERSION	Version number of the file format.	Two or more digits separated by periods. For example, "0.6".
#GENOME_REFERENCE	Human genome build used for assembly.	"NCBI build XX" where X's are digits.
#SAMPLE	Complete Genomics identifier of the sample from which the library was created.	"GSXXXXX-DNA_YZZ" where<br>▪ X's are digits<br>▪ -DNA_ is literal<br>▪ YZZ is the location of the sample in a 96-well plate with Y as one of "A" through "H" and ZZ is one of "01" through "12"<br>For example "GS12345-DNA_A01".
#WINDOW_SHIFT	Shift, in bases, between consecutive windows in which coverage is calculated.	Positive integer.
#WINDOW_WIDTH	Width, in bases, of windows in which coverage is calculated.	Positive integer.
#TYPE	Indicates the type of data contained in the file.	DEPTH-OF-COVERAGE: Positive integer.

Content Description ASM/CNV/depthOfCoverage_100000-[ASM-ID].tsv

	Column Name	Description
1	chromosome	Chromosome name in text: `chr1`, `chr2`,…, `chr22`, `chrX`, `chrY`. The mitochondrion is represented as `chrM`. The pseudoautosomal regions within the sex chromosomes X and Y are reported at their coordinates on chromosome X.
2	begin	Beginning of window being described. See description of the window width.
3	end	End of window being described. See description of the window width.
4	uniqueSequenceCoverage	Average per base coverage of the interval of length `WINDOW_WIDTH` centered at the indicated position by unique, fully mapping reads. In a fully mapping read, both arms map with expected order, orientation, and separation, and the weight of this mapping indicates only one high-probability mapping.
5	weightSumSequenceCoverage	Average per base coverage of the interval of length `WINDOW_WIDTH` centered at the indicated position as determined by adding the weight ratio for each full DNB mapping covering this position. In the case of a DNB that is mapped to more than one location, each mapped location receives a fractional contribution to coverage. This weight ratio is a measure of the probability that the mapping is correct for this DNB.
6	gcCorrectedCvg	Average per base coverage of the interval of length `WINDOW_WIDTH` centered at the indicated position as determined by the GC-corrected, weight-sum coverage by full DNB mapping of the spanned positions. The *gcCorrectedCvg* is no-called ('N') in regions of the genome with very high or very low GC content.
7	avgNormalizedCoverage	Average per base coverage of the interval of length `WINDOW_WIDTH` centered at the indicated position as determined by the GC-corrected, weight-sum full DNB mapping covering the spanned positions, normalized relative to a set of standard genomes. *avgNormalizedCoverage* is no-called (‘N’) in regions of the genome with very high or very low GC content, as well as in regions with very low average coverage among the baseline samples.

Structural Variation Files

The SV Directory contains information on detected junctions and provides alignment information for the DNBs supporting each junction cluster. Figure 8 shows the contents of the SV results directory.

Figure 8: SV Directory Contents

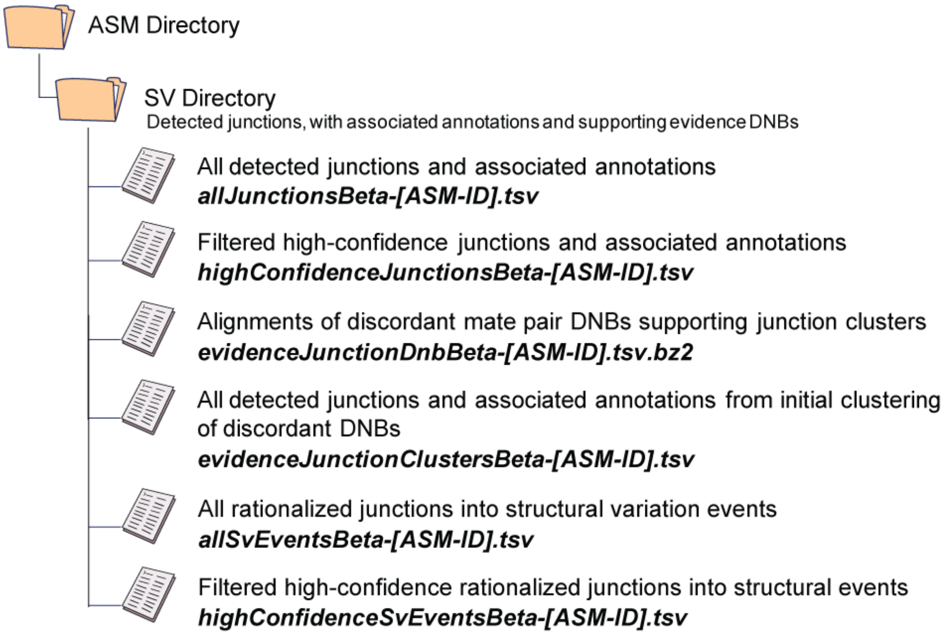

Structural variants change the structure of the genome, and include events such as insertions, deletions, inversions, and translocation. Complete Genomics SV Analysis Pipeline identifies junctions between regions of the genome being sequenced that are not adjacent on the reference genome. Events such as translocations, inversions, and insertions manifest as two or more junctions.

DNB mappings found during the standard assembly process are analyzed to find clusters of DNBs in which each arm maps uniquely to the reference genome, but with an unexpected mate pair length or anomalous orientation. Currently this distance threshold is set to 150 bp greater than the largest mate gap in the data after discarding the most extreme 2.0% of the distribution, or about 700 bp. The mean mate pair length estimated for each sequenced genome, along with 95% confidence interval, is reported in the ***summary.tsv*** file. Each cluster represented by five or more DNBs is reported as a junction with associated annotations, such as coordinates of breakpoints, estimated from this initial clustering in the ***evidenceJunctionClustersBeta-[ASM-ID].tsv*** file. Alignments of DNBs for each cluster are reported in the ***evidenceJunctionDnbBeta-[ASM-ID].tsv.bz2*** file.

After a junction has been identified, DNBs one mate gap away on both sides of the junction are gathered for local de novo assembly of the transition sequence — sequences that are either novel or non-contiguous with the reference genome on either the left or right side of the junction. Currently, the output includes the transition sequence with the highest confidence and not an attempt to define ploidy of the event. Each junction, annotated with a list of repetitive sequence and genes on either side of the junction, is reported with a unique Junction ID in the ***allJunctionsBeta-[ASM-ID].tsv*** file. Junctions reported in the ***allJunctionsBeta-[ASM-ID].tsv*** file are then filtered using a set of criteria to obtain a set of high-confidence junctions. These junctions are reported in the ***highConfidenceJunctionsBeta-[ASM-ID].tsv*** file.

A junction is defined as a region of genomic sequence consisting of three sections: “Left” indicates the position that is closer to the first position of chromosome 1; “Right” indicates the position that is closer to the last position of chromosome Y; and “Transition”. Left is synonymous for “5' direction” and Right is synonymous for “3' direction”. The left and right positions of the junction are defined as the “begin” of the half-open zero-length reference interval (space coordinates), marking the boundary between the

left/right section and the transition section. This boundary can be interpreted as the breakpoints of the identified junction, whose position can be further refined by de novo assembly.

Figure 9: Example of a Typical Junction

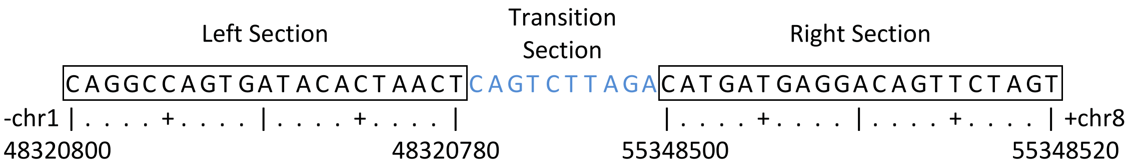

1. A “left” section of n_L bases that maps, exactly or approximately, to a section of the reference genome (either strand).
2. A “transition” section of n_T bases of genomic sequence (n_T can be zero). The transition sequence is represented in blue text in Figure 9.
3. A “right” section of n_R bases that maps, exactly or approximately, to a section of the reference genome (either strand).

A junction can be represented a second way: the junction shown above can be obtained by swapping the positions and lengths of the left and right section, changing their strands, and reverse complementing the sequence of the transition region. Thus, we define a canonical representation of junctions in which the left side is at an earlier position on the reference than the right side.

It is important to understand that a junction does not necessarily imply a physical connection between the genomic sequences to the left and right of the junction breakpoint. A junction can also be explained by sequence similarity of one DNB arm to another region in the genome. Several annotations provided in the junction files help discern between these two possibilities. For example, success in assembling a sequence across the junction breakpoint gives stronger support to there being a physical connection between the two sections of the junction. The number of discordant mate pairs supporting the junction reported in the *DiscordantMatePairAlignments* field correlates with confidence that junction is a true event. Finally, junctions flanked by repeating genomic elements are of lower confidence, as likelihood of false mappings is increased in repeats regions.

Detected Junctions and Associated Annotations

ASM/SV/allJunctionsBeta-[ASM-ID].tsv

This junction file gives information for individual junctions that were detected in the sequenced genome. Each junction is given a unique identifier and is ordered by the chromosomal position of the left section of the junction. Associated data and annotations for each junction are also included.

If the transition sequence is missing, *TransitionLength* is zero and *TransitionSequence* is empty. This expresses the fact that transition from the left to the right side takes place without any intervening bases of new sequence. If local *de novo* assembly is unsuccessful, the transition sequence is unknown, *TransitionSequence* field is empty, and the *TransitionLength* is calculated from the initial clustering of DNBs during junction detection.

Example — *ASM/SV/allJunctionsBeta-[ASM-ID].tsv*

The data in this example appears in three sections (15 columns, 6 columns, and 4 columns) to accommodate the width of the file content. The second and third sections of data repeat the *Id* column at the left edge to more easily match the data with the previous section of data; the *Id* column is not repeated in the actual data.

- Junction with Id 2972 is detected on Chr1, with *LeftPosition* at 53594099 and *RightPosition* at 53595603. The distance between the left and right putative breakpoints, as measured on the reference genome, is 1504 bp (as indicated in *Distance* column), while the *TransitionLength* of the junction is 175 bp in the sequenced genome. This observation is consistent with a deletion of 1329 bp (1504bp – 175bp) in the sequenced genome. The *LeftSection* and *LeftGene* fields indicate that the left section of the junction overlaps with an AluJr4 and transcript NM_006671 of the SLC1A7 gene, which resides on the negative strand. The *RightGene* field indicates that the right section of the junction also overlaps with transcript NM_006671 of the SLC1A7 gene. Viewing this junction region in the UCSC genome browser reveals that the putative deletion occurs in the intronic region of the SLC1A7 gene.
- Junction with Id 2460 has a left section that maps to Chr1, at *LeftPosition* 121484195 and right section that maps to chr8, at *RightPosition* 43786732. This observation is consistent with an interchromosomal event in the sequenced genome. However, information provided in other fields within this file indicates low confidence in this junction call:
    - The number of discordant mate pair mappings supporting the junction is at the threshold of detection. Note this example shows data from pipeline 2.4 where the minimum number of DNBs required to support a junction was three. This threshold was increased to five DNBs in pipeline 2.5.
    - An attempt to locally assemble sequence across the left and right sections of the junction failed.
    - *KnownUnderrepresentedRepeat* flag is set, indicating that the ALR/Alpha repeat sequence that is not properly represented in the reference, overlaps at least one side of the junction.
    - Both left and right sections of the junction are very short, indicating that the interchromosomal event, even if real, affects only a very short patch of sequence.
- Junction with Id 2888 detected on chr1, with *LeftPosition* at 234318646 and *RightPosition* at 234319749. The distance between the left and right putative breakpoints, as measured on the reference genome, is 1103 bp (as indicated in *Distance* column), while the *TransitionLength* of the junction is 0 bp in the sequenced genome. This observation is consistent with a deletion of 1103 bp (1103bp – 0bp) in the sequenced genome. As indicated in *JunctionSequenceResolved* field, assembly of sequence across left and right sections of the junction was successful, with no transition sequence detected. The putative deletion overlaps with NM_173508 transcript of the SLC35F3 gene (which resides on the positive strand) and a known variation in dbSNP (rs67814471(chr1:234318644-

234319747) reported in the *xRef* field). Viewing this junction region in the UCSC genome browser reveals that the putative deletion occurs in the intronic region of the SLC35F3 gene.

>Id	LeftChr	LeftPosition	LeftStrand	LeftLength	RightChr	RightPosition	RightStrand	RightLength	StrandConsistent	Interchromosomal	Distance	DiscordantMatePairAlignments	JunctionSequenceResolved	TransitionSequence
2972	chr1	53594099	+	343	chr1	53595603	+	240	Y	N	1504	13	Y	AATAACCTCGTGAAGGAGGTATTCTTCCCACTTTATAGATAAGGACACTGAGGCTCAGATGCTAAAAG…
2460	chr1	121484195	+	36	chr8	43786732	+	35	Y	Y		3	N	
2888	chr1	234318646	+	424	chr1	234319749	+	458	Y	N	1103	68	Y	

>Id	TransitionLength	LeftRepeatClassification	RightRepeatClassification	LeftGenes	RightGenes	XRef
2972	175	AluJr4:SINE:Alu		NM_006671:-	NM_006671:-	
2460	534	ALR/Alpha:Satellite: centr;Self chain; Tandem period 171; Tandem period 340	ALR/Alpha:Satellite: centr;Tandem period 171; Tandem period 342; Tandem period 513			
2888	0	L1MC5:LINE:L1		NM_173508:+	NM_173508:+	rs67814471 (chr1:234318644-234319747)

>Id	DeletedTransposableElement	KnownUnderrepresentedRepeat	FrequencyInBaselineGenomeSet	AssembledSequence	EventId	Type	RelatedJunctions
2972			0.2	cgggaggatcgattgagccctggagttgaaAGTTACAGTGAGCTG TAATAACCTCGTGAAGGAGGTATTCTTCCCACTTTATAGATAAGG ACACTGAGGCTCAGATGCTAAAAGGCTTGTTTACATTTGCACATC TAGAGGGTGACTCCAAAGCCCTGTTCCTGCCCTGTAGCCTTTGCA GATTTCAACCACCCCCGCCCATGCTTCCTGCTCCCCCGCCACATG CCGCTGGCCcgaccccttgacagtggctttcttgttcag	3013	Deletion	
2460		Y	0.95		1440	Interchromosomal	
2888			0.2	tcctgtgaactctgaccatatctctagtccATTTTCTATACAAAA GGagcactcagttcaaattcacattggttact	3476	Deletion	

File-Specific Header Description *ASM/SV/allJunctionsBeta-[ASM-ID].tsv*

Key	Description	Allowed Values
#ASSEMBLY_ID	Name of the assembly.	"<assembly-name>-ASM". For example, "GS000000474-ASM".
#SOFTWARE_VERSION	Assembly pipeline build number.	Two or more digits separated by periods.
#GENERATED_BY	Assembly pipeline component that generated the output.	Alpha-numeric string.
#GENERATED_AT	Date and time of the assembly.	Year-Month-Day Time. For example "2010-Sep-08 20:27:52.457773".
#FORMAT_VERSION	Version number of the file format.	Two or more digits separated by periods. For example, "0.6".
#GENOME_REFERENCE	Human genome build used for assembly.	"NCBI build XX" where X's are digits.
#SAMPLE	Complete Genomics identifier of the sample from which the library was created.	"GSXXXXX-DNA_YZZ" where<br>▪ X's are digits<br>▪ -DNA_ is literal<br>▪ YZZ is the location of the sample in a 96-well plate with Y as one of "A" through "H" and ZZ is one of "01" through "12"<br>For example "GS12345-DNA_A01".
#TYPE		JUNCTIONS
#DBSNP_BUILD	dbSNP version used for annotation.	"dbSNP build XXX" where X's are digits. For example, "dbSNP build 130".
#GENE_ANNOTATIONS	NCBI annotation build.	"NCBI build XX.X" where X's are digits.

Content Description — *ASM/SV/allJunctionsBeta-[ASM-ID].tsv*

	Column Name	Description
1	Id	Identifier for the junction. This consists of positive integers. Junction Ids are consistent across all junction files for a given assembly.
2	LeftChr	Left chromosome name in text: `chr1`, `chr2`,..., `chr22`, `chrX`, `chrY`. The mitochondrion is represented as `chrM`. The pseudoautosomal regions within the sex chromosomes X and Y are reported at their coordinates on chromosome X.
3	LeftPosition	Zero-based left position of the junction, as illustrated in Figure 9.
4	LeftStrand	Left strand ("+" or "-").
5	LeftLength	The distance between the first position of the left-most mate read and the last position of the right-most mate read in the cluster, on the left side of the junction, n_L.
6	RightChr	Right chromosome name in text: `chr1`, `chr2`,..., `chr22`, `chrX`, `chrY`. The mitochondrion is represented as `chrM`, though this may be absent from SV analyses. The pseudoautosomal regions within the sex chromosomes X and Y are reported at their coordinates on chromosome X.
7	RightPosition	Zero-based right position of the junction, as illustrated in Figure 9.
8	RightStrand	Right strand ("+" or "-").
9	RightLength	The distance between the first position of the left-most mate read and the last position of the right-most mate read in the cluster, on the right side of the junction, n_L.
10	StrandConsistent	Indicates whether left section and right section of junction are on the same (+,+) or opposite strand (+,-). Possible values are Y and N.
11	Interchromosomal	Indicates whether left section and right section of junction map to the same or different chromosomes. Possible values are Y and N.
12	Distance	The distance between *LeftPosition* and *RightPosition*, as measured on the reference genome.
13	DiscordantMatePairAlignments	A number expressing the amount of DNB support available for this junction.
14	JunctionSequenceResolved	Indicates whether local de novo assembly successfully assembled sequences from gathered DNBs to transition from left section to right section of junction. Possible values are Y and N.
15	TransitionSequence	The base sequence of the transition section. This can be blank if the transition section is unknown or missing.
16	TransitionLength	The length of the transition sequence, n_T. It can be blank if the transition section is unknown, or zero if the transition section is known to be missing.
17	LeftRepeatClassification	Repetitive genomic elements, such as segmental duplication, satellite, or self chain, overlapping left section of junction.
18	RightRepeatClassification	Repetitive genomic elements, such as segmental duplication, satellite, or self chain, overlapping right section of junction.
19	LeftGenes	Gene(s) overlapping left section of junction. For each gene, the transcript name and the strand that contains the gene are specified. For example, "NM_173508:+".
20	RightGenes	Gene(s) overlapping right section of junction. For each gene, transcript name and the strand that contains the gene are specified. For example, "NM_173508:+".
21	XRef	For junctions consistent with a deletion event, variations in dbSNP that are similar to the putatively deleted genomic region between *LeftPosition* and *RightPosition* of junction.

	Column Name	Description
22	DeletedTransposableElement	For junctions consistent with a deletion event, transposable elements of AluY and L1 subclasses (with divergence at or below 2%) that overlap genomic region between *LeftPosition* and *RightPosition* of junction. The total divergence of the repeat element from the consensus sequence is reported after the repeat name, for example “L1HS 0.8%”.
23	KnownUnderrepresentedRepeat	Repetitive genomic elements known to be underrepresented in the human reference genome overlapping either of the junction sides. These genomic elements include ALR/Alpha, GAATGn, HSATII, LSU_rRNA_Hsa, and RSU_rRNA_Hsa.
24	FrequencyInBaseline	Frequency that junction is detected in set of baseline genomes. The files containing junctions detected across the baseline genome set and their frequencies are available for download. See SV Baseline Genome Dataset.
25	AssembledSequence	Sequence from DNBs used to seed local de novo assembly (in lowercase) and sequence locally assembled from the gathered DNBs.
26	EventID	Positive integer identifier for the event that includes this junction. Event IDs are consistent across all junction files for a given assembly.
27	Type	Structural rearrangement composed of one or more junctions. Possible values include:<br>▪ `artifact`<br>▪ `complex`<br>▪ `deletion`<br>▪ `tandem-duplication`<br>▪ `probable-inversion`<br>▪ `inversion`<br>▪ `distal-duplication`<br>▪ `distal-duplication-by-mobile-element`<br>▪ `interchromosomal`<br>Note that this category always describes the event type for an individual sample.
28	RelatedJunctions	Identifier of other junctions that make up the event indicated in *EventID* field. This semicolon-separated list does not include the current junction.

High-confidence Junctions and Associated Annotations

ASM/SV/highConfidenceJunctionsBeta-[ASM-ID].tsv

The ***highConfidenceJunctionsBeta-[ASM-ID].tsv*** file contains a filtered subset of the junctions reported in the ***allJunctionsBeta-[ASM-ID].tsv*** file. This subset represents our high confidence calls—junctions that likely resulted from a true physical connection between the left and right sections of the junctions. To obtain the junctions reported in this file, we applied the following filter criteria to the junctions in the ***allJunctionsBeta-[ASM-ID].tsv*** file:

- Include the junction if *DiscordantMatePairAlignments* ≥ 10 (10 or more discordant mate pairs in cluster) AND
- Include the junction if *JunctionSequenceResolve* = Y (local de novo assembly is successful) AND
- Exclude interchromosomal junction if present in any genomes in baseline samples (*FrequencyInBaseline* > 0) AND
- Exclude the junction if overlap with known underrepresented repeats (*KnownUnderrepresentedRepeat* = Y): ALR/Alpha, GAATGn, HSATII, LSU_rRNA_Hsa, and RSU_rRNA_Hsa AND
- Exclude the junction if the length of either of the side sections is less than 70 base pairs.

The header and file format description for the High-confidence Junctions and Associated Annotations file are the same as the “Detected Junctions and Associated Annotations” files.

© Complete Genomics, Inc.

Alignments of DNBs in Junction Cluster

ASM/SV/evidenceJunctionDnbBeta-[ASM-ID].tsv.bz2

Junctions are detected by finding clusters of DNBs in which mate pairs map uniquely to the reference genome, but with an unexpected mate pair length or anomalous orientation. Alignments of the individual DNB mate pairs supporting each cluster are reported in the ***evidenceJunctionDnbBeta-[ASM-ID].tsv.bz2*** file.

Example *ASM/SV/evidenceJunctionDnbBeta-[ASM-ID].tsv.bz2*

The example shows an Evidence Junction DNBs file. The first section shows the first 11 columns; the remaining 9 columns appear in the lower section, with the sequence and score data truncated. The second section of data repeats the *JunctionId* column at the left edge to more easily match the data with the previous section of data; the *JunctionId* column is not repeated in the actual data.

>JunctionId	Slide	Lane	FileNumInLane	DnbOffsetInLaneFile	LeftDnbSide	LeftStrand	LeftChromosome	LeftOffsetInReference	LeftAlignment	LeftMappingQuality
511	GS14634-FS3	L05	6	22998080	R	+	chr1	212657217	10M5N10M0N10M2B5M	~
511	GS14635-FS3	L02	3	3959434	R	+	chr1	212657201	10M5N10M0N10M2B5M	~
511	GS14635-FS3	L06	1	6983686	R	+	chr1	212657195	10M6N10M0N10M2B5M	p
512	GS14634-FS3	L07	1	3616508	L	-	chr1	211707545	10M6N10M0N10M2B5M	~
-512	GS14635-FS3	L08	7	13280153	R	+	chr1	211707551	10M5N10M0N10M2B5M	I
512	GS14640-FS3	L01	1	21038723	L	-	chr1	211707653	10M7N10M0N10M1B5M	r
512	GS14640-FS3	L07	2	15167398	R	+	chr1	211707541	10M6N10M0N10M2B5M	+

>JunctionId	RightDnbSide	RightStrand	RightChromosome	RightOffsetInReference	RightAlignment	RightMappingQuality	EstimatedMateDistance	Sequence	Scores
511	L	-	chr1	212658103	10M6N10M0N10M2B5M	u	330	TGGTGAGTCAC…	6'%$&58749578…
511	L	-	chr1	212658095	10M6N10M0N10M2B5M	q	306	CTTTATAGTAGG…	699/)$(4%27874…
511	L	-	chr1	212658057	10M5N10M0N10M2B5M	k	262	GAATATACAATA…	899::577427778…
512	R	+	chr1	211708880	10M6N10M0N10M2B5M	j	354	ATTGGGGCACC…	698:61/8086.775…
-512	L	-	chr1	211708820	10M5N10M0N10M2B5M	|	300	CTCTGTGGCCAT…	5+0$(23336-)&64…
512	R	+	chr1	211708795	10M5N10M0N10M2B5M	7	377	AAAAAAGCTCAG…	778/+97:88.8888…
512	L	-	chr1	211708803	10M6N10M0N10M2B5M	t	273	GAAAGAGCAAA…	*3642,%8778767…

File-Specific Header Description ASM/SV/evidenceJunctionDnbBeta-[ASM-ID].tsv.bz2

Key	Description	Allowed Values
#ASSEMBLY_ID	Name of the assembly	"<assembly-name>-ASM". For example, "GS000000474-ASM".
#FORMAT_VERSION	Version number of the file format	Two or more digits separated by periods. For example, "0.6".
#GENERATED_AT	Date and time of the assembly	Year-Month-Day Time. For example "2010-Sep-08 20:27:52.457773".
#GENERATED_BY	Assembly pipeline component that generated the output.	Alpha-numeric string.
#SAMPLE	Complete Genomics identifier of the sample from which the library was created	"GSXXXXX-DNA_YZZ" where<br>▪ X's are digits<br>▪ -DNA_ is literal<br>▪ YZZ is the location of the sample in a 96-well plate with Y as one of "A" through "H" and ZZ is one of "01" through "12"<br>For example "GS12345-DNA_A01".
#SOFTWARE_VERSION	Assembly pipeline build number	Two or more digits separated by periods.
#TYPE	Indicates the type of data contained in the file.	JUNCTION-DNBS: DNB alignments supporting the detected junction in a genomic interval.

Content Description ASM/SV/evidenceJunctionDnbBeta-[ASM-ID].tsv.bz2

	Column Name	Description
1	JunctionId	Identifier for junction that this DNB alignment supports. Junction Ids are consistent across all junction files for a given assembly.
2	Slide	Identifier for the slide from which data for this DNB was obtained.
3	Lane	Identifier for the lane within the slide from which data for this DNB was obtained.
4	FileNumInLane	The file number of the reads file describing this DNB. (For example, *X* in ***reads_[SLIDE-LANE]_00X.tsv.bz2***.)
5	DnbOffsetInLaneFile	Record within data for the slide lane in ***reads_[SLIDE-LANE]_00X.tsv.bz2*** that corresponds to this DNB.
6	LeftDnbSide	Identifies the side of the DNB that was associated with the "left" (that is, earlier in the reference; on lower-numbered chromosome or with smaller offset within the same chromosome) side of the cluster.<br>L if the left side of the DNB belongs to the left side of the cluster<br>R if the right side of the DNB belongs to the left side of the cluster<br>For the simple case of junctions that connect "+" strand sequence to "+" strand sequence, the left side of DNB belongs to the left side of the cluster if the DNB was produced from the "+" strand of the genomic DNA.
7	LeftStrand	The strand of the half-DNB, "+" or "-", expressed relative to the reference genome.
8	LeftChromosome	Left chromosome name in text: chr1, chr2,..., chr22, chrX, chrY. The mitochondrion is represented as chrM, though this may be absent from SV analyses. The pseudoautosomal regions within the sex chromosomes X and Y are reported at their coordinates on chromosome X.
9	LeftOffsetInReference	The chromosomal position on the reference genome at which the half-DNB starts (as seen on the "+" strand).

	Column Name	Description
10	LeftAlignment	The alignment of the half-DNB to the left section of junction, provided in an extended CIGAR format (see "Alignment CIGAR Format").
11	LeftMappingQuality	A Phred-like encoding of the probability that this half-DNB mapping is incorrect, encoded as a single character with ASCII-33. The Phred score is obtained by subtracting 33 from the ASCII code of the character.
12	RightDnbSide	Identifies the side of the DNB that was associated with the right side of the cluster.
13	RightStrand	The strand of the half-DNB, "+" or "-", expressed relative to the reference genome.
14	RightChromosome	Left chromosome name in text: `chr1`, `chr2`,..., `chr22`, `chrX`, `chrY`. The mitochondrion is represented as `chrM`, though this may be absent from SV analyses. The pseudoautosomal regions within the sex chromosomes X and Y are reported at their coordinates on chromosome X.
15	RightOffsetInReference	The chromosomal position on the reference genome at which the half-DNB starts (as seen on the "+" strand).
16	RightAlignment	The alignment of the half-DNB to the right section of junction, provided in an extended CIGAR format (see "Alignment CIGAR Format").
17	RightMappingQuality	A Phred-like encoding of the probability that this half-DNB mapping is incorrect, encoded as a single character with ASCII-33. The mapping quality is related to the existence of alternate mappings; the Phred score is obtained by subtracting 33 from the ASCII code of the character.
18	EstimatedMateDistance	Estimate of the distance between the left and right arm of the DNB in the assayed genome, taking the junction into account.
19	Sequence	Sequence of the DNB arm bases in the DNB order (same as in the ***reads_[SLIDE-LANE]_00X.tsv.bz2*** file).
20	Scores	Phred-like error scores for DNB bases in the DNB order, not separated (same as in the ***reads_[SLIDE-LANE]_00X.tsv.bz2*** file).

Evidence Junctions and Annotations

ASM/SV/evidenceJunctionClustersBeta-[ASM-ID].tsv

The ***evidenceJunctionClustersBeta-[ASM-ID].tsv*** file contains the same junctions reported in the ***allJunctionsBeta-[ASM-ID].tsv*** file. However, junction annotations such as the putative junction breakpoints (*LeftPosition* and *RightPosition*), junction section lengths (*LeftLength* and *RightLength*), distance between breakpoints (*Distance*), and transition length are estimated from the initial clustering of DNBs during junction detection process (i.e., before these values are further refined by local de novo assembly).

Junction information provided in this file is thus consistent with the mappings and alignments of DNBs provided in the ***evidenceJunctionDnbBeta-[ASM-ID].tsv.bz2*** file. Junction annotations that are provided in other junction files, such as overlapping genes or known underrepresented repeats, are not reported in the ***evidenceJunctionClustersBeta-[ASM-ID].tsv*** file. However, the annotation fields are kept in the file to maintain the same structure as other junctions files, ***allJunctionsBeta-[ASM-ID].tsv*** and ***highConfidenceJunctionsBeta-[ASM-ID].tsv***.

File-Specific Header Description ASM/SV/evidenceJunctionClustersBeta-[ASM-ID].tsv

Key	Description	Allowed Values
#ASSEMBLY_ID	Name of the assembly	"<assembly-name>-ASM". For example, "GS000000474-ASM".
#SOFTWARE_VERSION	Assembly pipeline build number	Two or more digits separated by periods.
#GENERATED_BY	Assembly pipeline component that generated the output.	Alpha-numeric string.
#GENERATED_AT	Date and time of the assembly	Year-Month-Day Time. For example "2010-Sep-08 20:27:52.457773".
#FORMAT_VERSION	Version number of the file format	Two or more digits separated by periods. For example, "0.6".
#GENOME_REFERENCE	Human genome build used for assembly	"NCBI build XX" where X's are digits.
#SAMPLE	Complete Genomics identifier of the sample from which the library was created	"GSXXXXX-DNA_YZZ" where<br>▪ X's are digits<br>▪ -DNA_ is literal<br>▪ YZZ is the location of the sample in a 96-well plate with Y as one of "A" through "H" and ZZ is one of "01" through "12"<br>For example "GS12345-DNA_A01".
#TYPE		JUNCTIONS
#DBSNP_BUILD	dbSNP version used for annotation	"dbSNP build XXX" where X's are digits. For example, "dbSNP build 130".

Content Description *ASM/SV/evidenceJunctionClustersBeta-[ASM-ID].tsv*

	Column Name	Description
1	Id	Identifier for the junction. This consists of positive integers. Junction Ids are consistent across all junction files for a given assembly.
2	LeftChr	Left chromosome name in text: `chr1`, `chr2`,…, `chr22`, `chrX`, `chrY`. The mitochondrion is represented as `chrM`, though this may be absent from SV analyses. The pseudoautosomal regions within the sex chromosomes X and Y are reported at their coordinates on chromosome X.
3	LeftPosition	Zero-based left position of the junction, as defined in the previous section.
4	LeftStrand	Left strand ("+" or "-").
5	LeftLength	The distance between the first position of the left-most mate read and the last position of the right-most mate read in the cluster, on the left side of the junction, n_L.
6	RightChr	Right chromosome name in text: `chr1`, `chr2`,…, `chr22`, `chrX`, `chrY`. The mitochondrion is represented as `chrM`, though this may be absent from SV analyses. The pseudoautosomal regions within the sex chromosomes X and Y are reported at their coordinates on chromosome X.
7	RightPosition	Zero based right position of the junction, as defined in the previous section.
8	RightStrand	Right strand ("+" or "-").
9	RightLength	The distance between the first position of the left-most mate read and the last position of the right-most mate read in the cluster, on the right side of the junction, n_L.
10	StrandConsistent	Indicates whether left section and right section of junction are on the same (+,+) or opposite strand (+,-). Possible values are `Y` and `N`.
11	Interchromosomal	Indicates whether left section and right section of junction map to the same or different chromosomes. Possible values are `Y` and `N`.
12	Distance	The distance between *LeftPosition* and *RightPosition*, as measured on the reference genome.
13	DiscordantMatePairAlignments	A number expressing the amount of DNB support available for this junction.
14	JunctionSequenceResolved	"`NA`". *JunctionSequenceResolved* is not called since information reported in this file is generated before attempting local de novo assembly. This field is present to maintain the same structure as other junction files.
15	TransitionSequence	"`NA`". *TransitionSequence* is not called since information reported in this file is generated before attempting local de novo assembly. This field is present to maintain the same structure as other junction files.
16	TransitionLength	The length of the transition sequence, n_T. It can be blank if the transition section is unknown or zero if the transition section is known to be missing.
17	LeftRepeatClassification	'`NA`'. Repeat and gene annotations are provided in other junctions files, ***allJunctionsBeta-[ASM-ID].tsv*** and ***highConfidenceJunctionsBeta-[ASM-ID].tsv***. This field is present to maintain the same structure as other junction files.
18	RightRepeatClassification	'`NA`'. Repeat and gene annotations are provided in other junctions files, ***allJunctionsBeta-[ASM-ID].tsv*** and ***highConfidenceJunctionsBeta-[ASM-ID].tsv***. This field is present to maintain the same structure as other junction files.
19	LeftGenes	'`NA`'. Repeat and gene annotations are provided in other junctions files, ***allJunctionsBeta-[ASM-ID].tsv*** and ***highConfidenceJunctionsBeta-[ASM-ID].tsv***. This field is present to maintain the same structure as other junction files.

	Column Name	Description
20	RightGenes	'NA'. Repeat and gene annotations are provided in other junctions files, ***allJunctionsBeta-[ASM-ID].tsv*** and ***highConfidenceJunctionsBeta-[ASM-ID].tsv***. This field is present to maintain the same structure as other junction files.
21	XRef	Variation in dbSNP that overlap genomic region between *LeftPosition* and *RightPosition* of junction. Annotation is also provided if junction seems to indicate a deletion not reported in dbSNP (for example, "novel 1223 bp (chr1:6261535-6262758)").
22	DeletedTransposableElement	Transposable elements such as Alu or LINEs, that overlap genomic region between *LeftPosition* and *RightPosition* of the junction.
23	KnownUnderrepresentedRepeat	NA. Repeat and gene annotations are provided in other junctions files, ***allJunctionsBeta-[ASM-ID].tsv*** and ***highConfidenceJunctionsBeta-[ASM-ID].tsv***. This field is present to maintain the same structure as other junction files.
24	FrequencyInBaseline	Frequency that junction is detected in set of baseline genomes. The files containing junctions detected across the baseline genome set and their frequencies are available for download. See "SV Baseline Genome Dataset".
25	AssembledSequence	NA. *AssembledSequence* is not called because information reported in this file is generated before attempting local *de novo* assembly. This field is present to maintain the same structure as other junction files.

Structural Rearrangement Events

ASM/SV/allSvEventsBeta-[ASM-ID].tsv and ASM/SV/highConfidenceSvEventsBeta-[ASM-ID].tsv

Junctions are defined as regions of the genome where sequences are not adjacent or in the same orientation as present in the reference genome. Structural rearrangement events include deletions, inversions, and translocations, and are represented by one or more junctions. Complete Genomics SV pipeline uses the CGA Tools junctions2events utility to rationalize sets of junctions into event types and annotates each event with both discordant mate pair support and biological information. Annotations include predicted gene impact and putative gene fusions. Events reported in the ***allSvEventsBeta-[ASM-ID].tsv*** file are identified by rationalizing all junctions (found in the ***allJunctionsBeta-[ASM-ID].tsv*** file) detected in a sample. Events reported in the ***highConfidenceSvEventsBeta-[ASM-ID].tsv*** file are identified by rationalizing high-confidence junctions (found in the ***highConfidenceJunctionsBeta-[ASM-ID].tsv*** file) within the context of all detected junctions (in the ***allJunctionsBeta-[ASM-ID].tsv*** file). In other words, rationalization is attempted for each junction in the ***highConfidenceJunctionsBeta-[ASM-ID].tsv*** file, but potential events can be constructed using junctions in the ***allJunctionsBeta-[ASM-ID].tsv*** file as potential "partners".

For detailed description of how structural variation event types are deduced from junction data, refer to the junctions2events in *CGA Tools User Guide*. Briefly, this process involves generating an undirected graph of related junctions and then stepping through the following heuristic process:

1. Junctions within 700 bp on at least one side are considered to be connected.
2. Connected components with more than two junctions are assigned as "complex" events.
3. For every other junction, attempts are made to find a related junction in such a way that, together, they may be interpreted as a distance duplication of contiguous sequence. This is done generally by scanning up to 10000000 bp in the direction away from the break indicated by the junction side.
4. Junctions are considered related when their sides can be paired to bound a contiguous piece of sequence from the inside, while their remaining sides bound a small piece of sequence from the outside.
5. Pairs of related junctions are assigned as "inversion" events when the junctions change strand, and the sequence chunk bounded from the inside overlaps to a large degree with the sequence chunk bounded from the outside. For cases with no significant overlap, the event is assigned as "distal duplication".
6. Junction pairs that are connected, but not related in the sense described above, are assigned as "complex" events.
7. For isolated junctions, attempts are made to find a mobile element within 2000 bp that may have caused the junction by copying the adjacent sequence.
8. Remaining isolated junctions that connect sequence on different chromosomes are not classified any further and are assigned as "interchromosomal" events.
9. Finally, the isolated junctions that have both sides on the same chromosome are interpreted based on the strands of the junction sides: Junctions with +/+ sides are assigned as "deletions", -/- as "tandem duplications", and strand-inconsistent junctions as "probable inversions".

In addition to classifying the events by the type, Complete Genomics also annotates each event with biological information:

1. Every event is annotated with the list of all potentially disrupted genes; these are the genes that overlap at least one of the junction side positions for any of the junctions that were grouped into the event.

2. Events that may indicate a copy number change of a stretch of sequence (e.g., “deletion”, “tandem-duplication”, and “distal-duplication” events), all the genes that are completely contained in the affected sequence are included.
3. Possible fusion gene events are identified as follows:
   - When a junction appears to connect two different genes (for example, A and B) in a strand-consistent manner, it is considered a possible gene fusion (described in the file as “A/B”).
   - When a junction connects the region upstream of gene C to an intact gene D in a strand-consistent manner, it is annotated using “TSS-UPSTREAM[C]/D” notation; the size of the upstream region is defined as 7500 bp.

Example ASM/SV/allSvEventsBeta-[ASM-ID].tsv and ASM/SV/highConfidenceSvEventsBeta-[ASM-ID].tsv

This example shows an excerpt from an ***allSvEventsBeta-[ASM-ID].tsv*** file, including the header and a few rows that illustrate a selection of the data you can expect in this file. The second and following sections of data repeat the *EventId* column at the left edge to more easily match the data with the previous section of data; the *EventId* column is not repeated in the actual data.

The ***highConfidenceSvEventsBeta-[ASM-ID].tsv*** file has the identical format and file content would only differ in the events that it reports.

```
#GENERATED_BY        cgatools
#GENERATED_AT        2011-Aug-27
                     20:09:44.666333
#SOFTWARE_VERSION    2.0.0.5
#FORMAT_VERSION      2
#TYPE                SV-EVENTS
```

>EventId	Type	RelatedJunctionIds	MatePairCounts	FrequenciesInBaselineGenomeSet	OriginRegionChr	OriginRegionBegin	OriginRegionEnd	OriginRegionLength	OriginRegionStrand
2	probable-inversion	6	4	0.56	chrM	3520	3752	232	-
3	distal-duplication	7;1412	16;5	0.54;0.52	chrX	131394197	131394262	65	-
7	complex	11;542	7;5	0.48;0.48					
12	artifact	16	3	0					
29	inversion	34;3650	161;83	1.00;1.00	chr21	27374152	27374699	547	-
71	interchromosomal	77	4	0.1	chr14	19494133	19494133	0	-
423	deletion	522	78	0	chrX	154205937	154226596	20659	+
713	tandem-duplication	835	18	0	chr19	10537664	10649198	111534	+
991	tandem-duplication	1193	15	0	chr8	6493316	6599670	106354	+
2773	distal-duplication-by-mobile-element	3275	29	0.54	chr4	80894087	80894475	388	+
3681	probable-inversion	4433	8	0.46	chr1	145833009	147394618	1561609	-

>EventId	DestinationRegionChr	DestinationRegionBegin	DestinationRegionEnd	DestinationRegionLength	DestinationRegionStrand	DisruptedGenes	ContainedGenes	GeneFusions
2								
3	chrX	131393591	131393591	0	+			
7								
12								
29	chr21	27374158	27374705	547	+	APP		
71	chr19	19632141	19632141	0	+	NDUFA13		
423						F8		
713						PDE4A	KEAP1; PDE4A; S1PR5	TSS-UPSTREAM[ATG4D]/PDE4A
991						AGPAT5; MCPH1		AGPAT5/MCPH1
2773	chr5	21207713	21207713	0	+	ANTXR2		
3681								TSS-UPSTREAM[GPR89B]/GPR89A

>EventId	RelatedMobileElement	MobileElementChr	MobileElementBegin	MobileElementEnd	MobileElementStrand
2					
3					
7					
12					
29					
71					
423					
713					
991					
2773	L1:L1HS:0.5	chr4	80888061	80894087	+
3681					

File-Specific Header Description ASM/SV/allSvEventsBeta-[ASM-ID].tsv and ASM/SV/highConfidenceSvEventsBeta-[ASM-ID].tsv

Key	Description	Allowed Values
#GENERATED_BY	Assembly pipeline component that generated the output.	`cgatools`
#GENERATED_AT	Date and time of the assembly.	Year-Month-Day Time. For example "2010-Sep-08 20:27:52.457773".
#SOFTWARE_VERSION	Assembly pipeline build number.	Two or more digits separated by periods.
#FORMAT_VERSION	Version number of the file format.	Two or more digits separated by periods. For example, "0.6".
#TYPE		`SV-EVENTS`
#DBSNP_BUILD	dbSNP version used for annotation.	"`dbSNP build XXX`" where X's are digits. For example, "dbSNP build 130".
#GENE_ANNOTATIONS	NCBI annotation build.	"`NCBI build XX.X`" where X's are digits.

Content Description ASM/SV/allSvEventsBeta-[ASM-ID].tsv and ASM/SV/highConfidenceSvEventsBeta-[ASM-ID].tsv

	Column Name	Description
1	EventId	Identifier for the event. This consists of positive integers. Event Ids are consistent across all junction files for a given assembly.
2	Type	Structural rearrangement composed of one or more junctions. Possible values include: `artifact`, `complex`, `deletion`, `tandem-duplication`, `probable-inversion`, `inversion`, `distal-duplication`, `distal-duplication-by-mobile-element`, and `interchromosomal`.
3	RelatedJunctionIds	Junction identifier(s) of junctions that the event is composed of. Identifiers are semicolon-separated in cases where an event is represented by multiple junctions.
4	MatePairCounts	A number expressing the amount of DNB support available for each junction that the event is composed of. Numbers are semicolon-separated in cases where an event is represented by multiple junctions. They are in the order in which junction identifiers are listed in the *RelatedJunctionIds* field.
5	FrequenciesInBaselineGenomeSet	Frequency that the junction(s) is detected in set of baseline genomes. Numbers are semicolon-separated in cases where an event is represented by multiple junctions. They are in the order in which junction identifiers are listed in the *RelatedJunctionIds* field.
6	OriginRegionChr	Chromosome name in text: `chr1`, `chr2`,..., `chr22`, `chrX`, `chrY`. The mitochondrion is represented as `chrM`. The pseudoautosomal regions within the sex chromosomes X and Y are reported at their coordinates on chromosome X.
7	OriginRegionBegin	Reference coordinate specifying the start of the region where the indicated event is likely to have originated. The coordinate uses the half-open, zero-based coordinate system. See "Sequence Coordinate System" for more information.
8	OriginRegionEnd	Reference coordinate specifying the end of the region where the indicated event is likely to have originated. The coordinate uses the half-open, zero-based coordinate system. See "Sequence Coordinate System" for more information.

	Column Name	Description
9	OriginRegionLength	The distance between the left-most mate read and the right-most mate read in the junction cluster(s) representing the event at the origin site.
10	OriginRegionStrand	Strand ("+" or "-") of the indicated event at the origin site.
11	DestinationRegionChr	Chromosome name in text: `chr1`, `chr2`,…, `chr22`, `chrX`, `chrY`. The mitochondrion is represented as `chrM`. The pseudoautosomal regions within the sex chromosomes X and Y are reported at their coordinates on chromosome X. Values are only present for the following events: inversion, distal-duplication, distal-duplication-by-mobile-element, and interchromosomal.
12	DestinationRegionBegin	Reference coordinate specifying the start of the region where the indicated event is likely to have been inserted. The coordinate uses the half-open, zero-based coordinate system. See "Sequence Coordinate System" for more information. Values are only present for the following event types: `inversion`, `distal-duplication`, `distal-duplication-by-mobile-element`, and `interchromosomal`.
13	DestinationRegionEnd	Reference coordinate specifying the start of the region where the indicated event is likely to have been inserted. The coordinate uses the half-open, zero-based coordinate system. See "Sequence Coordinate System" for more information. Values are only present for the following event types: `inversion`, `distal-duplication`, `distal-duplication-by-mobile-element`, and `interchromosomal`.
14	DestinationRegionLength	The distance between the left-most mate read and the right-most mate read in the junction cluster(s) representing the event at the destination site.
15	DestinationRegionStrand	Strand ("+" or "-") of the indicated event at the destination site.
16	DisruptedGenes	Gene(s) overlapping at least one of the junction section positions of the event.
17	ContainedGenes	Gene(s) that are completely contained in event.
18	GeneFusions	Junction that appears to either<br>1) connect two different genes (for example, A and B) in a strand-consistent manner or<br>2) connect upstream region of gene A to an intact gene B. In the former case, fusion event is described as `A/B`, where `A` and `B` are gene symbols.<br>In the latter case, fusion event is described as `TSS-UPSTREAM[A]/B`, where A and B are gene symbols.
19	RelatedMobileElement	For duplication events caused by a mobile element, this column contains the description of the element in the format:<br>`Family:Name:DivergencePercent`<br>For example: `L1:L1HS:0.5`.<br>Information for transposed locations in the reference genome is taken from the RepeatMasker track from UCSC Genome Browser track.
20	MobileElementChr	Chromosome name in text: `chr1`, `chr2`,…, `chr22`, `chrX`, `chrY`. The mitochondrion is represented as `chrM`. The pseudoautosomal regions within the sex chromosomes X and Y are reported at their coordinates on chromosome X.
21	MobileElementBegin	Coordinate specifying the start of the consensus sequence of the specified mobile element. Uses half-open, zero-based coordinate system.
22	MobileElementEnd	Coordinate specifying the end of the consensus sequence of the specified mobile element. Uses half-open, zero-based coordinate system. See "Sequence Coordinate System" for more information.
23	MobileElementStrand	Strand ("+" or "-") of the indicated mobile element.

Mobile Element Insertion Files

The MEI Directory contains information on detected mobile element insertions and provides associated files to help researchers interpret the zygosity of the insertion events and choose the appropriate specificity and sensitivity tradeoff.

Figure 10: MEI Directory Contents

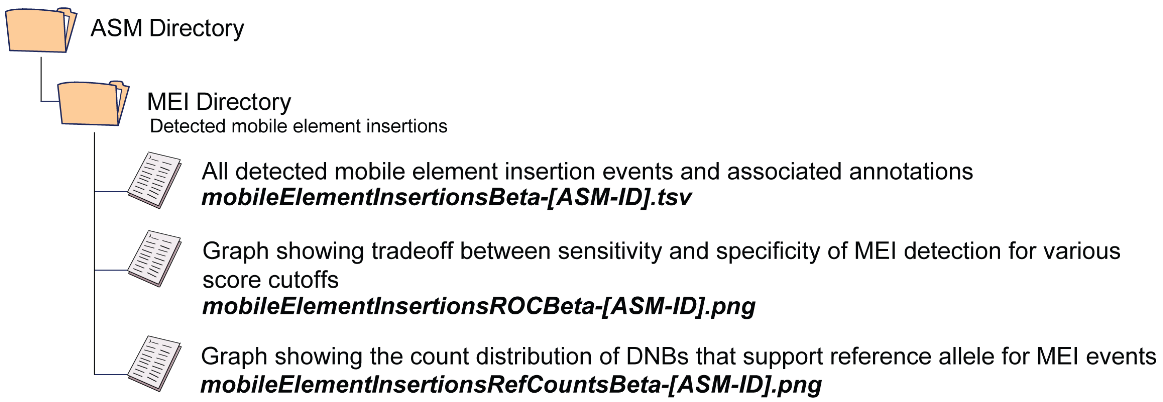

The mobile element insertion dataset describes loci of transposable element incorporation that are novel with respect to the reference genome. Candidate insertion sites are first identified by searching for DNBs that map uniquely to the reference with one arm and to ubiquitous sequence with the other arm. Only DNBs where the latter arm cannot be locally aligned to the reference sequence are considered further as candidate insertion loci. After the candidate sites are identified, the location, type, and orientation of the inserted elements are refined using the DNBs that map in the vicinity of the insertion site with one arm and cannot be mapped to the reference with the other arm. The insertion element type is determined by attempting to align each unmapped arm to the sequences of various possible mobile elements in the sequence database described in Table 8, and computing the log-likelihood score for each sequence based on these alignments. The reported *ElementType* is the element that receives the best log likelihood score. The *ElementTypeScore* is the difference between the log likelihood score for that element and the log likelihood score for the next best element. Additionally, *InsertionScore* is provided to indicate the level of confidence in the insertion event detection. This score is computed as the difference between the log likelihood score for the insertion and the log likelihood score for reference (i.e., no insertion event of any type). If the best score exceeds 10 dB, the insertion site is reported in the ***mobileElementInsertionsBeta-[ASM-ID].tsv*** results file.

Note that the 10 dB threshold is very low and was selected for the completeness of the results. The ROC curve graph in the ***mobileElementInsertionsROCBeta-[ASM-ID].png*** file is provided to facilitate selection of a threshold that would best meet your requirements on sensitivity and specificity of the MEI detection.

For each candidate mobile element insertion site, the data also includes the count of the number of DNBs that map across the insertion site—DNBs where one arm map upstream and one arm map downstream of the reference range where the insertion is likely to be located—with mate gap distance that would be unlikely had the DNBs come from the allele where the insertion was present. The count is reported in the *referenceDnbCount* field of the ***mobileElementInsertionsBeta-[ASM-ID].tsv*** file and allows determination of the zygosity of the MEI events. A distribution graph of these counts for the sequenced genome is provided in ***mobileElementInsertionsRefCountsBeta-[ASM-ID].png*** to help with the selection of the appropriate threshold to separate heterozygous and homozygous events.

Table 8: Mobile Element Sequence Database

Element Family	Element Types	Source of Consensus Sequence
ALU	AluJo, AluJb, AluSc, AluSg, AluSp, AluSq, AluSx, AluSz, AluY, AluYa5, AluYa8, AluYb8, AluYb9, AluYc1, AluYc2, AluYd2, AluYd3, AluYd8, AluYa1, AluYa4, AluYg6, AluYh9, AluYi6	Consensus sequences from RepeatMasker database. All ALU subtypes are included for completeness; the types outside of the AluY subfamily are rarely polymorphic.<br>See RepeatMasker in "References" for more information.
LINE	L1HS, L1MA3, L1MA5, L1MA7, L1PA2, L1PA3, L1PA4, L1PA5, L1PA7, L1PA10, L1PA11, L1PA13, L1PA15, L1PA17_5, L1PREC2	Consensus sequences from RepeatMasker database. All LINE types that are known to be polymorphic in human population based on the deletions data or previous publications are included.
ERV	HERVK	Consensus sequence from RepeatMasker database. This subtype is known to be polymorphic based on the deletion data.
LTR	LTR12C, LTR2, LTR22B, LTR5, LTR5_Hs	Consensus sequences from RepeatMasker database. All LTR types that are known to be polymorphic in human population based on the deletions data or previous publications are included.
MER	MER11A, MER11B, MER11C	Consensus sequences from RepeatMasker database. MER11C is known to be polymorphic based on the deletion data, and the related subtypes are included for completeness, although they are rarely polymorphic.
SVA	SVA, SVA_F1, SVA_A, SVA_B, SVA_C, SVA_D, SVA_E, SVA_F	Consensus sequences from RepeatMasker database, with the exception of SVA_F1, which was extracted from the reference genome based on the data in the publication Damert[1] et al.

[1] *Damert A., et al. "5'-Transducing SVA retrotransposon groups spread efficiently throughout the human genome". Genome Res. 2009. Hg18 locus chr10:101587199-101590749, strand adjusted.*

Mobile Element Insertion Sites

ASM/MEI/mobileElementInsertionsBeta-[ASM-ID].tsv

This file gives information for individual putative mobile element insertion events that were detected in the sequenced genome.

Example *ASM/MEI/mobileElementInsertionsBeta-[ASM-ID].tsv*

The first section shows the first 14 columns; the remaining 5 columns appear in the lower section. The second section of data repeats the *Chromosome* column at the left edge to more easily match the data with the previous section of data; the *Chromosome* column is not repeated in the actual data.

>Chromosome	InsertRangeBegin	InsertRangeEnd	Strand	ElementType	ElementTypeScore	ElementSequenceBegin	ElementSequenceEnd	NextBestElementType	InsertionScore	InsertionDnbCount	InsertionLeftDnbCount	InsertionRightDnbCount	ReferenceDnbCount
chr1	13669146	13669374	-	AluSq	200	8	308	AluSp	568	34	2	32	N
chr1	14308791	14309259	-	AluSz	0	38	295	AluSx	377	16	11	5	N
chr1	16024501	16024531	-	AluYc2	8	223	109	AluYg6	78	5	5	0	0
chr1	16027996	16028051	-	AluYd3	0	8	49	AluYd2	90	5	0	5	0
chr1	16623244	16623386	+	AluYi6	224	21	312	AluY	1201	49	34	15	31
chr1	16763053	16763083	-	AluYb8	0	30	309	AluYb9	93	5	5	0	254
chr1	16766550	16767061	-	L1PREC2	15	4475	4513		15	1	0	1	N
chr1	16796809	16797031	+	AluYb8	29	10	329	AluYb9	250	14	4	10	N

>Chromosome	GeneOverlap	XRef	FrequencyInBaseline	NovelEventCountForScore	KnownEventSensitivityForScore
chr1			1	637	0.966
chr1		ALU:P1_MEI_1340	0.558	797	0.975
chr1			0.154	1816	0.997
chr1			0.192	1678	0.996
chr1	SPATA21:-:INTRON		1	381	0.863
chr1	NBPF1:-:EXON		0.673	1646	0.996
chr1	NBPF1:-:EXON		0.577	3427	1
chr1	NBPF1:-:INTRON		0.596	996	0.988

File-Specific Header Description ASM/MEI/mobileElementInsertionsBeta-[ASM-ID].tsv

Key	Description	Allowed Values
#ASSEMBLY_ID	Name of the assembly	"<assembly-name>-ASM". For example, "GS000000474-ASM".
#SOFTWARE_VERSION	Assembly pipeline build number	Two or more digits separated by periods.
#GENERATED_BY	Assembly pipeline component that generated the output.	Alpha-numeric string.
#GENERATED_AT	Date and time of the assembly	Year-Month-Day Time. For example "2010-Sep-08 20:27:52.457773".
#FORMAT_VERSION	Version number of the file format	Two or more digits separated by periods. For example, "0.6".
#GENOME_REFERENCE	Human genome build used for assembly	"NCBI build XX" where X's are digits.
#SAMPLE	Complete Genomics identifier of the sample from which the library was created	"GSXXXXX-DNA_YZZ" where<br>▪ X's are digits<br>▪ -DNA_ is literal<br>▪ YZZ is the location of the sample in a 96-well plate with Y as one of "A" through "H" and ZZ is one of "01" through "12"<br>For example "GS12345-DNA_A01".
#TYPE	Indicates the type of data contained in the file	"MEI": mobile element insertions detected.
#GENE_ANNOTATIONS	NCBI annotation build	"NCBI build XX.X" where X's are digits.
#MEI_1000G_ANNOTATIONS	Version of the 1000 genomes data set used for annotations	"INITIAL-DATA-RELEASE".

Content Description ASM/MEI/mobileElementInsertionsBeta-[ASM-ID].tsv

	Column Name	Description
1	Chromosome	Left chromosome name in text: chr1, chr2,..., chr22, chrX, chrY. The pseudoautosomal regions within the sex chromosomes X and Y are reported at their coordinates on chromosome X. The mitochondrion is currently excluded from the mobile element detection pipeline.
2	InsertRangeBegin	Reference coordinate specifying the start of the region where the insertion event is likely to reside using the half-open, zero-based coordinate system. See "Sequence Coordinate System" for more information.
3	InsertRangeEnd	Reference coordinate specifying the end of the region where the insertion event is likely to reside using the half-open, zero-based coordinate system. See "Sequence Coordinate System" for more information.
4	Strand	Strand ("+" or "-") of the inserted element.
5	ElementType	Family and type of the element. For most mobile elements, Repeat Masker names are used, for example "AluYa5" or "L1HS".
6	ElementTypeScore	Phred-like confidence that the element type was detected correctly, based on the likelihood ratio with the next most likely element type.
7	ElementSequenceBegin	Coordinate specifying the start of the inserted fragment within the consensus sequence of the mobile element. While ALU mobile elements tend to be inserted intact, L1 and SVA mobile elements are frequently truncated at one or both ends. Uses half-open, zero-based coordinate system.

	Column Name	Description
8	ElementSequenceEnd	Coordinate specifying the end of the inserted fragment within the consensus sequence of the mobile element. While ALU mobile elements tend to be inserted intact, L1 and SVA mobile elements are frequently truncated at one or both ends. Uses half-open, zero-based coordinate system.
9	NextBestElementType	Element type that received the next best score after the type reported in the *ElementType* column.
10	InsertionScore	Phred-like confidence that the insertion is present at this locus, based on the likelihood ratio with no insertion of any type. If the best score exceeds 10 dB, the insertion site is reported.
11	InsertionDnbCount	Total number of DNBs that support the insertion at this locus.
12	InsertionLeftDnbCount	Number of DNBs that support the insertion and map to the reference upstream of the insertion site with one of the arms.
13	InsertionRightDnbCount	Number of DNBs that support the insertion and map to the reference downstream of the insertion site with one of the arms.
14	ReferenceDnbCount	Number of DNBs that contradict the insertion hypothesis and support the reference allele at this locus. Value can be 'N' in cases where this count cannot be determined.
15	GeneOverlap	Gene overlapping the insertion range. The content of this field has the following format: `NCBI-GENE-SYMBOL:STRAND:EXON-OR-INTRON` For example: `DDEFL1:-:INTRON` Multiple gene entries are semicolon separated.
16	XRef	Cross-reference to the events in 1000 genomes MEI dataset, in the following format: `TYPE:ID`. For example: `ALU:P1_MEI_3277`
17	FrequencyInBaseline	Frequency this event was detected in the set of baseline genomes.
18	NovelEventCountFor InsertionScore	Count of novel events (with respect to the 1000 genomes dataset) detected with the score of this event or higher.
19	KnownEventSensitivityFor InsertionScore	Fraction of known events (that is, present in 1000 genomes dataset) that are detected with the score of this event or higher.

Mobile Element Insertion ROC Graph

ASM/MEI/mobileElementInsertionsROCBeta-[ASM-ID].png

This graph shows the relationship between the number of the novel events detected and the sensitivity to the known (present in 1000 genomes data set) events as a function of insertion score. Score cutoffs that result in 50%, 75%, 90%, 95%, and 98% sensitivity to the known events are annotated on the graph. In Figure 11 for example, filtering of insertion events with a score cutoff of 692 results in 95% sensitivity to the known events, and approximately 580 novel events detected. The thin blue line shows the same relationship based on the DNB count cutoffs instead of the score cutoffs.

Figure 11: Mobile Element Insertion ROC Graph

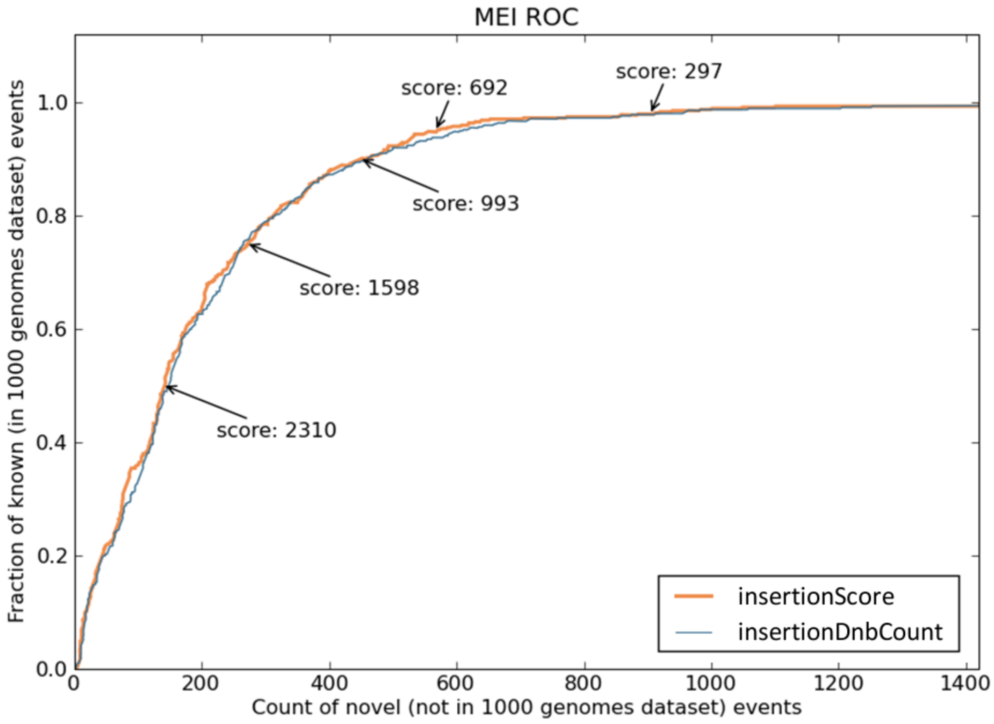

Mobile Element Insertion Reference Counts Graph

ASM/MEI/mobileElementInsertionsRefCountsBeta-[ASM-ID].png

This graph shows the distribution of the DNB counts that support reference allele for known insertion events detected in the sequenced genome. As in Figure 12, this distribution is usually bi-modal, corresponding to the homozygous insertions (peaking at zero DNBs) and heterozygous insertions (centered at approximately 80 DNBs for this genome). The optimal threshold that separates homozygous and heterozygous insertions depends on the coverage; for the genome in Figure 12, the distributions are well separated such that any threshold between 10 to 30 DNBs would be reasonable.

Figure 12: Mobile Element Insertion Reference Counts Graph

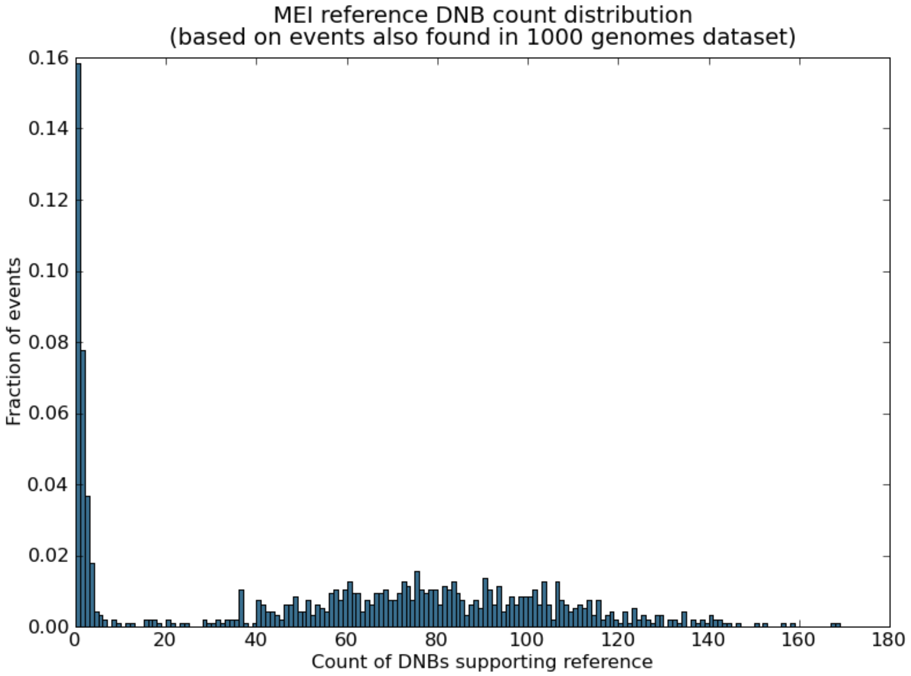

Assemblies Underlying Called Variants Files

The EVIDENCE Directory contains supporting information for intervals in the reference sequence where there is substantial evidence for variations from the reference sequence. The assembly software ordinarily first identifies locations on the genome where variations from the homozygous reference are suggested, and then attempts to resolve the sequence at these locations by synthesizing the available evidence using local *de novo* assembly. This directory contains files that enumerate these locations on the genome, list the allele sequences corresponding with the most likely diploid hypothesis at each location, and list the individual DNB reads and their alignments supporting each allele and the alternative reference sequence hypothesis.

Figure 13: EVIDENCE Directory Contents

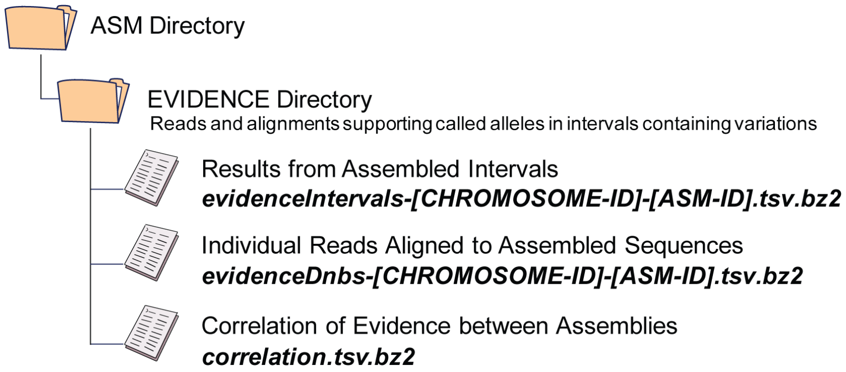

To handle segmental duplications and similar sequences in the reference, the Complete Genomics assembly process can incorporate some reads into more than one assembly, and these reads will be weighted as evidence by the alignment probabilities to each region's alleles. When pairs of genomic intervals share a subset of reads, information is provided on the pairwise correlations between those intervals. These correlation scores form additional criteria for accepting or rejecting a variation call.

For normal genomes, the information in this directory allows for a detailed investigation of the supporting evidence for each allele. For abnormal genomes such as tumors, in which both the ploidy and purity might vary, this information might help assess the strength of evidence for putative novel alleles observed.

The ***evidenceIntervals-[CHROMOSOME-ID]-[ASM-ID].tsv.bz2*** file provides data for intervals where there is substantial evidence for variations from the reference sequence. Data is only reported for the genomic intervals that satisfy the following criteria:

- The most likely hypothesis explaining the observed data differs from the homozygous reference hypothesis,
- The most likely hypothesis is more likely than the homozygous reference hypothesis by a threshold (currently a score difference of 10).

Note that the criteria for reporting interval data in this file is less stringent than that required to make a homozygous and heterozygous variant call. Thus, it is possible that interval data for a no-call locus are included in the file.

The ***evidenceDnbs-[CHROMOSOME-ID].tsv.bz2*** file provides, for each allele, alignments for all DNBs that support one of the alleles reported over another by a score difference of 3. Because of this score difference criterion, it is possible that alignments for some DNBs that support the called allele are not included in the ***evidenceDnbs-[CHROMOSOME-ID].tsv.bz2*** file. In addition, only the best alignment is shown for each DNB-allele pair. The data of each type (evidence intervals, evidence DNBs) are split into several files, one for each chromosome.

The EVIDENCE Directory contains supporting information for intervals in the reference sequence where there is substantial evidence for variations from the reference sequence. This information may be converted to other formats such as SAM. For more information, see the *CGA Tools User Guide*.

Alignment CIGAR Format

Alignments of DNBs and alleles to the reference sequence are represented in the evidence files in a "CIGAR-like" format, which resembles the CIGAR representation used in SAM format files. It has additional features to support overlaps in the DNB structure, as can occur between reads r1 and r2 or between r3 and r4 in the DNB architecture depicted in Figure 1.

The CIGAR representation is a concatenation of a sequence of integers and modifiers. For example, "10M3N10M" denotes an alignment with 10 matching or mismatching bases, followed by a 3-base gap, followed by 10 matching or mismatching bases. For DNB alignments to an allele or reference sequence reported in the file ***evidenceDnbs-[CHROMOSOME-ID]-[ASM-ID].tsv.bz2***, the modifiers may be interpreted as described in Table 9.

Table 9: Alignment CIGAR Format Modifiers in *evidenceDnbs-[CHROMOSOME-ID]-[ASM-ID].tsv.bz2*

CIGAR Modifier	Description
**M**	Position within a DNB read that aligns to a base of sequence (can be a match, a mismatch, or a no-call).
**N**	Bases in the sequence corresponding to a gap in the DNB (unsequenced bases between reads).
**B**	Bases in the sequence corresponding to an overlap between consecutive reads within a DNB.
**I**	Bases in the DNB that correspond to an insertion within the sequence to which it is aligned.
**P**	Gap bases in the DNB (unsequenced bases between reads) that correspond to an insertion of bases within the sequence to which it is aligned.
**D**	Bases in the sequence that are deleted within the DNB.

The CIGAR format is also used to represent the alignments of alleles to the reference sequence in ***evidenceIntervals-[CHROMOSOME-ID]-[ASM-ID].tsv.bz2***. For these alignments, the modifiers are as follows in Table 10.

Table 10: Alignment CIGAR Format Modifiers in *evidenceIntervals-[CHROMOSOME-ID]-[ASM-ID].tsv.bz2*

CIGAR Modifier	Description
**M**	Position where the allele and reference sequence are aligned (can be a match, a mismatch, or a no-call)
**I**	Bases in the allele that are an insertion with respect to the reference sequence.
**D**	Bases in the reference sequence that are deleted within the allele.

Results from Assembled Intervals

ASM/EVIDENCE/evidenceIntervals-[CHROMOSOME-ID]-[ASM-ID].tsv.bz2

The ***evidenceIntervals-[CHROMOSOME-ID]-[ASM-ID].tsv.bz2*** file includes results of the assembled intervals.

Example ***ASM/EVIDENCE/evidenceIntervals-[CHROMOSOME-ID]-[ASM-ID].tsv.bz2***

>IntervalId	Chromosome	OffsetInChromosome	Length	Ploidy	AlleleIndexes	EvidenceScoreVAF	EvidenceScoreEAF	Allele0	Allele1
60	chr22	16063032	33	2	0;1	248	241	GAGAACAGCCTGGGCAACAAAGTGAGACCCAAT	GAGTACAGCCTTGGCAACAAAGTGAGACCTAAT
61	chr22	16063078	37	2	0;1	103	86	AGAGAAAAAATAGCTGGGTGTGGTGGCACTCACCTGT	AGAGAAAAAATAGCTGGGTGTGTTGGCACTCACCTGT
62	chr22	16063140	46	2	1;2	640	636	CTCCTGAGCCTAGTTGGTTGAGGCTGCAGTGAGCCAAGATCATGCC	CTCCTGAGCCTAGGTGGTTGAGGCTGCAGTGAGCCAAGATCACACC
63	chr22	16063250	44	2	0;1	100	86	ACAATAACTTTGGTTTTGTCACTAATATGCTGAATATTTTTGTT	ACAGTAACTTTGGTTTTGTCACTAATACGCTGAATATTTTTGTT
64	chr22	16063342	51	2	0;1	63	37	TGGTATTAGCTGTCCTACGTACCTGACGACCTAATGCTTAACCTAATGTTC	TGGAATTAGCTCTCCTGCATACCTGATGACCTAATGCTTAACCTAATCTTC
65	chr22	16063420	30	3	1;2;3	370	354	GTTGCAATAGAGTTCTTTACCCCAAAGTCT	GTTACAAGAGAGTTCTTTACCCCAAAGTCT

>IntervalId	Allele2	Allele3	Allele1Alignment	Allele2Alignment	Allele3Alignment
60			33M		
61			37M		
62	CTCTTGAGCCTAGGTGGTTGAGGCTGCAGTGAGCCAAGATCATGCC		46M	46M	
63			44M		
64			51M		
65	GTTACAAGAGAGTTCTTTACCTCAAAGTCT	GTTGCAATAGAGTTCTTTACCCCAAAATCT	30M	30M	30M

File-Specific Header Description ASM/EVIDENCE/evidenceIntervals-[CHROMOSOME-ID]-[ASM-ID].tsv.bz2

Key	Description	Allowed Values
#ASSEMBLY_ID	Name of the assembly	"<assembly-name>-ASM". For example, "GS000000474-ASM".
#CHROMOSOME	Identifier of the chromosome that the reference score and coverage data apply to. Data for the pseudo-autosomal regions on chromosome Y are reported at their coordinates on chromosome X.	chr1-chr22, chrM, chrX, chrY
#FORMAT_VERSION	Version number of the file format	Two or more digits separated by periods. For example, "0.6".
#GENERATED_AT	Date and time of the assembly	Year-Month-Day Time. For example "2010-Sep-08 20:27:52.457773".
#GENERATED_BY	Assembly pipeline component that generated the output.	Alpha-numeric string
#SAMPLE	Complete Genomics identifier of the sample from which the library was created	"GSXXXXX-DNA_YZZ" where<br>▪ X's are digits<br>▪ -DNA_ is literal<br>▪ YZZ is the location of the sample in a 96-well plate with Y as one of "A" through "H" and ZZ is one of "01" through "12"<br>For example "GS12345-DNA_A01".
#SOFTWARE_VERSION	Assembly pipeline build number	Two or more digits separated by periods.
#TYPE	Indicates the type of data contained in the file.	"EVIDENCE-INTERVALS": genomic intervals over which supporting evidence is provided for the called sequence.

Content Description ASM/EVIDENCE/evidenceIntervals-[CHROMOSOME-ID]-[ASM-ID].tsv.bz2

	Column Name	Description
1	IntervalId	Identifier for this evidence interval. Cross-referenced with the *IntervalId* in the ***evidenceDnbs*** file.
2	Chromosome	Chromosome name in text: chr1, chr2,..., chr22, chrX, chrY. The mitochondrion is represented as chrM. The pseudoautosomal regions within the sex chromosomes X and Y are reported at their coordinates on chromosome X.
3	OffsetInChromosome	Reference coordinate specifying the start of the genomic interval. Uses the half-open zero-based coordinate system. See "Sequence Coordinate System" for more information.
4	Length	Length in bases of the evidence interval.
5	Ploidy	Ploidy of the sequence over the interval. The *Ploidy* value is 1 for the non-pseudoautosomal fractions of the sex chromosomes in a male genome; the value is 2 otherwise.
6	AlleleIndexes	Semicolon-separated indices of the alleles in the called sequence. *Allele0* is always the reference allele. The number of alleles equals the ploidy specified for the interval. For example, for a diploid interval in which the Assembly software predicts heterozygosity with one copy each of allele 0 and allele 1, *AlleleIndexes* would be "0;1". A diploid interval with a single homozygous SNP predicted within it would have *AlleleIndexes* = "1;1".
7	EvidenceScoreVAF	Score representing the strength of evidence for the called sequence over the interval, based on the Variable Allele Fraction model.

	Column Name	Description
8	EvidenceScoreEAF	Score representing the strength of evidence for the called sequence over the interval, based on the Equal Allele Fraction model.
9	Allele0	The sequence of Allele0, which by construction is identical to the reference genome over the evidence interval.
10	Allele1	The sequence of Allele1, which must differ from the reference sequence.
11	Allele2	The sequence of Allele2, which must differ from the reference sequence. Blank unless the most likely sequence hypothesis has two non-reference alleles.
12	Allele3	The sequence of Allele3, which must differ from the reference sequence. Blank unless the most likely sequence hypothesis has three non-reference alleles.
13	Allele1Alignment	The alignment of Allele1 to the reference genome, specified in a CIGAR format (see "Alignment CIGAR Format" for details).
14	Allele2Alignment	The alignment of Allele2 to the reference genome, specified in a CIGAR format (see "Alignment CIGAR Format" for details). Blank when Allele2 is absent.
15	Allele3Alignment	The alignment of Allele3 to the reference genome, specified in a CIGAR format (see "Alignment CIGAR Format" for details).

Individual Reads Aligned to Assembled Sequences

ASM/EVIDENCE/evidenceDnbs-[CHROMOSOME-ID]-[ASM-ID].tsv.bz2

Example *ASM/EVIDENCE/evidenceDnbs-[CHROMOSOME-ID]-[ASM-ID].tsv.bz2*

The first section shows the first 12 columns; the remaining 9 columns appear in the lower section, with the sequence and score data truncated.

>IntervalId	Chromosome	Slide	Lane	FileNumInLane	DnbOffsetInLaneFile	AlleleIndex	Side	Strand	OffsetInAllele	AlleleAlignment	OffsetInReference
65	chr22	GS14642-FS3	L08	3	3579906	0	R	+	-29	10M6N10M0N10M2B5M	16063391
65	chr22	GS14642-FS3	L08	5	10399222	0	R	+	-5	10M6N10M0N10M2B5M	16063415
65	chr22	GS14643-FS3	L02	4	24165207	0	L	+	-9	5M2B10M0N10M7N10M	16063411
65	chr22	GS14643-FS3	L02	5	24213764	0	R	+	24	10M6N10M1N10M2B5M	16063444
65	chr22	GS14643-FS3	L02	6	29606652	0	R	-	-34	5M2B10M0N10M6N10M	16063386
65	chr22	GS14643-FS3	L04	5	1001590	0	R	-	-12	5M2B10M0N10M5N10M	16063408
65	chr22	GS14643-FS3	L05	2	25412715	0	R	-	-2	5M2B10M0N10M6N10M	16063418
65	chr22	GS14643-FS3	L05	3	20979119	0	R	-	14	5M2B10M0N10M5N10M	16063434
65	chr22	GS14643-FS3	L06	7	2297379	0	L	-	17	10M5N10M0N10M2B5M	16063437
65	chr22	GS14643-FS3	L07	3	7496185	0	R	-	13	5M2B10M0N10M6N10M	16063433

>IntervalId	ReferenceAlignment	MateOffsetInReference	MateReferenceAlignment	MappingQuality	ScoreAllele0	ScoreAllele1	ScoreAllele2	ScoreAllele3	Sequence	Scores
0	10M6N10M0N10M2B5M	16062954	5M2B10M0N10M6N10M	$	3	0	0	3	AGGTGTGCGGTGG	8991443637677667;…
0	10M6N10M0N10M2B5M	16063029	5M2B10M0N10M6N10M	$	3	0	0	0	TTTGAGAGAACAG	898:7007386*6767;…
0	5M2B10M0N10M7N10M	16063796	10M7N10M0N10M2B5M	9	24	0	0	0	TGGAAAAACTTGT	89:::4**')767778;…
0	10M6N10M1N10M2B5M	16063112	5M2B10M0N10M7N10M	?	30	30	30	2	TGTACACTAAGGA	89:;:7.05646/668;…
0	5M2B10M0N10M6N10M	16063803	10M5N10M0N10M2B5M	$	3	0	0	3	CAAAAAACAGATT	894::65897457768:…
0	5M2B10M0N10M5N10M	16063801	10M6N10M0N10M2B5M	'	6	0	0	3	AAAACACAGATTT	899;:88388666568:…
0	5M2B10M0N10M6N10M	16063830	10M5N10M0N10M2B5M	$	3	0	0	3	ATTAGAGAAAAAA	899:1,,6*2667748:;…
0	5M2B10M0N10M5N10M	16063815	10M5N10M0N10M2B5M	.	13	13	0	0	GGGAAAAGATAGA	899:987388677668:…
0	10M5N10M0N10M2B5M	16063078	5M2B10M0N10M5N10M	G	38	38	8	9	TTCTCTCGTCTTG	898:7$0/-0672677;…
0	5M2B10M0N10M6N10M	16063922	10M6N10M0N10M2B5M	8	23	23	1	0	GCTCTCTGCTTGT	6297967255367768;…

© Complete Genomics, Inc.

File-Specific Header Description ASM/EVIDENCE/evidenceDnbs-[CHROMOSOME-ID]-[ASM-ID].tsv.bz2

Key	Description	Allowed Values
#ASSEMBLY_ID	Name of the assembly	"<assembly-name>-ASM". For example, "GS000000474-ASM".
#CHROMOSOME	Identifier of the chromosome that the reference score and coverage data apply to. Data for the pseudo-autosomal regions on chromosome Y are reported at their coordinates on chromosome X.	chr1-chr22, chrM, chrX, chrY
#FORMAT_VERSION	Version number of the file format	Two or more digits separated by periods. For example, "0.6".
#GENERATED_AT	Date and time of the assembly	Year-Month-Day Time. For example "2010-Sep-08 20:27:52.457773".
#GENERATED_BY	Assembly pipeline component that generated the output	Alpha-numeric string.
#SAMPLE	Complete Genomics identifier of the sample from which the library was created	"GSXXXXX-DNA_YZZ" where<br>▪ X's are digits<br>▪ -DNA_ is literal<br>▪ YZZ is the location of the sample in a 96-well plate with Y as one of "A" through "H" and ZZ is one of "01" through "12"<br>For example "GS12345-DNA_A01".
#SOFTWARE_VERSION	Assembly pipeline build number	Two or more digits separated by periods.
#TYPE	Type of data contained in the file	"EVIDENCE-DNBS": DNB alignments supporting the called alleles in a genomic interval.

Content Description ASM/EVIDENCE/evidenceDnbs-[CHROMOSOME-ID]-[ASM-ID].tsv.bz2

	Column Name	Description
1	IntervalId	Identifier for this evidence interval. Cross-referenced with the *IntervalId* from the ***evidenceIntervals*** file.
2	Chromosome	Chromosome name in text: chr1, chr2,…,chr22, chrX, chrY. The mitochondrion is represented as chrM. The pseudoautosomal regions within the sex chromosomes X and Y are reported at their coordinates on chromosome X.
3	Slide	Identifier for the Slide from which data for this half-DNB was obtained.
4	Lane	Identifier for the lane within the slide from which data for this half-DNB was obtained.
5	FileNumInLane	The file number of the reads file describing this DNB. (For example, *X* in ***reads_[SLIDE-LANE]_00X.tsv.bz2***.)
6	DnbOffsetInLaneFile	Record within data for the slide lane in ***reads_[SLIDE-LANE]_00X.tsv.bz2*** that corresponds to this DNB.
7	AlleleIndex	An index specifying the allele this half-DNB mapping supports the most. If the half-DNB mapping supports two alleles equally well, another record for the half-DNB mapping is created in the file, where *AlleleIndex* specifies the second allele. The sequence of the allele and its alignment to the reference are specified in ***evidenceIntervals-[CHROMOSOME-ID]-[ASM-ID].tsv.bz2***. (see "Alignment CIGAR Format").

	Column Name	Description
8	Side	A single character, "L" or "R", specifying the location of this half-DNB within the DNB. For DNBs with the architecture specified in Figure 1, "L" refers to bases 1 through 30 of the 60-base DNB read set; "R" refers to bases 31 through 60.
9	Strand	The strand of the half-DNB, "+" or "-", expressed relative to the reference genome.
10	OffsetInAllele	The position at which the half-DNB starts (as seen on the "+" strand) relative to the start of the allele sequence in the evidence interval. The offset may be positive or negative.
11	AlleleAlignment	The alignment of the half-DNB to the allele sequence, provided in an extended CIGAR format (see "Alignment CIGAR Format").
12	OffsetInReference	The chromosomal position on the reference genome at which the half-DNB starts (as seen on the "+" strand).
13	ReferenceAlignment	The alignment of the half-DNB to the reference genome, specified in a CIGAR format (see "Alignment CIGAR Format").
14	MateOffsetInReference	The chromosomal position at which the mate of this half-DNB starts on the reference genome.
15	MateReferenceAlignment	Alignment of the mate of this half-DNB to the reference genome, specified in a CIGAR format (see "Alignment CIGAR Format").
16	MappingQuality	A Phred-like encoding of the probability that this half-DNB mapping is correct, encoded as a single character with ASCII-33. The mapping quality is related to the existence of alternate mappings; the Phred score is obtained by subtracting 33 from the ASCII code of the character.
17	ScoreAllele0	A value proportional to `log P(DNB|G0)`, where `G0` is the reference genome. *ScoreAllele0*, *ScoreAllele1*, and *ScoreAllele2* for a given DNB within an interval can be compared. For example, the difference in *ScoreAllele0* and *ScoreAllele1* equals the likelihood ratio in decibel of this DNB. A higher "score" indicates the DNB had better alignments to the given allele.
18	ScoreAllele1	A value proportional to `log P(DNB|G1)`, where `G1` is the reference genome with both alleles replaced by Allele1 in the region of interest. *ScoreAllele0*, *ScoreAllele1*, and *ScoreAllele2* for a given DNB within an interval can be compared. For example, the difference in *ScoreAllele0* and *ScoreAllele1* equals the likelihood ratio in decibel of this DNB. A higher "score" indicates the DNB had better alignments to the given allele.
19	ScoreAllele2	A value proportional to `log P(DNB|G2)`, where `G2` is the reference genome with both alleles replaced by Allele2 in the region of interest. *ScoreAllele0*, *ScoreAllele1*, and *ScoreAllele2* for a given DNB within an interval can be compared. For example, the difference in *ScoreAllele0* and *ScoreAllele1* equals the likelihood ratio in decibel of this DNB. A higher "score" indicates the DNB had better alignments to the given allele.
20	ScoreAllele3	A value proportional to `log P(DNB|G3)`, where `G3` is the reference genome with both alleles replaced by Allele2 in the region of interest. *ScoreAllele0*, *ScoreAllele1*, and *ScoreAllele2* for a given DNB within an interval can be compared. For example, the difference in *ScoreAllele0* and *ScoreAllele1* equals the likelihood ratio in decibel of this DNB. A higher "score" indicates the DNB had better alignments to the given allele.
21	Sequence	Sequence of the DNB arm bases in the DNB order (same as in the ***reads_[SLIDE-LANE]_00X.tsv.bz2*** file).
22	Scores	Phred-like error scores for DNB bases in the DNB order, not separated (same as in the ***reads_[SLIDE-LANE]_00X.tsv.bz2*** file).

Correlation of Evidence between Assemblies

ASM/EVIDENCE/correlation.tsv.bz

The correlation file ***correlation.tsv.bz2*** describes the results of a pairwise correlation analysis of all pairs of genomic intervals that share evidence from some of the same DNBs – this can happen when DNBs map well to more than one location on the genome (for example, segmental duplications or regions with tandem repeats). The analysis evaluates the likelihood of three two-region hypotheses with respect to the reference hypothesis:

- that a non-reference allele occurs only in the first region,
- that a non-reference allele occurs only in the second region, and
- that a non-reference allele occurs in both regions.

The relative likelihood for each hypothesis to the null (reference) hypothesis is reported in Phred-like scores. The Assembly software uses evidence of correlations among called loci to no-call one or both instances of putative variations.

Example ***ASM/EVIDENCE/correlation.tsv.bz***

>Chromosome1	OffsetInChromosome1	Length1	Chromosome2	OffsetInChromosome2	Length2	P1	P2	P12
chr1	13105032	14	chr2	190497232	18	185	97	248
chr1	13105032	14	chr14	20749158	18	185	60	211
chr1	13105219	24	chr2	190497045	7	728	173	890
chr1	13105219	24	chr2	190497528	7	728	68	792
chr1	13105482	50	chr2	190497232	18	1434	97	1361
chr1	13105482	50	chr14	20749158	18	1434	60	1341
chr1	13105721	37	chr2	190497045	7	138	173	275
chr1	13106094	24	chr2	190497085	14	250	952	1203
chr1	13108848	44	chr1	13230405	48	119	2200	2320
chr1	13108848	44	chr1	13230487	35	119	460	579
chr1	13108848	44	chr1	13279277	13	119	109	134
chr1	13108848	44	chr1	13279740	7	119	40	155
chr1	13108848	44	chr1	13291798	32	119	2078	2196
chr1	13108848	44	chr1	13386265	19	119	478	596
chr1	13108848	44	chr1	13386739	19	119	327	445
chr1	13108848	44	chr1	13500074	13	119	109	134
chr1	13108848	44	chr1	13500537	7	119	40	155
chr1	13108848	44	chr1	13512595	32	119	2078	2196
chr1	13108848	44	chr1	13607106	19	119	567	685
chr1	13108848	44	chr1	13607580	19	119	443	561

File-Specific Header Description ASM/EVIDENCE/correlation.tsv.bz

Key	Description	Allowed Values
#ASSEMBLY_ID	Name of the assembly	"<assembly-name>-ASM". For example, "GS000000474-ASM".
#FORMAT_VERSION	Version number of the file format	Two or more digits separated by periods. For example, "0.6".
#GENERATED_AT	Date and time of the assembly	Year-Month-Day Time. For example "2010-Sep-08 20:27:52.457773".
#GENERATED_BY	Assembly pipeline component that generated the output	Alpha-numeric string.
#SAMPLE	Complete Genomics identifier of the sample from which the library was created	"GSXXXXX-DNA_YZZ" where<br>▪ X's are digits<br>▪ -DNA_ is literal<br>▪ YZZ is the location of the sample in a 96-well plate with Y as one of "A" through "H" and ZZ is one of "01" through "12"<br>For example "GS12345-DNA_A01".
#SOFTWARE_VERSION	Assembly pipeline build number	Two or more digits separated by periods.
#TYPE	Indicates the type of data contained in the file	"EVIDENCE-CORRELATION": information on correlations in supporting data between pairs of genomic intervals.

Content Description ASM/EVIDENCE/correlation.tsv.bz

	Column Name	Description
1	Chromosome1	Chromosome name for the first interval in text: chr1, chr2, …, chr22, chrX, chrY. The mitochondrion is represented as chrM. The pseudoautosomal regions within the sex chromosomes X and Y are reported at their coordinates on chromosome X.
2	OffsetInChromosome1	Reference coordinate specifying the start of the first genomic interval. Uses the half-open zero-based coordinate system. See "Sequence Coordinate System" for more information.
3	Length1	Length in bases of the first evidence interval.
4	Chromosome2	Chromosome name for the second interval in text: chr1, chr2, …, chr22, chrX, chrY. The mitochondrion is represented as chrM. The pseudo-autosomal regions within the sex chromosomes X and Y are reported at their coordinates on chromosome X.
5	OffsetInChromosome2	Reference coordinate specifying the start of the second genomic interval. Uses the half-open zero-based coordinate system. See "Sequence Coordinate System" for more information.
6	Length2	Length in bases of the second evidence interval.
7	P1	Score representing the likelihood of the hypothesis that a non-reference allele exists in the first interval and the second interval is homozygous reference.
8	P2	Score representing the likelihood of the hypothesis that a non-reference exists in the second interval and the first interval is homozygous reference.
9	P12	Score representing the likelihood of the hypothesis that a non-reference allele exists in both intervals.

Coverage and Reference Scores Files

The REF Directory contains the coverage and reference score data for each base position of the reference genome. The data are split into several files, named ***coverageRefScore-[CHROMOSOME-ID]-[ASM-ID].tsv.bz2***, one corresponding to each chromosome. The chromosome number is also represented in the header key "#CHROMOSOME".

Figure 14: REF Directory Contents

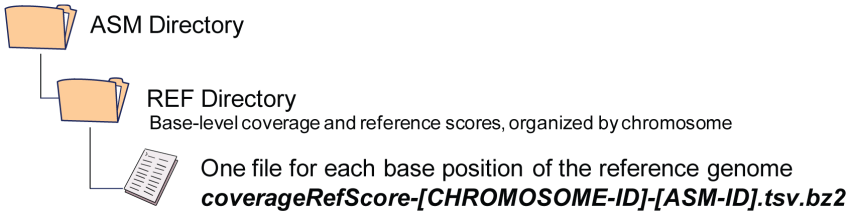

Coverage and Reference Scores

ASM/REF/coverageRefScore-[CHROMOSOME ID]-[ASM ID].tsv.bz2

Four coverage numbers are reported: The *uniqueSequenceCoverage* represents the number of fully (for example, both DNB ends) mapped DNBs that overlap each base position and that map only to this location. More precisely, it counts all full-DNB mappings that have a mapping weight ratio of 0.99:1 or better supporting its placement at this position. The *weightSumSequenceCoverage*, by contrast, computes the sum of all DNBs which may map to this location, each weighted by their mapping weight ratio. The *gcCorrectedCoverage* represents the weight-sum of all DNBs which may map to this location, corrected by GC bias as described in "Copy Number Variation". The *grossWeightSumCoverage* represents the number of half-DNBs which may map to this location, each weighted by their mapping weight ratio.

The reference score (*refScore*) is a measure of confidence that the base at that position is the same as that in the reference genome (such as a call of homozygous reference). The reference score is computed based on an examination of several alternate hypotheses, including all heterozygous SNPs and some single-base insertions and deletions at the position. Low reference scores are one method used to trigger the local *de novo* assembler.

As discussed elsewhere, the coverage numbers and reference scores are computed from the initial mapping results and not from the final *de novo* assemblies. The initial mappings have false negatives (reads that should align to a region but have significant degrees of difference) and false positives (alignments reported to a region that are due to repetitive DNA) that may be resolved by the later, more sensitive and specific algorithms used in *de novo* assembly.

Example *ASM/REF/coverageRefScore-[CHROMOSOME ID]-[ASM ID].tsv.bz2*

>offset	refScore	uniqueSequence Coverage	weightSumSequence Coverage	gcCorrected Coverage	grossWeightSumSequence Coverage
9411210	46	15	27	26	55
9411211	46	18	32	31	64
9411212	61	23	38	36	70
9411213	63	26	42	40	77
9411214	80	27	45	43	83
9411215	78	29	48	46	88
9411216	98	37	57	54	98
9411217	98	34	58	55	98

File-Specific Header DescriptionASM/REF/coverageRefScore-[CHROMOSOME ID]-[ASM ID].tsv.bz2

Key	Description	Allowed Values
#ASSEMBLY_ID	Name of the assembly	"<assembly-name>-ASM". For example, "GS000000474-ASM".
#CHROMOSOME	Identifier of the chromosome that the reference score and coverage data apply to. Data for the pseudo-autosomal regions on chromosome Y are reported at their coordinates on chromosome X.	chr1-chr22, chrM, chrX, chrY
#FORMAT_VERSION	Version number of the file format	Two or more digits separated by periods. For example, "0.6".
#GENERATED_AT	Date and time of the assembly	Year-Month-Day Time. For example "2010-Sep-08 20:27:52.457773".

Key	Description	Allowed Values
#GENERATED_BY	Assembly pipeline component that generated the output.	Alpha-numeric string.
#SAMPLE	Complete Genomics identifier of the sample from which the library was created	"GSXXXXX-DNA_YZZ" where<br>▪ X's are digits<br>▪ -DNA_ is literal<br>▪ YZZ is the location of the sample in a 96-well plate with Y as one of "A" through "H" and ZZ is one of "01" through "12"<br>For example "GS12345-DNA_A01".
#SOFTWARE_VERSION	Assembly pipeline build number	Two or more digits separated by periods.
#TYPE	Indicates the type of data contained in the file	"REFMETRICS": reference scores (scores indicating the likelihood of the assembled genome being identical to the reference at each genomic position) and coverage information.

Content Description ASM/REF/coverageRefScore-[CHROMOSOME ID]-[ASM ID].tsv.bz2

	Column Name	Description
1	offset	0-based position within chromosome for the base.
2	refScore	Reference score for the position. Positive values indicate greater confidence that the position is homozygous and identical to the reference genome.
3	uniqueSequenceCoverage	Coverage of this position by unique, fully mapping reads (both arms map with expected order, orientation and separation, and the weight of this mapping indicates only one high-probability mapping).
4	weightSumSequenceCoverage	Coverage of this position as determined by adding the weight ratio for each full DNB mapping covering this position. The weight ratio is a measure of the probability that the mapping is correct for this DNB.
5	gcCorrectedCoverage	Coverage of this position as determined by the weight-sum full DNB mapping covering this position, corrected for GC bias. The *gcCorrectedCoverage* is no-called ('N') in regions of the genome with very high or very low GC content.
6	grossWeightSumSequence-Coverage	Coverage of this position as determined by adding the weight ratio for all reads covering this position, whether or not their mates map. The weight ratio is a measure of the probability that the mapping is correct for this DNB.

Quality and Characteristics of Sequenced Genome Files

The REPORTS Directory contains information about the quality and characteristics of the sequenced genome organized in the following files:

- ***coverage-[ASM-ID].tsv*** and ***coverageCoding-[ASM-ID].tsv*** files provide unique and weight-sum sequence coverage, along with GC bias-corrected weight-sum coverage and gross weight-sum coverage, allowing you to assess the distribution of coverage across the whole genome or the autosomal exome, respectively.
- ***coverageByGcContent-[ASM-ID].tsv*** and ***coverageByGcContentCoding-[ASM-ID].tsv*** files report normalized coverage for cumulative GC base content percentile, allowing you to assess the level of GC bias across the whole genome or autosomal exome, respectively.
- ***indelLength-[ASM-ID].tsv***, ***indelLengthCoding-[ASM-ID].tsv***, ***substitutionLength-[ASM-ID].tsv***, and ***substitutionLengthCoding-[ASM-ID].tsv*** files report the size distribution of indel and substitution called genome-wide or in coding regions.
- ***circos-[ASM-ID].html*** and ***circos-[ASM-ID].png*** files provide a Circos visualization of small variations, CNVs, and Structural Variations identified in the sequenced genome, along with associated data such as Lesser Allele Fraction (LAF) and heterozygous and homozygous SNP density. The ***circosLegend.png*** file provides the legend that defines the data being visualized.

Figure 15: REPORTS Directory Contents

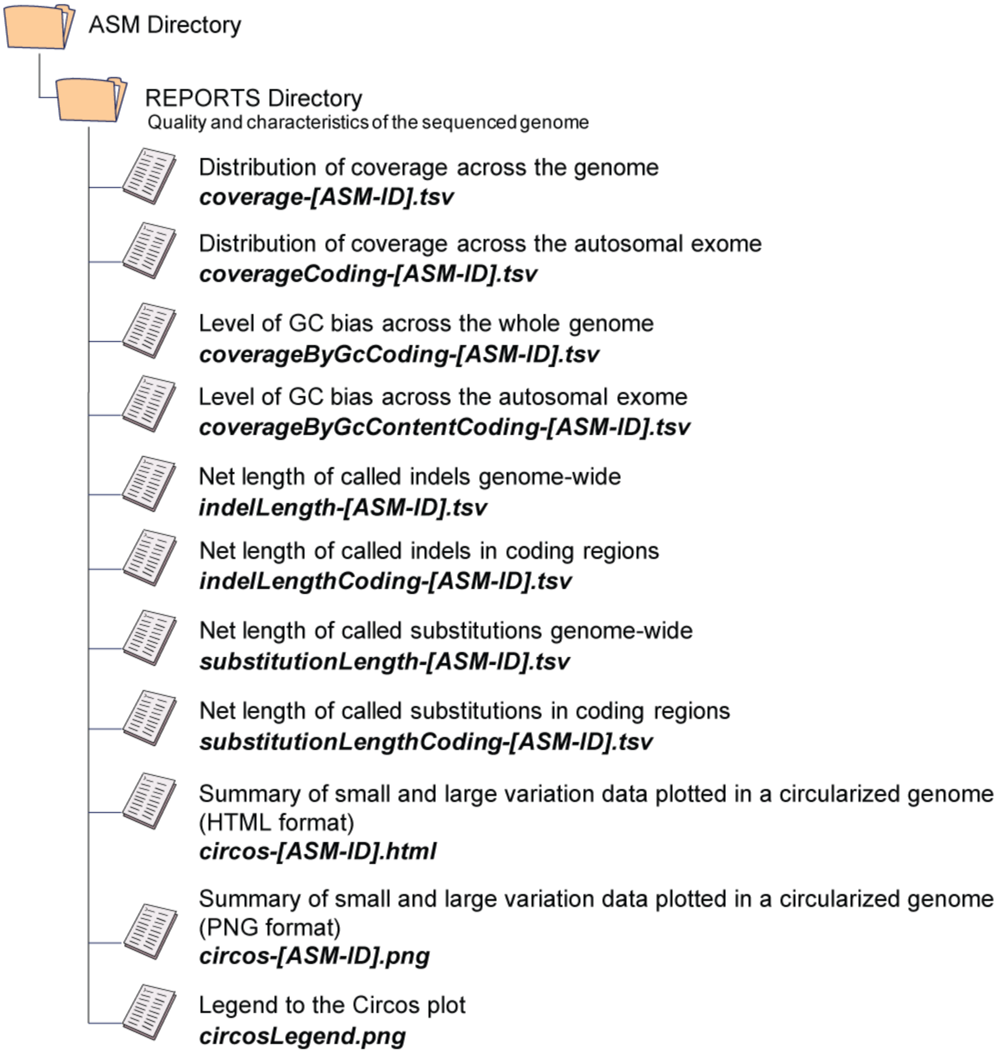

Coverage Distribution Report File

ASM/REPORTS/coverage-[ASM-ID].tsv and ASM/REPORTS/coverageCoding-[ASM-ID].tsv

Example ***ASM/REPORTS/coverage-[ASM-ID].tsv and ASM/REPORTS/coverageCoding-[ASM-ID].tsv***

>coverage	uniqueSequence Coverage	cumulativeUniqueSequence Coverage	weightSumSequence Coverage	cumulativeWeightSumSequence Coverage
0	33866120	33866120	8596485	8596485
1	10159591	44025711	2732635	11329120
2	7766133	51791844	2519762	13848882
3	6439176	58231020	2511738	16360620
4	5714158	63945178	2618900	18979520
5	5257558	69202736	2770202	21749722
6	4946507	74149243	2957547	24707269

Figure 16 shows how the data from the coverage file can be plotted to show the genome-wide distribution. Note that there are regions in the reference genome that have zero coverage. These regions represent:

- Highly repetitive sequence where a large number of mappings to the reference genome are marked as overflow and, therefore, do not contribute to coverage calculation,
- Sequence high in GC content, or
- Sequences that are present in the reference genome but are deleted in the population.

Figure 16: Plot of Genome-wide Coverage Distribution Generated from File *coverage-[ASM-ID].tsv*

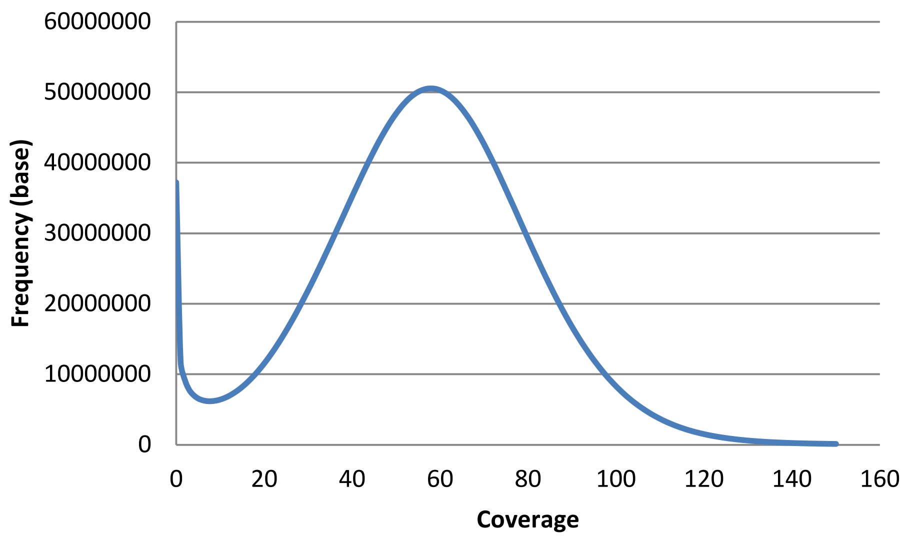

File-Specific Header Description ASM/REPORTS/coverage-[ASM-ID].tsv and ASM/REPORTS/coverageCoding-[ASM-ID].tsv

Key	Description	Allowed Values
#ASSEMBLY_ID	Name of the assembly	"<assembly-name>-ASM". For example, "GS000000474-ASM".
#SOFTWARE_VERSION	Assembly pipeline build number	Two or more digits separated by periods.
#GENERATED_BY	Assembly pipeline component that generated the output	Alpha-numeric string.
#GENERATED_AT	Date and time of the assembly	Year-Month-Day Time. For example "2010-Sep-08 20:27:52.457773".
#FORMAT_VERSION	Version number of the file format	Two or more digits separated by periods. For example, "0.6".
#GENOME_REFERENCE	Human genome build used for assembly	"NCBI build XX" where X's are digits.
#SAMPLE	Complete Genomics identifier of the sample from which the library was created	"GSXXXXX-DNA_YZZ" where<br>▪ X's are digits<br>▪ -DNA_ is literal<br>▪ YZZ is the location of the sample in a 96-well plate with Y as one of "A" through "H" and ZZ is one of "01" through "12"<br>For example "GS12345-DNA_A01"
#TYPE	Indicates the type of data contained in the file	For ***coverage-[ASM-ID].tsv*** file, "COVERAGE-DISTRIBUTION": Positive integer.<br>For ***coverageCoding-[ASM-ID].tsv*** file, "COVERAGE-DISTRIBUTION-CODING": Positive integer.

Content Description ASM/REPORTS/coverage-[ASM-ID].tsv and ASM/REPORTS/coverageCoding-[ASM-ID].tsv

	Column Name	Description
1	coverage	Number of bases in the reference genome covered (overlapped) by the number of uniquely mapping reads specified in the coverage column.
2	uniqueSequenceCoverage	Number of unique, fully mapping reads at a given coverage depth. In a fully mapping read, both arms map with expected order, orientation, and separation, and the weight of this mapping indicates only one high-probability mapping.
3	cumulativeUniqueSequenceCoverage	Cumulative number of unique, fully mapping reads at a given coverage depth.
4	weightSumSequenceCoverage	Number reads determined by adding the weight ratio for each full DNB mapping covering this position, at a given coverage depth. The weight ratio is a measure of the probability that the mapping is correct for this DNB. Here, reads are weighted by a mapping confidence factor between 0 and 1, where less unique mappings are assigned lower values.
5	cumulativeWeightSumSequence-Coverage	Cumulative number of reads determined by adding the weight ratio for each full DNB mapping covering this position, at a given coverage depth. Here, reads are weighted by a mapping confidence factor between 0 and 1, where less unique mappings are assigned lower values.

Coverage-by-GC-Content Report File

ASM/REPORTS/coverageByGcContent-[ASM-ID].tsv and ASM/REPORTS/coverageByGcContentCoding-[ASM-ID].tsv

The ***coverageByGcContent-[ASM-ID].tsv*** and ***coverageByGcContentCoding-[ASM-ID].tsv*** files report normalized coverage for cumulative GC base content percentile, allowing you to assess the level of GC bias across the genome.

Example ***ASM/REPORTS/coverageByGcContent-[ASM-ID].tsv and ASM/REPORTS/coverageByGcContentCoding-[ASM-ID].tsv***

>cumulativeBasePercentage	normalizedCoverage
7.04E-05	0.00041848
0.000150721	0.001128587
0.000270162	0.000607162
0.000395816	0.000735849
0.000517516	0.001430133
0.000656339	0.000832555
0.000832799	0.00080396
0.001006116	0.000821151
0.001195357	0.000979591

Figure 17 shows an example of a plot of normalized coverage across the spectrum of GC content seen in the genome generated from information contained in the ***coverageByGcContent-[ASM-ID].tsv*** file.

Figure 17: Unique Sequence Coverage by GC Content

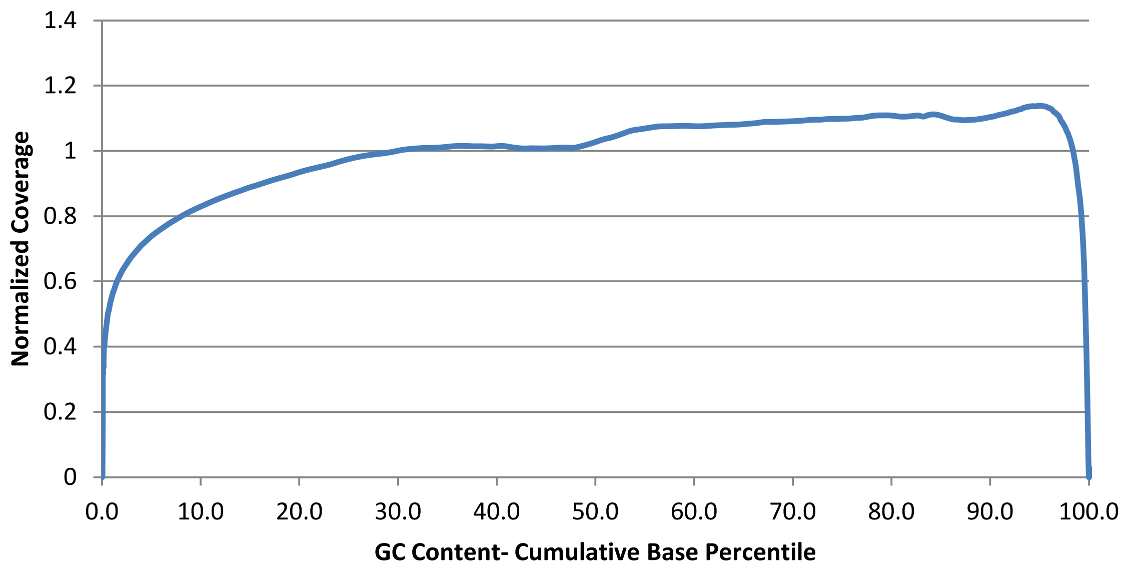

File-Specific Header Description *ASM/REPORTS/coverageByGcContent-[ASM-ID].tsv and ASM/REPORTS/coverageByGcContentCoding-[ASM-ID].tsv*

Key	Description	Allowed Values
#ASSEMBLY_ID	Name of the assembly	"<assembly-name>-ASM". For example, "GS000000474-ASM".
#SOFTWARE_VERSION	Assembly pipeline build number	Two or more digits separated by periods
#GENERATED_BY	Assembly pipeline component that generated the output.	Alpha-numeric string
#GENERATED_AT	Date and time of the assembly	Year-Month-Day Time. For example "2010-Sep-08 20:27:52.457773".
#FORMAT_VERSION	Version number of the file format	Two or more digits separated by periods. For example, "0.6".
#GENOME_REFERENCE	Human genome build used for assembly	"NCBI build XX" where X's are digits.
#SAMPLE	Complete Genomics identifier of the sample from which the library was created	"GSXXXXX-DNA_YZZ" where<br>▪ X's are digits<br>▪ -DNA_ is literal<br>▪ YZZ is the location of the sample in a 96-well plate with Y as one of "A" through "H" and ZZ is one of "01" through "12"<br>For example "GS12345-DNA_A01".
#WINDOW_WIDTH	Width, in bases, of windows in which GC content is calculated	Positive integer.
#TYPE	Indicates the type of data contained in the file	For ***coverageByGcContent-[ASM-ID].tsv*** file, "COVERAGE-BY-GC": Positive integer.<br>For ***coverageByGcContentCoding-[ASM-ID].tsv*** file, "COVERAGE-BY-GC-CODING": Positive integer.

Content Description *ASM/REPORTS/coverageByGcContent-[ASM-ID].tsv and ASM/REPORTS/coverageByGcContentCoding-[ASM-ID].tsv*

	Column Name	Description
1	cumulativeBasePercentage	GC content is computed in 501-bp windows. A GC bin at the 1st percentile indicates that 1% of genomic bases have this or lower %GC. A GC bin at the 99th percentile indicates that only 1% of genomic bases have higher GC content.
2	normalizedCoverage	Coverage normalized to genome-wide average.

Indel Net Length Report File

ASM/REPORTS/IndelLength-[ASM-ID].tsv

The ***IndelLength-[ASM-ID].tsv*** file reports the net length of called indels genome-wide.

Example — *ASM/REPORTS/IndelLength-[ASM-ID].tsv*

```
size     count
-6       5638
-5       8527
-4       27529
-3       20047
-2       44635
-1       148085
0        0
1        161119
2        36716
3        15460
4        20821
5        6454
6        3698
```

File-Specific Header Description — *ASM/REPORTS/IndelLength-[ASM-ID].tsv*

Key	Description	Allowed Values
#ASSEMBLY_ID	Name of the assembly	"<assembly-name>-ASM". For example, "GS000000474-ASM".
#SOFTWARE_VERSION	Assembly pipeline build number	Two or more digits separated by periods.
#GENERATED_BY	Assembly pipeline component that generated the output	Alpha-numeric string.
#GENERATED_AT	Date and time of the assembly	Year-Month-Day Time. For example "2010-Sep-08 20:27:52.457773".
#FORMAT_VERSION	Version number of the file format	Two or more digits separated by periods. For example, "0.6".
#GENOME_REFERENCE	Human genome build used for assembly	"NCBI build XX" where X's are digits.
#SAMPLE	Complete Genomics identifier of the sample from which the library was created	"GSXXXXX-DNA_YZZ" where<br>▪ X's are digits<br>▪ -DNA_ is literal<br>▪ YZZ is the location of the sample in a 96-well plate with Y as one of "A" through "H" and ZZ is one of "01" through "12"<br>For example "GS12345-DNA_A01".
#TYPE	Type of data contained in the file	"INDEL-LENGTH": positive and negative integer.

Content Description — *ASM/REPORTS/IndelLength-[ASM-ID].tsv*

Column Name	Description
size	Net length, in bases, of called insertions or deletions. Negative integer values indicate length of deletions, while positive integer values indicate length of insertions.
count	Number of insertions or deletions observed at that net length.

Indel Net Length in Coding Region Report File

ASM/REPORTS/IndelLengthCoding-[ASM-ID].tsv

The ***IndelLengthCoding-[ASM-ID].tsv*** file reports the net length of called indels in coding regions.

Example ***ASM/REPORTS/IndelLengthCoding-[ASM-ID].tsv***

```
size     count
-6       26
-5       2
-4       14
-3       72
-2       28
-1       85
0        0
1        87
2        13
3        53
4        4
5        3
6        12
```

Figure 18 plots the indel net length identified in the coding regions of the genome generated from information contained in the ***IndelLengthCoding-[ASM-ID].tsv*** file.

Figure 18: Indel Net Length in Coding Region

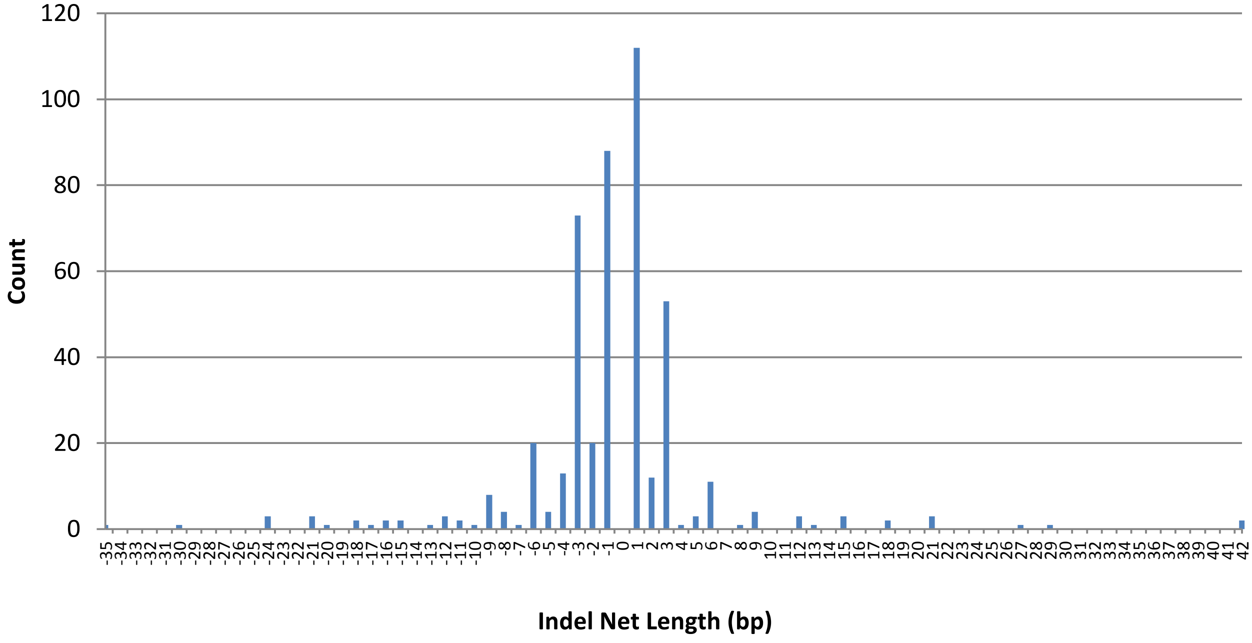

File-Specific Header Description ASM/REPORTS/IndelLengthCoding-[ASM-ID].tsv

Key	Description	Allowed Values
#ASSEMBLY_ID	Name of the assembly	"<assembly-name>-ASM". For example, "GS000000474-ASM".
#SOFTWARE_VERSION	Assembly pipeline build number	Two or more digits separated by periods.
#GENERATED_BY	Assembly pipeline component that generated the output	Alpha-numeric string.
#GENERATED_AT	Date and time of the assembly	Year-Month-Day Time. For example "2010-Sep-08 20:27:52.457773".
#FORMAT_VERSION	Version number of the file format	Two or more digits separated by periods. For example, "0.6".
#GENOME_REFERENCE	Human genome build used for assembly	"NCBI build XX" where X's are digits.
#SAMPLE	Complete Genomics identifier of the sample from which the library was created	"GSXXXXX-DNA_YZZ" where<br>▪ X's are digits<br>▪ -DNA_ is literal<br>▪ YZZ is the location of the sample in a 96-well plate with Y as one of "A" through "H" and ZZ is one of "01" through "12"<br>For example "GS12345-DNA_A01".
#TYPE	Type of data contained in the file	"INDEL-LENGTH-CODING": positive and negative integer.

Content Description ASM/REPORTS/IndelLengthCoding-[ASM-ID].tsv

Column Name	Description
size	Net length, in bases, of called insertions or deletions. Negative integer values indicate net length of deletions found in coding region of genome, while positive integer values indicate net length of insertions found in coding region genome.
count	Number of insertions or deletions observed at that net length.

Substitution Net Length File Report File

ASM/REPORTS/substitutionLength-[ASM-ID].tsv

The ***substitutionLength-[ASM-ID].tsv*** file reports the net length of called substitutions genome-wide.

Example *ASM/REPORTS/substitutionLength-[ASM-ID].tsv*

```
size     count
-6       772
-5       735
-4       1701
-3       1473
-2       3392
-1       8851
0        53101
1        7757
2        2563
3        1044
4        1101
5        543
6        462
```

File-Specific Header Description *ASM/REPORTS/substitutionLength-[ASM-ID].tsv*

Key	Description	Allowed Values
#ASSEMBLY_ID	Name of the assembly	"`<assembly-name>-ASM`". For example, "GS000000474-ASM".
#SOFTWARE_VERSION	Assembly pipeline build number	Two or more digits separated by periods.
#GENERATED_BY	Assembly pipeline component that generated the output.	Alpha-numeric string.
#GENERATED_AT	Date and time of the assembly	Year-Month-Day Time. For example "2010-Sep-08 20:27:52.457773".
#FORMAT_VERSION	Version number of the file format	Two or more digits separated by periods. For example, "0.6".
#GENOME_REFERENCE	Human genome build used for assembly	"`NCBI build XX`" where X's are digits.
#SAMPLE	Complete Genomics identifier of the sample from which the library was created	"`GSXXXXX-DNA_YZZ`" where<br>▪ X's are digits<br>▪ `-DNA_` is literal<br>▪ YZZ is the location of the sample in a 96-well plate with Y as one of "A" through "H" and ZZ is one of "01" through "12"<br>For example "GS12345-DNA_A01".
#TYPE	Type of data contained in the file	"`SUBSTITUTION-LENGTH`": Net length of called substitutions in the genome.

Content Description *ASM/REPORTS/substitutionLength-[ASM-ID].tsv*

Column Name	Description
size	Net length, in bases, of called substitutions. Negative and positive integers indicate length-changing substitutions, while 0 represents net length-conserving substitutions.
count	Number of substitutions observed at that net length.

Substitution Net Length in Coding Region Report File

ASM/REPORTS/substitutionLengthCoding-[ASM-ID].tsv

The ***substitutionLengthCoding-[ASM-ID].tsv*** file reports the net length of called substitutions in coding regions.

Example ***ASM/REPORTS/substitutionLengthCoding-[ASM-ID].tsv***

```
size	count
-6	0
-5	1
-4	0
-3	3
-2	3
-1	9
0	272
1	12
2	7
3	3
4	0
5	0
6	2
```

Figure 19 shows the substitution net length identified in the coding regions of the genome generated from information contained in the ***substitutionLengthCoding-[ASM-ID].tsv*** file.

Figure 19: Distribution of Substitution Net Length in Coding Regions

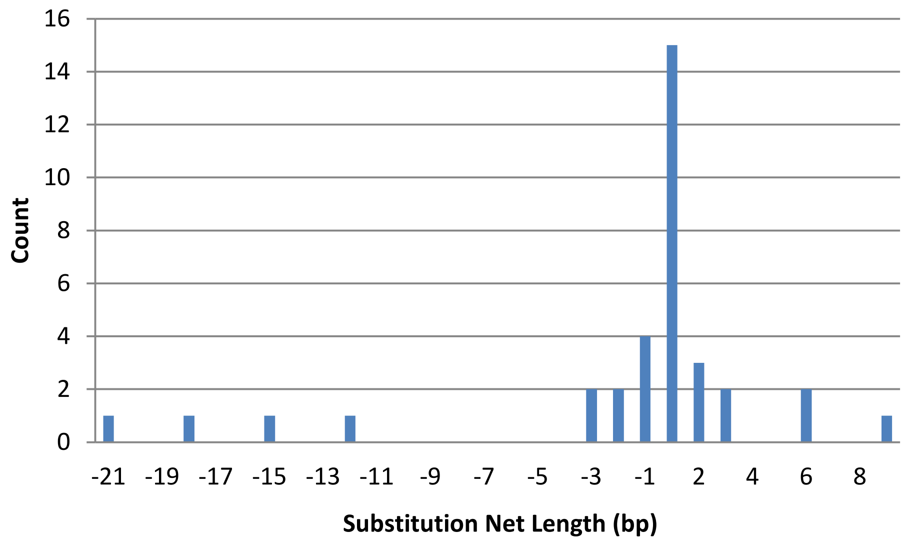

File-Specific Header Description ASM/REPORTS/substitutionLengthCoding-[ASM-ID].tsv

Key	Description	Allowed Values
#ASSEMBLY_ID	Name of the assembly	"<assembly-name>-ASM". For example, "GS000000474-ASM".
#SOFTWARE_VERSION	Assembly pipeline build number	Two or more digits separated by periods.
#GENERATED_BY	Assembly pipeline component that generated the output.	Alpha-numeric string.
#GENERATED_AT	Date and time of the assembly	Year-Month-Day Time. For example "2010-Sep-08 20:27:52.457773".
#FORMAT_VERSION	Version number of the file format	Two or more digits separated by periods. For example, "0.6".
#GENOME_REFERENCE	Human genome build used for assembly	"NCBI build XX" where X's are digits.
#SAMPLE	Complete Genomics identifier of the sample from which the library was created	"GSXXXXX-DNA_YZZ" where<br>▪ X's are digits<br>▪ -DNA_ is literal<br>▪ YZZ is the location of the sample in a 96-well plate with Y as one of "A" through "H" and ZZ is one of "01" through "12"<br>For example "GS12345-DNA_A01".
#TYPE	Type of data contained in the file	"SUBSTITUTION-LENGTH-CODING": positive and negative integer.

Content Description ASM/REPORTS/substitutionLengthCoding-[ASM-ID].tsv

Column Name	Description
size	Net length, in bases, of called substitutions. Negative and positive integers indicate net length-changing substitutions, while 0 represents length-conserving substitutions.
count	Number of substitutions observed at that net length.

Circos Visualization of Small Variations, CNVs, SVs, and Associated Data: Non-Tumor Sample

ASM/REPORTS/circos-[ASM-ID].html and ASM/REPORTS/circos-[ASM-ID].png

The ***circos-[ASM-ID].html*** and ***circos-[ASM-ID].png*** files provide a Circos visualization of variations detected in the genome, along with other associated data. The two files contain the same Circos plot, but the ***circos-[ASM-ID].html*** file includes a legend describing the layout of the Circos plot. The file ***circosLegend.png*** contains only the legend that describes the layout of the Circos plot.

Different genomic information is plotted for non-tumor and tumor samples (determined by 'tumor status' indicated in the Sample Manifest file when the sample is delivered to Complete Genomics). The following section describes the non-tumor files; the tumor files are described in "Tumor Genome Circos Visualization of Small Variations, CNVs, SVs, and Associated Data".

Example ASM/REPORTS/circos-[ASM-ID].html and ASM/REPORTS/circos-[ASM-ID].png

Figure 20: Non-Tumor Circos Visualization

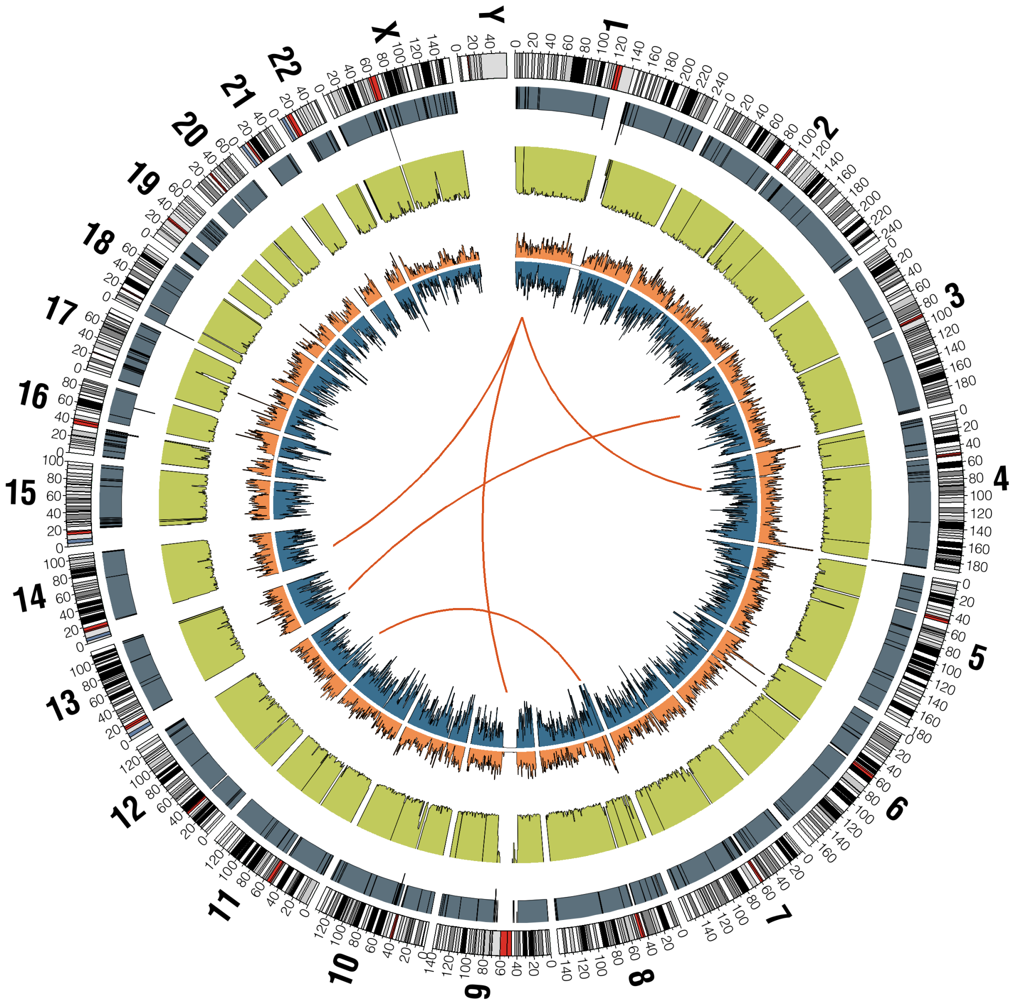

Example ASM/REPORTS/circosLegend.png

Figure 21: Non-Tumor Circos Visualization Legend

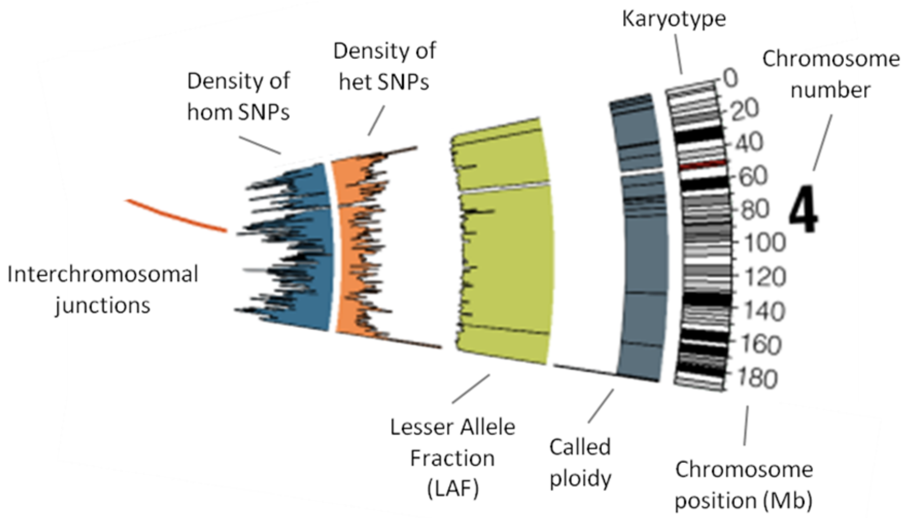

Content Description ASM/REPORTS/circosLegend.png

Label	Description
Interchromosomal junctions	Interchromosomal junctions identified in the sequenced genome. Only high-confidence interchromosomal junctions described in ***highConfidenceJunctionsBeta*** file are plotted.
Density of hom SNPs	Density of high confidence homozygous SNPs in 1Mb windows, arbitrarily scaled in a histogram with y-axis pointing inward.
Density of het SNPs	Density of heterozygous SNPs in 1Mb windows, arbitrarily scaled in a histogram with y-axis pointing outward.
Lesser Allele Fraction (LAF)	Single-sample LAF estimate for 100 kb windows, with y-axis scale of 0 to 0.5, pointing inward. Estimates are based on read counts at all fully-called variant loci.
Called ploidy	CNV called ploidy from ***cnvSegmentsBetaDiploid*** file. Arbitrarily scaled with Y-axis pointing inward.
Karyotype	Standard Circos ideogram depicting chromosome position and chromosome number.
Chromosome position	Reference coordinate along the chromosome.
Chromosome number	Chromosome number: 1, 2,...,22, X, Y.

Tumor Genome Circos Visualization of Small Variations, CNVs, SVs, and Associated Data

ASM/REPORTS/circos-[ASM-ID].html and ASM/REPORTS/circos-[ASM-ID].png

The ***circos-[ASM-ID].html*** and ***circos-[ASM-ID].png*** file provides a Circos visualization of variations detected in the Tumor Genome, along with other associated data. The two files contain the same Circos plot, but the ***circos-[ASM-ID].html*** file includes a legend describing the layout of the Circos plot. The file ***circosLegend.png*** contains only the legend that describes the layout of the Circos plot.

Different genomic information is plotted for non-tumor and tumor samples (determined by 'tumor status' indicated in the Sample Manifest file when the sample is delivered to Complete Genomics). The following section describes the tumor files; the non-tumor files are described in "Circos Visualization of Small Variations, CNVs, SVs, and Associated Data: Non-Tumor Sample".

Example ASM/REPORTS/circos-[ASM-ID].html and ASM/REPORTS/circos-[ASM-ID].png

Figure 22: Tumor Circos Visualization

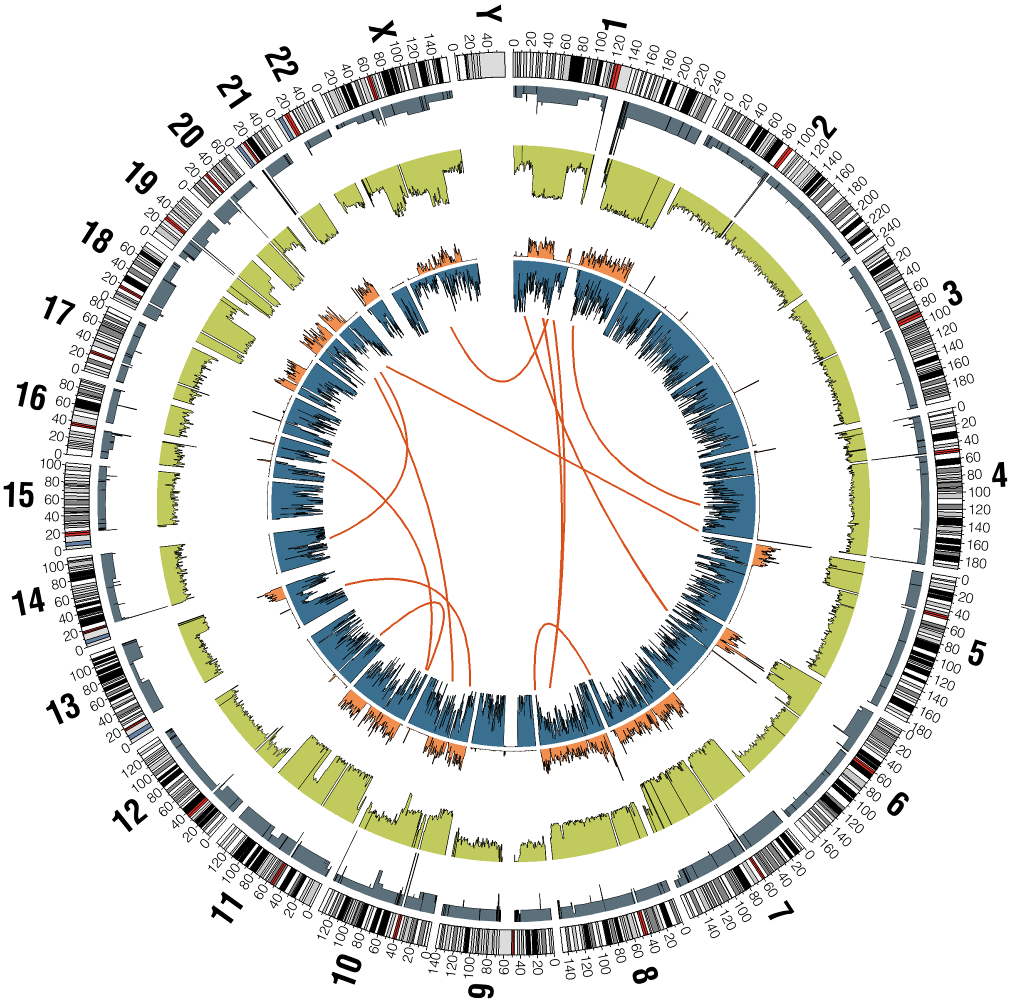

Example *ASM/REPORTS/circosLegend.png*

Figure 23: Tumor Circos Visualization Legend

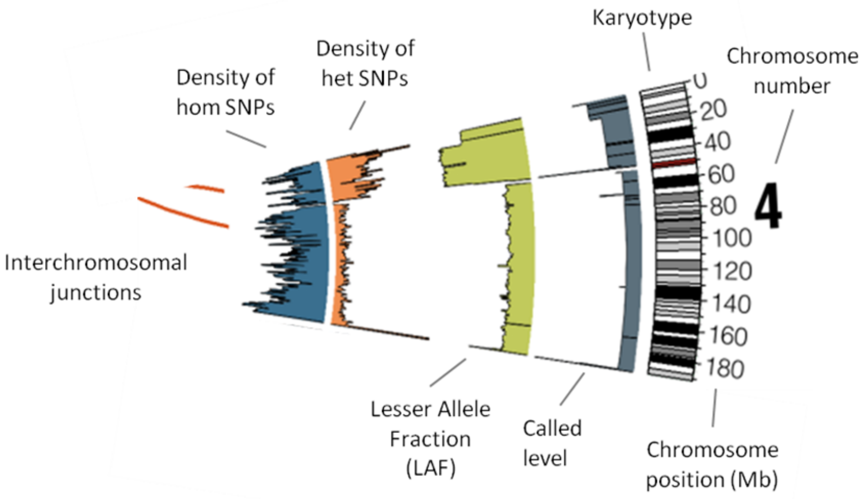

Content Description *ASM/REPORTS/circosLegend.png*

The labels are described from inside the circle toward the outside.

Label	Description
Interchromosomal junctions	Interchromosomal junctions identified in the sequenced genome. Only high-confidence interchromosomal junctions described in ***highConfidenceJunctionsBeta*** file are plotted.
Density of hom SNPs	Density of high confidence homozygous SNPs in 1Mb windows, arbitrarily scaled in a histogram with the Y-axis pointing inward.
Density of het SNPs	Density of heterozygous SNPs in 1Mb windows, arbitrarily scaled in a histogram with Y-axis pointing outward.
Lesser Allele Fraction (LAF)	Single-sample LAF estimate for 100 kb windows, with Y-axis scale of 0 to 0.5, pointing inward. Estimates are based on read counts at all fully-called variant loci.
Called level	CNV called level from ***cnvSegmentsBetaNonDiploid*** file. Arbitrarily scaled with the Y-axis pointing inward.
Karyotype	Standard Circos ideogram depicting chromosome position and chromosome number.
Chromosome position	Reference coordinate along the chromosome.
Chromosome number	Chromosome number: 1, 2,…,22, X, Y.

Library Information

The LIB directory contains a subdirectory which houses files that provide information about the genomic DNA clone library used during assembly.

Figure 24: LIB Directory Contents

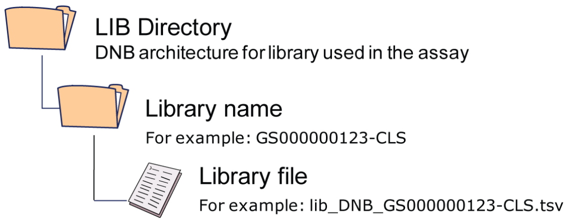

Architecture of Reads and Gaps

LIB/lib_DNB_[LIBRARY-NAME].tsv

The file ***lib_DNB_[LIBRARY-NAME].tsv*** describes the architecture of reads and gaps within all DNBs in the library. The information is useful in the interpretation of reads in ***reads_[SLIDE-LANE]_00X.tsv***. The DNB is described as a series of objects of different types (reads, gaps, mate gap) sequentially following one another.

Example — *LIB/lib_DNB_[LIBRARY-NAME].tsv*

>id	type	armId	indArm	objArm	min	max
0	read	0	0	0	10	10
1	gap	0	1	0	-9	-1
2	read	0	2	1	10	10
3	gap	0	3	1	-1	-1
4	read	0	4	2	10	10
5	mategap	0	5	0	116	873
6	read	1	0	0	10	10
7	gap	1	1	0	-9	-2
8	read	1	2	1	10	10
9	gap	1	3	1	-1	-1
10	read	1	4	2	10	10

File-Specific Header Description — *LIB/lib_DNB_[LIBRARY-NAME].tsv*

Key	Description	Allowed Values
#ASSEMBLY_ID	Name of the assembly.	"<assembly-name>-ASM". For example, "GS000000474-ASM".
#FORMAT_VERSION	Version number of the file format.	Two or more digits separated by periods. For example, "0.6".
#GENERATED_AT	Date and time of the assembly.	Year-Month-Day Time. For example "2010-Sep-08 20:27:52.457773".
#GENERATED_BY	Assembly pipeline component that generated the output.	Alpha-numeric string.
#LIBRARY	Identifier of the library from which the DNBs were generated.	
#SAMPLE	Complete Genomics identifier of the sample from which the library was created.	"GSXXXXX-DNA_YZZ" where<br>▪ X's are digits<br>▪ -DNA_ is literal<br>▪ YZZ is the location of the sample in a 96-well plate with Y as one of "A" through "H" and ZZ is one of "01" through "12"<br>For example "GS12345-DNA_A01".
#SOFTWARE_VERSION	Assembly pipeline build number.	Two or more digits separated by periods.
#TYPE	Indicates the type of data contained in the file.	"LIB-DNB": description of the architecture of reads within DNBs in a library.

Content Description LIB/lib_DNB_[LIBRARY-NAME].tsv

Column Name	Description	Text Format
id	Position of the object within each DNB, numbered from 0 to n-1, where n is the number of objects (reads and gaps) within each DNB.	int
type	Object type: currently one of `read`, `gap`, or `mategap`	string
armID	Number of the half-DNB: 0-left, 1-right.	int
indArm	0-based position of the object within an arm.	int
objArm	0-based position of this object type within an arm, e.g. the second gap within the second arm has “1” for this field.	int
min	Minimum length in bases for the object.<br>N.B. The minimum and maximum values for mate gaps given in this table exclude the most extreme 0.05% of values on either end of the observed distribution. The values for small gaps in this table describe the minimum and maximum values observed in the most frequent small gap tuples for the given arm, accounting for 99.9% of observations.	int
max	Maximum length in bases for the object. Blank when maximum is not specified.<br>**Note**: The minimum and maximum values for mate gaps given in this table exclude the most extreme 0.05% of values on either end of the observed distribution. The values for small gaps in this table describe the minimum and maximum values observed in the most frequent small gap tuples for the given arm, accounting for 99.9% of observations.	int

Empirically Observed Mate Gap Distribution

LIB/lib_gaps_M_[LIBRARY-NAME].tsv

The ***lib_gaps_M_[LIBRARY-NAME].tsv*** file describes the empirically observed mate gap distribution for the library.

Example — *LIB/lib_gaps_M_[LIBRARY-NAME].tsv*

>mateGap	frequency
196	1.72E-06
197	1.72E-06
198	1.20E-05
199	2.28E-05
200	3.37E-05
201	4.63E-05
202	5.79E-05

File-Specific Header Description — *LIB/lib_gaps_M_[LIBRARY-NAME].tsv*

Key	Description	Allowed Values
#ASSEMBLY_ID	Name of the assembly.	"<assembly-name>-ASM". For example, "GS000000474-ASM".
#FORMAT_VERSION	Version number of the file format.	Two or more digits separated by periods. For example, "0.6".
#GENERATED_AT	Date and time of the assembly.	Year-Month-Day Time. For example "2010-Sep-08 20:27:52.457773".
#GENERATED_BY	Assembly pipeline component that generated the output.	Alpha-numeric string.
#LIBRARY	Identifier of the library from which the DNBs were generated.	
#SAMPLE	Complete Genomics identifier of the sample from which the library was created.	"GSXXXXX-DNA_YZZ" where<br>▪ X's are digits<br>▪ -DNA_ is literal<br>▪ YZZ is the location of the sample in a 96-well plate with Y as one of "A" through "H" and ZZ is one of "01" through "12"<br>For example "GS12345-DNA_A01".
#SOFTWARE_VERSION	Assembly pipeline build number.	Two or more digits separated by periods.
#TYPE	Indicates the type of data contained in the file.	"LIB-MATE-GAPS": describes the empirically observed mate gap distribution for the library.

Content Description — *LIB/lib_gaps_M_[LIBRARY-NAME].tsv*

Column Name	Description
mateGap	The number of genomic bases between the two arms of the DNB.
frequency	The fraction of DNBs observed to have the given mate gap.

Empirical Intraread Gap Distribution

LIB/lib_gaps_rollup_[ARM]_[LIBRARY-NAME].tsv

The ***lib_gaps_rollup_[ARM]_[LIBRARY-NAME].tsv*** file describes the frequency of observation of gap tuples for the given arm for the library. A gap tuple is a set of gap values for all the small gaps in the arm.

Example *LIB/lib_gaps_rollup_[ARM]_[LIBRARY-NAME].tsv*

>gaps	frequency
-3;-1	0.591405
-4;-1	0.278702
-2;-1	0.102601
-5;-1	0.00759106
-9;-1	0.00535504
-8;-1	0.00443704
-7;-1	0.00375003
-1;-1	0.00316403
-6;-1	0.00299502

File-Specific Header Description *LIB/lib_gaps_rollup_[ARM]_[LIBRARY-NAME].tsv*

Key	Description	Allowed Values
#ASSEMBLY_ID	Name of the assembly.	"<assembly-name>-ASM". For example, "GS000000474-ASM".
#FORMAT_VERSION	Version number of the file format.	Two or more digits separated by periods. For example, "0.6".
#GENERATED_AT	Date and time of the assembly.	Year-Month-Day Time. For example "2010-Sep-08 20:27:52.457773".
#GENERATED_BY	Assembly pipeline component that generated the output.	Alpha-numeric string.
#LIBRARY	Identifier of the library from which the DNBs were generated.	
#SAMPLE	Complete Genomics identifier of the sample from which the library was created.	"GSXXXXX-DNA_YZZ" where<br>▪ X's are digits<br>▪ -DNA_ is literal<br>▪ YZZ is the location of the sample in a 96-well plate with Y as one of "A" through "H" and ZZ is one of "01" through "12"<br>For example "GS12345-DNA_A01".
#SOFTWARE_VERSION	Assembly pipeline build number.	Two or more digits separated by periods.
#TYPE	Indicates the type of data contained in the file.	"LIB-SMALL-GAPS-ROLLUP": describes the frequency of observation of gap tuples for the given arm for the library.

Content Description *LIB/lib_gaps_rollup_[ARM]_[LIBRARY-NAME].tsv*

Column Name	Description
gaps	Semicolon-separated list of the small gaps in the arm, in DNB order.
frequency	The fraction of DNBs observed to have the given gaps.

Sequence-dependent Empirical Intraread Gap Distribution

LIB/lib_gaps_[ARM][ID]_[LIBRARY-NAME].tsv

The ***lib_gaps_[ARM][ID]_[LIBRARY-NAME].tsv*** file describes the frequency of observation of small gap values depending on nearby genomic sequence for the given arm for the library.

Example *LIB/lib_gaps_[ARM][ID]_[LIBRARY-NAME].tsv*

The first section of the example shows the first six columns of the ***lib_gaps*** file; the remaining four columns appear in the lower section, with the sequence values repeated at the left edge to more easily match the data with the previous section of data; the sequence values are not repeated in the actual data.

>sequence:23-28;firstGap:0;gapCount:2	gaps:-9;-1	gaps:-8;-1	gaps:-7;-1	gaps:-6;-1	gaps:-5;-1
AAAAA	0.004755	0.003129	0.002112	0.001603	0.005494
AAAAC	0.004872	0.003631	0.003211	0.002481	0.007825
AAAAG	0.004612	0.003038	0.002074	0.001798	0.005847
AAAAT	0.004846	0.003275	0.002452	0.001774	0.006209
AAAAN	0.004769	0.003228	0.00237	0.001823	0.006126
AAACA	0.003628	0.00271	0.001803	0.001681	0.004927

	gaps:-4;-1	gaps:-3;-1	gaps:-2;-1	gaps:-1;-1
AAAAA	0.2334	0.7179	0.03073	0.0008607
AAAAC	0.3793	0.4757	0.1228	0.0001874
AAAAG	0.1976	0.6993	0.08549	0.0002345
AAAAT	0.3158	0.5669	0.09843	0.0003832
AAAAN	0.2726	0.6336	0.07492	0.0004959
AAACA	0.07049	0.9112	0.001255	0.002339

File-Specific Header Description *LIB/lib_gaps_[ARM][ID]_[LIBRARY-NAME].tsv*

Key	Description	Allowed Values
#ASSEMBLY_ID	Name of the assembly.	"<assembly-name>-ASM". For example, "GS000000474-ASM".
#FORMAT_VERSION	Version number of the file format.	Two or more digits separated by periods. For example, "0.6".
#GENERATED_AT	Date and time of the assembly.	Year-Month-Day Time. For example "2010-Sep-08 20:27:52.457773".
#GENERATED_BY	Assembly pipeline component that generated the output.	Alpha-numeric string.
#LIBRARY	Identifier of the library from which the DNBs were generated.	
#SAMPLE	Complete Genomics identifier of the sample from which the library was created.	"GSXXXXX-DNA_YZZ" where<br>▪ X's are digits<br>▪ -DNA_ is literal<br>▪ YZZ is the location of the sample in a 96-well plate with Y as one of "A" through "H" and ZZ is one of "01" through "12"<br>For example "GS12345-DNA_A01".
#SOFTWARE_VERSION	Assembly pipeline build number.	Two or more digits separated by periods.
#TYPE	Indicates the type of data contained in the file.	"LIB-SEQDEP-GAPS": describes the frequency of observation of small gap values depending on nearby genomic sequence for the given arm for the library.

Content Description *LIB/lib_gaps_[ARM][ID]_[LIBRARY-NAME].tsv*

Column Name	Description
sequence:[sequenceStart]-[sequenceEnd];firstGap:[N];gapCount:[M]	Here “sequenceStart” is the 0-based number of bases from the clone end (toward the mate gap) of the sequence start, or for [ID] > 0, the number of bases from the end of the last gap described in the previous gaps file. The “sequenceEnd” is one past the end of the sequence, using the same coordinate system as sequenceStart. [N] and [M] determine which gaps are described by the file. They are gap offsets in order from the end of the clone. The data rows for this column contain base sequence. The sequence data is the genomic sequence in order from the clone end, on the same strand as the clone strand for the left arm, and on the opposite strand for the right arm. This facilitates analysis of gap frequency asymmetries in otherwise symmetric DNB architectures. The sequence may have N’s in which case the gap frequencies are rollups.
gaps:[Gap N];…;[Gap N+M-1]	This header describes a gap tuple, and the data values describe the frequency of occurrence for that gap tuple, given the sequence. Here, “Gap N” is the gap value for gap N.

Reads and Mapping Data

The MAP Directory contains reads, scores, and initial alignments to the reference genome for each DNB. Data is organized by slide and lane. Each subdirectory name is the identifier for the lane, for example "GS08089-FS3-L01" would represent data for the first lane (L01) of the slide "GS08089-FS3".

Figure 25: MAP Directory Contents

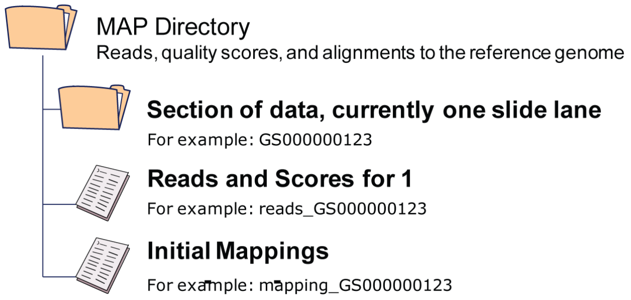

Reads and mappings are split within each lane to keep the data below a 5 GB file size threshold. A lane directory containing: ***reads_[SLIDE-LANE]_001.tsv.bz2***, ***reads_[SLIDE-LANE]_002.tsv.bz2***, and ***reads_[SLIDE-LANE]_003.tsv.bz2*** will have corresponding mappings files: ***mapping_[SLIDE-LANE]_001.tsv.bz2***, ***mapping_[SLIDE-LANE]_002.tsv.bz2***, and ***mapping_[SLIDE-LANE]_003.tsv.bz2***. Previously, reads and mappings were contained in single files.

The following sections describe the files in each lane subdirectory within the MAP Directory.

Reads and Quality Scores

MAP/reads_[SLIDE-LANE]_00X.tsv.bz2

The file ***reads_[SLIDE-LANE]_00X.tsv.bz2*** is a tab-delimited text file (compressed with bzip2) containing the reads and associated quality scores.

Example — *MAP/reads_[SLIDE-LANE]_00X.tsv.bz2*

This example shows a sample set of rows from a ***reads_[SLIDE-LANE]_00X.tsv*** file for hypothetical DNBs of length 20, showing the ASCII-33-encoded, single-character quality scores. DNBs with the structure illustrated in Figure 1 would have 60 bases and corresponding scores, with the first 30 bases corresponding to the left half-DNB and the last 30 bases to the right half-DNB.

>flags	reads	scores
1	AGTGAGACACCTGAGGGNGA	SXXX<NDUETSUBTMW]!\Z
4	AAATATATTTTGTAGTCNAG	PKMZH@+E6CN)KJ)[)!Z5
0	CTTCTCTGGTTTATTGTNTG	UXW6XTTP/R(0MST3[!],

The interpretations of all allowed values for the *flags* field are described in Figure 26:

Figure 26: Allowed flag Field Values

**flags**	**0**	**1**	**2**	**4**	**5**	**6**	**8**	**9**	**10**
LeftHalfDnbNoMatches		x			x			x	
LeftHalfDnbMapOverflow			x			x			x
RightHalfDnbNoMatches				x	x	x			
RightHalfDnbMapOverflow							x	x	x

A value of flags = 0 indicates that both arms of the DNB mapped to the reference genome. If a flag other than 0 is set the corresponding arm has no mappings in the mapping file. For example, a flag of 4 (no matches) or 8 (overflow) indicates mappings are only available for the left arm and not the right.

File-Specific Header Description — *MAP/reads_[SLIDE-LANE]_00X.tsv.bz2*

**Key**	**Description**	**Allowed Values**
#ASSEMBLY_ID	Name of the assembly	"`<assembly-name>-ASM`". For example, "GS000000474-ASM".
#BATCH_FILE_NUMBER	Number of the batch of a split data file	Positive 1-based integer.
#BATCH_OFFSET	Offset of the first record in a batch to the position of the record in a non-split file	Positive 0-based integer.
#FIELD_SIZE	Size of the lane fields	Positive integer.
#FORMAT_VERSION	Version number of the file format	Two or more digits separated by periods. For example, "0.6".
#GENERATED_AT	Date and time of the assembly	Year-Month-Day Time. For example "2010-Sep-08 20:27:52.457773".
#GENERATED_BY	Assembly pipeline component that generated the output	Alpha-numeric string.
#LANE	Identifier of the slide lane from which the reads were extracted	
#LIBRARY	Identifier of the library from which the DNBs were generated	

Key	Description	Allowed Values
#SAMPLE	Complete Genomics identifier of the sample from which the library was created	"GSXXXXX-DNA_YZZ" where<br>▪ X's are digits<br>▪ -DNA_ is literal<br>▪ YZZ is the location of the sample in a 96-well plate with Y as one of "A" through "H" and ZZ is one of "01" through "12"<br>For example "GS12345-DNA_A01".
#SLIDE	Flow slide identification code	
#SOFTWARE_VERSION	Assembly pipeline build number	Two or more digits separated by periods.
#TYPE	Indicates the type of data contained in the file	"READS": reads file.

Content Description — *MAP/reads_[SLIDE-LANE]_00X.tsv.bz2*

Name	Description	Text Format
flags	Mapping characteristics of the DNBs, represented in bits within an integer. Individual flags described below.	Integer (base 10), for example "8".
reads	The base calls read from a single DNB, in an order specified in ***lib_DNB_[LIBRARY-ID].tsv***. Base positions for which no information is available are denoted by "N" in the *reads* field.	One character per base, not separated.
scores	Quality scores for reads. Each score is a Phred-like transformation of the error probability associated with a single base read. Base positions for which no information is available are assigned a score of 0.	One ASCII-33-encoded character per base, not separated. The Phred quality score can be inferred from the ASCII code of the displayed character. For example, a score of "A" has the ASCII code 65, and a Phred quality score of 65 – 33 = 32. This corresponds to a discordance probability of 10 (32/10) = 0.00063.
flag: LeftHalfDnbNoMatches	The left half of this DNB yielded no mappings to the reference genome.	0x01
flag: LeftHalfDnbMapOverflow	The left half of this DNB yielded a large number of mappings to the reference genome [indicative of highly repetitive sequence; mappings not tracked for this half-DNB].	0x02
flag: RightHalfDnbNoMatches	The right half of this DNB yielded no mappings to the reference genome.	0x04
flag: RightHalfDnbMapOverflow	The right half of this DNB yielded a large number of mappings to the reference genome (indicative of highly repetitive sequence; mappings not tracked for this half-DNB).	0x08

Initial Mappings

MAP/mapping_[SLIDE-LANE]_00X.tsv.bz2

The file ***mapping_[SLIDE-LANE]_00X.tsv.bz2*** is a tab-separated text file containing initial mapping information to the reference genome (compressed with bzip2) for the reads in ***reads_[SLIDE-LANE]_00X.tsv.bz2.*** Each row of the ***mapping_[SLIDE-LANE]_00X.tsv.bz2*** file corresponds to the alignment of a single half-DNB to the reference genome, with information on the most likely mate for this half-DNB. This file does not contain the bases and scores for each read. However, the mappings for each read are stored sequentially and in the same order as in ***reads_[SLIDE-LANE]_00X.tsv.bz2***. This format does not allow for random access to a genomic location, and retrieval of reads and mappings corresponding to one or several genomic regions would require a full scan of both files.

Example — *MAP/mapping_[SLIDE-LANE]_00X.tsv.bz2*

In accordance with the column definitions, flags that are odd numbers signify the last mapping record for a DNB. In this example, mappings for four DNBs are shown:

- For the first DNB, there is one mapping available for each half-DNB, with both close to one another on chromosome 18. The *mateRec* field for the two half-DNB mappings is populated with 1 and 0 respectively, indicating that these two are best mates for one another. Based on the flags values of 0 and 3, it is shown that both half-DNBs map to the forward strand.
- For the fourth and last DNB, there is only one mapping available. Based on flags = 5, it can be inferred that it is a mapping of the left half-DNB to the reverse strand of the reference genome. The *offsetInChr* field (representing the starting coordinate of the mapping, in zero-based half-open coordinates described in "Sequence Coordinate System") and *gap* fields are described with respect to the forward strand, however, and not in the order of the bases in ***reads_[SLIDE-LANE]_00X.tsv.bz2***. That is, for the DNB architecture represented in Figure 1, the bases in this reverse-strand-mapped, left half-DNB map to the right of *offsetInChr*. Because no mate mapping was found for this half-DNB, *mateRec* is populated with its own record position within the mappings for the DNB, which is 0.
- The third DNB has one mapping available for each half-DNB on chromosome 7, both on the reverse strand based on the values of flags. Again, *mateRec* indicates that the two mappings are mated with one another.
- The second DNB, represented in rows 3 – 8 of the example, has six, half-DNB mappings. The *mateRec* field values for these rows indicate that this DNB has three pairs of mated mappings on the genome: one each on chromosomes 7, 8, and 19. For example, the record numbers of the two chromosome 7 mappings within the set for this DNB are 0 and 3; the *mateRec* fields in these records are 3 and 0 respectively. The values of flags indicate that the first three rows (rows 3 – 5 in the example) correspond to the left half-DNB and the next three rows (rows 6 – 8 in the example) correspond to the right half-DNB; they also indicate that the chromosome 19 mappings are to the reverse strand.

Figure 27: Example Initial Mapping File *mapping_[SLIDE-LANE]_00X.tsv.bz2*

0	>flags	chromosome	offsetInChr	gap1	gap2	gap3	weight	mateRec	armWeight
1	0	chr18	54911965	-2	0	5	(	1	!
2	3	chr18	54912325	5	0	-3	(	0	!
3	0	chr7	92578954	-2	0	6	!	3	!
4	0	chr8	59803146	-2	0	6	!	4	!
5	4	chr19	19695620	4	0	-2	!	5	!
6	2	chr7	92579332	6	0	-3	!	0	!
7	2	chr8	59803538	6	0	-3	!	1	!
8	7	chr19	19695239	-3	0	6	!	2	!
9	4	chr7	101416273	6	1	-2	L	1	L
10	7	chr7	101415891	-2	0	5	L	0	L
11	5	chr8	85763053	5	0	-2	j	0	j

File-Specific Header Description MAP/mapping_[SLIDE-LANE]_00X.tsv.bz2

Key	Description	Allowed Values
#ASSEMBLY_ID	Name of the assembly	"<assembly-name>-ASM". For example, "GS000000474-ASM".
#BATCH_FILE_NUMBER	Number of the batch of a split data file	Positive 1-based integer.
#FORMAT_VERSION	Version number of the file format	Two or more digits separated by periods. For example, "0.6".
#GENERATED_AT	Date and time of the assembly	Year-Month-Day Time. For example "2010-Sep-08 20:27:52.457773".
#GENERATED_BY	Assembly pipeline component that generated the output	Alpha-numeric string.
#LANE	Identifier of the slide lane from which the reads were extracted	
#LIBRARY	Identifier of the library from which the DNBs were generated	
#SAMPLE	Complete Genomics identifier of the sample from which the library was created	"GSXXXXX-DNA_YZZ" where<br>▪ X's are digits<br>▪ -DNA_ is literal<br>▪ YZZ is the location of the sample in a 96-well plate with Y as one of "A" through "H" and ZZ is one of "01" through "12"<br>For example "GS12345-DNA_A01".
#SLIDE	Flow slide identification code	
#SOFTWARE_VERSION	Assembly pipeline build number	Two or more digits separated by periods.
#TYPE	Indicates the type of data contained in the file	"MAPPINGS": alignments of reads to the reference genome

Content Description MAP/mapping_[SLIDE-LANE]_00X.tsv.bz2

Column Name	Description	Text format
flags	Mapping characteristics encoded in bit fields, described in Table 11.	integer
chromosome	Chromosome name in text: chr1, chr2,...,chr22, chrX, chrY.<br>The pseudoautosomal regions within the sex chromosomes X and Y are reported at their coordinates on chromosome X.	
offsetInChr	Starting coordinate on chromosome, 0-based (see "Sequence Coordinate System" for more information).	
gap1 .. gap[n]	There are *n* tab-separated gap fields, where *n* is the number of gaps in the half-DNB as defined in ***lib_DNB_[LIBRARY-NAME].tsv***. Currently n = 2; that is, there are 2 gaps per half-DNB. The column contains the length of each gap within the half-DNB. Gaps are listed in order of chromosomal position. Overlaps are represented as negative numbers.	integer
weight	Mapping weight. This is a Phred encoding of the probability that this half-DNB mapping is incorrect. For DNBs with no consistent mappings, this column matches the *armWeight* column. For DNBs with consistent mappings, the probability that this half-DNB mapping is incorrect is computed using information about which mappings have a consistent mate mapping, whereas the *armWeight* always uses the information about mappings for that arm in isolation.	ASCII-33

Column Name	Description	Text format
mateRec	Zero-based index of the best mate for the current half-DNB, counting within the half-DNB mappings for the current DNB. Equals the index of the current mapping if no mate mappings are found.	integer
armWeight	Mapping weight. This is a Phred encoding of the probability that this half-DNB mapping is incorrect, independent of the mappings of its mate.	ASCII-33
flag: LastDNBRecord	Set if the current mapping is last mapping record of the DNB	0x01
flag: side	The arm within the DNB that yielded this mapping. The left arm (that is, the first half of the bases in the *reads* column of ***reads_[SLIDE-LANE]_00X.tsv.bz2***) is represented by 0; the right arm (the second half of the bases in the *reads* column of ***reads_[SLIDE-LANE]_00X.tsv.bz2***) is represented by 1., Right – 1	0x02
flag: strand	forward - 0, reverse – 1	0x04

The allowed values for the *flags* field in ***mapping_[SLIDE-LANE]_00X.tsv.bz2*** and their interpretation are shown in Table 11.

Table 11: Initial Mapping File Flags Field Values

flags	LastDNBRecord	side	strand
0	no	Left	+
1	yes	Left	+
2	no	Right	+
3	yes	Right	+
4	no	Left	-
5	yes	Left	-
6	no	Right	-
7	yes	Right	-

Association between Initial Mappings and Reads Data

DNB mappings in ***mapping_[SLIDE-LANE]_00X.tsv*** are stored in the same order as records for DNBs in the ***reads_[SLIDE-LANE]_00X.tsv*** file, allowing for an association between them. Within a DNB, all left-arm mappings precede right-arm mappings. The number of mapping records corresponding to each DNB is variable, and flags within the two files help to associate records within the two with each other.

The ***reads_[SLIDE-LANE]_00X.tsv*** file includes read and score data for each DNB that passes basic quality filters. The flags corresponding to each DNB contain information on whether each of its constituent half-DNBs yielded mappings to the reference genome. There are three possibilities for each DNB:

1. If either *LeftHalfDnbNoMatch* or *LeftHalfDnbMapOverflow* is set to 1, no mapping records are expected for the left half-DNB in ***mapping_[SLIDE-LANE]_00X.tsv***.
2. If either *RightHalfDnbNoMatch* or *RightHalfDnbMapOverflow* is set to 1, no mapping records are expected for the right half-DNB in ***mapping_[SLIDE-LANE]_00X.tsv***.
3. The last half-DNB mapping record in ***mapping_[SLIDE-LANE]_00X.tsv*** corresponding to this read will have the *LastDNBRecord* flag set to 1, indicating that the next mapping record corresponds to a new DNB.

Using these rules, it is possible to scan the ***mapping_[SLIDE-LANE]_00X.tsv*** and ***reads_[SLIDE-LANE]_00X.tsv*** files together, associating the mappings in ***mapping_[SLIDE-LANE]_00X.tsv*** with reads and scores in ***reads_[SLIDE-LANE]_00X.tsv***. Mappings are associated with the next record in ***reads_[SLIDE-LANE]_00X.tsv*** following a record with the *LastDNBRecord* flag set to 1; however, records in ***reads_[SLIDE-LANE]_00X.tsv*** for which no mappings are expected, due to rules (1) and (2) above, are skipped.

Glossary

allele (as used in variations file)

An arbitrary designation of one diploid allele over another in a variations file.

dB (decibel)

A log scale used by Complete Genomics for expressing probabilities and odds. dB are well known to bioinformaticians as the basis of the "Phred scale": 10 dB means the likelihood ratio is 10:1, 20 dB means 100:1, 30 dB is 1000:1, etc. Formally, the value of an odds-ratio
R= P1/P2 expressed in dB is 10 x log10 R.

In cases where dB is used to encode an error probability P (as in a basecall quality score or a mis-mapping probability) the score is expressed as -10 x log10 P. In both cases bigger scores in dB are "better".

In all putatively variant regions, the assembler considers many hypotheses (essentially, possible consensus sequences) and computes probabilities of the observed read data under each these hypotheses. We perform a likelihood ratio test between the most likely hypothesis and the next most likely, and we express this score in decibels (dB). The variant scores factor in quantity of evidence (read depth), quality of evidence (base call quality values), and mapping probabilities. The column header for the variation score is "total score" in the variations file.

Scores for variants are not calibrated on an absolute scale to error rate. A score of 30 dB does not necessarily indicate that the P(error)=0.001.

20 dB is presently the minimum score for calling a homozygous variant and 40 dB is the minimum for a heterozygous variant. Based on empirical testing, these thresholds were chosen to balance call-rate and accuracy.

DNB

DNA Nano Ball, an individual library construct. The role of DNBs is roughly equivalent to that of "clones" in many other platforms.

DNB Arm

One end of a DNB insert sequence, from either side of the mate-pair gap. The DNB Arm may be called an "end" or "read end" or "paired end" on other platforms.

evidence

The assembly underlying a small variant call. It includes the final allele sequences called, and for each the alignments of the supporting DNB to that sequence.

evidence interval

The coordinates on the reference genome corresponding to an assembled region.

indel

Short for "Insertion or Deletion".

initial mapping

By comparison with some other pipelines used with other types of data, the Complete Genomics bioinformatics process involves an initial mapping followed by a refinement of these mappings by local de novo assembly. The assemblies, and not the initial mappings, represent the final determination of the location of a DNB. See "Complete Genomics Service FAQ" for more information.

Lesser Allele Fraction (LAF)

When two alleles are present at a site, the lesser allele fraction is the part of the sample containing one of the alleles, specifically the one that is present in 50% or less of the sample. For pure, diploid samples, heterozygous SNPs have an allele fraction of 0.5 for each allele. When samples are not pure (heterogeneous) or not diploid, alleles at heterozygous sites will be greater than and less than 0.5. LAF represents the allele fraction for the allele present at ≤ 50% of the sample.

locus (as used in variations file)

A region of the genome containing variations on either or both alleles. An arbitrary threshold is used to determine when nearby variations are part of the same loci or separate loci.

no-call-rc, no-call-ri

All no-call variant types indicate that the sequence could not be fully resolved, either because of limited or no information, or because of contradictory information. When some portions of the allele sequence can be called but others not, we will indicate this as "no-call-rc" (no-call, reference-consistent) if those called portions are the same as the reference. We use no-call-ri (no-call, reference-inconsistent) if they are not.

In some cases, one may wish to be conservative and consider any such region entirely no-called, and thus neither a match nor a mismatch between sample and reference.

read Gap, mate gap

Complete Genomics reads have two kinds of gaps as shown in Figure 1. There are two positions in each DNB arm where the bases overlap in the source DNA: these are intra-read negative gaps. Also, there is a larger mate-gap region (approx. 350-400 bp) in between the two reads from one DNB, as is the case for other paired-end and mate-pair sequencing methods.

refScore, Reference Score

Complete Genomics computes a value called the reference score reported in the ***coverageRefScore*** file. This score indicates whether the corresponding mapped reads are consistent with the reference sequence (positive values) or not (negative values). This score is an excellent predictor for the strength of evidence for homozygous reference calls.

Similar to the method by which variant scores are computed, the reference score is the log-odds ratio of P(ref) over P(non-ref), expressed in dB, where the P(non-ref) involves examining only a limited number of alternate hypotheses. These include all possible SNPs at every position in homozygous and heterozygous form, plus, at selected positions, one-base insertions and deletions, as well as some changes in homopolymer length. This computation is performed based on the initial mapping results and, like the variation scores, is not precisely calibrated to P(error). Reference scores are also not precisely calibrated to variation scores.

In spite of the lack of calibration, a reference score in one sample can be considered against the variation score of another sample to assist in sample-sample comparison, particularly when asking whether a variant seen in one sample might be a false negative in another.

varScoreEAF

Complete Genomics computes a value called the varScoreEAF. This score indicates whether the corresponding mapped reads are consistent with called variant. It is derived from the probability estimates under maximum likelihood equal allele fraction model. Specifically, it is equal to

$$10 * \log 10\left(\frac{\mathrm{P(Call\ is\ true)}}{\mathrm{P(Call\ is\ false)}}\right)$$

Although this score is not calibrated, we provide a means for researchers to calibrate these score, by providing a set of files that resulted from replicate calibration. For more details, see *Complete Genomics Small Variations Methods*.

varScoreVAF

Complete Genomics computes a value called the varScoreVAF. This score indicates whether the corresponding mapped reads are consistent with called variant. It is derived from the probability estimates under maximum likelihood variable allele fraction model. Specifically, it is equal to

$$10 * \log 10\left(\frac{\mathrm{P(Call\ is\ true)}}{\mathrm{P(Call\ is\ false)}}\right)$$

Although this score is not calibrated, we provide a means for researchers to calibrate these score, by providing a set of files that resulted from replicate calibration. For more details, see *Complete Genomics Small Variations Methods*.

sub

A "sub" is a block substitution where a series of reference bases are replaced with another series of bases. This event may or may not be length conserving.

© Complete Genomics, Inc.
